# Description of Staphylococcal Strains from Straw-Coloured Fruit Bat (*Eidolon helvum*) and Diamond Firetail (*Stagonopleura guttata*) and a Review of their Phylogenetic Relationships to Other Staphylococci

**DOI:** 10.3389/fcimb.2022.878137

**Published:** 2022-05-11

**Authors:** Stefan Monecke, Frieder Schaumburg, Adebayo O. Shittu, Stefan Schwarz, Kristin Mühldorfer, Christian Brandt, Sascha D. Braun, Maximilian Collatz, Celia Diezel, Darius Gawlik, Dennis Hanke, Helmut Hotzel, Elke Müller, Martin Reinicke, Andrea T. Feßler, Ralf Ehricht

**Affiliations:** ^1^ Leibniz Institute of Photonic Technology (IPHT), Jena, Germany; ^2^ InfectoGnostics Research Campus, Jena, Germany; ^3^ Institute of Medical Microbiology, University Hospital Münster, Münster, Germany; ^4^ Department of Microbiology, Obafemi Awolowo University, Ile-Ife, Nigeria; ^5^ Institute of Microbiology and Epizootics, Freie Universität Berlin, Berlin, Germany; ^6^ Veterinary Centre for Resistance Research (TZR), Freie Universität Berlin, Berlin, Germany; ^7^ Department of Wildlife Diseases, Leibniz Institute for Zoo and Wildlife Research, Berlin, Germany; ^8^ Institute for Infectious Diseases and Infection Control, Jena University Hospital, Jena, Germany; ^9^ Illumina GmbH, Berlin, Germany; ^10^ Friedrich-Loeffler-Institut (Federal Research Institute for Animal Health), Institute of Bacterial Infections and Zoonoses, Jena, Germany; ^11^ Institute of Physical Chemistry, Friedrich-Schiller-University, Jena, Germany

**Keywords:** *Staphylococcus aureus*, *Staphylococcus schweitzeri*, *Staphylococcus argenteus*, *Staphylococcus singaporensis*, *Staphylococcus roterodami*, whole genome sequencing (WGS), DNA microarray

## Abstract

The phylogenetic tree of the *Staphylococcus aureus* complex consists of several distinct clades and the majority of human and veterinary *S. aureus* isolates form one large clade. In addition, two divergent clades have recently been described as separate species. One was named *Staphylococcus argenteus*, due to the lack of the “golden” pigment staphyloxanthin. The second one is *S. schweitzeri*, found in humans and animals from Central and West Africa. In late 2021, two additional species, *S. roterodami* and *S. singaporensis*, have been described from clinical samples from Southeast Asia. In the present study, isolates and their genome sequences from wild Straw-coloured fruit bats (*Eidolon helvum*) and a Diamond firetail (*Stagonopleura guttata*, an estrildid finch) kept in a German aviary are described. The isolates possessed staphyloxanthin genes and were closer related to *S. argenteus* and *S. schweitzeri* than to *S. aureus*. Phylogenetic analysis revealed that they were nearly identical to both, *S. roterodami* and *S. singaporensis*. We propose considering the study isolates, the recently described *S. roterodami* and *S. singaporensis* as well as some Chinese strains with MLST profiles stored in the PubMLST database as different clonal complexes within one new species. According to the principle of priority we propose it should be named *S. roterodami*. This species is more widespread than previously believed, being observed in West Africa, Southeast Asia and Southern China. It has a zoonotic connection to bats and has been shown to be capable of causing skin and soft tissue infections in humans. It is positive for staphyloxanthin, and it could be mis-identified as *S. aureus* (or *S. argenteus*) using routine procedures. However, it can be identified based on distinct MLST alleles, and “*S. aureus*” sequence types ST2470, ST3135, ST3952, ST3960, ST3961, ST3963, ST3965, ST3980, ST4014, ST4075, ST4076, ST4185, ST4326, ST4569, ST6105, ST6106, ST6107, ST6108, ST6109, ST6999 and ST7342 belong to this species.

## Introduction

The phylogenetic tree of bacteria traditionally considered *Staphylococcus (S.) aureus* consists of several distinct clades. Most human and veterinary *S. aureus* isolates from all over the world form one large clade. In addition, two divergent clades have recently been elevated to the status of species (Tong et al., 2015; Becker et al., 2019). One has been named *S. argenteus*, due to its lack of the “golden” carotenoid pigment staphyloxanthin (Holt et al., 2011) regarded as a unique property of *S. aureus*. Isolates assigned or re-assigned to this new species have been described in many countries including Australia (Ng et al., 2009), Thailand (Indrawattana et al., 2019; Pumipuntu, 2019), Laos (Yeap et al., 2017), Cambodia (Ruimy et al., 2009), Myanmar (Aung et al., 2019), Japan (Ohnishi et al., 2018; Aung et al., 2019; Kitagawa et al., 2020), China and Taiwan (Chen et al., 2018), on Indian Ocean islands such as the Comoros or Mayotte (Dupieux et al., 2015), Gabon (Schuster et al., 2017), Trinidad and Tobago (Monecke et al., 2014) and Brazil (Rossi et al., 2020). Sporadic isolates also have been identified in the United Arab Emirates (Senok et al., 2020), several European countries (Dupieux et al., 2015; Rigaill et al., 2018; Tång Hallbäck et al., 2018; Diot et al., 2020; Kukla et al., 2020; Söderquist et al., 2020) as well as in Canada and the United States of America (Eshaghi et al., 2021). These observations could be associated with travel and migration. Aside from the lack of the carotenoid pigment gene cluster, *S. argenteus* isolates carry the same genes as *S. aureus*, albeit they occur as distinct allelic variants (Monecke et al., 2010), and its genes are located in the genome following the same order as in *S. aureus*. Traditional PCR-based multilocus sequence typing (MLST) can be performed on *S. argenteus* using slightly modified primers (Ng et al., 2009; Ruimy et al., 2009; Holt et al., 2011) and as in *S. aureus*, the resulting sequence types (ST) cluster into closely related groups known as clonal complexes (CCs). Moreover, CC affiliation also correlates with the presence or absence of certain genomic islands essentially identical to their counterparts in *S. aureus*. Thus, ST1223, ST1850 (formerly ST75), ST2198, ST2250, ST2596/2793 (Aung et al., 2019; Hsu et al., 2020), ST2854 and ST4587 can be regarded as founders of homonymous CCs. Some mobile genetic elements from *S. aureus* have also been identified in *S. argenteus*. This includes SCC*mec* IV and V elements that carry the methicillin/beta-lactam resistance gene *mecA*, phages harbouring the Panton-Valentine leukocidin gene (Dupieux et al., 2015; Aung et al., 2017; Senok et al., 2020) and the pathogenicity-island-borne *tst1* [encoding toxic shock syndrome toxin 1 (Aung et al., 2017)]. *S. argenteus* can asymptomatically be carried in the nares. It also can cause the same types of infections as *S. aureus* (Becker et al., 2019), i.e., skin and soft tissue infections (Ohnishi et al., 2018), osteomyelitis (Rigaill et al., 2018) or endoprosthesis infections (Diot et al., 2020; Söderquist et al., 2020) and sepsis (Chen et al., 2018; Kitagawa et al., 2020). *S. argenteus* also has been implicated in food poisoning (Suzuki et al., 2017). Some lineages of *S. argenteus* have been identified in animals such as rabbits (Indrawattana et al., 2019), dairy cattle (Pumipuntu, 2019; Rossi et al., 2020) and a wild gorilla (Schuster et al., 2017).

The other entity, *S. schweitzeri* consists of several sequence types (ST1857, ST1872, ST1873, ST1874, ST2022, ST2058, ST2059, ST2067, ST2071, ST2463, ST2464, ST2465, ST2467, ST3952, ST3958, ST3960, ST3961, ST3962, ST3963, ST3980, ST4316, ST4326, ST5117, ST5600 and ST5602). The alleles of core genome genes of *S. schweitzeri* are distinct from those of *S. aureus* and *S. argenteus*. However, published genome sequences of *S. schweitzeri* and experiments with DNA microarrays (Okuda et al., 2016) indicate that certain genomic island genes (*agr* alleles, capsule type, *egc, cna, seh*, carotenoid locus genes) closely resemble their *S. aureus* counterparts. The presence of these genes in *S. schweitzeri* is related to their CC affiliation, as also noted in *S. aureus*. So far, *S. schweitzeri* has been observed in four different regions. It was first identified in Gabon (Tong et al., 2015) from where it was also reported in other studies (Schaumburg et al., 2012; Schaumburg et al., 2015; Okuda et al., 2016) as well as in Côte d’Ivoire (Schaumburg et al., 2012; Schaumburg et al., 2015), Nigeria (Akobi et al., 2012) and the Democratic Republic of Congo, DRC (Schaumburg et al., 2015). Most of these isolates originated from non-human primates (Schaumburg et al., 2012) or from “bush-meat”, i.e., poached or hunted wildlife sold on local markets (Schaumburg et al., 2015). Some isolates from healthy humans have been identified, suggesting that humans carry this lineage sporadically and asymptomatically (Tong et al., 2015; Schaumburg et al., 2015; Okuda et al., 2016). In Nigeria, *S. schweitzeri* was recovered from faecal samples of the Straw-coloured fruit bats (*Eidolon helvum*) on the premises of a university campus (Akobi et al., 2012). This observation caused concerns of zoonotic transmission as these isolates were also detected on fomites in the same university (Shittu et al., 2020). However, a transmission of *S. schweitzeri* from animals to humans has not yet been observed. A large study from the DRC, Gabon and Côte d’Ivoire investigated rural populations and did not identify *S. schweitzeri* among humans despite close contact with bushmeat and wildlife (Schaumburg et al., 2015). The pathogenicity of *S. schweitzeri* remains unclear as humans were found to be colonised rather than infected (Schaumburg et al., 2015; Okuda et al., 2016). However, *in vitro* experiments suggest that *S. schweitzeri* is as virulent as *S. aureus* (Grossmann et al., 2021). Generally, one might assume that it is a zoonotic species that might asymptomatically colonise humans (Becker et al., 2019) and appears to be restricted to Central/West Africa. However, more data are needed to assess the distribution and a possible clinical significance of *S. schweitzeri.*

In autumn 2021, two new species of “*S. aureus*-like” staphylococci were described from human samples. These were named *S. roterodami* and *S. singaporensis* (Schutte et al., 2021; Chew et al., 2021). A single isolate of *S. roterodami* was identified from an infected wound of a Dutch traveller returning from Bali, Indonesia, prompting bacteriological investigations and genome sequencing (Schutte et al., 2021). A study (Chew et al., 2021) investigating a possible presence of *S. argenteus/schweitzeri* in Singapore identified 37 *S. argenteus* and six “unknowns” assigned to five novel STs and described as a new species, *S. singaporensis*. Four of these six isolates were associated with skin and soft tissue infections.

We describe a group of animal isolates submitted to the authors´ laboratories for characterisation as suspected *S. argenteus* or *S. schweitzeri.* These were characterised and sequenced. We also review their relationship to *S. aureus, S. argenteus, S. schweitzeri, S. roterodami* and *S. singaporensis.*

## Material and Methods

### Animals and Isolates

Seven isolates originated from faecal samples of the Straw-coloured fruit bat (*Eidolon helvum*), collected on a university campus in Ile-Ife, Nigeria, for earlier studies (Akobi et al., 2012; Olatimehin et al., 2018). The eighth isolate was recovered from a pulmonary specimen of a captive Diamond firetail (*Stagonopleura guttata*), an estrildid finch. The bird was kept in an aviary in a zoological collection in Berlin, Germany. The carcass of the deceased bird was submitted for necropsy. Lung tissue samples revealing disseminated white to yellowish miliary lesions were subsequently forwarded for microbiological investigations with suspected avian mycobacteriosis, and indeed pulmonary smears were positive for acid-fast bacilli.

An overview of isolates and typing data is provided in **Table 1**. The isolates were characterised by microarray (see below and **Supplemental File 1**). Three isolates, two from bats and the one from the finch were selected for phenotypic characterisation and whole-genome sequencing (WGS).

**Table 1:** Details of animals and isolates described herein (bold font), as well as of related isolates described otherwise or listed in the MLST database.

Isolate ID	Host	Sample type	Collected	Reference	Location	MLST	Comments
**BDS-53B**	Straw-coloured fruit bat, *Eidolon helvum*	Faecal	2016		Student Union Building, Obafemi Awolowo University, Ile-Ife, Nigeria	ST3965 (272-616-543-190-268-447-389)	
**BDS-53E**	*Eidolon helvum*	Faecal	2016		Student Union Building, Obafemi Awolowo University, Ile-Ife, Nigeria	ST4326 (272-616-543-190-268-499-537)	*spa* type t16757 (741-12-96-17-16-371)
**BDS-54**	*Eidolon helvum*	Faecal	2016		Student Union Building, Obafemi Awolowo University, Ile-Ife, Nigeria	ST3963 (272-357-306-190-268-448-548)	
**BDS-69C**	*Eidolon helvum*	Faecal	2016		Student Union Building, Obafemi Awolowo University, Ile-Ife, Nigeria	ST3952 (272-603-543-190-268-447-37)	*spa* type t17074 (26-22-17-20-17-13-12-17-17-16-16)
**BDH-128**	*Eidolon helvum*	Faecal	2015	(Olatimehin et al., 2018)	Health Centre, Obafemi Awolowo University, Ile-Ife, Nigeria	ST3961 (272-357-306-190-268-448-277)	*spa* type t16748 (741-12-96-17-16-371)
**BDH-147**	*Eidolon helvum*	Faecal	2015	(Olatimehin et al., 2018)	Health Centre, Obafemi Awolowo University, Ile-Ife, Nigeria	ST3960 (272-603-543-190-268-447-537)	*spa* type t17079 (26-23-23-13-23-31-29-17-25-16-28-17-25-17-25-16-28)
**BDH-157**	*Eidolon helvum*	Faecal	2015		Health Centre, Obafemi Awolowo University, Ile-Ife, Nigeria	ST3980 (272-357-306-190-268-448-37)	*spa* type t16747 (741-12-17-17-17-16-371)
**Zoo-28**	Diamond firetail*, S. guttata*	Pulmonary sample			Tierpark Berlin; Germany	ST7342 (723-888-907-571-868-807-830)	*spa* type t16114 (712-12-713-17-25-16-371)
BDS-92	*Eidolon helvum*	Faecal	2016	PUBMLST ID 32390	Health Centre, Obafemi Awolowo University, Ile-Ife, Nigeria	ST4014 (272-616-543-190-488-447-11)	*spa* type t16757 (742-743)
AOS157Y	Steering wheel of a car	Fomite		(Shittu et al., 2020)	Health Centre, Obafemi Awolowo University, Ile-Ife, Nigeria	ST3961 (272-357-306-190-268-448-277)	
R20	Bat	Faecal	2008	PUBMLST ID 5861	Ile-Ife, Nigeria	ST3135 (349-357-400-240-356-342-389)	
F16	*Eidolon helvum*	Faecal		PUBMLST ID 4779 (Akobi et al., 2012);	Ile-Ife, Nigeria	ST2470 (272-357-306-190-268-270-277)	
EMCR19	Human, *Homo sapiens*	Wound swab		(Schutte et al., 2021), GenBank CAJGUT01	Netherlands/Bali	ST6999 (818-1013-883-553-836-778-939)	*“S. roterodami”*
SS21	*Homo sapiens*			(Chew et al., 2021), GenBank JABWHB	Singapore	ST6105 (722-884-803-214-743-684-828)	*“S. singaporensis”*
SS35	*Homo sapiens*			(Chew et al., 2021), GenBank NZ_JABWPO	Singapore	ST6106 (722-885-805-214-744-685-831)	*“S. singaporensis”*
SS60	*Homo sapiens*			(Chew et al., 2021), GenBank NZ_JABWHF	Singapore	ST6107 (723-886-804-214-745-686-830)	*“S. singaporensis”*
SS87	*Homo sapiens*			(Chew et al., 2021), GenBank NZ_JABWHE	Singapore	ST6108 (722-887-806-481-746-684-829)	*“S. singaporensis”*
SS90 and SS251	*Homo sapiens*			(Chew et al., 2021), GenBank NZ_JABWHD and NZ_JABWHC	Singapore	ST6109 (724-888-807-481-747-684-277)	*“S. singaporensis”*
Sta1873	Food sample			PUBMLST ID 32453	Guangzhou, China	ST4075 (476-4-1-315-500-469-555)	
YNSA-323	Food			PUBMLST ID 32733	Yunnan, China	ST4185 (476-421-562-315-500-469-555)	
3574A1	Food			PUBMLST ID 33253	Guangzhou, China	ST4569 (532-1-1-315-567-513-617)	
Sta1874	Food			PUBMLST ID 32454	Guangzhou, China	ST4076 (475-4-1-315-499-465-553)	*spa* type t11641
SA1	*Homo sapiens*	Wound swab	2015	PUBMLST ID 32428	Rio de Janeiro, Brazil	ST4051 (403-1-1-190-1-1-1)	Combines CC1 MLST alleles with *S. roterodami*-like *gmk* sequence.
78085	*Homo sapiens*	Skin swab	2011	PUBMLST ID 5812	Denmark	ST3089 (349-57-45-2-7-58-52)	*mecC*-MRSA. Combines CC130 MLST alleles with *S. roterodami-*like *arcC* sequence.
3245	Food sample			PUBMLST ID 33090	Guangzhou, China	ST4466 (5-4-1-315-4-6-3)	Combines CC7 MLST alleles with *S. roterodami*-like *gmk* sequence. *spa* type t796.
TXA, TXBA140, A1404N, A1404W, A1524, A1525, A109, Z1403, K990W	Rhesus, *Macaca mulatta*,Long-tailed macaques, *M. fascicularis*, Southern pig-tailed macaque, *M. nemestrina*	Nasal and wound swabs	2015	(Soge et al., 2016; Roberts et al., 2018), SAMN04362246, SAMN04362247	Seattle, USA (animals imported from Asia)	ST3268 (1-14-430-214-10-303-329)	*spa* type t13638, SCC*mec* V*/*VT. Combines possibly CC45-like MLST alleles with a *S. roterodami*-like *gmk* sequence.
Several isolates	*M. fascicularis, Homo sapiens*	Surgical site, nasal and perianal swab	2014	(Hsu et al., 2017)	Singapore (imported animals as well as human contacts)	ST2817 (1-14-360-214-10-303-329) and ST3268 (1-14-430-214-10-303-329)	SCC*mec* V. Combines possibly CC45-like MLST alleles with a *S. roterodami*-like *gmk* sequence.
Several isolates	*Macaca mulatta, M. fascicularis*	Faecal	2017	(Li et al., 2020)	Shanghai, China (imported animals)	ST3268 (1-14-430-214-10-303-329)	*spa* type t13638, SCC*mec* V. Combines possibly CC45-like MLST alleles with a *S. roterodami*-like *gmk* sequence.

### Antimicrobial Susceptibility Testing and Biochemical Tests

Antimicrobial susceptibility testing was performed by the Vitek 2 automated system (bioMérieux, Nuertingen, Germany; **Supplemental File 2**) using the AST-P608 panel (benzylpenicillin, oxacillin, cefoxitin, gentamicin, tobramycin, ciprofloxacin, levofloxacin, moxifloxacin, erythromycin, clindamycin incl. inducible resistance, linezolid, teicoplanin, vancomycin, tetracycline, fosfomycin, nitrofurantoin, fusidic acid, mupirocin, rifampicin) according to manufacturer´s instructions. EUCAST clinical breakpoints for *S. aureus* (https://www.eucast.org/fileadmin/src/media/PDFs/EUCAST_files/Breakpoint_tables/v_11.0_Breakpoint_Tables.pdf) were applied to classify the isolates as susceptible, intermediate or resistant. *S. aureus* ATCC^®^ 29213 served as quality control strain. In addition, biochemical tests were performed using the bioMérieux Gram-positive identification card (GP) for the same device.

### MLST and *spa* Typing

MLST is based on sequencing seven housekeeping genes, *arcC, aroE, glpF, gmk, pta, tpi* and *yqiL.* Sequencing of PCR products was performed as previously described (Enright et al., 2000), or the sequences of the target genes were extracted from assembled whole-genome sequence data. The sequences were assigned to MLST alleles and STs using the *S. aureus* section (https://pubmlst.org/bigsdb?db=pubmlst_saureus_seqdef&page=sequenceQuery ) of the PubMLST website (Jolley et al., 2018).

In addition, *spa* typing was performed as previously described (Harmsen et al., 2003) using repeat definitions and nomenclature as provided on the Ridom website (http://spa.ridom.de/).

### Microarray-Based Genotyping

Isolates were characterised using the DNA microarray-based kit (Interarray *S. aureus*, fzmb GmbH, Research Centre for Medical Technology and Biotechnology, Bad Langensalza, Germany). Primer and probe sequences have been published previously (Monecke et al., 2008; Monecke et al., 2011). The array covers 333 different targets related to approximately 170 different genes and their allelic variants allowing detection of virulence and resistance factors. Isolates were assigned to clonal complexes (CCs) by automated comparison to a reference database. The procedures followed the manufacturer’s instructions as previously described (Monecke et al., 2008; Monecke et al., 2011). Briefly, *S. aureus* was cultured overnight on Colombia blood agar. DNA extraction was performed after enzymatic lysis. The next step was a multiplexed linear amplification using one specific primer per target. During that non-exponential amplification, biotin-16-dUTP was incorporated into single-stranded amplicons. After incubation and washing, hybridisation was performed to probes immobilised on the array. Hybridisations were detected by adding streptavidin horseradish peroxidase that triggered a localised dye precipitation resulting in a formation of visible spots. Microarrays were then scanned and analysed using an Arraymate (Alere Technologies GmbH (Abbott), Jena, Germany) reading device. A second microarray (Alere, Monecke et al., 2016) was used to detect additional markers (**Supplemental File 1**), including the staphyloxanthin locus.

### Illumina Sequencing

Three isolates were subjected to WGS with the Illumina MiSeq platform (Illumina, Inc., San Diego, USA). The whole-cell DNA was extracted using the QIAamp^®^ DNA Mini Kit (QIAGEN, Hilden, Germany) with some adaptations for staphylococci as described previously (Scholtzek et al., 2019). The libraries for WGS were prepared using the Nextera XT DNA Library Preparation Kit (Illumina, Inc., San Diego, USA) according to the manufacturer’s recommendations. The 2×300 bp paired-end sequencing in 40-fold multiplexes was performed on the Illumina MiSeq platform (Illumina, Inc., San Diego, USA).

### Nanopore Sequencing

Oxford Nanopore Technology (ONT) sequencing of the study isolates, i.e., BDS-53E, BDS-54 and Zoo-28, was performed using two different MinION flow cells (IDs: FL1339 and FAO01531; rev: FLO-MIN106D containing an R9.4.1 pore). Library preparations were done using the 1D genomic DNA by ligation kit (SQK-LSK109, ONT), and the native barcoding expansion kit (EXP-NBD104, ONT) following manufacturer’s instructions with minor adaptations. In summary, an AMPure bead (Agencourt AMPure XP, Beckman Coulter, Krefeld, Germany) clean-up step was performed before the library preparation. Potential nicks in DNA and DNA ends were repaired in a combined step using NEBNext FFPE DNA Repair Mix and NEBNext Ultra II End repair/dA-tailing Module (NewEngland Biolabs, Ipswich, USA) by tripling the incubation time. A subsequent second AMPure bead purification was followed by the ligation of sequencing adapters onto prepared ends and a third clean-up step with AMPure beads. An additional barcoding and clean-up step was performed prior to adapter ligation. Sequencing buffer and loading beads were added to the library. At the start of sequencing, an initial quality check of the flow cells showed 1289 (FL1339) and 1388 (FAO01531) active pores. Genomic DNA samples used for loading comprised a total amount of around 25.5 ng per strain (measured by Qubit 4 Fluorometer; ThermoFisher Scientific, Waltham, USA). The sequencing ran for 48 hrs using the MinKNOW software version 20.06.5.

### Sequence Assembly and Polishing

For all nanopore data sets, the guppy basecaller (v4.2.2, Oxford Nanopore Technologies, Oxford, UK) translated and trimmed the MinION raw data (fast5) into quality tagged sequence reads (4,000 reads per fastq-file). Filtlong (v0.2.0) was used for bacterial DNA with a median read quality of 14 and a minimum read length of 1,000 bp to get a smaller and better subset of reads. The median read quality of 15.5 and a N50 read length of approximately 13,000 bp for each sample was highly suitable for assembly. Flye (v2.8.3) was used to assemble the reads to provide high quality contigs. Then, a racon-medaka (4-times racon v1.4.3; 1-time medaka v1.2.0) pipeline was applied for polishing. Moreover, pilon (v1.23) polished the sequences using Illumina sequence data (**Supplemental Files 3**/**4**/**5a**). The NCBI Prokaryotic Genome Annotation Pipeline (PGAP version 2021-01-11.build 5132) was used for annotating all assembled contigs in combination with an in-house database of published staphylococcal gene sequences (**Supplemental Files 3**/**4**/**5b**).

### Phylogenetic Analysis

**Figure 1:**
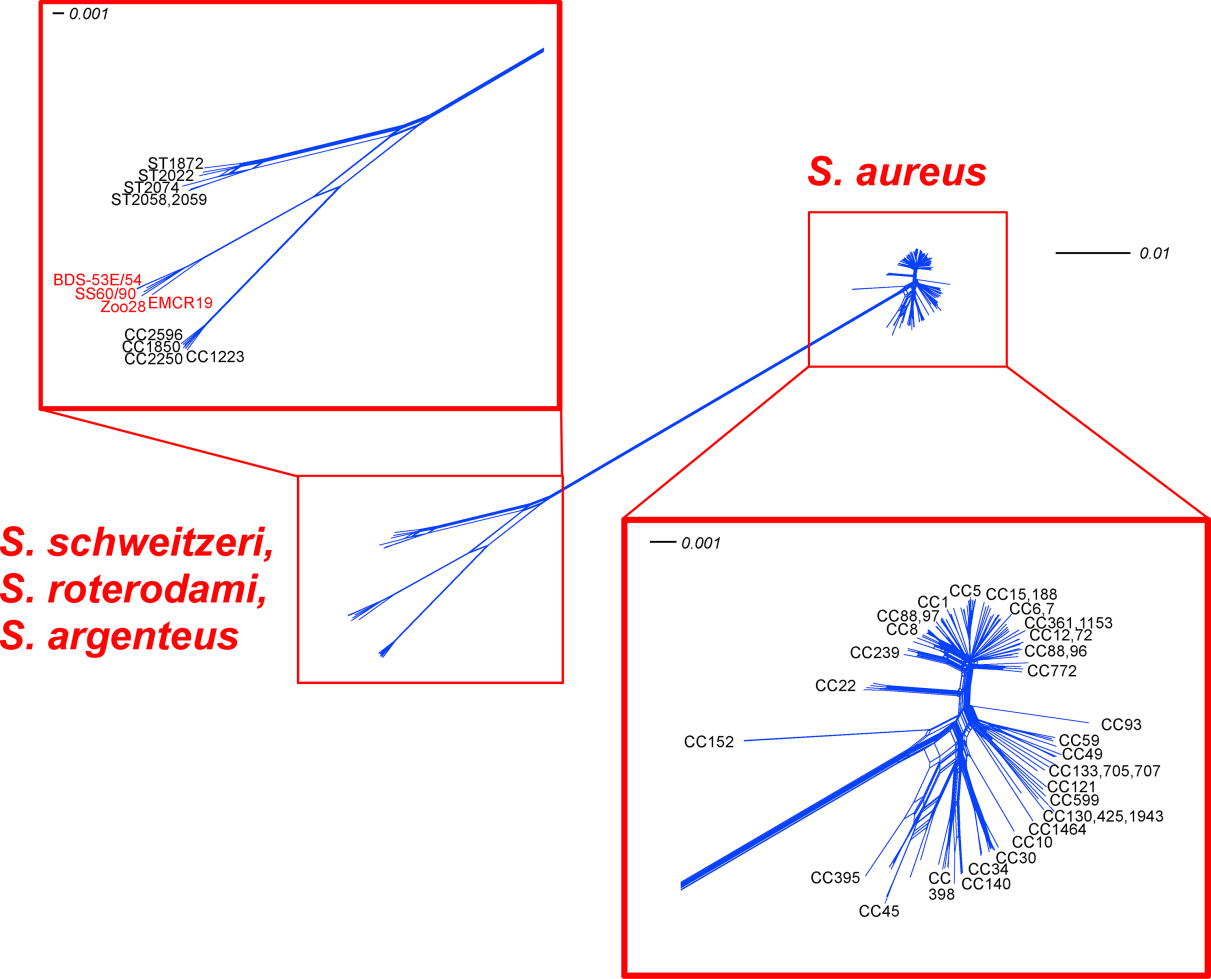
SplitsTree analysis for 154 core genomic markers of the study isolates, “*S. roterodami*” EMCR19 as well as “*S. singaporensis*” SS60 and SS90 compared to diverse *S. aureus, S. argenteus* and *S. schweitzeri* CCs. Note, for the genes that were found inverted in the Zoo-28 genome, reverse complement sequences were used for analysis.

We selected a panel of 154 core genome markers for tree construction using SplitsTree (Monecke et al., 2021). These genes and genome sequences used for visualisation (**Figure 1**) are listed in **Supplemental File 6**. Inclusion criteria were the presence of the genes in all CCs of *S. aureus/argenteus/schweitzeri* clonal complexes and uniform length in all published genomes. Sequences were concatenated and analysed using SplitsTree 4.0 (Huson and Bryant, 2006) using default settings (characters transformation, uncorrected P; distance transformation, Neighbour-Net; and variance, ordinary least squares).

### Analysis of the Core Genome

**Figure 2:**
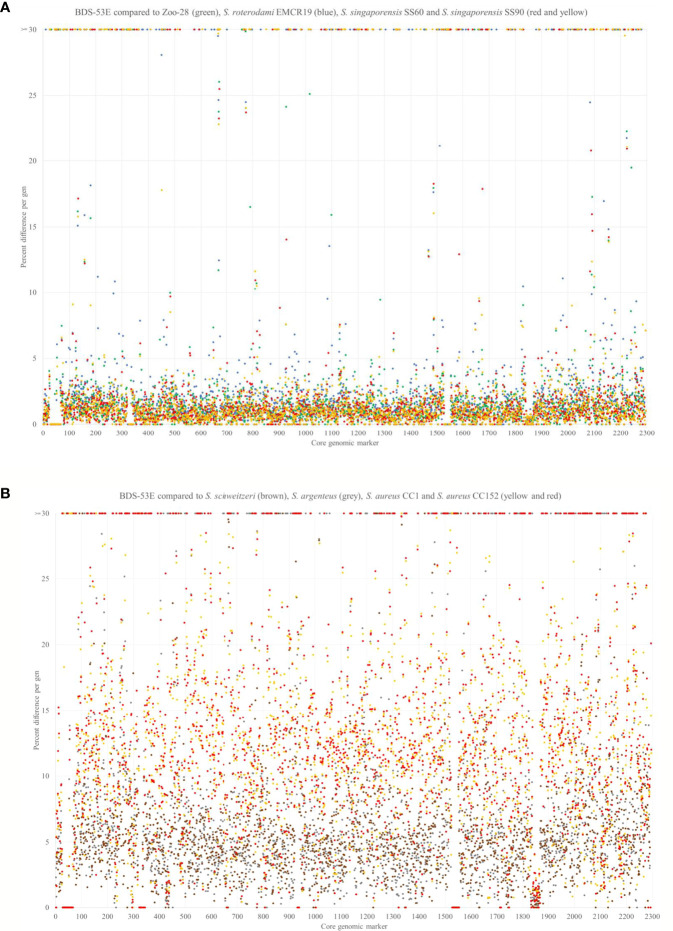
**(A)** SNP analysis comparing 2292 non-motile genes of BDS-53E with the Zoo-28 (green), “*S. roterodami”* EMCR19 (blue) as well as with *“S. singaporensis”* SS60 and SS90 (red and yellow). For each single gene, the number of differences (**Supplemental File 7B**) to the comparator strains was counted and expressed in percentage. For genes that were present in one, but absent in the other isolate, this percentage was set as 100%. Note, for the genes that were found inverted in the Zoo-28 genome, reverse complement sequences were used for analysis. **(B)** SNP analysis comparing 2292 non-motile genes of BDS-53E with *S. schweitzeri* (NCTC13712, LR134304.1, brown), *S. argenteus* (MSHR1132, FR821777.2, grey), *S. aureus* CC1 (MW2, BA000033.2, yellow) and *S. aureus* CC152 (SA17_S6, CP010941.1, red). The genes around pos. 1550 represent a genomic island that is absent in the study isolates (protease genes *splF, splE, splD2, splC, splB, splA*, leukocidin *lukD/E*, lantibiotic epidermin gene cluster). The highly conserved genes around pos. 1850 are those encoding ribosomal proteins.

A comprehensive analysis of existing sequence databases and available literature led to the definition of a set of 2,167 genes (**Supplemental File 7A**) in the core genome of *S. aureus*, *S. argenteus* and *S. schweitzeri.* These genes are almost universally present although in few cases sequences might be absent due to random mutation or sequencing/assembly artefacts. These genes always appeared in the same order within the genome, regardless of the identity of the isolate. In addition, 125 markers from major genomic islands were considered always present or usually present (with presence or absence being linked to species or CC affiliation). They also appear in the same positions within the genome. Genes in this category are the staphyloxanthin gene cluster, the *set/ssl* loci and the *egc* enterotoxin gene cluster. Genomic islands that might occupy variable locations within the genome were not considered. The sequences of these core genome and major genomic island genes were analysed and compared to each other and to reference sequences. A few genomic island genes not present in any of the isolates compared in the present study were excluded. Thus, a total of 2,292 genes were considered representing roughly 2,040,000 nucleotide positions per genome. For each gene, the number of nucleotides different from the comparator isolate was counted and expressed as a percentage of the length of the respective gene (**Supplemental File 7B**). Gaps in the alignment of different alleles of a given target gene were treated as mismatches. If a gene was present in one isolate but absent in the other isolate, this percentage was set as 100%. In addition, percentages were plotted over the positions in the genome (**Figure 2**). When comparing two isolates, median values for these percentages of all genes were calculated.

### Genome Taxonomy Database Toolkit (GTDB-tk)

We utilized GTDB-tk, a software toolkit for assigning objective taxonomic classifications to bacterial and archaeal genomes to determine if the genomes of the study isolates were novel species (Parks et al., 2018; Parks et al., 2020; Parks et al., 2022). To taxonomically assess the genomes, GTDB-Tk version 1.6.0 (Chaumeil et al., 2019) and the GTDB database version 202 (ref: https://gtdb.ecogenomic.org/stats/r202) containing 254,090 bacteria and 4,316 archaeal genomes) were utilised. Briefly, the target genomes were classified by the GTDB-Tk’s “classify” workflow (using the default settings) by placing them into the GTDB’s reference tree. We considered the average nucleotide identity (ANI), alignment fraction (AF) and its relative evolutionary divergence (RED) (Scholtzek et al., 2019) to the closely related reference genomes.

## Results

### Phenotypic Characterisation and Microarray-Based Genotyping

Based on the bioMérieux´ Gram-positive identification card (GP) for VITEK-2, the biochemical test results are summarised in **Table 2** (see also **Supplemental File 2**). Profiles were in accordance with *S. aureus*.

**Table 2:** Biochemical profiles, based on results for bioMérieux´ Gram-positive identification card (GP) for VITEK-2.

Pos.	Reaction	Code	BDS-53E	BDS-54	Zoo-28	SS21 (DSM111408)	EMCR19 (DSM111914)
2	D-Amygdalin	AMY	negative	negative	negative	negative	negative
4	Phosphatidylinositol phospholipase C	PIPLC	negative	negative	negative	negative	negative
5	D-Xylose	dXYL	negative	negative	negative	negative	negative
8	Arginine dihydrolase 1	ADH1	**positive**	**positive**	**positive**	**positive**	**positive**
9	b-Galactosidase	BGAL	negative	negative	negative	negative	negative
11	a-Glucosidase	AGLU	**positive**	**positive**	negative	**positive**	negative
13	Ala Phe Pro arylamidase	APPA	negative	negative	negative	negative	negative
14	Cyclodextrin	CDEX	negative	negative	negative	negative	negative
15	L-Aspartate arylamidase	AspA	negative	negative	negative	negative	negative
16	b-Galactopyranosidase	BGAR	negative	negative	negative	negative	negative
17	a-Mannosidase	AMAN	negative	negative	negative	negative	negative
19	Phosphatase	PHOS	**positive**	**positive**	**positive**	**positive**	**positive**
20	Leucine arylamidase	LeuA	negative	negative	negative	negative	negative
23	L-Proline arylamidase	ProA	negative	negative	negative	negative	negative
24	b-Glucaronidase	BGURr	negative	negative	negative	negative	negative
25	a-Galactosidase	AGAL	negative	negative	negative	negative	negative
26	L-Pyrrolidonyl-arylamidase	PyrA	**positive**	**positive**	negative	**positive**	**positive**
27	b-Glucaronidase	BGUR	negative	negative	negative	negative	negative
28	Alanine arylamidase	AlaA	negative	negative	negative	negative	negative
29	Tyrosine arylamidase	TyrA	negative	negative	negative	negative	negative
30	D-Sorbitol	dSOR	negative	negative	negative	negative	negative
31	Urease	URE	**positive**	**positive**	negative	negative	negative
32	Polymyxin B resistance	POLYB	**positive**	**positive**	**positive**	**positive**	**positive**
37	D-Galactose	dGAL	negative	negative	**positive**	**positive**	**positive**
38	D-Ribose	dRIB	**positive**	negative	**positive**	**positive**	negative
39	L-Lactate alkalinisation	ILATk	**positive**	**positive**	**positive**	**positive**	**positive**
42	Lactose	LAC	negative	negative	negative	negative	negative
44	N-Acetyl-D-glucosamine	NAG	negative	negative	negative	negative	negative
45	D-Maltose	dMAL	**positive**	**positive**	**positive**	**positive**	**positive**
46	Bacitracin resistance	BACI	**positive**	**positive**	**positive**	**positive**	**positive**
47	Novobiocin resistance	NOVO	**positive**	negative	negative	negative	negative
50	Growth in 6.5% NaCl	NC6.5	**positive**	**positive**	**positive**	**positive**	**positive**
52	D-Mannitol	dMAN	**positive**	**positive**	**positive**	**positive**	**positive**
53	D-Mannose	dMNE	**positive**	**positive**	**positive**	negative	**positive**
54	Methyl-B-D-glucopyranoside	MBdG	**positive**	**positive**	**positive**	**positive**	**positive**
56	Pullulan	PUL	negative	negative	negative	negative	negative
57	D-Raffinose	dRAF	negative	negative	negative	negative	negative
58	O129 Resistance	O129R	**positive**	**positive**	**positive**	**positive**	**positive**
59	Salicin	SAL	negative	negative	negative	negative	negative
60	Saccharose/sucrose	SAC	**positive**	**positive**	**positive**	**positive**	**positive**
62	D-Trehalose	dTRE	negative	negative	**positive**	**positive**	**positive**
63	Arginine dihydrolase 2	ADH2s	negative	**positive**	negative	negative	negative
64	Optochin resistance	OPTO	**positive**	**positive**	**positive**	**positive**	**positive**

Based on their irregular microarray hybridisation patterns (see **Supplemental File 1**), an assignment of the isolates to any known lineage of *S. aureus* was not possible, suggesting affiliation to either *S. argenteus* or *S. schweitzeri*. However, isolates yielded positive signals for one *crtM* probe and weak signals for *crtP*. Since these probes recognise genes from the staphyloxanthin cluster, which by definition should be absent from *S. argenteus*, the isolates could not be assigned to this species. On the other hand, all three isolates carried *ycjY*, a marker on a genomic island identified in *S. argenteus* and some *S. aureus* lineages (CC12, CC361 and CC398), but absent from any *S. schweitzeri* tested or sequenced. The *orfX-*associated (Holt et al., 2011) *cas1* CRISPR-endonuclease 1 (FR821777.2; pos. 62,418...63,323, which can be observed in *S. argenteus* CC1850 and CC2250) was not detected. The isolates clearly clustered into two distinct putative CCs.

The Nigerian bat isolates were highly similar to each other. They yielded signals with *hld*, *agrC/D*-I and with *S. argenteus*-specific *agr* probes, indicating a presence of an *agr* gene cluster albeit an atypical allele. Similarly, *icaA* was the only *ica* gene detected by the array (while the others were detected by sequencing; see **Supplemental Files 3**, **4**). Capsule genes were not detected by the array although sequencing showed a presence of specific alleles. The *cna* gene was absent, while *sasG* was present. The *ssl* genes (encoding staphylococcal superantigen-like protein locus 1) were not detected by the array although one gene of this cluster was found by sequencing.

The bat isolates could further be subdivided based on reactivities with either *sdrC* or *sdrD* probes. For sequencing, one *sdrC*- and one *sdrD-*positive isolate (BDS-53E and BDS-54, respectively) were selected.

The German isolate Zoo-28 shared *agr* and *ica* reactivities. It differed in the absence of *sasG* and in the allelic variants of several adhesion factors. In contrast to the bat isolates, several *ssl* genes were present (see **Supplemental Files 1**, **5**). It also harboured the leukocidin genes *lukD/E*, which were absent from the bat isolates (although the latter component was identified only by sequencing).

### MLST and Phylogenetic Analysis

MLST yielded profiles that are shown in the first half of **Table 1**. Previously published MLST profiles that appeared related are listed in the second half of the table.

An MLST-like approach based on 154 core genomic markers (**Figure 1**; **Supplemental File 6**) led to the clustering of all *S. aureus*, with three major groups and three separate branches. One group comprised CC1, CC5, CC8 and most of the other *S. aureus* lineages. A second one included CC59, CC121 and several, mostly animal-associated, lineages such as CC49, CC130 and CC1464 (“*S. aureus* subsp. *anaerobius*”). The third group consisted mainly of CC30, CC45, CC398. The separate branches, CC22, and more conspicuously, CC93 and CC152, appeared to be more distant from other *S. aureus* lineages.

Another very distant branch consisted of *S. argenteus* lineages (CC1223, CC1850, CC2198, CC2250, CC2596 and CC4587). *S. schweitzeri* (ST1872, ST2022, ST2058, ST2059, ST(206-303-253-142-196-202-197); accession number CCEO01000001-CCEO01000054) was also clearly separate, but much closer to *S. argenteus* than *S. aureus*. The genomes of BDS-53E, BDS-54 and Zoo-28 were closely related to “*S. roterodami*/*singaporensis”* (EMCR19, SS60, SS90). The SplitsTree in **Figure 1** shows that the aforementioned “*S. roterodami*/*singaporensis”* isolates as well as the three sequenced study isolates were located between *S. argenteus* and *S. schweitzeri*.

### Sequence Analysis of the Core Genome and Major Genomic Islands

For the Nigerian bat isolates, the order of the predefined core genomic markers and major genomic island markers within the genomes was identical as observed with the published sequences of *S. aureus, S. argenteus* and *S. schweitzeri*. In the isolate Zoo-28, a large part of the genome was found inverted (already in the Nanopore sequence before polishing with Illumina) and integrated further downstream, into the *map/eap* gene, with *map/eap* fragments identified at pos. 1,991,191...1,992,912 and 2,090,178...2,091,407 of the genome. This inverted part of the chromosome contained approximately 69,000 bp or 60 genes from *namA* (NADH:flavin oxidoreductase) to *yrbD* (alanine:cation symporter family protein), including the chromosomal oligopeptide ABC transporter cluster *oppA/oppF/oppD/oppC/oppB*. It was flanked at both sides by transposase genes and genes of yet unknown function resulting in a total size of the insert of about 97 kb. Another transposase gene was identified at the original position where the inserted genes were supposed to be localised.

**Figure 3:**
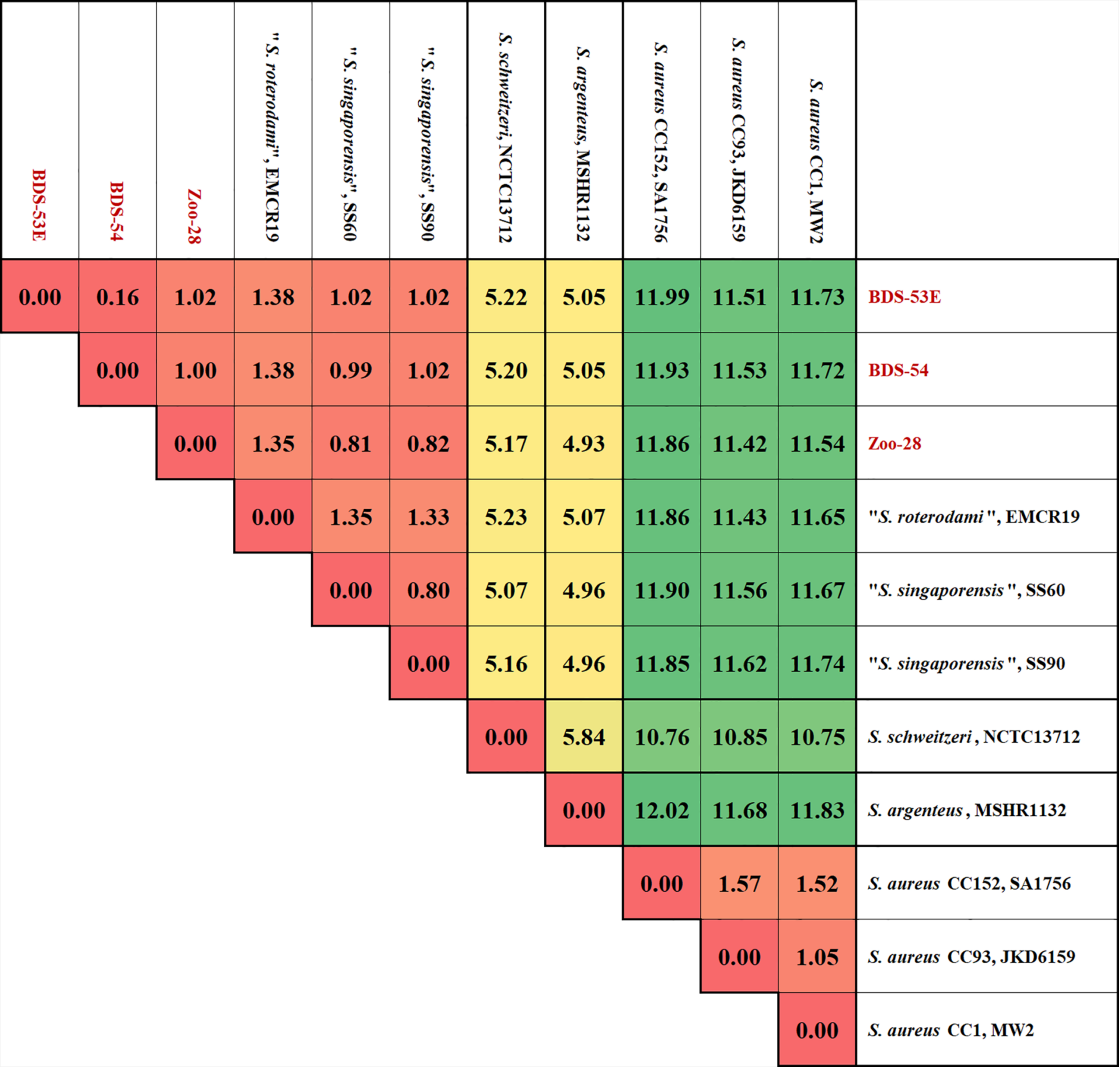
Matrix of differences between BDS-53E, Zoo-28, “*S. roterodami*” (EMCR19, CAJGUT01), “*S. singaporensis”* (SS60 and SS90, NZ_JABWHF and NZ_JABWHD), *S. schweitzeri*, (NCTC13712, LR134304.1), *S. argenteus* (MSHR1132, FR821777.2) and three *S. aureus* lineages, CC1 (MW2, BA000033.2), CC93 (JKD6159, CP002114.2) and CC152 (SA17_S6, CP010941.1). The percentages were calculated as explained for **Figure 2** and the image shows the median values over all these 2292 genes for each genome sequence compered to all others.

The analysis and comparison of the core genomic and major genomic island genes of the three isolates and reference sequences indicated that the differences of BDS-53E compared to the reference sequences are somewhat uniformly scattered all across the genome. Moreover, BDS-53E and Zoo-28 are similar to *S. roterodami* and *S. singaporensis* (**Figure 2**). Using the median values for the differences of all genes to compare sequences of the study isolates, we observed that these sequences and those of *S. roterodami* and *S. singaporensis* differed by approximately 1% (**Figures 2A**, **3**). Furthermore, we observed approximately 5% differences compared to both, *S. argenteus* and *S. schweitzeri*, and of about 11-12% compared to different *S. aureus* CCs (**Figures 2B**, **3**).

**Figure 4:**
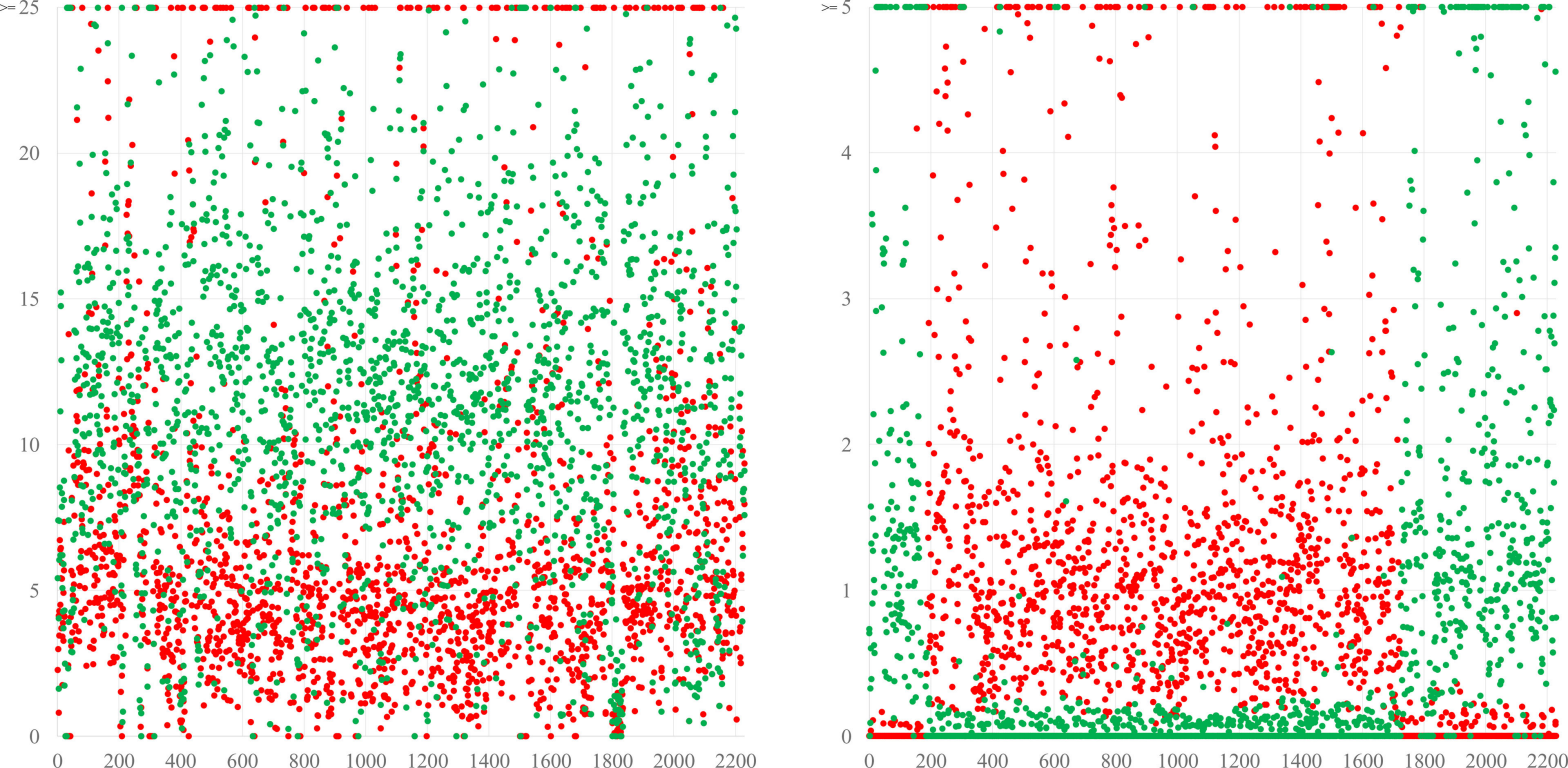
The left diagram shows a simplified version of the blot from **Figure 2**, comparing BDS-53E to the reference sequences of *S. schweitzeri* NCTC13712 (red) and the *S. aureus* CC1 strain MW2 (green). Differences are evenly distributed all across the genome, suggesting a continuous evolution and an accumulation of more mutations compared to *S. aureus* than to *S. schweitzeri* corresponding supposedly to a longer time of separate evolution. The right diagram shows the same analysis for a chimeric strain in which a fragment of “red” origin spanning from approximately position 1750 over *oriC* (pos. 0) to pos. 200 is integrated into a backbone genome of “green” provenance. These are actually *S. aureus* CC140 and CC8, in a ST6610 strain, see (Burgold-Voigt et al., 2021). For the Y-axes, different scales were used because differences between the different species are larger than those between different CCs of *S. aureus*.

### Analysis by GTDB-tk

The genome sequences of the study isolates were taxonomically placed in the genus of *Staphylococcus* without a species assignment as their closest relative, *S. argenteus* (GCF_000236925.1), shared only an average nucleotide identity of 93.86% to 94.01% (**Table 3**). The second most closely related species was *S. schweitzeri* (average nucleotide identity, ANI, 93.53 to 93.63), followed by *S. aureus* (ANI, 88.98 to 89.22). *S. singaporensis* and *S. roterodami* are not (yet) covered by the database.

**Table 3:** GTDB-tk results.

Genome	Closest relative	Average nucleotide identity to closest relative	Alignment fraction to closest relative
**BDS-53E**	*S. argenteus* (GCF_000236925.1)	93.86	0.91
**BDS-54**	*S. argenteus* (GCF_000236925.1)	94.01	0.92
**Zoo28**	*S. argenteus* (GCF_000236925.1)	93.99	0.91

### Resistance Genes and Antimicrobial Susceptibility Testing

All study isolates were methicillin susceptible and negative by both, array and sequencing, for *mecA/mecC* genes.

Antimicrobial susceptibility testing using the AST-P608 panel showed that BDS-53E was fully susceptible to all compounds tested. BDS-54 was susceptible to all compounds but intermediate to fluoroquinolones. However, in *grlA*, some single nucleotide polymorphisms (SNPs) were either observed in BDS-53E and BDS-54 (E310K, K650N, H767Q) or in the three study isolates and in *S. argenteus* and *S. schweitzeri*. Similarly, most SNPs in the *gyrA* gene separated the three study isolates plus *S. argenteus* and *S. schweitzeri* from *S. aureus* (e.g., E248V, V623I). Previously described mutations (Wang et al., 1998) were not identified.

A distinct result for BDS-53E (from the Gram-positive ID panel) was its novobiocin resistance. As mutations in *gyrB* (CP000253.1; 5,034...6,968) and *parE=grlB* (CP000253.1; 1,292,206...1,294,197) might be related to novobiocin resistance (Fujimoto-Nakamura et al., 2005), the sequences of these genes were analysed. The BDS-53E sequence of *gyrB* showed a unique mutation resulting in a substitution of aspartic acid by valine at amino acid position 494, which was not present neither in the other two study isolates nor in some reference sequences (MSHR1132, MW2, SA17_S6, JKD6159 and NCTC13712). The BDS-53E sequence of *parE=grlB* did not contain unique SNPs although in some positions, SNPs were observed that either all three study isolates shared (I490L), or had in common with *S. argenteus* and *S. schweitzeri*.

BDS-53E and BDS-54 carried *aadK*, a gene encoding an aminoglycoside 6-adenyltransferase gene not covered by the microarrays used. It was nearly identical (in 826 of 846 nt) to CP010526.1, 421,013...421,858. In both isolates, it was localised on putative pathogenicity islands integrated between the gene *guaA* (glutamine-hydrolysing GMP synthase) and Q8NY63 (putative protein), around position 400,000 in the genome. These pathogenicity islands additionally included several genes for “hypothetical proteins”, a DNA primase, an integrase and a small terminase subunit.

The isolate Zoo-28 was resistant to benzylpenicillin and tetracycline. A *blaZ/I/R* operon was identified together with *yolD* and *tnpA/B/C* genes as part of a transposon that showed 99.58% identity to Tn*553*, a non-conjugative transposon of the Tn*554* family. This transposon has recently been described in porcine *S. aureus* ST9 from China (Krüger et al., 2021). Like the original Tn*553*, the Tn*553* in Zoo-28 was also integrated into the chromosomal Q5HEJ7/*yolD-*like gene. Tetracycline resistance was attributable to the tetracycline resistance gene *tet*(M). It was accompanied by a Tn*916*-transposase, a Tn*916*-excisionase, a gene encoding D2N5T7 (a conjugative transposon protein), *yddH* (encoding a putative peptidase cell wall hydrolase), *ardA2* (coding for an antirestriction protein) and a couple of hypothetical proteins. The entire transposon was identical to Tn*916* (GenBank, U09422.1), a transposon first described in enterococci but also known from staphylococci such as CC398-MRSA-VT (S0385, GenBank AM990992.1 and 08BA02176, GenBank CP003808.1).

### Other Mobile Genetic Elements

All three sequenced study isolates did not harbour SCC*mec* elements. BDS-53E and BDS-54 carried clusters of twelve (BDS-54; see **Supplemental File 4B**) or twenty genes (BDS-53E; see **Supplemental File 3B**) associated with, and located directly downstream of *orfX.* These were automatically annotated as “hypothetical proteins”, transposases, helicases, methyltransferases, oxidoreductases and hydrolases. In addition, two of the un-sequenced bat isolates (BDS-69C, BDH-147) were positive in array experiments for B2Y834 (a marker usually associated with SCC*mec* IV A, IV E and IV c) as well as another one (BDH-128) with *ccrA-1*.

Zoo-28 lacked these genes, carrying transposase and helicase genes instead, as well as one gene, B6VQU0, which is otherwise known to occur in CC705 (GenBank AJ938182, pos. 34,735...35,634, downstream of *orfX*), as well as in SCC*mec* IV h/j elements.

Downstream of the SCC integration site, a large genomic island is situated whose gene content is related to CC affiliation. Typical genes of that genomic island, such as *seh* and ORF CM14 (characteristic for, *e.g.*, CC1, CC10, CC34, or CC93 or CC772), were absent from the study isolates. BDS-53E and BDS54 harboured in this position *mcrB* (type IV 5-methylcytosine-specific restriction enzyme subunit B), *mcrC* (subunit C) and Q6GD44 (putative acetyltransferase, GNAT family) genes. Zoo-28 differed, carrying C1PH96 (putative protein, carboxymuconolactone decarboxylase family), *lrpC* (HTH-type transcriptional regulator *Lrp/AsnC* family) and Q6GD44 genes.

BDS-53E and BDS-54 carried prophages that could be assigned to *Siphoviridae* based on sequence similarity to known *S. aureus* phages. In both cases, phages were integrated between the genes encoding a putative protein A5ITW8 and tRNA for serine, approximately at position 1,800,000 in the genomes. Phages were similar but not identical (see **Supplemental Files 3B**, **4B**). Both isolates harboured putative pathogenicity islands as described above.

In Zoo-28, no prophage was identified, but it carried as much as three different pathogenicity islands with genes for “hypothetical proteins”, integrases as well as for small terminase subunits. Finally, Zoo-28 carried Tn*916* and Tn*553*-like transposon as discussed above.

There was no evidence for plasmids in the three study isolates.

## Discussion

Evolution is a continuous process that does not occur in discrete steps. Thus, the classification of “evolving live forms” into discrete or distinct species is always problematic regardless of whether they are bacteria or higher organisms, such as herring gulls (Liebers et al., 2004), or cervids (Ludt et al., 2004). This distinction cannot be made without a certain element of arbitrariness. This is caused by an emphasis on specific features of the target organism that are considered sufficient to define a species and by the fact that different observers might prioritise different properties. For instance, traditionally, *Shigella* is a distinct taxonomic entity from *Escherichia* (*E.*) *coli* because of the “severity of dysentery” and its lethality in humans. However, an investigation of *Shigella* gene sequences shows only minimal differences compared to *E. coli* which do not justify its recognition as distinct genus or species (van den Beld and Reubsaet, 2012). For other life forms, taxonomists argue about the fertility of hybrids, or, in the case of the herring gulls mentioned above, mating calls and feet colour (Collinson et al., 2008). A numerical approach to gene analysis might reduce arbitrariness although different “threshold values” must carefully be considered for various clades of life forms.

The numerical approach for assessing nucleotide differences per gene length yielded some interesting results in the present study. First, within *S. aureus*, the median difference for all 2,292 genes considered was only 1 to 1.5%, even when comparing a CC1 reference sequence to such diverse, or deviant, lineages as CC93 or CC152. This observation gives an impression or benchmark for the largest possible difference *within* one established species. Median differences between *S. aureus* and *S. argenteu*s or *S. schweitzeri* were 10-12%, while it was about 5% between *S. argenteus* and *S. schweitzeri*. These data might provide an orientation on the level of distinction of valid species to one another. The study isolates differed from each other by 0.16% to 1.38% but differed from *S. argenteus* and *S. schweitzeri* by about 5% and from diverse *S. aureus* lineages (CC1, CC93, and CC152) by 11-12%. These observations suggest that the study isolates belonged to one distinct species, with the African isolates in one CC and the German zoo isolate in another one. However, a comparison of the genomes of the study isolates to the recently published genomes of the isolates assigned to the new species *S. roterodami* and *S. singaporensis* (Schutte et al., 2021; Chew et al., 2021) yielded median differences of around 1%, and the difference between these two species was about the same.

Our observations provide two options. First, one might conclude that a difference of more than 10% was required for recognition as a full species. Hence, *S. aureus* would be one species, while *S. schweitzeri, S. argenteus, S. roterodami, S. singaporensis* and the study isolates clustered to another one. Second, one might define a median difference of about 5% as a threshold for a species. This is implemented in GTDB-tk where a query genome is regarded as the same species as the closest reference sequence if it falls within an ANI of at least 95% and an AF of 65%. In this case, *S. aureus*, *S. schweitzeri* and *S. argenteus* would be three species, while a forth one comprised both *S. roterodami* and *S. singaporensis*, as well as the study isolates. However, phenotypical tests allow no clear distinction of these isolates from the other members of this “species” raising the question of whether sequence analysis should have priority over biochemical tests or not. Hence, a discussion about the definition of clear criteria for recognition as a discrete species is necessary especially as new technologies and lower costs facilitate sequencing of isolates that could not be sequenced before, including those from faeces of exotic animals, resulting in an unprecedented increase in the number of available genome sequences.

We present evidence for recognising a new species of coagulase- and staphyloxanthin-positive staphylococci positioned between *S. argenteus* and *S. schweitzeri*. The previously described species “*S. roterodami*” and “*S. singaporensis*” as well as isolates described herein all together should be regarded as a single species, *i.e.*, as the fourth one in the *S. aureus* complex in addition to *S. aureus, S. argenteus* and *S. schweitzeri*. According to the principle of priority, that species should be named *S. roterodami* as this name was published first, in September 2021 (Schutte et al., 2021).

This species has a much wider geographical range than previously thought, *i.e*, Nigeria, Southern China, Indonesia, Singapore and possibly Australia.

The majority of isolates described herein and two previously published MLST profiles originate from bat faeces from Nigeria. These two MLST profiles were posted to the MLST database by a Japanese and a British group (ST2470 and ST3135). Sequences are not identical albeit similar, and the sampling location was approximately the same as for the bat isolates described herein. One isolate (Zoo-28) was sampled from an estrildid finch living in a zoo in Germany. While this species is native and endemic to Australia, this individual zoo animal might have been colonised/infected by contact with other animals such as flying foxes kept in close proximity. Unfortunately, we cannot investigate that issue anymore due to alterations to the building and the aviary kept inside. “*S. roterodami*” and “*S. singaporensis*” were isolated from humans returning from Bali or living in Singapore, respectively, as discussed above. Further related STs, ST4075, ST4076, ST4569, were observed in isolates from unspecified food from the Guangzhou region, in the southern part of China. Another food isolate, ST4185 originated from Yunnan, a province in Southwestern China. These four STs share a unique *gmk* allele (*gmk*-315) suggesting affiliation to yet another CC. They also present ordinary *S. aureus*-like *aroE*-alleles and unique, deviant *arcC*-alleles.

*S. roterodami* is a polymorphic species, consisting of at least nine distinct CCs with the animal isolates described herein constituting two CCs. The Bali isolate originally described as *S. roterodami* represents another one. The six “*S. singaporensis*” isolates could be classified into five different CCs. Differences between the CCs of *S. roterodami* include carriage, or absence, of gene clusters that also define complexes within *S. aureus* or *S. argenteus*. These include *agr* locus genes, the *set/ssl* cluster, the enterotoxin gene cluster *egc, edinB+etD* and *sasG*. The four sequence types from Southern China might represent at least one additional CC.

In addition to the CCs discussed above, there are several STs in the MLST database that appear to be *S. aureus* although they contain one *S. roterodami* MLST allele each in addition to six regular *S. aureus* MLST markers. Whether this was evidence for cross-species hybridisation or chimerism involving *S. aureus* and *S. roterodami*, an accidentally identical accumulation of mutations, or merely technical issues, still needs to be clarified. These STs include ST4051 with *gmk*-190 while the other markers are in accordance with a CC1 profile, ST3089 that differs from CC130 in *arcC-*0349 and ST4466 that carries *gmk-*0315 although it otherwise resembles CC7. Finally, a MRSA lineage associated with imported macaques (*Macaca sp*.), ST3268/ST2817, was identified in the USA, China, and Singapore (Soge et al., 2016; Hsu et al., 2017; Roberts et al., 2018; Li et al., 2020) in which a *S. roterodami*-like *gmk* allele (*gmk*-214) is present, among other MLST alleles that could be derived from *S. aureus* CC45.

With regard to chimerism, one might argue that the *S. roterodami* complex was a group of chimeric *S. argenteus* isolates that acquired the gene cluster encoding the “golden” carotenoid pigment staphyloxanthin by chromosomal replacement, hybridisation, or chimerism. **Figure 1** contains information rendering that concept rather unlikely. The differences compared to the reference isolates affect all parts of the genome essentially and are distributed evenly across the genome (with the notable exception of a few highly conserved genes encoding ribosomal proteins). Previous work (Nimmo et al., 2015; Burgold-Voigt et al., 2021) showed how a part of the genome of a chimeric isolate genome would match the corresponding part from one parent strain, and differ from the same region of the other one, while this would be conversely for the rest of the genome (**Figure 4**). Therefore, we can assume that the similarities and differences of the *S. roterodami* complex compared to *S. aureus*, *S. argenteus* and *S. schweitzeri* do not result from a large-scale chromosomal replacement or chimerism. Thus, the isolates cannot be considered *S. argenteus* that by chance acquired the staphyloxanthin locus from elsewhere. The more likely explanation was a continuous evolution and accumulation of mutations over time. Based on the median differences calculated for core genomic markers (**Figure 3**), we postulate that the split from *S. aureus* occurred earlier than the one from *S. argenteus* and *S. schweitzeri*.

Identifying *S. aureus, S. argenteus, S. schweitzeri* and *S. roterodami* from humans and/or wild animals in Africa suggest that they could have originated from the continent, disseminating to other parts of the world through human migration. Thus, it would be interesting to screen African wildlife for other possible branches of the staphylococcal phylogenetic tree to understand the co-evolution of humans and animals with their coagulase-positive colonisers/pathogens.

*S. roterodami* has been found in symptomatically ill humans as well as in wild bats and a captive finch, suggesting a relatively broad zoonotic host spectrum as well as a certain virulence in humans. Besides, it can acquire resistance genes known from other staphylococci such as *blaZ*, *tet*(M), *aadK*, *aacA-aphD* and *aadD* as demonstrated in the study isolates and in those identified in Singapore (Chew et al., 2021). Thus, its clinical relevance could be comparable to that of *S. aureus* or *S. argenteus*.

Further studies should focus on staphylococcal isolates from humans, bats, rodents, birds, and atypical *S. aureus* isolates from Western and Central Africa, Southern and Southeastern Asia as well as Australia. While phenotypic tests might not be conclusive, unique MLST alleles (see **Table 1**) should help identifying *S. roterodami* isolates. However, a non-molecular algorithm for diagnostic procedures to identify the new species from routine diagnostic samples is needed.

## Data Availability Statement

The genome sequences of the study isolates were submitted to GenBank. The BioProject accession number is PRJNA810320, BioSamples are SAMN26244312 to 314 and the GenBank accession numbers are CP092781, CP092782 and CP092783. All other data are provided within the manuscript, or as Supplemental Files.

## Ethics Statement

No animal experiments were performed and no animal was sacrificed for this study. No ethical clearance was necessary as no animals were captured and no invasive samples were taken. The strains originated from environmental samples (i.e., bat faeces collected under trees used by wild bats for roosting) and from a *post mortem* sample from a zoo animal submitted for routine diagnostic procedures.

## Author Contributions

SM designed the study. SM, FS, AS, and ATF wrote the manuscript. FS, AS, ATF, and KM obtained samples and performed experiments (bacteriological work). HH, CD, MR, and SB performed experiments (sequencing). SM, CB, MC, and DH analysed sequence data. EM and DG performed experiments (bacteriological work and arrays). ATF, SS, and RE supervised the work and revised the manuscript. All authors contributed to the article and approved the submitted version.

## Funding

The Jena group acknowledges support by the German Federal Ministry of Education and Research, within the framework of the ADA project (13GW0456C) aiming to develop rapid tests for the detection and characterization of resistance genes and virulence factors in zoonotic *S. aureus*/MRSA. The FU Berlin group acknowledges funding by the German Federal Ministry of Education and Research under project number 01KI2009D a as part of the Research Network Zoonotic Infectious Diseases. AS was an awardee of the Georg Forster Research Fellowship (for Experienced Researchers) of the Alexander von Humboldt Foundation.

## Conflict of Interest

DG is employed by a company, Illumina, but he performed experiments for this study before commencing this employment.

The remaining authors declare that the research was conducted in the absence of any commercial or financial relationships that could be construed as a potential conflict of interest.

## Publisher’s Note

All claims expressed in this article are solely those of the authors and do not necessarily represent those of their affiliated organizations, or those of the publisher, the editors and the reviewers. Any product that may be evaluated in this article, or claim that may be made by its manufacturer, is not guaranteed or endorsed by the publisher.

## Supplementary Material

Supplemental File 1 Hybridisation profiles of study isolates and reference strains (pdf).

Supplemental File 2 Vitek Data (pdf).

Supplemental File 3
**(A)** Genome sequence of BDS-53E (fasta). **(B)** Genes identified within the genome sequence of BDS-53E (fasta).

Supplemental File 4
**(A)** Genome sequence of BDS-54 (fasta). **(B)** Genes identified within the genome sequence of BDS-54 (fasta).

Supplemental File 5
**(A)** Genome sequence of Zoo-28 (fasta). **(B)** Genes identified within the genome sequence of Zoo-28 (fasta).

Supplemental File 6 Strains and concatenated Sequences used for the Splitstree analysis.

Supplemental File 7
**(A)** Sequences for analyses of strain similarities as shown in and (fasta). **(B)** Percentages difference as used for **Figures 2A, B**.
