# Supplementary material for: Description of Staphylococcal Strains from Straw-Coloured Fruit Bat (Eidolon helvum) and Diamond Firetail (Stagonopleura guttata) and a Review of their Phylogenetic Relationships to Other Staphylococci
Source: Front Cell Infect Microbiol. 2022 May 11;12:878137. doi: 10.3389/fcimb.2022.878137 (PMC9132046; doi:10.3389/fcimb.2022.878137)
Supplement: Supplemental File 1 — Hybridisation profiles of study isolates and reference strains (pdf). [file DataSheet_1.zip › Supplemental file 1_Full Hybridisation profiles.pdf]

STRAIN / ISOLATE	GenBank	SPECIES MARKER							STAPHYLOXANTHIN BIOSYNTHESIS OPERON					REGULATORY GENES		
									crtM							
		rrnD1	gapA	katA	CoA	nuc1	spa	sbi	crtM-nonST93	crtM-ST93	crtN	crtO	crtP	sarA	saeS	vraS
		Domain 1 of 23S-rRNA	glyceraldehyde 3-phosphate dehydrogenase, locus 1	katalase A	coagulase	thermostable extracellular nuclease	staphylococcal protein A	IgG-binding protein	dehydrosqualene synthase		dehydrosqualene desaturase	staphyloxanthin acyltransferase	diaponeurosporene oxidase	staphylococcal accessory regulator A	histidine protein kinase, sae locus	sensor protein
**CC3960/3961**																
BDS-53E: in silico predicted hybridisation pattern	CP092783.1	POS	POS	NEG	POS	NEG	POS	NEG	NEG	POS	NEG	NEG	AMB	POS	POS	POS
Bat isolate BDS-53E		POS	POS	NEG	NEG	NEG	POS	NEG	NEG	POS	NEG	NEG	NEG	POS	POS	POS
Bat isolate BDS-53B		POS	POS	NEG	NEG	NEG	POS	NEG	NEG	POS	NEG	NEG	NEG	POS	POS	POS
BDS-54: in silico predicted hybridisation pattern	CP092782.1	POS	POS	NEG	POS	NEG	POS	NEG	NEG	POS	NEG	NEG	AMB	POS	POS	POS
Bat isolate BDS-54		POS	POS	NEG	NEG	NEG	POS	NEG	NEG	POS	NEG	NEG	NEG	POS	POS	POS
Bat isolate BDH-128		POS	POS	NEG	NEG	NEG	POS	NEG						POS	POS	POS
Bat isolate BDH-157		POS	POS	NEG	NEG	NEG	POS	NEG	NEG	POS	NEG	NEG	NEG	POS	POS	POS
Bat isolate BDS-69C		POS	POS	NEG	NEG	NEG	POS	NEG	NEG	POS	NEG	NEG	NEG	POS	POS	POS
Bat isolate BDH-147		POS	POS	NEG	NEG	NEG	POS	NEG	NEG	POS	NEG	NEG	NEG	POS	POS	POS
**CC7342**																
Zoo-28: in silico predicted hybridisation pattern	CP092781.1	POS	POS	NEG	POS	NEG	POS	NEG	NEG	POS	NEG	NEG	AMB	POS	POS	POS
Finch isolate Zoo-28		POS	POS	NEG	NEG	NEG	POS	NEG	NEG	POS	POS	NEG	AMB	POS	POS	POS
**"S. singaporensis", CC6105**																
SS21: in silico predicted hybridisation pattern	JABWHB	POS	POS	NEG	POS	NEG	POS	NEG	NEG	POS	NEG	NEG	AMB	NEG	POS	POS
DSM11148_Staphylococcus sp. SS21		POS	POS	NEG	NEG	NEG	POS	NEG	NEG	POS	NEG	NEG	NEG	POS	POS	POS
**"S. singaporensis", CC6106**																
SS35: in silico predicted hybridisation pattern	NZ_JABWPO	POS	POS	NEG	POS	NEG	POS	NEG	NEG	POS	NEG	NEG	AMB	NEG	POS	POS
**"S. singaporensis", CC6107**																
SS60: in silico predicted hybridisation pattern	NZ_JABWHF	POS	POS	NEG	POS	NEG	POS	NEG	NEG	POS	NEG	NEG	AMB	POS	POS	POS
**"S. singaporensis", CC6108**																
SS87: in silico predicted hybridisation pattern	NZ_JABWHE	POS	POS	NEG	POS	NEG	POS	NEG	NEG	POS	NEG	NEG	AMB	POS	POS	POS
**"S. singaporensis", CC6109**																
SS90: in silico predicted hybridisation pattern	NZ_JABWHD	POS	POS	NEG	NEG	NEG	POS	NEG	NEG	POS	NEG	NEG	AMB	POS	POS	POS
SS251: in silico predicted hybridisation pattern	NZ_JABWHC	POS	POS	NEG	NEG	NEG	POS	NEG	NEG	POS	NEG	NEG	AMB	POS	POS	POS
**"S. roterodami", CC6999**																
EMCR19: in silico predicted hybridisation pattern	CAJGUT	POS	POS	NEG	POS	NEG	POS	NEG	NEG	POS	NEG	NEG	POS	POS	POS	POS
DSM111914_Staphylococcus sp. EMCR19		POS	POS	NEG	NEG	NEG	POS	NEG	NEG	POS	NEG	NEG	NEG	POS	POS	POS
**S. argenteus, CC1850**																
MSHR1132: in silico predicted hybridisation pattern	FR821777.2	POS	POS	AMB	NEG	AMB	POS	POS	NEG	NEG	NEG	NEG	NEG	POS	POS	POS
DSM28299		POS	POS	POS	NEG	POS	POS	AMB	NEG	NEG	NEG	NEG	NEG	POS	POS	POS
**S. schweitzeri, CC2022**																
NCTC13712: in silico predicted hybridisation pattern	LR134304.1	POS	POS	AMB	NEG	NEG	POS	NEG	NEG	NEG	POS	NEG	AMB	POS	POS	POS
DSM28300		POS	POS	POS	NEG	NEG	NEG	NEG						POS	POS	POS
**S. saureus, CC1**																
MW2: in silico predicted hybridisation pattern	BA000033.2	POS	POS	POS	POS	POS	POS	POS	POS	NEG	POS	POS	POS	AMB	POS	POS
MW2		POS	POS	POS	POS	POS	POS	POS	POS	NEG	POS	POS	POS	POS	POS	POS

STRAIN / ISOLATE	REGULATORY GENES																	
	agrI				agrII				agrIII				agrIV			agrV (argenteus)		hld
	agrI (total)	agrB-I	agrC-I	agrD-I	agrII (total)	agrB-II	agrC-II	agrD-II	agrIII (total)	agrB-III	agrC-III	agrD-III	agrIV (total)	agrB-IV	agrC-IV	agrV-ST1850	agrV-ST2198	
	accessory gene regulator allele I				accessory gene regulator allele II				accessory gene regulator allele III				accessory gene regulator allele IV			ory gene regulator alleles from S. argenteus		haemolysin delta
**CC3960/3961**																		
BDS-53E: in silico predicted hybridisation pattern	NEG	NEG	POS	NEG	NEG	NEG	NEG	NEG	NEG	NEG	NEG	NEG	NEG	NEG	NEG	NEG	NEG	POS
Bat isolate BDS-53E	POS	NEG	POS	POS	NEG	NEG	NEG	NEG	NEG	NEG	NEG	NEG	NEG	NEG	NEG	NEG	NEG	POS
Bat isolate BDS-53B	POS	NEG	POS	POS	NEG	NEG	NEG	NEG	NEG	NEG	NEG	NEG	NEG	NEG	NEG	NEG	NEG	POS
BDS-54: in silico predicted hybridisation pattern	NEG	NEG	POS	NEG	NEG	NEG	NEG	NEG	NEG	NEG	NEG	NEG	NEG	NEG	NEG	NEG	NEG	POS
Bat isolate BDS-54	POS	NEG	POS	POS	NEG	NEG	NEG	NEG	NEG	NEG	NEG	NEG	NEG	NEG	NEG	NEG	NEG	POS
Bat isolate BDH-128	NEG	NEG	NEG	POS	NEG	NEG	NEG	NEG	NEG	NEG	NEG	NEG	NEG	NEG	NEG			POS
Bat isolate BDH-157	POS	NEG	POS	POS	NEG	NEG	NEG	NEG	NEG	NEG	NEG	NEG	NEG	NEG	NEG	AMB	NEG	POS
Bat isolate BDS-69C	AMB	NEG	AMB	POS	NEG	NEG	NEG	NEG	NEG	NEG	NEG	NEG	NEG	NEG	NEG	NEG	NEG	POS
Bat isolate BDH-147	POS	NEG	POS	POS	NEG	NEG	NEG	NEG	NEG	NEG	NEG	NEG	NEG	NEG	NEG	NEG	NEG	POS
**CC7342**																		
Zoo-28: in silico predicted hybridisation pattern	NEG	NEG	POS	NEG	NEG	NEG	NEG	NEG	NEG	NEG	NEG	NEG	NEG	NEG	NEG	NEG	NEG	POS
Finch isolate Zoo-28	POS	NEG	POS	POS	NEG	NEG	NEG	NEG	NEG	NEG	NEG	NEG	NEG	NEG	NEG	NEG	NEG	POS
**"S. singaporensis", CC6105**																		
SS21: in silico predicted hybridisation pattern	AMB	NEG	POS	AMB	NEG	NEG	NEG	NEG	NEG	NEG	NEG	NEG	NEG	NEG	NEG	NEG	NEG	POS
DSM11148_Staphylococcus sp. SS21	AMB	NEG	AMB	POS	NEG	NEG	NEG	NEG	NEG	NEG	NEG	NEG	NEG	NEG	NEG	NEG	NEG	POS
**"S. singaporensis", CC6106**																		
SS35: in silico predicted hybridisation pattern	NEG	NEG	NEG	NEG	NEG	NEG	NEG	AMB	NEG	NEG	NEG	NEG	NEG	NEG	NEG	NEG	NEG	POS
**"S. singaporensis", CC6107**																		
SS60: in silico predicted hybridisation pattern	NEG	NEG	NEG	NEG	NEG	NEG	NEG	NEG	NEG	NEG	NEG	NEG	NEG	NEG	NEG	NEG	NEG	POS
**"S. singaporensis", CC6108**																		
SS87: in silico predicted hybridisation pattern	NEG	NEG	NEG	NEG	NEG	NEG	NEG	AMB	NEG	NEG	NEG	NEG	NEG	NEG	NEG	NEG	NEG	POS
**"S. singaporensis", CC6109**																		
SS90: in silico predicted hybridisation pattern	AMB	NEG	POS	AMB	NEG	NEG	NEG	NEG	NEG	NEG	NEG	NEG	NEG	NEG	NEG	NEG	NEG	POS
SS251: in silico predicted hybridisation pattern	AMB	NEG	POS	AMB	NEG	NEG	NEG	NEG	NEG	NEG	NEG	NEG	NEG	NEG	NEG	NEG	NEG	POS
**"S. roterodami", CC6999**																		
EMCR19: in silico predicted hybridisation pattern	NEG	NEG	NEG	NEG	NEG	NEG	NEG	AMB	NEG	NEG	NEG	NEG	NEG	NEG	NEG	NEG	NEG	POS
DSM111914_Staphylococcus sp. EMCR19	NEG	NEG	NEG	NEG	NEG	NEG	NEG	NEG	NEG	NEG	NEG	NEG	NEG	NEG	NEG	NEG	NEG	POS
**S. argenteus, CC1850**																		
MSHR1132: in silico predicted hybridisation pattern	NEG	NEG	AMB	NEG	NEG	NEG	NEG	NEG	NEG	NEG	NEG	NEG	NEG	NEG	NEG	POS	NEG	POS
DSM28299	NEG	NEG	NEG	NEG	NEG	NEG	NEG	NEG	NEG	NEG	NEG	NEG	NEG	NEG	NEG	POS	NEG	POS
**S. schweitzeri, CC2022**																		
NCTC13712: in silico predicted hybridisation pattern	NEG	AMB	POS	NEG	NEG	NEG	NEG	NEG	NEG	NEG	NEG	NEG	POS	POS	POS	NEG	NEG	POS
DSM28300	NEG	POS	POS	NEG	NEG	NEG	NEG	NEG	NEG	NEG	NEG	NEG	POS	POS	POS			POS
**S. saureus, CC1**																		
MW2: in silico predicted hybridisation pattern	NEG	NEG	NEG	NEG	NEG	NEG	NEG	NEG	POS	POS	POS	POS	NEG	NEG	NEG	NEG	NEG	POS
MW2	NEG	NEG	NEG	NEG	NEG	NEG	NEG	NEG	POS	POS	AMB	POS	NEG	NEG	NEG	NEG	NEG	POS

STRAIN / ISOLATE	METHICILLIN RESISTANCE AND SCCmec TYPING																					
	ugpQ	mecA	delta_mecR1	mecR1	mecI	fudoh-PSM	cstB-SCC1 (ex Q2G1R6) (SCCmec II/III)	xylR/mecR2	mecC	blaZ (SCCmec XI)	plsSCC (COL)	mvaS-SCC	Q5HJW6	Q7A207	cstB-SCC2 (Q2G1R6)	Q9S0M4	kdpA-SCC	kdpB-SCC	kdpC-SCC	kdpD-SCC	kdpE-SCC	Q93IB7
	Glycerophosphoryl diester phosphodiesterase. Accompanies mecA in nearly all SCCmec sequences.	Modified penicillin binding protein (PBP2a)	Truncated methicillin resistance operon repressor 1. Truncated mecR1 is present in SCCmec I, IV, V, VI, VII; complete absence of mecR1 from SCCmec V, IX, X	Methicillin resistance operon repressor 1. Un-truncated sequence in SCCmec II, SCCmec III, SCCmec VIII	Methicillin-resistance regulatory protein. Present in SCCmec II (although absent from Irish SCCmec II variants C and E), SCCmec III, SCCmec VIII	Phenol soluble modulin from SCCmec	CsoR-like sulfur transferase-regulated genes B/metallo-beta-lactamase superfamily protein. Pseudogene containing two stop codons. Subtyping SCCmec II. Also present in SCCmec VIII and irregular elements	Methicillin resistance operon repressor 2, Homolog of xylose repressor. Located next to mec operon downstream of mecI (not present if mecI is truncated). Present in SCCmec II (although absent from Irish SCCmec II variants C and E), [illegible]	Alternate gene encoding a modified penicillin binding protein . Present in, and characteristic for, SCCmec XI	Beta-lactamase from SCCmec XI	Plasmin-sensitive surface protein, prevents bacterial adhesion in vitro, located in SCC, close to mec operon. Subtyping SCCmec I	Truncated 3-hydroxy-3-methylglutaryl CoA synthase. Subtyping SCCmec I, II, IV, V	Putative protein next to dru	Putative protein	CsoR-like sulfur transferase-regulated genes B/metallo-beta-lactamase superfamily protein. Present in SCCmec IVa (truncated) and SCCmec X, variably present in SCCmec I (usually present, but absent from MR1 GenBank ACZQ, Geraldine Clone)	Putative protein. Subtyping SCCmec I, SCCmec/ACME composites and SCCmec from WA40	Potassium-translocating ATPase A, chain 2	Potassium-transporting ATPase B, chain 1	Potassium-translocating ATPase C, chain 2	Sensor kinase protein	KDP operon transcriptional regulatory protein	LytTR domain DNA-binding regulator. Subtyping SCCmec III
**CC3960/3961**																						
BDS-53E: in silico predicted hybridisation pattern	NEG	NEG	NEG	NEG	NEG	NEG	NEG	NEG	NEG	NEG	NEG	NEG	NEG	NEG	NEG	NEG	NEG	NEG	NEG	NEG	NEG	NEG
Bat isolate BDS-53E	NEG	NEG	NEG	NEG	NEG	NEG	NEG	NEG	NEG	NEG	NEG	NEG	NEG	NEG	NEG	NEG	NEG	NEG	NEG	NEG	NEG	NEG
Bat isolate BDS-53B	NEG	NEG	NEG	NEG	NEG	NEG	NEG	NEG	NEG	NEG	NEG	NEG	NEG	NEG	NEG	NEG	NEG	NEG	NEG	NEG	NEG	NEG
BDS-54: in silico predicted hybridisation pattern	NEG	NEG	NEG	NEG	NEG	NEG	NEG	NEG	NEG	NEG	NEG	NEG	NEG	NEG	NEG	NEG	NEG	NEG	NEG	NEG	NEG	NEG
Bat isolate BDS-54	NEG	NEG	NEG	NEG	NEG	NEG	NEG	NEG	NEG	NEG	NEG	NEG	NEG	NEG	NEG	NEG	NEG	NEG	NEG	NEG	NEG	NEG
Bat isolate BDH-128	NEG	NEG	NEG	NEG	NEG			NEG	NEG	NEG	NEG						NEG	NEG	NEG	NEG	NEG	
Bat isolate BDH-157	NEG	NEG	NEG	NEG	NEG	NEG	NEG	NEG	NEG	NEG	NEG	NEG	NEG	NEG	NEG	NEG	NEG	NEG	NEG	NEG	NEG	NEG
Bat isolate BDS-69C	NEG	NEG	NEG	NEG	NEG	NEG	NEG	NEG	NEG	NEG	NEG	NEG	NEG	NEG	NEG	NEG	NEG	NEG	NEG	NEG	NEG	NEG
Bat isolate BDH-147	NEG	NEG	NEG	NEG	NEG	NEG	NEG	NEG	NEG	NEG	NEG	NEG	NEG	NEG	NEG	NEG	NEG	NEG	NEG	NEG	NEG	NEG
**CC7342**																						
Zoo-28: in silico predicted hybridisation pattern	NEG	NEG	NEG	NEG	NEG	NEG	NEG	NEG	NEG	NEG	NEG	NEG	NEG	NEG	NEG	NEG	NEG	NEG	NEG	NEG	NEG	NEG
Finch isolate Zoo-28	NEG	NEG	NEG	NEG	NEG	NEG	NEG	NEG	NEG	NEG	NEG	NEG	NEG	NEG	NEG	NEG	NEG	NEG	NEG	NEG	NEG	NEG
**"S. singaporensis", CC6105**																						
SS21: in silico predicted hybridisation pattern	NEG	NEG	NEG	NEG	NEG	NEG	NEG	NEG	NEG	NEG	NEG	NEG	NEG	NEG	NEG	NEG	NEG	NEG	NEG	NEG	NEG	NEG
DSM11148_Staphylococcus sp. SS21	NEG	NEG	NEG	NEG	NEG	NEG	NEG	NEG	NEG	NEG	NEG	NEG	NEG	NEG	NEG	NEG	NEG	NEG	NEG	NEG	NEG	NEG
**"S. singaporensis", CC6106**																						
SS35: in silico predicted hybridisation pattern	NEG	NEG	NEG	NEG	NEG	NEG	NEG	NEG	NEG	NEG	NEG	NEG	NEG	NEG	NEG	NEG	NEG	NEG	NEG	NEG	NEG	NEG
**"S. singaporensis", CC6107**																						
SS60: in silico predicted hybridisation pattern	NEG	NEG	NEG	NEG	NEG	NEG	NEG	NEG	NEG	NEG	NEG	NEG	NEG	NEG	NEG	NEG	NEG	NEG	NEG	NEG	NEG	NEG
**"S. singaporensis", CC6108**																						
SS87: in silico predicted hybridisation pattern	NEG	NEG	NEG	NEG	NEG	NEG	NEG	NEG	NEG	NEG	NEG	NEG	NEG	NEG	NEG	NEG	NEG	NEG	NEG	NEG	NEG	NEG
**"S. singaporensis", CC6109**																						
SS90: in silico predicted hybridisation pattern	NEG	NEG	NEG	NEG	NEG	NEG	NEG	NEG	NEG	NEG	NEG	NEG	NEG	NEG	NEG	NEG	NEG	NEG	NEG	NEG	NEG	NEG
SS251: in silico predicted hybridisation pattern	NEG	NEG	NEG	NEG	NEG	NEG	NEG	NEG	NEG	NEG	NEG	NEG	NEG	NEG	NEG	NEG	NEG	NEG	NEG	NEG	NEG	NEG
**"S. roterodami", CC6999**																						
EMCR19: in silico predicted hybridisation pattern	NEG	NEG	NEG	NEG	NEG	NEG	NEG	NEG	NEG	NEG	NEG	NEG	NEG	NEG	NEG	NEG	NEG	NEG	NEG	NEG	NEG	NEG
DSM111914_Staphylococcus sp. EMCR19	NEG	NEG	NEG	NEG	NEG	NEG	NEG	NEG	NEG	NEG	NEG	NEG	NEG	NEG	NEG	NEG	NEG	NEG	NEG	NEG	NEG	NEG
**S. argenteus, CC1850**																						
MSHR1132: in silico predicted hybridisation pattern	POS	POS	POS	NEG	NEG	NEG	NEG	NEG	NEG	NEG	NEG	POS	POS	AMB	POS	NEG	NEG	NEG	NEG	NEG	NEG	NEG
DSM28299	POS	POS	POS	NEG	NEG	NEG	NEG	NEG	neg	NEG	NEG	POS	POS	POS	POS	NEG	NEG	NEG	NEG	NEG	NEG	NEG
**S. schweitzeri, CC2022**																						
NCTC13712: in silico predicted hybridisation pattern	NEG	NEG	NEG	NEG	NEG	NEG	NEG	NEG	NEG	NEG	NEG	NEG	NEG	NEG	NEG	NEG	NEG	NEG	NEG	NEG	NEG	NEG
DSM28300	NEG	NEG	NEG	NEG	NEG			NEG	NEG	NEG	NEG						NEG	NEG	NEG	NEG	NEG	
**S. saureus, CC1**																						
MW2: in silico predicted hybridisation pattern	POS	POS	POS	NEG	NEG	NEG	NEG	NEG	NEG	NEG	NEG	POS	POS	AMB	POS	NEG	NEG	NEG	NEG	NEG	NEG	NEG
MW2	POS	POS	POS	NEG	NEG	NEG	NEG	NEG		NEG	NEG	POS	AMB	POS	POS	NEG	NEG	NEG	NEG	NEG	NEG	NEG

STRAIN / ISOLATE	METHICILLIN RESISTANCE AND SCCmec TYPING																					
	D1GU38		Q933A2	D1GU55	F9JXC0		B2Y834	B6VQU0	Q3YK51	ydhK	C1PH94	DUF1958	Q4LAG7		C5QAP8 (SCCmec XI)	Q8CU82	D3JD07	cas1		fusC (Q6GD50)	tirS	yeeA
	D1GU38	D1GU38 (TW20)			F9JXC0 (full)	F9JXC0 (trunc)							Q4LAG7 (SCCmecV, SO385)	Q4LAG7 (SCCfus 45394F/MSSA 476)				cas1 (MSHR1132)	cas1 (M06-171)			
	Subtyping SCCmec III, identification of SCCmec VT, SCCmec ZH47, SCCmec VII because of an association with (additional/second) ccrC copies	Putative protein	Putative ADP-ribosyltransferase. Subtyping SCCmec III and SCCmec IX	Putative membrane protein. Subtyping SCCmec III, additional marker for SCCmec VII	KDP operon transcriptional regulatory protein		Abortive phage resistance protein. Subtyping SCCmec IV, i.e., identification of SCCmec IV A, G, c and SCCmec MRSAZH47	Putative protein. Subtyping SCCmec IV, i.e., identification of SCCmec IVh/j	Putative protein. Subtyping SCCmec IV, i.e., identification of SCCmec IV g	Putative lipoprotein. Present in some composite elements comprising SCCmec and heavy metal resistance genes including the one in FPR3757, GenBank: CP000255.1	Putative protein	Putative protein. Subtyping SCCmec VT. Present, e.g., in PM1, GenBank BAFA but absent, e.g., in Strain 3957, GenBank AOFU	Putative protein located within SCCmec type V elements	Putative protein located within SCCfus elements	Putative protein. Identification of SCCmec XI	Putative protein. Present in some SCCmec/fus composite elements.	Putative protein. Present in some composite SCC elements	CRISPR-associated endonuclease 1. Present in MSHR1132	CRISPR-associated endonuclease 1. Present in M06/0171, GenBank HE980450.1	SCC-associated fusidic acid resistance gene. Present in "SCCfus" elements or together with mecA in composite elements.	Staphylococcal TIR-protein binding protein. Subtyping SCCfus because it is frequently, but not always, accompanying fusC	Putative DNA methyltransferase. Subtyping SCCmecIV/fus composite elements
**CC3960/3961**																						
BDS-53E: in silico predicted hybridisation pattern	NEG	NEG	NEG	NEG	NEG	NEG	NEG	NEG	NEG	NEG	NEG	NEG	NEG	NEG	NEG	NEG	NEG	NEG	NEG	NEG	NEG	NEG
Bat isolate BDS-53E	NEG	NEG	NEG	NEG	NEG	NEG	NEG	NEG	NEG	NEG	NEG	NEG	NEG	NEG	NEG	NEG	NEG	NEG	NEG	NEG	NEG	NEG
Bat isolate BDS-53B	NEG	NEG	NEG	NEG	NEG	NEG	NEG	NEG	NEG	NEG	NEG	NEG	NEG	NEG	NEG	NEG	NEG	NEG	NEG	NEG	NEG	NEG
BDS-54: in silico predicted hybridisation pattern	NEG	NEG	NEG	NEG	NEG	NEG	NEG	NEG	NEG	NEG	NEG	NEG	NEG	NEG	NEG	NEG	NEG	NEG	NEG	NEG	NEG	NEG
Bat isolate BDS-54	NEG	NEG	NEG	NEG	NEG	NEG	NEG	NEG	NEG	NEG	NEG	NEG	NEG	NEG	NEG	NEG	NEG	NEG	NEG	NEG	NEG	NEG
Bat isolate BDH-128																				NEG		
Bat isolate BDH-157	NEG	NEG	NEG	NEG	NEG	NEG	NEG	NEG	NEG	NEG	NEG	NEG	NEG	NEG	NEG	NEG	NEG	NEG	NEG	NEG	NEG	NEG
Bat isolate BDS-69C	NEG	NEG	NEG	NEG	NEG	NEG	POS	NEG	NEG	NEG	NEG	NEG	NEG	NEG	NEG	NEG	NEG	NEG	NEG	NEG	NEG	NEG
Bat isolate BDH-147	NEG	NEG	NEG	NEG	NEG	NEG	POS	NEG	NEG	NEG	NEG	NEG	NEG	NEG	NEG	NEG	NEG	NEG	NEG	NEG	NEG	NEG
**CC7342**																						
Zoo-28: in silico predicted hybridisation pattern	NEG	NEG	NEG	NEG	NEG	NEG	NEG	POS	NEG	NEG	NEG	NEG	NEG	NEG	NEG	NEG	NEG	NEG	NEG	NEG	NEG	NEG
Finch isolate Zoo-28	NEG	NEG	NEG	NEG	NEG	NEG	NEG	POS	NEG	NEG	NEG	NEG	NEG	NEG	NEG	NEG	NEG	NEG	NEG	NEG	NEG	NEG
**"S. singaporensis", CC6105**																						
SS21: in silico predicted hybridisation pattern	NEG	NEG	NEG	NEG	NEG	NEG	NEG	NEG	NEG	NEG	NEG	NEG	NEG	NEG	NEG	NEG	NEG	NEG	NEG	NEG	NEG	NEG
DSM11148_Staphylococcus sp. SS21	NEG	NEG	NEG	NEG	NEG	NEG	NEG	NEG	NEG	NEG	NEG	NEG	NEG	NEG	NEG	NEG	NEG	NEG	NEG	NEG	NEG	NEG
**"S. singaporensis", CC6106**																						
SS35: in silico predicted hybridisation pattern	NEG	NEG	NEG	NEG	NEG	NEG	NEG	NEG	NEG	NEG	NEG	NEG	NEG	NEG	NEG	NEG	NEG	NEG	NEG	NEG	NEG	NEG
**"S. singaporensis", CC6107**																						
SS60: in silico predicted hybridisation pattern	NEG	NEG	NEG	NEG	NEG	NEG	NEG	NEG	NEG	NEG	NEG	NEG	NEG	NEG	NEG	NEG	NEG	NEG	NEG	NEG	NEG	NEG
**"S. singaporensis", CC6108**																						
SS87: in silico predicted hybridisation pattern	NEG	NEG	NEG	NEG	NEG	NEG	NEG	NEG	NEG	NEG	NEG	NEG	NEG	NEG	NEG	NEG	NEG	NEG	NEG	NEG	NEG	NEG
**"S. singaporensis", CC6109**																						
SS90: in silico predicted hybridisation pattern	NEG	NEG	NEG	NEG	NEG	NEG	NEG	NEG	NEG	NEG	NEG	NEG	NEG	NEG	NEG	NEG	NEG	POS	NEG	NEG	NEG	NEG
SS251: in silico predicted hybridisation pattern	NEG	NEG	NEG	NEG	NEG	NEG	NEG	NEG	NEG	NEG	NEG	NEG	NEG	NEG	NEG	NEG	NEG	POS	NEG	NEG	NEG	NEG
**"S. roterodami", CC6999**																						
EMCR19: in silico predicted hybridisation pattern	NEG	NEG	NEG	NEG	NEG	NEG	NEG	NEG	NEG	NEG	NEG	NEG	NEG	NEG	NEG	NEG	NEG	NEG	NEG	NEG	NEG	NEG
DSM111914_Staphylococcus sp. EMCR19	NEG	NEG	NEG	NEG	NEG	NEG	NEG	NEG	NEG	NEG	NEG	NEG	NEG	NEG	NEG	NEG	NEG	NEG	NEG	NEG	NEG	NEG
**S. argenteus, CC1850**																						
MSHR1132: in silico predicted hybridisation pattern	NEG	NEG	NEG	NEG	NEG	NEG	NEG	NEG	NEG	NEG	NEG	NEG	NEG	NEG	NEG	NEG	NEG	POS	NEG	NEG	NEG	NEG
DSM28299	NEG	NEG	NEG	neg	NEG	NEG	NEG	NEG	neg	NEG	NEG	NEG	NEG	NEG	NEG	NEG	NEG	POS	NEG	NEG	NEG	NEG
**S. schweitzeri, CC2022**																						
NCTC13712: in silico predicted hybridisation pattern	NEG	NEG	NEG	NEG	NEG	NEG	NEG	NEG	NEG	NEG	NEG	NEG	NEG	NEG	NEG	NEG	NEG	NEG	NEG	NEG	NEG	NEG
DSM28300																				NEG		
**S. saureus, CC1**																						
MW2: in silico predicted hybridisation pattern	NEG	NEG	NEG	NEG	NEG	NEG	NEG	NEG	NEG	NEG	NEG	NEG	NEG	NEG	NEG	NEG	NEG	NEG	NEG	NEG	NEG	NEG
MW2	NEG	NEG	NEG	NEG	NEG	NEG	NEG	NEG	NEG	NEG	NEG	NEG	NEG	NEG	NEG	NEG	NEG	NEG	NEG	NEG	NEG	NEG

STRAIN / ISOLATE	METHICILLIN RESISTANCE AND SCCmec TYPING																	
	ACME				opp3B	opp3C	adhC	speG	ccrA/B-01		ccrA/B-02		ccrA/B-03		ccrAA/C		ccrA/B-04	
	arcA-SCC	arcB-SCC	arcC-SCC	arcD-SCC					ccrA-1	ccrB-1	ccrA-2	ccrB-2	ccrA-3	ccrB-3	ccrAA	ccrC (85-2082)	ccrA-4	ccrB-4
	Arginine deiminase	Ornithine carbamoyltransferase	Carbamate kinase	Arginine/ornithine antiporter	Oligopeptide permease, channel-forming protein	Oligopeptide permease, channel-forming protein	Alcohol dehydrogenase, zinc-containing	Spermidine N-acetyltransferase. Usually associated with ACME or composite SCCmec/ACME elements	Cassette chromosome recombinase A, type 1	Cassette chromosome recombinase B, type 1	Cassette chromosome recombinase A, type 2	Cassette chromosome recombinase B, type 2	Cassette chromosome recombinase A, type 3	Cassette chromosome recombinase B, type 3	hypoth. Protein associated with ccrC	Cassette chromosome recombinase C	Cassette chromosome recombinase A, type 4	Cassette chromosome recombinase B, type 4
**CC3960/3961**																		
BDS-53E: in silico predicted hybridisation pattern	NEG	NEG	NEG	NEG	NEG	NEG	NEG	NEG	NEG	NEG	NEG	NEG	NEG	NEG	NEG	NEG	NEG	NEG
Bat isolate BDS-53E	NEG	NEG	NEG	NEG	NEG	NEG	NEG	NEG	NEG	NEG	NEG	NEG	NEG	NEG	NEG	NEG	NEG	NEG
Bat isolate BDS-53B	NEG	NEG	NEG	NEG	NEG	NEG	NEG	NEG	NEG	NEG	NEG	NEG	NEG	NEG	NEG	NEG	NEG	NEG
BDS-54: in silico predicted hybridisation pattern	NEG	NEG	NEG	NEG	NEG	NEG	NEG	NEG	NEG	NEG	NEG	NEG	NEG	NEG	NEG	NEG	NEG	NEG
Bat isolate BDS-54	NEG	NEG	NEG	NEG	NEG	NEG	NEG	NEG	NEG	NEG	NEG	NEG	NEG	NEG	NEG	NEG	NEG	NEG
Bat isolate BDH-128	NEG	NEG	NEG	NEG					POS	NEG	NEG	NEG	NEG	NEG	NEG	NEG	NEG	NEG
Bat isolate BDH-157	NEG	NEG	NEG	NEG	NEG	NEG	NEG	NEG	NEG	NEG	NEG	NEG	NEG	NEG	NEG	NEG	NEG	NEG
Bat isolate BDS-69C	NEG	NEG	NEG	NEG	NEG	NEG	NEG	NEG	NEG	NEG	NEG	NEG	NEG	NEG	NEG	NEG	NEG	NEG
Bat isolate BDH-147	NEG	NEG	NEG	NEG	NEG	NEG	NEG	NEG	NEG	NEG	NEG	NEG	NEG	NEG	NEG	NEG	NEG	NEG
**CC7342**																		
Zoo-28: in silico predicted hybridisation pattern	NEG	NEG	NEG	NEG	NEG	NEG	NEG	NEG	NEG	NEG	NEG	NEG	NEG	NEG	NEG	NEG	NEG	NEG
Finch isolate Zoo-28	NEG	NEG	NEG	NEG	NEG	NEG	NEG	NEG	NEG	NEG	NEG	NEG	NEG	NEG	NEG	NEG	NEG	NEG
**"S. singaporensis", CC6105**																		
SS21: in silico predicted hybridisation pattern	NEG	NEG	NEG	NEG	NEG	NEG	NEG	NEG	NEG	NEG	NEG	NEG	NEG	NEG	NEG	NEG	NEG	NEG
DSM11148_Staphylococcus sp. SS21	NEG	NEG	NEG	NEG	NEG	NEG	NEG	NEG	NEG	NEG	NEG	NEG	NEG	NEG	NEG	NEG	NEG	NEG
**"S. singaporensis", CC6106**																		
SS35: in silico predicted hybridisation pattern	NEG	NEG	NEG	NEG	NEG	NEG	NEG	NEG	NEG	NEG	NEG	NEG	NEG	NEG	NEG	NEG	NEG	NEG
**"S. singaporensis", CC6107**																		
SS60: in silico predicted hybridisation pattern	NEG	NEG	NEG	NEG	NEG	NEG	NEG	NEG	NEG	NEG	NEG	NEG	NEG	NEG	NEG	NEG	NEG	NEG
**"S. singaporensis", CC6108**																		
SS87: in silico predicted hybridisation pattern	NEG	NEG	NEG	NEG	NEG	NEG	NEG	NEG	NEG	NEG	NEG	NEG	NEG	NEG	NEG	NEG	NEG	NEG
**"S. singaporensis", CC6109**																		
SS90: in silico predicted hybridisation pattern	NEG	NEG	NEG	NEG	NEG	NEG	NEG	NEG	NEG	NEG	NEG	NEG	NEG	NEG	NEG	NEG	NEG	NEG
SS251: in silico predicted hybridisation pattern	NEG	NEG	NEG	NEG	NEG	NEG	NEG	NEG	NEG	NEG	NEG	NEG	NEG	NEG	NEG	NEG	NEG	NEG
**"S. roterodami", CC6999**																		
EMCR19: in silico predicted hybridisation pattern	NEG	NEG	NEG	NEG	NEG	NEG	NEG	NEG	NEG	NEG	NEG	NEG	NEG	NEG	NEG	NEG	NEG	NEG
DSM111914_Staphylococcus sp. EMCR19	NEG	NEG	NEG	NEG	NEG	NEG	NEG	NEG	NEG	NEG	NEG	NEG	NEG	NEG	NEG	NEG	NEG	NEG
**S. argenteus, CC1850**																		
MSHR1132: in silico predicted hybridisation pattern	NEG	NEG	NEG	NEG	NEG	NEG	NEG	NEG	NEG	NEG	POS	POS	NEG	NEG	NEG	NEG	NEG	NEG
DSM28299	NEG	NEG	NEG	NEG	NEG	NEG	NEG	NEG	NEG	NEG	POS	POS	NEG	NEG	NEG	NEG	NEG	NEG
**S. schweitzeri, CC2022**																		
NCTC13712: in silico predicted hybridisation pattern	NEG	NEG	NEG	NEG	NEG	NEG	NEG	NEG	NEG	NEG	NEG	NEG	NEG	NEG	NEG	NEG	NEG	NEG
DSM28300	NEG	NEG	NEG	NEG					NEG	NEG	NEG	NEG	NEG	NEG	NEG	NEG	NEG	NEG
**S. saureus, CC1**																		
MW2: in silico predicted hybridisation pattern	NEG	NEG	NEG	NEG	NEG	NEG	NEG	NEG	NEG	NEG	POS	POS	NEG	NEG	NEG	NEG	NEG	NEG
MW2	NEG	NEG	NEG	NEG	NEG	NEG	NEG	NEG	NEG	NEG	POS	POS	NEG	NEG	NEG	NEG	NEG	NEG

STRAIN / ISOLATE	METHICILLIN RESISTANCE AND SCCmec TYPING												
	Q9XB68-dcs	Alternative SCC termini											
		SCCmec Terminus 1	SCCmec Terminus 2	SCCmec Terminus 3	SCCmec Terminus 4	SCCmec Terminus 5	SCCmec Terminus 6	SCCmec Terminus 7	SCCmec Terminus 9	SCCmec Terminus 10	SCCmec Terminus 11	SCCmec Terminus 12/13/14
	Located at the terminus of SCCmec next to orfX.This locus comprises the downstream constant segment (dcs) that in turn comprises a copy of the SCC direct repeat	SCC integration site alternate to dcs										
**CC3960/3961**												
BDS-53E: in silico predicted hybridisation pattern	NEG	NEG	NEG	NEG	NEG	NEG	NEG	NEG	NEG	NEG	NEG	NEG
Bat isolate BDS-53E	NEG	NEG	NEG	NEG	NEG	NEG	NEG	NEG	NEG	NEG	NEG	NEG
Bat isolate BDS-53B	NEG	NEG	NEG	NEG	NEG	NEG	NEG	NEG	NEG	NEG	NEG	NEG
BDS-54: in silico predicted hybridisation pattern	NEG	NEG	NEG	NEG	NEG	NEG	NEG	NEG	NEG	NEG	NEG	NEG
Bat isolate BDS-54	NEG	NEG	NEG	NEG	NEG	NEG	NEG	NEG	NEG	NEG	NEG	NEG
Bat isolate BDH-128	NEG											
Bat isolate BDH-157	NEG	NEG	NEG	NEG	NEG	NEG	NEG	NEG	NEG	NEG	NEG	NEG
Bat isolate BDS-69C	NEG	NEG	NEG	NEG	NEG	NEG	NEG	NEG	NEG	NEG	NEG	NEG
Bat isolate BDH-147	NEG	NEG	NEG	NEG	NEG	NEG	NEG	NEG	NEG	NEG	NEG	NEG
**CC7342**												
Zoo-28: in silico predicted hybridisation pattern	NEG	NEG	NEG	NEG	NEG	NEG	NEG	NEG	NEG	NEG	NEG	NEG
Finch isolate Zoo-28	NEG	NEG	NEG	NEG	NEG	NEG	NEG	NEG	NEG	NEG	NEG	NEG
**"S. singaporensis", CC6105**												
SS21: in silico predicted hybridisation pattern	NEG	NEG	NEG	NEG	NEG	NEG	NEG	NEG	NEG	NEG	NEG	NEG
DSM11148_Staphylococcus sp. SS21	NEG	NEG	NEG	NEG	NEG	NEG	NEG	NEG	NEG	NEG	NEG	NEG
**"S. singaporensis", CC6106**												
SS35: in silico predicted hybridisation pattern	NEG	NEG	NEG	NEG	NEG	NEG	NEG	NEG	NEG	NEG	NEG	NEG
**"S. singaporensis", CC6107**												
SS60: in silico predicted hybridisation pattern	NEG	NEG	NEG	NEG	NEG	NEG	NEG	NEG	NEG	NEG	NEG	NEG
**"S. singaporensis", CC6108**												
SS87: in silico predicted hybridisation pattern	NEG	NEG	NEG	NEG	NEG	NEG	NEG	NEG	NEG	NEG	NEG	NEG
**"S. singaporensis", CC6109**												
SS90: in silico predicted hybridisation pattern	NEG	NEG	NEG	NEG	NEG	NEG	NEG	NEG	NEG	NEG	NEG	NEG
SS251: in silico predicted hybridisation pattern	NEG	NEG	NEG	NEG	NEG	NEG	NEG	NEG	NEG	NEG	NEG	NEG
**"S. roterodami", CC6999**												
EMCR19: in silico predicted hybridisation pattern	NEG	NEG	NEG	NEG	NEG	NEG	NEG	NEG	NEG	NEG	NEG	NEG
DSM111914_Staphylococcus sp. EMCR19	NEG	NEG	NEG	NEG	NEG	NEG	NEG	NEG	NEG	NEG	NEG	NEG
**S. argenteus, CC1850**												
MSHR1132: in silico predicted hybridisation pattern	POS	NEG	NEG	NEG	NEG	NEG	NEG	NEG	NEG	NEG	NEG	NEG
DSM28299	POS	NEG	NEG	NEG	NEG	NEG	NEG	NEG	NEG	NEG	NEG	NEG
**S. schweitzeri, CC2022**												
NCTC13712: in silico predicted hybridisation pattern	NEG	NEG	NEG	NEG	NEG	NEG	NEG	NEG	NEG	NEG	NEG	NEG
DSM28300	NEG											
**S. saureus, CC1**												
MW2: in silico predicted hybridisation pattern	POS	NEG	NEG	NEG	NEG	NEG	NEG	NEG	NEG	NEG	NEG	NEG
MW2	POS	NEG	NEG	NEG	NEG	NEG	NEG	NEG	NEG	NEG	NEG	NEG

STRAIN / ISOLATE	HEAVY METAL RESISTANCES, SCCmec AND OTHERS																					
										arsB			arsC						cadD			
	merA	merB	mco -plasmid	copA2 -plasmid	copA2 -SCC	mco -SCC	arsA	arsD	arsD2	arsB (SCC)	arsB (chromos.)	arsB (chromos.-argenteus)	arsC (chromos.)	arsC (SCC or plasmidic)	czrB	czrC	cadA	cadC	cadD (total)	cadD (R35)	cadX (plasmid)	cadX (SCC)
	mercury resistance operon		Multi copper oxidase	Copper exporting ATPase	Copper exporting ATPase	Multi copper oxidase	arsenical pump-driving ATPase	Putative dehydrogenase	trans-acting repressor of arsenic resistance operon	arsenical pump membrane protein			arsenate reductase		zink and cobalt transporter protein	cadmium and zinc resistance gene C, heavy metal translocating P-type ATPase. Frequently associated with SCCmec elements from livestock MRSA	cadmium transporting ATPase	putative regulator of cadmium efflux	cadmium transport protein D		Putative regulator of cadmium efflux	
**CC3960/3961**																						
BDS-53E: in silico predicted hybridisation pattern	NEG	NEG	NEG	NEG	NEG	NEG	NEG	NEG	NEG	NEG	POS	NEG	NEG	NEG	POS	NEG	NEG	NEG	NEG	NEG	NEG	NEG
Bat isolate BDS-53E	NEG	NEG	NEG	NEG	NEG	NEG	NEG	NEG	NEG	NEG	POS	NEG	POS	NEG	POS	NEG	NEG	NEG	NEG	NEG	NEG	NEG
Bat isolate BDS-53B	NEG	NEG	NEG	NEG	NEG	NEG	NEG	NEG	NEG	NEG	POS	NEG	POS	NEG	POS	NEG	NEG	NEG	NEG	NEG	NEG	NEG
BDS-54: in silico predicted hybridisation pattern	NEG	NEG	NEG	NEG	NEG	NEG	NEG	NEG	NEG	NEG	POS	NEG	NEG	NEG	POS	NEG	NEG	NEG	NEG	NEG	NEG	NEG
Bat isolate BDS-54	NEG	NEG	NEG	NEG	NEG	NEG	NEG	NEG	NEG	NEG	POS	NEG	POS	NEG	POS	NEG	NEG	NEG	NEG	NEG	NEG	NEG
Bat isolate BDH-128	NEG	NEG																				
Bat isolate BDH-157	NEG	NEG	NEG	NEG	NEG	NEG	NEG	NEG	NEG	NEG	POS	NEG	POS	NEG	POS	NEG	NEG	NEG	NEG	NEG	NEG	NEG
Bat isolate BDS-69C	NEG	NEG	NEG	NEG	NEG	NEG	NEG	NEG	NEG	NEG	POS	NEG	AMB	NEG	POS	NEG	NEG	NEG	NEG	NEG	NEG	NEG
Bat isolate BDH-147	NEG	NEG	NEG	NEG	NEG	NEG	NEG	NEG	NEG	NEG	POS	NEG	POS	NEG	POS	NEG	NEG	NEG	NEG	NEG	NEG	NEG
**CC7342**																						
Zoo-28: in silico predicted hybridisation pattern	NEG	NEG	NEG	NEG	NEG	NEG	NEG	NEG	NEG	NEG	POS	NEG	NEG	NEG	POS	NEG	NEG	NEG	NEG	NEG	NEG	NEG
Finch isolate Zoo-28	NEG	NEG	NEG	NEG	NEG	NEG	NEG	NEG	NEG	NEG	POS	NEG	NEG	NEG	POS	NEG	NEG	NEG	NEG	NEG	NEG	NEG
**"S. singaporensis", CC6105**																						
SS21: in silico predicted hybridisation pattern	NEG	NEG	NEG	NEG	NEG	NEG	NEG	NEG	NEG	NEG	POS	NEG	NEG	NEG	POS	NEG	NEG	NEG	NEG	NEG	NEG	NEG
DSM11148_Staphylococcus sp. SS21	NEG	NEG	NEG	NEG	NEG	NEG	NEG	NEG	NEG	NEG	POS	NEG	NEG	NEG	POS	NEG	NEG	NEG	NEG	NEG	NEG	NEG
**"S. singaporensis", CC6106**																						
SS35: in silico predicted hybridisation pattern	NEG	NEG	NEG	NEG	NEG	NEG	NEG	NEG	NEG	NEG	POS	NEG	NEG	NEG	POS	NEG	NEG	NEG	NEG	NEG	NEG	NEG
**"S. singaporensis", CC6107**																						
SS60: in silico predicted hybridisation pattern	NEG	NEG	NEG	NEG	NEG	NEG	NEG	NEG	NEG	NEG	POS	NEG	NEG	NEG	POS	NEG	NEG	NEG	NEG	NEG	NEG	NEG
**"S. singaporensis", CC6108**																						
SS87: in silico predicted hybridisation pattern	NEG	NEG	NEG	NEG	NEG	NEG	NEG	NEG	NEG	NEG	POS	NEG	NEG	NEG	POS	NEG	NEG	NEG	NEG	NEG	NEG	NEG
**"S. singaporensis", CC6109**																						
SS90: in silico predicted hybridisation pattern	NEG	NEG	NEG	NEG	NEG	NEG	NEG	NEG	NEG	NEG	POS	NEG	NEG	NEG	POS	NEG	NEG	NEG	NEG	NEG	NEG	NEG
SS251: in silico predicted hybridisation pattern	NEG	NEG	NEG	NEG	NEG	NEG	NEG	NEG	NEG	NEG	POS	NEG	NEG	NEG	POS	NEG	NEG	NEG	NEG	NEG	NEG	NEG
**"S. roterodami", CC6999**																						
EMCR19: in silico predicted hybridisation pattern	NEG	NEG	NEG	NEG	NEG	NEG	NEG	NEG	NEG	NEG	POS	NEG	NEG	NEG	POS	NEG	NEG	NEG	NEG	NEG	NEG	NEG
DSM111914_Staphylococcus sp. EMCR19	NEG	NEG	NEG	NEG	NEG	NEG	NEG	NEG	NEG	NEG	POS	NEG	NEG	NEG	POS	NEG	NEG	NEG	NEG	NEG	NEG	NEG
**S. argenteus, CC1850**																						
MSHR1132: in silico predicted hybridisation pattern	NEG	NEG	NEG	NEG	NEG	NEG	NEG	NEG	NEG	NEG	NEG	POS	POS	NEG	POS	NEG	NEG	NEG	POS	NEG	POS	NEG
DSM28299	NEG	NEG	NEG	NEG	NEG	NEG	NEG		NEG	NEG	NEG	POS	POS	NEG	POS	NEG	NEG	NEG	POS	NEG	POS	NEG
**S. schweitzeri, CC2022**																						
NCTC13712: in silico predicted hybridisation pattern	NEG	NEG	NEG	NEG	NEG	NEG	NEG	NEG	NEG	NEG	NEG	NEG	NEG	NEG	NEG	NEG	NEG	NEG	NEG	NEG	NEG	NEG
DSM28300	NEG	NEG																				
**S. saureus, CC1**																						
MW2: in silico predicted hybridisation pattern	NEG	NEG	NEG	NEG	NEG	NEG	NEG	NEG	NEG	NEG	POS	NEG	NEG	NEG	POS	NEG	NEG	NEG	POS	NEG	POS	NEG
MW2	NEG	NEG	NEG	NEG	NEG	NEG	NEG	NEG	NEG	NEG	POS	NEG	NEG	NEG	POS	NEG	NEG	NEG	POS	NEG	POS	NEG

STRAIN / ISOLATE	RESISTANCE : PENICILLINASE			RESISTANCE : MLS-ANTIBIOTICS																
							ermC													
	blaZ	blaI	blaR	ermA	ermA-43	ermB	ermC	ermC-GM	ermF	ermT	linA/lnuA	lnuB	lsa-B	lsa-E	msrA	mefA	mph(C)	vat(A)	vat(B)	vga(A)
	beta-lactamase	beta lactamase repressor (inhibitor)	beta-lactamase regulatory protein	rRNA adenine N-6-methyl-transferase, erythromycin/clindamycin resistance		erythro-mycin/clindamycin resistance	rRNA adenine N-6-methyltrans-ferase	rRNA adenine methylase-like protein ErmGM	rRNA adenine N-6-methyltrans- ferase	rRNA adenine N-6-methyltrans- ferase	Lincosamid-Nucleotidyltransferase	lincosamide nucleotidyltransferase	lincosamide ABC transporter	lincosamide ABC transporter	energy-dependent efflux of erythro-mycin	macrolide efflux protein A	probable lysylphos-phatidyl-glycerol synthetase	virginiamycin A acetyltrans- ferase	acetyl-transferase inactivating streptogramin A	ATP binding protein, streptogramin-A-resistance
**CC3960/3961**																				
BDS-53E: in silico predicted hybridisation pattern	NEG	NEG	NEG	NEG	NEG	NEG	NEG	NEG	NEG	NEG	NEG	NEG	NEG	NEG	NEG	NEG	NEG	NEG	NEG	NEG
Bat isolate BDS-53E	NEG	NEG	NEG	NEG	NEG	NEG	NEG	NEG	NEG	NEG	NEG	NEG	NEG	NEG	NEG	NEG	NEG	NEG	NEG	NEG
Bat isolate BDS-53B	NEG	NEG	NEG	NEG	NEG	NEG	NEG	NEG	NEG	NEG	NEG	NEG	NEG	NEG	NEG	NEG	NEG	NEG	NEG	NEG
BDS-54: in silico predicted hybridisation pattern	NEG	NEG	NEG	NEG	NEG	NEG	NEG	NEG	NEG	NEG	NEG	NEG	NEG	NEG	NEG	NEG	NEG	NEG	NEG	NEG
Bat isolate BDS-54	NEG	NEG	NEG	NEG	NEG	NEG	NEG	NEG	NEG	NEG	NEG	NEG	NEG	NEG	NEG	NEG	NEG	NEG	NEG	NEG
Bat isolate BDH-128	NEG	NEG	NEG	NEG		NEG	NEG				NEG				NEG		NEG	NEG	NEG	NEG
Bat isolate BDH-157	NEG	NEG	NEG	NEG	NEG	NEG	NEG	NEG	NEG	NEG	NEG	NEG	NEG	NEG	NEG	NEG	NEG	NEG	NEG	NEG
Bat isolate BDS-69C	NEG	NEG	NEG	NEG	NEG	NEG	NEG	NEG	NEG	NEG	NEG	NEG	NEG	NEG	NEG	NEG	NEG	NEG	NEG	NEG
Bat isolate BDH-147	NEG	NEG	NEG	NEG	NEG	NEG	NEG	NEG	NEG	NEG	NEG	NEG	NEG	NEG	NEG	NEG	NEG	NEG	NEG	NEG
**CC7342**																				
Zoo-28: in silico predicted hybridisation pattern	POS	POS	AMB	NEG	NEG	NEG	NEG	NEG	NEG	NEG	NEG	NEG	NEG	NEG	NEG	NEG	NEG	NEG	NEG	NEG
Finch isolate Zoo-28	POS	POS	POS	NEG	NEG	NEG	NEG	NEG	NEG	NEG	NEG	NEG	NEG	NEG	NEG	NEG	NEG	NEG	NEG	NEG
**"S. singaporensis", CC6105**																				
SS21: in silico predicted hybridisation pattern	NEG	NEG	NEG	NEG	NEG	NEG	NEG	NEG	NEG	NEG	NEG	NEG	NEG	NEG	NEG	NEG	NEG	NEG	NEG	NEG
DSM11148_Staphylococcus sp. SS21	NEG	NEG	NEG	NEG	NEG	NEG	NEG	NEG	NEG	NEG	NEG	NEG	NEG	NEG	NEG	NEG	NEG	NEG	NEG	NEG
**"S. singaporensis", CC6106**																				
SS35: in silico predicted hybridisation pattern	NEG	NEG	NEG	NEG	NEG	NEG	NEG	NEG	NEG	NEG	NEG	NEG	NEG	NEG	NEG	NEG	NEG	NEG	NEG	NEG
**"S. singaporensis", CC6107**																				
SS60: in silico predicted hybridisation pattern	NEG	NEG	NEG	NEG	NEG	NEG	NEG	NEG	NEG	NEG	NEG	NEG	NEG	NEG	NEG	NEG	NEG	NEG	NEG	NEG
**"S. singaporensis", CC6108**																				
SS87: in silico predicted hybridisation pattern	NEG	NEG	NEG	NEG	NEG	NEG	NEG	NEG	NEG	NEG	NEG	NEG	NEG	NEG	NEG	NEG	NEG	NEG	NEG	NEG
**"S. singaporensis", CC6109**																				
SS90: in silico predicted hybridisation pattern	NEG	NEG	NEG	NEG	NEG	NEG	NEG	NEG	NEG	NEG	NEG	NEG	NEG	NEG	NEG	NEG	NEG	NEG	NEG	NEG
SS251: in silico predicted hybridisation pattern	NEG	NEG	NEG	NEG	NEG	NEG	NEG	NEG	NEG	NEG	NEG	NEG	NEG	NEG	NEG	NEG	NEG	NEG	NEG	NEG
**"S. roterodami", CC6999**																				
EMCR19: in silico predicted hybridisation pattern	NEG	NEG	NEG	NEG	NEG	NEG	NEG	NEG	NEG	NEG	NEG	NEG	NEG	NEG	NEG	NEG	NEG	NEG	NEG	NEG
DSM111914_Staphylococcus sp. EMCR19	NEG	NEG	NEG	NEG	NEG	NEG	NEG	NEG	NEG	NEG	NEG	NEG	NEG	NEG	NEG	NEG	NEG	NEG	NEG	NEG
**S. argenteus, CC1850**																				
MSHR1132: in silico predicted hybridisation pattern	POS	POS	POS	NEG	NEG	NEG	NEG	NEG	NEG	NEG	NEG	NEG	NEG	NEG	NEG	NEG	NEG	NEG	NEG	NEG
DSM28299	POS	POS	POS	NEG	NEG	NEG	NEG	NEG	NEG	NEG	NEG	NEG	NEG	NEG	NEG	NEG	NEG	NEG	NEG	NEG
**S. schweitzeri, CC2022**																				
NCTC13712: in silico predicted hybridisation pattern	NEG	NEG	NEG	NEG	NEG	NEG	NEG	NEG	NEG	NEG	NEG	NEG	NEG	NEG	NEG	NEG	NEG	NEG	NEG	NEG
DSM28300	NEG	NEG	NEG	NEG		NEG	NEG				NEG				NEG		NEG	NEG	NEG	NEG
**S. saureus, CC1**																				
MW2: in silico predicted hybridisation pattern	POS	POS	POS	NEG	NEG	NEG	NEG	NEG	NEG	NEG	NEG	NEG	NEG	NEG	NEG	NEG	NEG	NEG	NEG	NEG
MW2	POS	POS	POS	NEG	NEG	NEG	NEG	NEG	NEG	NEG	NEG	NEG	NEG	NEG	NEG	NEG	NEG	NEG	NEG	NEG

STRAIN / ISOLATE	RESISTANCE : MLS-ANTIBIOTICS						RESISTANCE : AMINOGLYSOSIDES										
									ant9				aadA		aadE		
	vga(A) (BM 3327)	vgaB	vgaC	vgaD	vgaE	vgb	aacA-aphD	aadD	ant9-C2944	ant9	ble	aad6	aadA-var1	aadA-var2	aadE-C2944	aadE	aphA3
		streptogramin A ABC	streptogramin A ABC transporter		streptogramin A ABC	virginia-mycin B hydrolase	bifunctional enzyme Aac/Aph, gentamicin resistance	amino-glycoside adenyl-transferase,tobramycin resistance	adenyltransferase AAd9		bleomycin resistance protein	aminoglycoside 6-adenylyltransferase	aminoglycoside-3'-adenylyltransferase		streptomycin aminoglycoside 6-adenyltransferase		3'5'-aminog-lycoside phospho-transferase, neo-/ kanamycin resistance
**CC3960/3961**																	
BDS-53E: in silico predicted hybridisation pattern	NEG	NEG	NEG	NEG	NEG	NEG	NEG	NEG	NEG	NEG	NEG	NEG	NEG	NEG	NEG	NEG	NEG
Bat isolate BDS-53E	NEG	NEG	NEG	NEG	NEG	NEG	NEG	NEG	NEG	NEG	NEG	NEG	NEG	NEG	NEG	NEG	NEG
Bat isolate BDS-53B	NEG	NEG	NEG	NEG	NEG	NEG	NEG	NEG	NEG	NEG	NEG	NEG	NEG	NEG	NEG	NEG	NEG
BDS-54: in silico predicted hybridisation pattern	NEG	NEG	NEG	NEG	NEG	NEG	NEG	NEG	NEG	NEG	NEG	NEG	NEG	NEG	NEG	NEG	NEG
Bat isolate BDS-54	NEG	NEG	NEG	NEG	NEG	NEG	NEG	NEG	NEG	NEG	NEG	NEG	NEG	NEG	NEG	NEG	NEG
Bat isolate BDH-128	NEG					NEG	NEG	NEG									NEG
Bat isolate BDH-157	NEG	NEG	NEG	NEG	NEG	NEG	NEG	NEG	NEG	NEG	NEG	NEG	NEG	NEG	NEG	NEG	NEG
Bat isolate BDS-69C	NEG	NEG	NEG	NEG	NEG	NEG	NEG	NEG	NEG	NEG	NEG	NEG	NEG	NEG	NEG	NEG	NEG
Bat isolate BDH-147	NEG	NEG	NEG	NEG	NEG	NEG	NEG	NEG	NEG	NEG	NEG	NEG	NEG	NEG	NEG	NEG	NEG
**CC7342**																	
Zoo-28: in silico predicted hybridisation pattern	NEG	NEG	NEG	NEG	NEG	NEG	NEG	NEG	NEG	NEG	NEG	NEG	NEG	NEG	NEG	NEG	NEG
Finch isolate Zoo-28	NEG	NEG	NEG	NEG	NEG	NEG	NEG	NEG	NEG	NEG	NEG	NEG	NEG	NEG	NEG	NEG	NEG
**"S. singaporensis", CC6105**																	
SS21: in silico predicted hybridisation pattern	NEG	NEG	NEG	NEG	NEG	NEG	NEG	NEG	NEG	NEG	NEG	NEG	NEG	NEG	NEG	NEG	NEG
DSM11148_Staphylococcus sp. SS21	NEG	NEG	NEG	NEG	NEG	NEG	NEG	NEG	NEG	NEG	NEG	NEG	NEG	NEG	NEG	NEG	NEG
**"S. singaporensis", CC6106**																	
SS35: in silico predicted hybridisation pattern	NEG	NEG	NEG	NEG	NEG	NEG	NEG	NEG	NEG	NEG	NEG	NEG	NEG	NEG	NEG	NEG	NEG
**"S. singaporensis", CC6107**																	
SS60: in silico predicted hybridisation pattern	NEG	NEG	NEG	NEG	NEG	NEG	NEG	NEG	NEG	NEG	NEG	NEG	NEG	NEG	NEG	NEG	NEG
**"S. singaporensis", CC6108**																	
SS87: in silico predicted hybridisation pattern	NEG	NEG	NEG	NEG	NEG	NEG	POS	POS	NEG	NEG	NEG	NEG	NEG	NEG	NEG	NEG	NEG
**"S. singaporensis", CC6109**																	
SS90: in silico predicted hybridisation pattern	NEG	NEG	NEG	NEG	NEG	NEG	NEG	NEG	NEG	NEG	NEG	NEG	NEG	NEG	NEG	NEG	NEG
SS251: in silico predicted hybridisation pattern	NEG	NEG	NEG	NEG	NEG	NEG	NEG	NEG	NEG	NEG	NEG	NEG	NEG	NEG	NEG	NEG	NEG
**"S. roterodami", CC6999**																	
EMCR19: in silico predicted hybridisation pattern	NEG	NEG	NEG	NEG	NEG	NEG	NEG	NEG	NEG	NEG	NEG	NEG	NEG	NEG	NEG	NEG	NEG
DSM111914_Staphylococcus sp. EMCR19	NEG	NEG	NEG	NEG	NEG	NEG	NEG	NEG	NEG	NEG	NEG	NEG	NEG	NEG	NEG	NEG	NEG
**S. argenteus, CC1850**																	
MSHR1132: in silico predicted hybridisation pattern	NEG	NEG	NEG	NEG	NEG	NEG	NEG	NEG	NEG	NEG	NEG	NEG	NEG	NEG	NEG	NEG	NEG
DSM28299	NEG	NEG	NEG	NEG	NEG	NEG	NEG	NEG	NEG	NEG	NEG	NEG	NEG	NEG	NEG	NEG	NEG
**S. schweitzeri, CC2022**																	
NCTC13712: in silico predicted hybridisation pattern	NEG	NEG	NEG	NEG	NEG	NEG	NEG	NEG	NEG	NEG	NEG	NEG	NEG	NEG	NEG	NEG	NEG
DSM28300	NEG					NEG	NEG	NEG									NEG
**S. saureus, CC1**																	
MW2: in silico predicted hybridisation pattern	NEG	NEG	NEG	NEG	NEG	NEG	NEG	NEG	NEG	NEG	NEG	NEG	NEG	NEG	NEG	NEG	NEG
MW2	NEG	NEG	NEG	NEG	NEG	NEG	NEG	NEG	NEG	NEG	NEG	NEG	NEG	NEG	NEG	NEG	NEG

STRAIN / ISOLATE	RESISTANCE : MISCELLANEOUS GENES																				
									tetM												
	sat	dfrA	dfrG	far1	mupA	mupB	tetK	tetL	tetM (com-bined)	tetM-O	tetM-S	cat	cfr	fexA	apmA	fosB	qacA	qacC	smr	vanA	vanB	vanZ
	strepto-thricine-acetyl-transferase	dihydro-folate reductase type 1	dihydrofolate reductase	fusidic acid resistence	mupirocin resistence protein	plasmidic isoleucyl-tR	tetracycline resistance	Tetracycline resistanc	tetracycline resistance			chloramphenicol acetyltransferase	23S rRNA methyltransferase	chloramphenicol/florfenicol exporter	aminocyclitol acetylt	metallothiol transferase	quaternary ammonium compound resistance protein A	quaternary ammonium compound resistance protein C		vancomycin resistance gene	vancomycin resistance gene from enterococci and Clostridium	teicoplanin resistance gene from enterococci
**CC3960/3961**																						
BDS-53E: in silico predicted hybridisation pattern	NEG	NEG	NEG	NEG	NEG	NEG	NEG	NEG	NEG	NEG	NEG	NEG	NEG	NEG	NEG	NEG	NEG	NEG	NEG	NEG	NEG	NEG
Bat isolate BDS-53E	NEG	NEG	NEG	NEG	NEG	NEG	NEG	NEG	NEG	NEG	NEG	NEG	NEG	NEG	NEG	NEG	NEG	NEG	NEG	NEG	NEG	NEG
Bat isolate BDS-53B	NEG	NEG	NEG	NEG	NEG	NEG	NEG	NEG	NEG	NEG	NEG	NEG	NEG	NEG	NEG	NEG	NEG	NEG	NEG	NEG	NEG	NEG
BDS-54: in silico predicted hybridisation pattern	NEG	NEG	NEG	NEG	NEG	NEG	NEG	NEG	NEG	NEG	NEG	NEG	NEG	NEG	NEG	NEG	NEG	NEG	NEG	NEG	NEG	NEG
Bat isolate BDS-54	NEG	NEG	NEG	NEG	NEG	NEG	NEG	NEG	NEG	NEG	NEG	NEG	NEG	NEG	NEG	NEG	NEG	NEG	NEG	NEG	NEG	NEG
Bat isolate BDH-128	NEG	NEG		NEG	NEG		NEG		NEG			NEG	NEG	NEG		NEG	NEG	NEG		NEG	NEG	NEG
Bat isolate BDH-157	NEG	NEG	NEG	NEG	NEG	NEG	NEG	NEG	NEG	NEG	NEG	NEG	NEG	NEG	NEG	NEG	NEG	NEG	NEG	NEG	NEG	NEG
Bat isolate BDS-69C	NEG	NEG	NEG	NEG	NEG	NEG	NEG	NEG	NEG	NEG	NEG	NEG	NEG	NEG	NEG	NEG	NEG	NEG	NEG	NEG	NEG	NEG
Bat isolate BDH-147	NEG	NEG	NEG	NEG	NEG	NEG	NEG	NEG	NEG	NEG	NEG	NEG	NEG	NEG	NEG	NEG	NEG	NEG	NEG	NEG	NEG	NEG
**CC7342**																						
Zoo-28: in silico predicted hybridisation pattern	NEG	NEG	NEG	NEG	NEG	NEG	NEG	NEG	POS	NEG	NEG	NEG	NEG	NEG	NEG	NEG	NEG	NEG	NEG	NEG	NEG	NEG
Finch isolate Zoo-28	NEG	NEG	NEG	NEG	NEG	NEG	NEG	NEG	POS	NEG	NEG	NEG	NEG	NEG	NEG	NEG	NEG	NEG	NEG	NEG	NEG	NEG
**"S. singaporensis", CC6105**																						
SS21: in silico predicted hybridisation pattern	NEG	NEG	NEG	NEG	NEG	NEG	NEG	NEG	NEG	NEG	NEG	NEG	NEG	NEG	NEG	NEG	NEG	NEG	NEG	NEG	NEG	NEG
DSM11148_Staphylococcus sp. SS21	NEG	NEG	NEG	NEG	NEG	NEG	NEG	NEG	NEG	NEG	NEG	NEG	NEG	NEG	NEG	NEG	NEG	NEG	NEG	NEG	NEG	NEG
**"S. singaporensis", CC6106**																						
SS35: in silico predicted hybridisation pattern	NEG	NEG	NEG	NEG	NEG	NEG	NEG	NEG	NEG	NEG	NEG	NEG	NEG	NEG	NEG	NEG	NEG	NEG	NEG	NEG	NEG	NEG
**"S. singaporensis", CC6107**																						
SS60: in silico predicted hybridisation pattern	NEG	NEG	NEG	NEG	NEG	NEG	NEG	NEG	NEG	NEG	NEG	NEG	NEG	NEG	NEG	NEG	NEG	NEG	NEG	NEG	NEG	NEG
**"S. singaporensis", CC6108**																						
SS87: in silico predicted hybridisation pattern	NEG	NEG	NEG	NEG	POS	NEG	NEG	NEG	NEG	NEG	NEG	NEG	NEG	NEG	NEG	NEG	NEG	POS	NEG	NEG	NEG	NEG
**"S. singaporensis", CC6109**																						
SS90: in silico predicted hybridisation pattern	NEG	NEG	NEG	NEG	NEG	NEG	NEG	NEG	NEG	NEG	NEG	NEG	NEG	NEG	NEG	NEG	NEG	NEG	NEG	NEG	NEG	NEG
SS251: in silico predicted hybridisation pattern	NEG	NEG	NEG	NEG	NEG	NEG	NEG	NEG	NEG	NEG	NEG	NEG	NEG	NEG	NEG	NEG	NEG	NEG	NEG	NEG	NEG	NEG
**"S. roterodami", CC6999**																						
EMCR19: in silico predicted hybridisation pattern	NEG	NEG	NEG	NEG	NEG	NEG	NEG	NEG	NEG	NEG	NEG	NEG	NEG	NEG	NEG	NEG	NEG	NEG	NEG	NEG	NEG	NEG
DSM111914_Staphylococcus sp. EMCR19	NEG	NEG	NEG	NEG	NEG	NEG	NEG	NEG	NEG	NEG	NEG	NEG	NEG	NEG	NEG	NEG	NEG	NEG	NEG	NEG	NEG	NEG
**S. argenteus, CC1850**																						
MSHR1132: in silico predicted hybridisation pattern	NEG	NEG	NEG	NEG	NEG	NEG	NEG	NEG	NEG	NEG	NEG	NEG	NEG	NEG	NEG	POS	POS	NEG	NEG	NEG	NEG	NEG
DSM28299	NEG	NEG	NEG	NEG	NEG	NEG	NEG	NEG	NEG	NEG	NEG	NEG	NEG	NEG	NEG	POS	POS	NEG	NEG	NEG	NEG	NEG
**S. schweitzeri, CC2022**																						
NCTC13712: in silico predicted hybridisation pattern	NEG	NEG	NEG	NEG	NEG	NEG	NEG	NEG	NEG	NEG	NEG	NEG	NEG	NEG	NEG	POS	NEG	NEG	NEG	NEG	NEG	NEG
DSM28300	NEG	NEG		NEG	NEG		NEG		NEG			NEG	NEG	NEG		NEG	NEG	NEG		NEG	NEG	NEG
**S. saureus, CC1**																						
MW2: in silico predicted hybridisation pattern	NEG	NEG	NEG	NEG	NEG	NEG	NEG	NEG	NEG	NEG	NEG	NEG	NEG	NEG	NEG	NEG	NEG	NEG	NEG	NEG	NEG	NEG
MW2	NEG	NEG	NEG	NEG	NEG	NEG	NEG	NEG	NEG	NEG	NEG	NEG	NEG	NEG	NEG	NEG	NEG	NEG	NEG	NEG	NEG	NEG

STRAIN / ISOLATE	VIRULENCE : TOX.SCHOCK.T.			VIRULENCE : ENTEROTOXINS																	
	tst1			seA			seB	seC	seD	seD2	seE	seH	seJ	seK	seL	seN2	seQ	seR	seS	seT	seU2	seW
	tst1 (consensus)	tst1 ("human" allele)	tst1 ("bovine" allele)	entA	entA (320E)	entA (N315) / entP																
	toxic shock syndrome toxin 1			Enterotoxin A	Enterotoxin A, allele from strain 320E	Enterotoxin A, allele from strain N315 = Entero-toxin P	Enterotoxin B	Enterotoxin C	Enterotoxin D	Enterotoxin similar to EntD	Enterotoxin E	Enterotoxin H	Enterotoxin J	Enterotoxin K	Enterotoxin L	Putative Enterotoxin N2	Enterotoxin Q	Enterotoxin R	Enterotoxin S	Enterotoxin T	Enterotoxin U2	Putative Enterotoxin
**CC3960/3961**																						
BDS-53E: in silico predicted hybridisation pattern	NEG	NEG	NEG	NEG	NEG	NEG	NEG	NEG	NEG	NEG	NEG	NEG	NEG	NEG	NEG	NEG	NEG	NEG	NEG	NEG	NEG	NEG
Bat isolate BDS-53E	NEG	NEG	NEG	NEG	NEG	NEG	NEG	NEG	NEG	NEG	NEG	NEG	NEG	NEG	NEG	NEG	NEG	NEG	NEG	NEG	NEG	NEG
Bat isolate BDS-53B	NEG	NEG	NEG	NEG	NEG	NEG	NEG	NEG	NEG	NEG	NEG	NEG	NEG	NEG	NEG	NEG	NEG	NEG	NEG	NEG	NEG	NEG
BDS-54: in silico predicted hybridisation pattern	NEG	NEG	NEG	NEG	NEG	NEG	NEG	NEG	NEG	NEG	NEG	NEG	NEG	NEG	NEG	NEG	NEG	NEG	NEG	NEG	NEG	NEG
Bat isolate BDS-54	NEG	NEG	NEG	NEG	NEG	NEG	NEG	NEG	NEG	NEG	NEG	NEG	NEG	NEG	NEG	NEG	NEG	NEG	NEG	NEG	NEG	NEG
Bat isolate BDH-128	NEG	NEG	NEG	NEG	NEG	NEG	NEG	NEG	NEG		NEG	NEG	NEG	NEG	NEG		NEG	NEG				
Bat isolate BDH-157	NEG	NEG	NEG	NEG	AMB	NEG	NEG	NEG	NEG	NEG	NEG	NEG	NEG	NEG	NEG	NEG	NEG	NEG	NEG	NEG	NEG	NEG
Bat isolate BDS-69C	NEG	NEG	NEG	NEG	NEG	NEG	NEG	NEG	NEG	NEG	NEG	NEG	NEG	NEG	NEG	NEG	NEG	NEG	NEG	NEG	NEG	NEG
Bat isolate BDH-147	NEG	NEG	NEG	NEG	NEG	NEG	NEG	NEG	NEG	NEG	NEG	NEG	NEG	NEG	NEG	NEG	NEG	NEG	NEG	NEG	NEG	NEG
**CC7342**																						
Zoo-28: in silico predicted hybridisation pattern	NEG	NEG	NEG	NEG	NEG	NEG	NEG	NEG	NEG	NEG	NEG	NEG	NEG	NEG	NEG	NEG	NEG	NEG	NEG	NEG	NEG	NEG
Finch isolate Zoo-28	NEG	NEG	NEG	NEG	NEG	NEG	NEG	NEG	NEG	NEG	NEG	NEG	NEG	NEG	NEG	NEG	NEG	NEG	NEG	NEG	NEG	NEG
**"S. singaporensis", CC6105**																						
SS21: in silico predicted hybridisation pattern	NEG	NEG	NEG	NEG	NEG	NEG	NEG	NEG	NEG	NEG	NEG	NEG	NEG	NEG	NEG	NEG	NEG	NEG	NEG	NEG	NEG	NEG
DSM11148_Staphylococcus sp. SS21	NEG	NEG	NEG	NEG	NEG	NEG	NEG	NEG	NEG	NEG	NEG	NEG	NEG	NEG	NEG	NEG	NEG	NEG	NEG	NEG	NEG	NEG
**"S. singaporensis", CC6106**																						
SS35: in silico predicted hybridisation pattern	NEG	NEG	NEG	NEG	NEG	NEG	NEG	NEG	NEG	NEG	NEG	NEG	NEG	NEG	NEG	NEG	NEG	NEG	NEG	NEG	NEG	NEG
**"S. singaporensis", CC6107**																						
SS60: in silico predicted hybridisation pattern	NEG	NEG	NEG	NEG	NEG	NEG	NEG	NEG	NEG	NEG	NEG	NEG	NEG	NEG	NEG	NEG	NEG	NEG	NEG	NEG	NEG	NEG
**"S. singaporensis", CC6108**																						
SS87: in silico predicted hybridisation pattern	NEG	NEG	NEG	NEG	NEG	NEG	NEG	NEG	NEG	NEG	NEG	NEG	NEG	NEG	NEG	NEG	NEG	NEG	NEG	NEG	NEG	NEG
**"S. singaporensis", CC6109**																						
SS90: in silico predicted hybridisation pattern	NEG	NEG	NEG	NEG	NEG	NEG	NEG	NEG	NEG	NEG	NEG	NEG	NEG	NEG	NEG	NEG	NEG	NEG	NEG	NEG	NEG	NEG
SS251: in silico predicted hybridisation pattern	NEG	NEG	NEG	NEG	NEG	NEG	NEG	NEG	NEG	NEG	NEG	NEG	NEG	NEG	NEG	NEG	NEG	NEG	NEG	NEG	NEG	NEG
**"S. roterodami", CC6999**																						
EMCR19: in silico predicted hybridisation pattern	NEG	NEG	NEG	NEG	NEG	NEG	NEG	NEG	NEG	NEG	NEG	NEG	NEG	NEG	NEG	NEG	NEG	NEG	NEG	NEG	NEG	NEG
DSM111914_Staphylococcus sp. EMCR19	NEG	NEG	NEG	NEG	NEG	NEG	NEG	NEG	NEG	NEG	NEG	NEG	NEG	NEG	NEG	NEG	NEG	NEG	NEG	NEG	NEG	NEG
**S. argenteus, CC1850**																						
MSHR1132: in silico predicted hybridisation pattern	NEG	NEG	NEG	NEG	NEG	NEG	NEG	NEG	NEG	NEG	NEG	NEG	NEG	NEG	NEG	NEG	NEG	NEG	NEG	NEG	NEG	NEG
DSM28299	NEG	NEG	NEG	NEG	NEG	NEG	NEG	NEG	NEG	NEG	NEG	NEG	NEG	NEG	NEG	NEG	NEG	NEG	NEG	NEG	NEG	NEG
**S. schweitzeri, CC2022**																						
NCTC13712: in silico predicted hybridisation pattern	NEG	NEG	NEG	NEG	NEG	NEG	NEG	NEG	NEG	NEG	NEG	NEG	NEG	NEG	NEG	NEG	NEG	NEG	NEG	NEG	POS	NEG
DSM28300	NEG	NEG	NEG	NEG	NEG	NEG	NEG	NEG	NEG		NEG	NEG	NEG	NEG	NEG		NEG	NEG				
**S. saureus, CC1**																						
MW2: in silico predicted hybridisation pattern	NEG	NEG	NEG	POS	NEG	NEG	NEG	POS	NEG	NEG	NEG	POS	NEG	POS	POS	NEG	POS	NEG	NEG	NEG	NEG	NEG
MW2	NEG	NEG	NEG	POS	NEG	NEG	NEG	POS	NEG	NEG	NEG	POS	NEG	POS	POS	NEG	POS	NEG	NEG	NEG	NEG	NEG

STRAIN / ISOLATE	VIRULENCE : ENTEROTOXINS								VIRULENCE : HLG AND LEUKOCIDINS														
										lukS											lukY		
	egc (total)	selg	seli	selm	sen	selo	selu	ORF CM14	lukF	lukS	lukS (ST22+ST45)	hlgA	lukF/S (int)	lukF-PV	lukS-PV	lukF-PV (P83)	lukM	lukD	lukE	lukX	lukY	lukY (ST30+ST45)	lukY (ST1850)
	egc cluster	Enterotoxin G	Enterotoxin I	Enterotoxin M	Enterotoxin N	Enterotoxin O	Enterotoxin U and/or Y	Enterotoxin-like protein ORF CM14	Haemolysin gamma / leukocidin, component B	Haemolysin gamma / leukocidin, component C		Haemolysin gamma,	intermedius group leukocidin	Panton Valentine leukocidin F component	Panton Valentine leukocidin S component	F component from ruminant leukocidin	S component from ruminant leukocidin	leukocidin D component	leukocidin E component	leukocidin/ haemolysin toxin family protein	leukocidin/haemolysin toxin family protein		
**CC3960/3961**																							
BDS-53E: in silico predicted hybridisation pattern	NEG	NEG	NEG	NEG	NEG	NEG	NEG	NEG	NEG	AMB	AMB	AMB	NEG	NEG	NEG	NEG	NEG	NEG	NEG	POS	NEG	NEG	NEG
Bat isolate BDS-53E	NEG	NEG	NEG	NEG	NEG	NEG	NEG	NEG	NEG	AMB	POS	POS	NEG	NEG	NEG	NEG	NEG	NEG	NEG	NEG	NEG	NEG	NEG
Bat isolate BDS-53B	NEG	NEG	NEG	NEG	NEG	NEG	NEG	NEG	NEG	NEG	POS	POS	NEG	NEG	NEG	NEG	NEG	NEG	NEG	NEG	NEG	NEG	NEG
BDS-54: in silico predicted hybridisation pattern	NEG	NEG	NEG	NEG	NEG	NEG	NEG	NEG	NEG	AMB	AMB	NEG	NEG	NEG	NEG	NEG	NEG	NEG	NEG	POS	NEG	NEG	NEG
Bat isolate BDS-54	NEG	NEG	NEG	NEG	NEG	NEG	NEG	NEG	NEG	NEG	POS	NEG	NEG	NEG	NEG	NEG	NEG	NEG	NEG	NEG	NEG	NEG	NEG
Bat isolate BDH-128	NEG	NEG	NEG	NEG		NEG	NEG	NEG	NEG	NEG	NEG	NEG		NEG	NEG	NEG	NEG	NEG	NEG	NEG	NEG	NEG	
Bat isolate BDH-157	NEG	NEG	NEG	NEG	NEG	NEG	NEG	NEG	NEG	AMB	POS	NEG	NEG	NEG	NEG	NEG	NEG	NEG	NEG	NEG	NEG	NEG	NEG
Bat isolate BDS-69C	NEG	NEG	NEG	NEG	NEG	NEG	NEG	NEG	NEG	NEG	POS	AMB	NEG	NEG	NEG	NEG	NEG	NEG	NEG	NEG	NEG	NEG	NEG
Bat isolate BDH-147	NEG	NEG	NEG	NEG	NEG	NEG	NEG	NEG	NEG	AMB	POS	POS	NEG	NEG	NEG	NEG	NEG	NEG	NEG	NEG	NEG	NEG	NEG
**CC7342**																							
Zoo-28: in silico predicted hybridisation pattern	NEG	NEG	NEG	NEG	NEG	NEG	NEG	NEG	NEG	AMB	AMB	AMB	NEG	NEG	NEG	NEG	NEG	POS	NEG	POS	AMB	NEG	NEG
Finch isolate Zoo-28	NEG	NEG	NEG	NEG	NEG	NEG	NEG	NEG	NEG	NEG	NEG	NEG	NEG	NEG	NEG	NEG	NEG	POS	NEG	AMB	AMB	NEG	NEG
**"S. singaporensis", CC6105**																							
SS21: in silico predicted hybridisation pattern	NEG	NEG	NEG	NEG	NEG	NEG	NEG	NEG	NEG	AMB	AMB	AMB	NEG	NEG	NEG	NEG	NEG	NEG	NEG	POS	NEG	NEG	NEG
DSM11148_Staphylococcus sp. SS21	NEG	NEG	NEG	NEG	NEG	NEG	NEG	NEG	NEG	NEG	POS	NEG	NEG	NEG	NEG	NEG	NEG	NEG	NEG	NEG	NEG	NEG	NEG
**"S. singaporensis", CC6106**																							
SS35: in silico predicted hybridisation pattern	NEG	NEG	NEG	NEG	NEG	NEG	NEG	NEG	NEG	AMB	AMB	AMB	NEG	NEG	NEG	NEG	NEG	NEG	NEG	POS	NEG	NEG	NEG
**"S. singaporensis", CC6107**																							
SS60: in silico predicted hybridisation pattern	POS	POS	POS	POS	NEG	POS	POS	NEG	NEG	AMB	AMB	AMB	NEG	NEG	NEG	NEG	NEG	POS	NEG	POS	NEG	NEG	NEG
**"S. singaporensis", CC6108**																							
SS87: in silico predicted hybridisation pattern	NEG	NEG	NEG	NEG	NEG	NEG	NEG	NEG	NEG	AMB	AMB	AMB	NEG	NEG	NEG	NEG	NEG	POS	NEG	POS	AMB	NEG	NEG
**"S. singaporensis", CC6109**																							
SS90: in silico predicted hybridisation pattern	NEG	NEG	NEG	NEG	NEG	NEG	NEG	NEG	NEG	AMB	AMB	AMB	NEG	NEG	NEG	NEG	NEG	POS	NEG	POS	NEG	NEG	NEG
SS251: in silico predicted hybridisation pattern	NEG	NEG	NEG	NEG	NEG	NEG	NEG	NEG	NEG	AMB	AMB	AMB	NEG	NEG	NEG	NEG	NEG	POS	NEG	POS	NEG	NEG	NEG
**"S. roterodami", CC6999**																							
EMCR19: in silico predicted hybridisation pattern	NEG	NEG	NEG	NEG	NEG	NEG	NEG	NEG	NEG	AMB	AMB	AMB	NEG	NEG	NEG	NEG	NEG	POS	NEG	POS	AMB	NEG	NEG
DSM111914_Staphylococcus sp. EMCR19	NEG	NEG	NEG	NEG	NEG	NEG	NEG	NEG	NEG	NEG	POS	NEG	NEG	NEG	NEG	NEG	NEG	AMB	NEG	AMB	AMB	NEG	NEG
**S. argenteus, CC1850**																							
MSHR1132: in silico predicted hybridisation pattern	POS	AMB	POS	POS	POS	POS	POS	NEG	NEG	NEG	NEG	NEG	NEG	NEG	NEG	NEG	NEG	NEG	NEG	NEG	NEG	NEG	POS
DSM28299	POS	POS	POS	POS	POS	POS	POS	NEG	NEG	NEG	NEG	NEG	NEG	NEG	NEG	NEG	NEG	NEG	NEG	NEG	NEG	NEG	POS
**S. schweitzeri, CC2022**																							
NCTC13712: in silico predicted hybridisation pattern	POS	POS	POS	POS	POS	AMB	POS	NEG	POS	NEG	NEG	AMB	NEG	NEG	NEG	NEG	NEG	NEG	NEG	AMB	NEG	NEG	NEG
DSM28300	POS	POS	POS	POS		AMB	POS	NEG	POS	AMB	AMB	NEG		NEG	NEG	NEG	NEG	NEG	NEG	NEG	NEG	NEG	
**S. saureus, CC1**																							
MW2: in silico predicted hybridisation pattern	NEG	NEG	NEG	NEG	NEG	NEG	NEG	NEG	POS	POS	AMB	POS	NEG	POS	POS	NEG	NEG	POS	POS	POS	POS	NEG	NEG
MW2	NEG	NEG	NEG	NEG	NEG	NEG	NEG	NEG	POS	POS	NEG	POS	NEG	POS	POS	NEG	NEG	POS	POS	POS	POS	NEG	NEG

STRAIN / ISOLATE	VIRULENCE : HAEMOLYSINS								VIRULENCE : HLB-CONV PHAGES			VIRULENCE : OTHER FACTORS								
			hlIII		hlb															
	corB (=hl)	hla	hlIII (cons)	hlIII (other than RF122)	hlb-probe 1	hlb-probe 2	hlb-probe 3	un-truncated hlb	sak	chp	scn	etA	etB	etD	etE / "etD2"	edinA	edinB	edinC	esxA	esxB
	Putative membrane protein	Haemolysin alpha	Putative membrane protein		haemolysin beta				staphylo-kinase	chemotaxis-inhibiting protein (CHIPS)	Staphyl. Comple-ment inhibitor	exfoliative toxin serotype A	exfoliative toxin serotype B	exfoliative toxin D	exfoliative toxin D2	epidermal cell differen-tiation inhibitor	epidermal cell differen-tiation inhibitor B	epidermal cell differen-tiation inhibitor C	virulence factor esxA	virulence factor esxB
**CC3960/3961**																				
BDS-53E: in silico predicted hybridisation pattern	NEG	NEG	NEG	NEG	AMB	AMB	NEG	NEG	NEG	NEG	NEG	NEG	NEG	NEG	NEG	NEG	NEG	NEG	POS	NEG
Bat isolate BDS-53E	NEG	NEG	NEG	NEG	NEG	POS	NEG	NEG	NEG	NEG	NEG	NEG	NEG	NEG	NEG	NEG	NEG	NEG	POS	NEG
Bat isolate BDS-53B	NEG	NEG	NEG	NEG	NEG	POS	NEG	NEG	NEG	NEG	NEG	NEG	NEG	NEG	NEG	NEG	NEG	NEG	POS	NEG
BDS-54: in silico predicted hybridisation pattern	NEG	NEG	NEG	NEG	AMB	AMB	NEG	NEG	NEG	NEG	NEG	NEG	NEG	NEG	NEG	NEG	NEG	NEG	POS	POS
Bat isolate BDS-54	NEG	NEG	NEG	NEG	NEG	POS	NEG	NEG	NEG	NEG	NEG	NEG	NEG	NEG	NEG	NEG	NEG	NEG	POS	POS
Bat isolate BDH-128	NEG	NEG	NEG	NEG	NEG	NEG	NEG	NEG	NEG	NEG	NEG	NEG	NEG	NEG		NEG	NEG	NEG		
Bat isolate BDH-157	NEG	NEG	NEG	NEG	NEG	POS	NEG	NEG	NEG	NEG	NEG	NEG	NEG	NEG	NEG	NEG	NEG	NEG	POS	POS
Bat isolate BDS-69C	NEG	NEG	NEG	NEG	NEG	POS	NEG	NEG	NEG	NEG	NEG	NEG	NEG	NEG	NEG	NEG	NEG	NEG	POS	POS
Bat isolate BDH-147	NEG	NEG	NEG	NEG	NEG	POS	NEG	NEG	NEG	NEG	NEG	NEG	NEG	NEG	NEG	NEG	NEG	NEG	POS	POS
**CC7342**																				
Zoo-28: in silico predicted hybridisation pattern	NEG	NEG	NEG	NEG	NEG	NEG	NEG	NEG	NEG	NEG	NEG	NEG	NEG	NEG	NEG	NEG	NEG	NEG	POS	POS
Finch isolate Zoo-28	NEG	NEG	NEG	NEG	NEG	NEG	NEG	NEG	NEG	NEG	NEG	NEG	NEG	NEG	NEG	NEG	NEG	NEG	POS	POS
**"S. singaporensis", CC6105**																				
SS21: in silico predicted hybridisation pattern	NEG	NEG	NEG	NEG	AMB	AMB	NEG	NEG	NEG	NEG	NEG	NEG	NEG	NEG	NEG	NEG	NEG	NEG	POS	POS
DSM11148_Staphylococcus sp. SS21	NEG	NEG	NEG	NEG	NEG	AMB	NEG	NEG	NEG	NEG	NEG	NEG	NEG	NEG	NEG	NEG	NEG	NEG	POS	POS
**"S. singaporensis", CC6106**																				
SS35: in silico predicted hybridisation pattern	NEG	NEG	NEG	NEG	NEG	NEG	NEG	NEG	NEG	NEG	NEG	NEG	NEG	NEG	NEG	NEG	NEG	NEG	POS	NEG
**"S. singaporensis", CC6107**																				
SS60: in silico predicted hybridisation pattern	NEG	AMB	NEG	NEG	AMB	AMB	NEG	NEG	NEG	NEG	NEG	NEG	NEG	NEG	NEG	NEG	NEG	NEG	POS	NEG
**"S. singaporensis", CC6108**																				
SS87: in silico predicted hybridisation pattern	NEG	NEG	NEG	NEG	NEG	NEG	NEG	NEG	NEG	NEG	NEG	NEG	NEG	NEG	NEG	NEG	NEG	NEG	POS	NEG
**"S. singaporensis", CC6109**																				
SS90: in silico predicted hybridisation pattern	NEG	NEG	NEG	NEG	AMB	AMB	NEG	NEG	NEG	NEG	NEG	NEG	NEG	NEG	NEG	NEG	NEG	NEG	POS	POS
SS251: in silico predicted hybridisation pattern	NEG	NEG	NEG	NEG	AMB	AMB	NEG	NEG	NEG	NEG	NEG	NEG	NEG	NEG	NEG	NEG	NEG	NEG	POS	POS
**"S. roterodami", CC6999**																				
EMCR19: in silico predicted hybridisation pattern	NEG	NEG	NEG	NEG	AMB	NEG	AMB	NEG	NEG	NEG	NEG	NEG	NEG	POS	NEG	NEG	POS	NEG	POS	NEG
DSM111914_Staphylococcus sp. EMCR19	NEG	NEG	NEG	NEG	NEG	NEG	NEG	NEG	NEG	NEG	NEG	NEG	NEG	POS	NEG	NEG	POS	NEG	POS	NEG
**S. argenteus, CC1850**																				
MSHR1132: in silico predicted hybridisation pattern	NEG	NEG	NEG	NEG	AMB	NEG	NEG	NEG	POS	NEG	POS	NEG	NEG	NEG	NEG	NEG	NEG	NEG	POS	POS
DSM28299	NEG	NEG	NEG	NEG	NEG	NEG	NEG	NEG	POS	NEG	POS	NEG	NEG	NEG	NEG	NEG	NEG	NEG	POS	POS
**S. schweitzeri, CC2022**																				
NCTC13712: in silico predicted hybridisation pattern	AMB	AMB	NEG	AMB	POS	AMB	AMB	NEG	NEG	NEG	NEG	NEG	NEG	NEG	NEG	NEG	POS	NEG	POS	NEG
DSM28300	AMB	NEG	NEG	NEG	POS	POS	POS	NEG	NEG	NEG	NEG	NEG	NEG	NEG		NEG	POS	NEG		
**S. saureus, CC1**																				
MW2: in silico predicted hybridisation pattern	POS	POS	POS	POS	POS	POS	POS	POS	POS	NEG	POS	NEG	NEG	NEG	NEG	NEG	NEG	NEG	POS	POS
MW2	POS	POS	POS	POS	AMB	POS	AMB	NEG	POS	NEG	POS	NEG	NEG	NEG	NEG	NEG	NEG	NEG	POS	POS

STRAIN / ISOLATE	VIRULENCE : PROTEASES										VIRULENCE : STAPHYLOCOCCAL SUPERANTIGEN/ENTEROTOXIN-LIKE GENES (SET/SSL)											
	aur			splA	splB	splE	sspA	sspB	sspP		setC / selX	ssl01						ssl02		ssl03		
	aur (cons)	aur (Other than MRSA252)	aur (MRSA252)						sspP (cons)	sspP (other than ST93)		ssl01/set6 (COL)	ssl01/set6 (Mu50+ N315)	ssl01/set6 (MW2+ MSSA476)	ssl01/set6 (MRSA252)	ssl01/set6 (RF122)	ssl01/set6 (other alleles)	ssl02/set7	ssl02/set7 (MRSA252)	ssl03/set8_probe 1	ssl03/set8_probe 2	ssl03/set8 (MRSA252, SAR0424)
	aureolysin			serin- protease A	serin- protease B	serin- protease E	glutamylendopeptidase	Staphopain B, protease	Staphopain A (Staphylopain A), protease		Staphyl. exotoxin-like protein	Staphylococcal superantigen-like protein 1						Staphylococcal superantigen-like protein 2		Staphylococcal superantigen-like protein 3		
**CC3960/3961**																						
BDS-53E: in silico predicted hybridisation pattern	AMB	NEG	AMB	NEG	NEG	NEG	NEG	NEG	NEG	NEG	POS	NEG	NEG	NEG	NEG	NEG	NEG	NEG	NEG	NEG	NEG	NEG
Bat isolate BDS-53E	NEG	NEG	NEG	NEG	NEG	NEG	NEG	NEG	NEG	NEG	POS	NEG	NEG	NEG	NEG	NEG	NEG	NEG	NEG	NEG	NEG	NEG
Bat isolate BDS-53B	NEG	NEG	NEG	NEG	NEG	NEG	NEG	NEG	NEG	NEG	POS	NEG	NEG	NEG	NEG	NEG	NEG	NEG	NEG	NEG	NEG	NEG
BDS-54: in silico predicted hybridisation pattern	AMB	NEG	AMB	NEG	NEG	NEG	NEG	NEG	NEG	NEG	POS	NEG	NEG	NEG	NEG	NEG	NEG	NEG	NEG	NEG	NEG	NEG
Bat isolate BDS-54	NEG	NEG	NEG	NEG	NEG	NEG	NEG	NEG	NEG	NEG	POS	NEG	NEG	NEG	NEG	NEG	NEG	NEG	NEG	NEG	NEG	NEG
Bat isolate BDH-128	NEG	NEG	NEG	NEG	NEG	NEG	NEG	NEG	NEG	NEG	POS	NEG	NEG	NEG	NEG	NEG	NEG	NEG	NEG	NEG	NEG	NEG
Bat isolate BDH-157	NEG	NEG	NEG	NEG	NEG	NEG	NEG	NEG	NEG	NEG	POS	NEG	NEG	NEG	NEG	NEG	NEG	NEG	NEG	NEG	NEG	NEG
Bat isolate BDS-69C	NEG	NEG	NEG	NEG	NEG	NEG	NEG	NEG	NEG	NEG	POS	NEG	NEG	NEG	NEG	NEG	NEG	NEG	NEG	NEG	NEG	NEG
Bat isolate BDH-147	NEG	NEG	NEG	NEG	NEG	NEG	NEG	NEG	NEG	NEG	POS	NEG	NEG	NEG	NEG	NEG	NEG	NEG	NEG	NEG	NEG	NEG
**CC7342**																						
Zoo-28: in silico predicted hybridisation pattern	AMB	NEG	AMB	NEG	NEG	NEG	NEG	NEG	NEG	NEG	POS	NEG	NEG	NEG	NEG	NEG	POS	POS	NEG	NEG	NEG	NEG
Finch isolate Zoo-28	NEG	NEG	NEG	NEG	NEG	NEG	NEG	NEG	NEG	NEG	POS	NEG	NEG	NEG	NEG	NEG	POS	POS	NEG	NEG	NEG	NEG
**"S. singaporensis", CC6105**																						
SS21: in silico predicted hybridisation pattern	AMB	NEG	AMB	NEG	NEG	NEG	NEG	NEG	NEG	NEG	POS	NEG	NEG	NEG	NEG	NEG	NEG	POS	NEG	NEG	NEG	NEG
DSM11148_Staphylococcus sp. SS21	NEG	NEG	NEG	NEG	NEG	NEG	NEG	NEG	NEG	NEG	POS	NEG	NEG	NEG	NEG	NEG	POS	POS	NEG	NEG	NEG	NEG
**"S. singaporensis", CC6106**																						
SS35: in silico predicted hybridisation pattern	AMB	NEG	AMB	NEG	NEG	NEG	NEG	NEG	NEG	NEG	POS	NEG	NEG	NEG	NEG	NEG	POS	POS	NEG	NEG	NEG	NEG
**"S. singaporensis", CC6107**																						
SS60: in silico predicted hybridisation pattern	AMB	NEG	AMB	NEG	NEG	NEG	NEG	NEG	NEG	NEG	POS	NEG	NEG	POS	NEG	NEG	NEG	POS	NEG	NEG	NEG	NEG
**"S. singaporensis", CC6108**																						
SS87: in silico predicted hybridisation pattern	AMB	NEG	AMB	NEG	NEG	NEG	NEG	NEG	NEG	NEG	POS	NEG	NEG	NEG	NEG	NEG	POS	POS	NEG	NEG	NEG	NEG
**"S. singaporensis", CC6109**																						
SS90: in silico predicted hybridisation pattern	AMB	NEG	AMB	NEG	NEG	NEG	NEG	NEG	NEG	NEG	POS	NEG	NEG	NEG	NEG	NEG	POS	POS	NEG	NEG	NEG	NEG
SS251: in silico predicted hybridisation pattern	AMB	NEG	AMB	NEG	NEG	NEG	NEG	NEG	NEG	NEG	POS	NEG	NEG	NEG	NEG	NEG	POS	POS	NEG	NEG	NEG	NEG
**"S. roterodami", CC6999**																						
EMCR19: in silico predicted hybridisation pattern	AMB	NEG	AMB	AMB	POS	POS	NEG	NEG	NEG	NEG	AMB	NEG	NEG	NEG	NEG	NEG	POS	NEG	NEG	NEG	NEG	NEG
DSM111914_Staphylococcus sp. EMCR19	NEG	NEG	NEG	NEG	POS	POS	NEG	NEG	NEG	NEG	POS	NEG	NEG	NEG	NEG	NEG	NEG	NEG	NEG	NEG	NEG	NEG
**S. argenteus, CC1850**																						
MSHR1132: in silico predicted hybridisation pattern	AMB	AMB	NEG	NEG	NEG	NEG	NEG	NEG	NEG	NEG	POS	NEG	NEG	NEG	NEG	NEG	NEG	NEG	NEG	NEG	NEG	NEG
DSM28299	NEG	POS	NEG	NEG	NEG	NEG	NEG	NEG	NEG	NEG	POS	NEG	NEG	NEG	NEG	NEG	POS	NEG	NEG	NEG	NEG	NEG
**S. schweitzeri, CC2022**																						
NCTC13712: in silico predicted hybridisation pattern	AMB	NEG	NEG	NEG	NEG	NEG	NEG	NEG	POS	POS	POS	NEG	NEG	NEG	NEG	NEG	POS	POS	NEG	NEG	NEG	NEG
DSM28300	NEG	NEG	NEG	NEG	NEG	NEG	NEG	NEG	POS	POS	POS	NEG	NEG	NEG	NEG	NEG	POS	POS	NEG	NEG	NEG	NEG
**S. saureus, CC1**																						
MW2: in silico predicted hybridisation pattern	POS	POS	NEG	POS	POS	NEG	POS	POS	POS	POS	POS	NEG	NEG	POS	NEG	NEG	NEG	POS	AMB	POS	POS	NEG
MW2	POS	POS	NEG	POS	POS	NEG	POS	POS	POS	POS	POS	NEG	NEG	POS	NEG		NEG	POS	NEG	POS	POS	NEG

STRAIN / ISOLATE	VIRULENCE : STAPHYLOCOCCAL SUPERANTIGEN/ENTEROTOXIN-LIKE GENES (SET/SSL)																					
	ssl04		ssl05				ssl06		ssl07			ssl08	ssl09			ssl10			ssl11			
	ssl04/set9	ssl04/set9 (MRSA252, SAR0425)	ssl05/set3_probe 1	ssl05/set3 (RF122, probe-611)	ssl05/set3_probe 2 (612)	ssl05/set3 (MRSA252)	ssl06/set21	ssl06 (NCTC8325+ MW2)	ssl07/set1	ssl07/set1 (MRSA252)	ssl07/set1 (AF188836)		ssl09/set5_probe 1	ssl09/set5_probe 2	ssl09/set5 (MRSA252)	ssl10/set4	ssl10 (RF122)	ssl10/set4 (MRSA252)	ssl11/set2 (COL)	ssl11/set2 (Mu50+ N315)	ssl11/set2 (MW2+ MSSA476)	ssl11/set2 (MRSA252)
	Staphylococcal superantigen-like protein 4		Staphylococcal superantigen-like protein 5				Staphylococcal superantigen-like protein 6		Staphylococcal superantigen-like protein 7			Staphylococcal superantigen-like protein 8	Staphylococcal superantigen-like protein 9			Staphylococcal superantigen-like protein 10			Staphylococcal superantigene-like protein 11			
**CC3960/3961**																						
BDS-53E: in silico predicted hybridisation pattern	NEG	NEG	NEG	NEG	NEG	NEG	NEG	NEG	NEG	NEG	NEG	NEG	NEG	NEG	NEG	NEG	NEG	NEG	NEG	NEG	NEG	NEG
Bat isolate BDS-53E	NEG	NEG	NEG	NEG	NEG	NEG	NEG	NEG	NEG	NEG	NEG	NEG	NEG	NEG	NEG	NEG	NEG	NEG	NEG	NEG	NEG	NEG
Bat isolate BDS-53B	NEG	NEG	NEG	NEG	NEG	NEG	NEG	NEG	NEG	NEG	NEG	NEG	NEG	NEG	NEG	NEG	NEG	NEG	NEG	NEG	NEG	NEG
BDS-54: in silico predicted hybridisation pattern	NEG	NEG	NEG	NEG	NEG	NEG	NEG	NEG	NEG	NEG	NEG	NEG	NEG	NEG	NEG	NEG	NEG	NEG	NEG	NEG	NEG	NEG
Bat isolate BDS-54	NEG	NEG	NEG	NEG	NEG	NEG	NEG	NEG	NEG	NEG	NEG	NEG	NEG	NEG	NEG	NEG	NEG	NEG	NEG	NEG	NEG	NEG
Bat isolate BDH-128	NEG	NEG	NEG	NEG	NEG	NEG	NEG	NEG	NEG	NEG	NEG	NEG	NEG	NEG	NEG	POS	NEG	NEG	NEG	NEG	NEG	NEG
Bat isolate BDH-157	NEG	NEG	NEG	NEG	NEG	NEG	NEG	NEG	NEG	NEG	NEG	NEG	NEG	NEG	NEG	POS	NEG	NEG	NEG	NEG	NEG	NEG
Bat isolate BDS-69C	NEG	NEG	NEG	NEG	NEG	NEG	NEG	NEG	NEG	NEG	NEG	NEG	NEG	NEG	NEG	NEG	NEG	NEG	NEG	NEG	NEG	NEG
Bat isolate BDH-147	NEG	NEG	NEG	NEG	NEG	NEG	NEG	NEG	NEG	NEG	NEG	NEG	NEG	NEG	NEG	NEG	NEG	NEG	NEG	NEG	NEG	NEG
**CC7342**																						
Zoo-28: in silico predicted hybridisation pattern	NEG	NEG	NEG	NEG	NEG	NEG	AMB	POS	NEG	NEG	NEG	POS	AMB	AMB	NEG	POS	NEG	NEG	NEG	NEG	NEG	NEG
Finch isolate Zoo-28	NEG	POS	NEG	NEG	NEG	NEG	AMB	POS	NEG	NEG	NEG	POS	NEG	NEG	NEG	POS	NEG	NEG	NEG	NEG	POS	NEG
**"S. singaporensis", CC6105**																						
SS21: in silico predicted hybridisation pattern	NEG	NEG	NEG	POS	NEG	NEG	AMB	POS	NEG	NEG	NEG	POS	AMB	AMB	NEG	POS	NEG	NEG	NEG	NEG	NEG	NEG
DSM11148_Staphylococcus sp. SS21	NEG	AMB	NEG	NEG	NEG	NEG	NEG	AMB	NEG	NEG	NEG	NEG	NEG	AMB	NEG	POS	NEG	NEG	NEG	NEG	NEG	NEG
**"S. singaporensis", CC6106**																						
SS35: in silico predicted hybridisation pattern	NEG	NEG	NEG	POS	NEG	NEG	AMB	POS	NEG	NEG	NEG	POS	NEG	NEG	NEG	POS	NEG	NEG	NEG	NEG	NEG	NEG
**"S. singaporensis", CC6107**																						
SS60: in silico predicted hybridisation pattern	NEG	NEG	NEG	POS	NEG	NEG	NEG	NEG	NEG	NEG	NEG	POS	AMB	AMB	NEG	POS	NEG	NEG	NEG	NEG	NEG	NEG
**"S. singaporensis", CC6108**																						
SS87: in silico predicted hybridisation pattern	NEG	NEG	NEG	POS	NEG	NEG	NEG	NEG	NEG	NEG	NEG	POS	AMB	AMB	NEG	POS	NEG	NEG	NEG	NEG	NEG	NEG
**"S. singaporensis", CC6109**																						
SS90: in silico predicted hybridisation pattern	NEG	NEG	NEG	POS	NEG	NEG	NEG	NEG	NEG	NEG	NEG	POS	AMB	AMB	NEG	POS	NEG	NEG	NEG	NEG	NEG	POS
SS251: in silico predicted hybridisation pattern	NEG	NEG	NEG	POS	NEG	NEG	NEG	NEG	NEG	NEG	NEG	POS	AMB	AMB	NEG	POS	NEG	NEG	NEG	NEG	NEG	POS
**"S. roterodami", CC6999**																						
EMCR19: in silico predicted hybridisation pattern	NEG	NEG	NEG	POS	NEG	NEG	NEG	NEG	NEG	NEG	NEG	POS	AMB	AMB	NEG	NEG	NEG	POS	NEG	NEG	NEG	NEG
DSM111914_Staphylococcus sp. EMCR19	NEG	NEG	NEG	POS	AMB	NEG	NEG	NEG	NEG	NEG	NEG	POS	NEG	NEG	NEG	NEG	NEG	POS	NEG	NEG	NEG	NEG
**S. argenteus, CC1850**																						
MSHR1132: in silico predicted hybridisation pattern	NEG	POS	NEG	NEG	NEG	NEG	NEG	NEG	NEG	NEG	NEG	NEG	NEG	NEG	NEG	NEG	NEG	POS	NEG	NEG	NEG	NEG
DSM28299	NEG	POS	NEG	NEG	NEG	NEG	NEG	NEG	NEG	NEG	NEG	NEG	NEG	NEG	NEG	POS	NEG	AMB	NEG	NEG	NEG	NEG
**S. schweitzeri, CC2022**																						
NCTC13712: in silico predicted hybridisation pattern	NEG	NEG	NEG	NEG	NEG	AMB	POS	POS	NEG	NEG	NEG	POS	NEG	NEG	NEG	POS	NEG	NEG	NEG	NEG	NEG	NEG
DSM28300	NEG	AMB	NEG	NEG	NEG	POS	POS	POS	NEG	NEG	NEG	POS	NEG	NEG	NEG	POS	NEG	AMB	NEG	NEG	NEG	NEG
**S. saureus, CC1**																						
MW2: in silico predicted hybridisation pattern	POS	NEG	POS	NEG	POS	NEG	POS	POS	POS	NEG	NEG	POS	POS	POS	NEG	POS	NEG	NEG	NEG	NEG	POS	NEG
MW2	POS	NEG	POS	NEG	POS	NEG	POS	POS	POS	NEG	NEG	POS	POS	POS	NEG	POS	NEG	NEG	NEG	NEG	POS	NEG

STRAIN / ISOLATE	VIRULENCE : SET/SSL GENES					CAPSULE- AND BIOFILM-ASSOCIATED GENES																
	setB3		setB2			Capsule type 1				Capsule type 5				Capsule type 8								
	setB3	setB3 (MRSA252)	setB2	setB2 (MRSA252)	setB1	cap 1	capH1	capJ1	capK1	cap 5	capH5	capJ5	capK5	cap 8	capH8	capI8	capJ8	capK8	icaA	icaC	icaD	bap
	Staphylococcal exotoxin-like protein, second locus					Capsule type 1	capsular poly-saccharide synthesis enzyme	O-antigen polymerase	capsular poly-saccharide biosyn-thesis protein	Capsule type 5	capsular poly-saccharide synthesis enzyme	O-antigen polymerase	capsular poly-saccharide biosyn-thesis protein	Capsule type 8	capsular poly-saccharide synthesis enzyme	capsular poly-saccharide biosyn-thesis protein	O-antigen poly-merase	capsular poly-saccharide biosyn-thesis protein	intercellular adhesion protein A	intercellular adhesion protein C	biofilm PIA synthesis protein D	Surface protein involved in biofilm formation
**CC3960/3961**																						
BDS-53E: in silico predicted hybridisation pattern	NEG	NEG	NEG	NEG	POS	NEG	NEG	NEG	NEG	NEG	NEG	NEG	NEG	NEG	NEG	NEG	NEG	NEG	POS	NEG	NEG	NEG
Bat isolate BDS-53E	NEG	NEG	NEG	NEG	AMB	NEG	NEG	NEG	NEG	NEG	NEG	NEG	NEG	NEG	NEG	NEG	NEG	NEG	POS	NEG	NEG	NEG
Bat isolate BDS-53B	NEG	NEG	NEG	NEG	AMB	NEG	NEG	NEG	NEG	NEG	NEG	NEG	NEG	NEG	NEG	NEG	NEG	NEG	POS	NEG	NEG	NEG
BDS-54: in silico predicted hybridisation pattern	NEG	NEG	NEG	NEG	POS	NEG	NEG	NEG	NEG	NEG	NEG	NEG	NEG	NEG	NEG	NEG	NEG	NEG	POS	NEG	NEG	NEG
Bat isolate BDS-54	NEG	NEG	NEG	NEG	AMB	NEG	NEG	NEG	NEG	NEG	NEG	NEG	NEG	NEG	NEG	NEG	NEG	NEG	POS	NEG	NEG	NEG
Bat isolate BDH-128	NEG	NEG	NEG	NEG	NEG	NEG	NEG	NEG	NEG	NEG	NEG	NEG	NEG	NEG	NEG	NEG	NEG	NEG	NEG	NEG	NEG	NEG
Bat isolate BDH-157	NEG	NEG	NEG	NEG	POS	NEG	NEG	NEG	NEG	NEG	NEG	NEG	NEG	NEG	NEG	NEG	NEG	NEG	POS	NEG	NEG	NEG
Bat isolate BDS-69C	NEG	NEG	NEG	NEG	NEG	NEG	NEG	NEG	NEG	NEG	NEG	NEG	NEG	NEG	NEG	NEG	NEG	NEG	POS	NEG	NEG	NEG
Bat isolate BDH-147	NEG	NEG	NEG	NEG	POS	NEG	NEG	NEG	NEG	NEG	NEG	NEG	NEG	NEG	NEG	NEG	NEG	NEG	POS	NEG	NEG	NEG
**CC7342**																						
Zoo-28: in silico predicted hybridisation pattern	NEG	NEG	NEG	NEG	POS	NEG	NEG	NEG	NEG	NEG	NEG	NEG	NEG	NEG	NEG	NEG	NEG	NEG	AMB	NEG	NEG	NEG
Finch isolate Zoo-28	NEG	NEG	NEG	NEG	POS	NEG	NEG	NEG	NEG	NEG	NEG	NEG	NEG	NEG	NEG	NEG	NEG	NEG	NEG	NEG	NEG	NEG
**"S. singaporensis", CC6105**																						
SS21: in silico predicted hybridisation pattern	NEG	NEG	NEG	NEG	POS	NEG	NEG	NEG	NEG	NEG	NEG	NEG	NEG	NEG	NEG	NEG	NEG	NEG	POS	NEG	NEG	NEG
DSM11148_Staphylococcus sp. SS21	NEG	NEG	NEG	NEG	NEG	NEG	NEG	NEG	NEG	NEG	NEG	NEG	NEG	NEG	NEG	NEG	NEG	NEG	NEG	NEG	NEG	NEG
**"S. singaporensis", CC6106**																						
SS35: in silico predicted hybridisation pattern	NEG	NEG	NEG	NEG	POS	NEG	NEG	NEG	NEG	NEG	NEG	NEG	NEG	NEG	NEG	NEG	NEG	NEG	POS	NEG	NEG	NEG
**"S. singaporensis", CC6107**																						
SS60: in silico predicted hybridisation pattern	NEG	NEG	NEG	NEG	POS	NEG	NEG	NEG	NEG	NEG	NEG	NEG	NEG	NEG	NEG	NEG	NEG	NEG	POS	NEG	NEG	NEG
**"S. singaporensis", CC6108**																						
SS87: in silico predicted hybridisation pattern	NEG	NEG	NEG	NEG	POS	NEG	NEG	NEG	NEG	NEG	NEG	NEG	NEG	NEG	NEG	NEG	NEG	NEG	POS	NEG	NEG	NEG
**"S. singaporensis", CC6109**																						
SS90: in silico predicted hybridisation pattern	NEG	NEG	NEG	NEG	POS	NEG	NEG	NEG	NEG	NEG	NEG	NEG	NEG	NEG	NEG	NEG	NEG	NEG	POS	NEG	NEG	NEG
SS251: in silico predicted hybridisation pattern	NEG	NEG	NEG	NEG	POS	NEG	NEG	NEG	NEG	NEG	NEG	NEG	NEG	NEG	NEG	NEG	NEG	NEG	POS	NEG	NEG	NEG
**"S. roterodami", CC6999**																						
EMCR19: in silico predicted hybridisation pattern	NEG	NEG	NEG	NEG	POS	NEG	NEG	NEG	NEG	NEG	NEG	NEG	NEG	NEG	NEG	NEG	NEG	NEG	POS	NEG	NEG	NEG
DSM111914_Staphylococcus sp. EMCR19	NEG	NEG	NEG	NEG	NEG	NEG	NEG	NEG	NEG	NEG	NEG	NEG	NEG	NEG	NEG	NEG	NEG	NEG	POS	NEG	NEG	NEG
**S. argenteus, CC1850**																						
MSHR1132: in silico predicted hybridisation pattern	NEG	NEG	NEG	NEG	NEG	NEG	NEG	NEG	NEG	NEG	NEG	NEG	NEG	NEG	NEG	NEG	NEG	NEG	POS	NEG	NEG	NEG
DSM28299	NEG	NEG	NEG	NEG	NEG	NEG	NEG	NEG	NEG	NEG	NEG	NEG	NEG	NEG	NEG	NEG	NEG	NEG	POS	NEG	NEG	NEG
**S. schweitzeri, CC2022**																						
NCTC13712: in silico predicted hybridisation pattern	POS	NEG	POS	NEG	POS	NEG	NEG	NEG	NEG	AMB	NEG	AMB	POS	NEG	NEG	NEG	NEG	NEG	POS	NEG	NEG	NEG
DSM28300	POS	NEG	POS	NEG	POS	NEG	NEG	NEG	NEG	POS	POS	POS	POS	NEG	NEG	NEG	NEG	NEG	POS	NEG	NEG	NEG
**S. saureus, CC1**																						
MW2: in silico predicted hybridisation pattern	POS	NEG	POS	NEG	POS	NEG	NEG	NEG	NEG	NEG	NEG	NEG	NEG	POS	POS	POS	POS	POS	POS	POS	POS	NEG
MW2	POS	NEG	POS	NEG	POS	NEG	NEG	NEG	NEG	NEG	NEG	NEG	NEG	POS	POS	POS	POS	POS	POS	POS	POS	NEG

STRAIN / ISOLATE	ADHAESION FACTORS / GENES ENCODING MICROBIAL SURFACE COMPONENTS RECOGNIZING ADHESIVE MATRIX MOLECULES (MSCRAMM GENES)																	
	bbp							clfA					clfB					cna
	bbp	bbp (cons)	bbp (COL+MW2)	bbp (MRSA252)	bbp (Mu50)	bbp (RF122)	bbp (ST45)	clfA	clfA (cons)	clfA (COL+RF122)	clfA (MRSA252)	clfA (Mu50+MW2)	clfB	clfB (cons)	clfB (COL+Mu50)	clfB (MW2)	clfB (RF122)	
	Bone sialoprotein-binding protein							Clumping factor A					Clumping factor B					Collagen-binding adhesin
**CC3960/3961**																		
BDS-53E: in silico predicted hybridisation pattern	NEG	NEG	NEG	NEG	NEG	NEG	NEG	POS	NEG	NEG	NEG	POS	POS	NEG	POS	NEG	NEG	NEG
Bat isolate BDS-53E	NEG	NEG	NEG	NEG	NEG	NEG	NEG	NEG	NEG	NEG	NEG	NEG	POS	NEG	POS	NEG	NEG	NEG
Bat isolate BDS-53B	NEG	NEG	NEG	NEG	NEG	NEG	NEG	NEG	NEG	NEG	NEG	NEG	POS	NEG	POS	NEG	NEG	NEG
BDS-54: in silico predicted hybridisation pattern	NEG	NEG	NEG	NEG	NEG	NEG	NEG	POS	NEG	NEG	NEG	POS	POS	NEG	POS	NEG	NEG	NEG
Bat isolate BDS-54	NEG	NEG	NEG	NEG	NEG	NEG	NEG	NEG	NEG	NEG	NEG	NEG	POS	NEG	POS	NEG	NEG	NEG
Bat isolate BDH-128	NEG	NEG	NEG	NEG	NEG	NEG	NEG	NEG	NEG	NEG	NEG	NEG	POS	NEG	POS	NEG	NEG	NEG
Bat isolate BDH-157	NEG	NEG	NEG	NEG	NEG	NEG	NEG	NEG	NEG	NEG	NEG	NEG	POS	NEG	POS	NEG	NEG	NEG
Bat isolate BDS-69C	NEG	NEG	NEG	NEG	NEG	NEG	NEG	NEG	NEG	NEG	NEG	NEG	POS	NEG	POS	NEG	NEG	NEG
Bat isolate BDH-147	NEG	NEG	NEG	NEG	NEG	NEG	NEG	NEG	NEG	NEG	NEG	NEG	POS	NEG	POS	NEG	NEG	NEG
**CC7342**																		
Zoo-28: in silico predicted hybridisation pattern	NEG	NEG	NEG	NEG	NEG	NEG	NEG	POS	NEG	NEG	POS	NEG	POS	NEG	POS	NEG	NEG	NEG
Finch isolate Zoo-28	NEG	NEG	NEG	NEG	NEG	NEG	NEG	POS	NEG	NEG	POS	NEG	POS	AMB	POS	NEG	NEG	NEG
**"S. singaporensis", CC6105**																		
SS21: in silico predicted hybridisation pattern	POS	AMB	NEG	NEG	POS	NEG	NEG	NEG	NEG	NEG	NEG	NEG	POS	NEG	POS	NEG	NEG	NEG
DSM11148_Staphylococcus sp. SS21	POS	POS	NEG	NEG	NEG	NEG	NEG	NEG	NEG	NEG	NEG	NEG	POS	NEG	POS	NEG	NEG	NEG
**"S. singaporensis", CC6106**																		
SS35: in silico predicted hybridisation pattern	POS	POS	NEG	NEG	POS	NEG	NEG	POS	NEG	NEG	NEG	POS	POS	NEG	POS	NEG	NEG	NEG
**"S. singaporensis", CC6107**																		
SS60: in silico predicted hybridisation pattern	POS	POS	NEG	NEG	POS	NEG	NEG	POS	NEG	NEG	NEG	POS	POS	NEG	POS	NEG	NEG	NEG
**"S. singaporensis", CC6108**																		
SS87: in silico predicted hybridisation pattern	POS	POS	NEG	NEG	POS	NEG	NEG	NEG	NEG	NEG	NEG	NEG	POS	NEG	POS	NEG	NEG	NEG
**"S. singaporensis", CC6109**																		
SS90: in silico predicted hybridisation pattern	POS	AMB	NEG	POS	NEG	NEG	NEG	NEG	NEG	NEG	NEG	NEG	POS	NEG	POS	NEG	NEG	NEG
SS251: in silico predicted hybridisation pattern	POS	AMB	NEG	POS	NEG	NEG	NEG	NEG	NEG	NEG	NEG	NEG	POS	NEG	POS	NEG	NEG	NEG
**"S. roterodami", CC6999**																		
EMCR19: in silico predicted hybridisation pattern	POS	AMB	NEG	POS	NEG	NEG	NEG	NEG	NEG	NEG	NEG	NEG	POS	NEG	POS	NEG	NEG	NEG
DSM111914_Staphylococcus sp. EMCR19	POS	AMB	NEG	POS	NEG	NEG	NEG	POS	NEG	NEG	POS	NEG	POS	NEG	POS	NEG	NEG	NEG
**S. argenteus, CC1850**																		
MSHR1132: in silico predicted hybridisation pattern	POS	POS	NEG	POS	NEG	NEG	NEG	NEG	NEG	NEG	NEG	NEG	POS	NEG	POS	NEG	NEG	NEG
DSM28299	POS	POS	NEG	POS	NEG	NEG	NEG	POS	NEG	NEG	POS	NEG	POS	POS	POS	NEG	NEG	NEG
**S. schweitzeri, CC2022**																		
NCTC13712: in silico predicted hybridisation pattern	NEG	AMB	NEG	NEG	NEG	NEG	NEG	POS	POS	NEG	NEG	NEG	POS	POS	POS	NEG	NEG	NEG
DSM28300	POS	POS	NEG	NEG	NEG	NEG	NEG	POS	POS	NEG	NEG	NEG	POS	AMB	POS	NEG	NEG	NEG
**S. saureus, CC1**																		
MW2: in silico predicted hybridisation pattern	POS	POS	POS	NEG	NEG	NEG	NEG	POS	POS	NEG	NEG	POS	POS	POS	NEG	POS	AMB	POS
MW2	POS	POS	POS	NEG	NEG	NEG	NEG	POS	POS	NEG	NEG	POS	POS	POS	NEG	POS	POS	POS

STRAIN / ISOLATE	ADHAESION FACTORS / GENES ENCODING MICROBIAL SURFACE COMPONENTS RECOGNIZING ADHESIVE MATRIX MOLECULES (MSCRAMM GENES)														
	ebh	ebpS						efb		fnbA					
	ebh (cons)	ebpS	ebpS_probe 612	ebpS_probe 614	ebpS (01-1111)	ebpS (COL)	eno	efb / fib	efb / fib (MRSA252)	fnbA	fnbA (cons)	fnbA (COL)	fnbA (MRSA252)	fnbA (Mu50+MW2)	fnbA (RF122)
	Cell wall associated fibronectin-binding protein	cell surface elastin binding protein					enolase	fibrinogen binding protein (19 kDa)		fibronectin-binding protein A					
**CC3960/3961**															
BDS-53E: in silico predicted hybridisation pattern	AMB	NEG	NEG	NEG	NEG	NEG	POS	POS	NEG	POS	POS	NEG	NEG	NEG	NEG
Bat isolate BDS-53E	NEG	NEG	NEG	NEG	NEG	NEG	POS	POS	NEG	POS	POS	NEG	NEG	NEG	NEG
Bat isolate BDS-53B	NEG	NEG	NEG	NEG	NEG	NEG	POS	POS	NEG	POS	POS	NEG	NEG	NEG	NEG
BDS-54: in silico predicted hybridisation pattern	NEG	NEG	NEG	NEG	NEG	NEG	POS	POS	NEG	POS	POS	NEG	NEG	NEG	NEG
Bat isolate BDS-54	NEG	NEG	NEG	NEG	NEG	NEG	POS	POS	NEG	POS	POS	NEG	NEG	NEG	NEG
Bat isolate BDH-128	NEG	NEG	NEG	NEG	NEG	NEG	POS	POS	NEG	POS	POS	NEG	NEG	NEG	NEG
Bat isolate BDH-157	NEG	NEG	NEG	NEG	NEG	NEG	POS	POS	NEG	POS	POS	NEG	NEG	NEG	NEG
Bat isolate BDS-69C	NEG	NEG	NEG	NEG	NEG	NEG	POS	POS	NEG	POS	POS	NEG	NEG	NEG	NEG
Bat isolate BDH-147	AMB	NEG	NEG	NEG	NEG	NEG	POS	POS	NEG	POS	POS	NEG	NEG	NEG	NEG
**CC7342**															
Zoo-28: in silico predicted hybridisation pattern	AMB	NEG	NEG	NEG	NEG	NEG	POS	AMB	NEG	POS	AMB	NEG	POS	NEG	NEG
Finch isolate Zoo-28	NEG	NEG	NEG	NEG	NEG	NEG	POS	AMB	NEG	POS	AMB	NEG	POS	NEG	NEG
**"S. singaporensis", CC6105**															
SS21: in silico predicted hybridisation pattern	AMB	NEG	NEG	NEG	NEG	NEG	POS	POS	NEG	POS	POS	NEG	NEG	NEG	NEG
DSM11148_Staphylococcus sp. SS21	NEG	NEG	NEG	NEG	NEG	NEG	POS	AMB	NEG	POS	POS	NEG	NEG	NEG	NEG
**"S. singaporensis", CC6106**															
SS35: in silico predicted hybridisation pattern	AMB	NEG	NEG	NEG	NEG	NEG	POS	POS	NEG	POS	POS	NEG	POS	NEG	NEG
**"S. singaporensis", CC6107**															
SS60: in silico predicted hybridisation pattern	AMB	NEG	NEG	NEG	NEG	NEG	POS	POS	NEG	POS	POS	NEG	NEG	NEG	NEG
**"S. singaporensis", CC6108**															
SS87: in silico predicted hybridisation pattern	AMB	NEG	NEG	NEG	NEG	NEG	POS	POS	NEG	POS	POS	NEG	NEG	NEG	POS
**"S. singaporensis", CC6109**															
SS90: in silico predicted hybridisation pattern	AMB	NEG	NEG	NEG	NEG	NEG	POS	AMB	NEG	POS	POS	NEG	NEG	NEG	NEG
SS251: in silico predicted hybridisation pattern	AMB	NEG	NEG	NEG	NEG	NEG	POS	AMB	NEG	POS	POS	NEG	NEG	NEG	NEG
**"S. roterodami", CC6999**															
EMCR19: in silico predicted hybridisation pattern	AMB	NEG	NEG	NEG	NEG	NEG	POS	POS	NEG	POS	POS	NEG	NEG	NEG	NEG
DSM111914_Staphylococcus sp. EMCR19	NEG	NEG	NEG	NEG	NEG	NEG	POS	POS	NEG	POS	POS	NEG	NEG	NEG	POS
**S. argenteus, CC1850**															
MSHR1132: in silico predicted hybridisation pattern	NEG	NEG	NEG	NEG	NEG	NEG	POS	AMB	AMB	POS	POS	NEG	NEG	NEG	NEG
DSM28299	NEG	NEG	NEG	NEG	NEG	NEG	POS	NEG	NEG	POS	POS	NEG	NEG	NEG	POS
**S. schweitzeri, CC2022**															
NCTC13712: in silico predicted hybridisation pattern	NEG	NEG	NEG	NEG	NEG	NEG	AMB	AMB	NEG	POS	POS	NEG	NEG	POS	NEG
DSM28300	NEG	NEG	NEG	NEG	NEG	NEG	POS	NEG	NEG	POS	POS	NEG	NEG	POS	NEG
**S. saureus, CC1**															
MW2: in silico predicted hybridisation pattern	POS	POS	POS	POS	NEG	NEG	POS	POS	NEG	POS	POS	NEG	NEG	POS	NEG
MW2	POS	POS	POS	POS	NEG	NEG	POS	POS	NEG	POS	POS	NEG	NEG	POS	NEG

STRAIN / ISOLATE	ADHAESION FACTORS / GENES ENCODING MICROBIAL SURFACE COMPONENTS RECOGNIZING ADHESIVE MATRIX MOLECULES (MSCRAMM GENES)																					
	fnbB							map				sasG					sdrC					
	fnbB	fnbB (COL)	fnbB (COL+Mu50+ MW2)	fnbB (Mu50)	fnbB (MW2)	fnbB (ST15)	fnbB (ST45-2)	map	map (COL)	map (MRSA252)	map (Mu50+MW2)	sasG	sasG (COL+Mu50)	sasG (MW2)	sasG (OtherThan25 2+122	sasX / sesI	sdrC	sdrC (B1)	sdrC (COL)	sdrC (Mu50)	sdrC (MW2+MRSA 252+RF122)	sdrC (OtherThan25 2+RF122)
	Fibronectin-binding protein B							Major histocompatibility complex class II analog protein (=Extracellular adherence protein, eap)				Staphylococcus aureus surface protein G					Ser-Asp rich fibrinogen-/bone sialoprotein-binding protein C					
**CC3960/3961**																						
BDS-53E: in silico predicted hybridisation pattern	POS	NEG	AMB	AMB	NEG	NEG	NEG	NEG	NEG	NEG	NEG	POS	NEG	POS	POS	NEG	POS	NEG	NEG	AMB	NEG	NEG
Bat isolate BDS-53E	POS	NEG	POS	AMB	NEG	NEG	NEG	NEG	NEG	NEG	NEG	POS	NEG	POS	POS	NEG	POS	NEG	NEG	POS	NEG	NEG
Bat isolate BDS-53B	POS	NEG	POS	AMB	NEG	NEG	NEG	NEG	NEG	NEG	NEG	POS	NEG	POS	POS	NEG	POS	NEG	NEG	POS	NEG	NEG
BDS-54: in silico predicted hybridisation pattern	POS	NEG	AMB	AMB	NEG	NEG	NEG	NEG	NEG	NEG	NEG	POS	NEG	POS	POS	NEG	NEG	NEG	NEG	NEG	NEG	NEG
Bat isolate BDS-54	POS	NEG	POS	AMB	NEG	NEG	NEG	NEG	NEG	NEG	NEG	POS	NEG	POS	POS	NEG	NEG	NEG	NEG	NEG	NEG	NEG
Bat isolate BDH-128	POS	NEG	POS	AMB	NEG	NEG	NEG	NEG	NEG	NEG	NEG	POS	NEG	POS	POS		NEG	NEG	NEG	NEG	NEG	NEG
Bat isolate BDH-157	POS	NEG	POS	AMB	NEG	AMB	NEG	NEG	NEG	NEG	NEG	POS	NEG	POS	POS	NEG	NEG	NEG	NEG	NEG	NEG	NEG
Bat isolate BDS-69C	POS	NEG	POS	AMB	NEG	NEG	NEG	NEG	NEG	NEG	NEG	POS	NEG	POS	POS	NEG	POS	NEG	NEG	POS	NEG	NEG
Bat isolate BDH-147	POS	NEG	POS	AMB	NEG	AMB	NEG	NEG	NEG	NEG	NEG	POS	NEG	POS	POS	NEG	POS	NEG	NEG	POS	AMB	NEG
**CC7342**																						
Zoo-28: in silico predicted hybridisation pattern	POS	NEG	AMB	POS	NEG	NEG	NEG	NEG	NEG	NEG	NEG	NEG	NEG	NEG	NEG	NEG	POS	NEG	NEG	AMB	NEG	NEG
Finch isolate Zoo-28	POS	NEG	POS	AMB	NEG	NEG	NEG	NEG	NEG	NEG	NEG	NEG	NEG	NEG	NEG	NEG	POS	NEG	NEG	POS	AMB	NEG
**"S. singaporensis", CC6105**																						
SS21: in silico predicted hybridisation pattern	POS	NEG	AMB	NEG	NEG	NEG	AMB	NEG	NEG	NEG	NEG	POS	AMB	NEG	POS	NEG	POS	NEG	NEG	AMB	NEG	NEG
DSM11148_Staphylococcus sp. SS21	POS	NEG	POS	NEG	NEG	NEG	AMB	NEG	NEG	NEG	NEG	POS	POS	NEG	POS	NEG	POS	NEG	NEG	POS	NEG	NEG
**"S. singaporensis", CC6106**																						
SS35: in silico predicted hybridisation pattern	POS	NEG	AMB	NEG	AMB	NEG	NEG	NEG	NEG	NEG	NEG	NEG	NEG	NEG	NEG	NEG	POS	NEG	NEG	AMB	NEG	NEG
**"S. singaporensis", CC6107**																						
SS60: in silico predicted hybridisation pattern	POS	NEG	AMB	NEG	NEG	NEG	AMB	NEG	NEG	NEG	NEG	POS	AMB	NEG	POS	NEG	POS	NEG	POS	NEG	NEG	NEG
**"S. singaporensis", CC6108**																						
SS87: in silico predicted hybridisation pattern	POS	NEG	AMB	AMB	NEG	NEG	NEG	NEG	AMB	NEG	NEG	NEG	NEG	NEG	NEG	NEG	POS	NEG	NEG	AMB	NEG	NEG
**"S. singaporensis", CC6109**																						
SS90: in silico predicted hybridisation pattern	POS	NEG	AMB	AMB	NEG	NEG	NEG	NEG	NEG	NEG	NEG	NEG	NEG	NEG	NEG	NEG	POS	NEG	NEG	AMB	NEG	NEG
SS251: in silico predicted hybridisation pattern	POS	NEG	AMB	AMB	NEG	NEG	NEG	NEG	NEG	NEG	NEG	NEG	NEG	NEG	NEG	NEG	POS	NEG	NEG	AMB	NEG	NEG
**"S. roterodami", CC6999**																						
EMCR19: in silico predicted hybridisation pattern	POS	NEG	AMB	POS	NEG	NEG	NEG	NEG	AMB	NEG	NEG	NEG	NEG	NEG	NEG	NEG	POS	NEG	POS	NEG	NEG	NEG
DSM111914_Staphylococcus sp. EMCR19	POS	NEG	POS	AMB	NEG	NEG	NEG	NEG	NEG	NEG	NEG	NEG	NEG	NEG	NEG	NEG	POS	NEG	POS	NEG	NEG	NEG
**S. argenteus, CC1850**																						
MSHR1132: in silico predicted hybridisation pattern	POS	NEG	NEG	AMB	NEG	AMB	NEG	NEG	NEG	NEG	NEG	POS	NEG	POS	NEG	NEG	POS	NEG	NEG	NEG	NEG	NEG
DSM28299	POS	NEG	NEG	POS	NEG	AMB	NEG	NEG	NEG	NEG	NEG	POS	NEG	POS	NEG	NEG	POS	NEG	NEG	NEG	NEG	NEG
**S. schweitzeri, CC2022**																						
NCTC13712: in silico predicted hybridisation pattern	POS	NEG	POS	NEG	AMB	NEG	NEG	NEG	NEG	NEG	NEG	POS	NEG	POS	POS	NEG	NEG	NEG	NEG	NEG	NEG	NEG
DSM28300	POS	NEG	POS	NEG	AMB	NEG	NEG	NEG	NEG	NEG	NEG	POS	NEG	POS	POS		NEG	NEG	NEG	NEG	NEG	NEG
**S. saureus, CC1**																						
MW2: in silico predicted hybridisation pattern	POS	NEG	AMB	NEG	AMB	NEG	NEG	POS	AMB	NEG	POS	POS	NEG	POS	POS	NEG	POS	NEG	NEG	NEG	POS	POS
MW2	POS	NEG	POS	NEG	POS	NEG	NEG	NEG	AMB	NEG	AMB	POS	NEG	POS	POS	NEG	POS	NEG	NEG	NEG	POS	POS

STRAIN / ISOLATE	ADHAESION FACTORS / MSCRAMM GENES											IMMUNOD.AG.B		DEFENSIN RESIST.		TRANSFERRIN BINDING PROT			PUTATIVE TRANSPORTER			
	sdrD					vwb						isaB		mprF		isdA			lmrP			
	sdrD	sdrD (cons)	sdrD (COL+MW2)	sdrD (Mu50)	sdrD (other)	vwb	vwb (cons)	vwb (COL+MW2)	vwb (MRSA252)	vwb (Mu50)	vwb (RF122)	isaB	isaB (MRSA252)	mprF (COL+MW2)	mprF (Mu50+252)	isdA (cons)	isdA (MRSA252)	isdA (Other Than MRSA252)	lmrP (OtherThanRF 122)	lmrP (OtherThanRF 122)	lmrP (RF122)	lmrP (RF122)
	Ser-Asp rich fibrinogen-/bone sialoprotein-binding protein D					van Willebrand factor binding protein						immunodominant antigen B		defensin resistance protein		transferrin-binding protein			hypothetical protein, similar to integral membrane protein LmrP			
**CC3960/3961**																						
BDS-53E: in silico predicted hybridisation pattern	NEG	NEG	NEG	NEG	NEG	NEG	AMB	NEG	NEG	NEG	NEG	NEG	POS	POS	AMB	NEG	NEG	NEG	NEG	AMB	NEG	NEG
Bat isolate BDS-53E	NEG	NEG	NEG	NEG	NEG	POS	POS	NEG	NEG	NEG	NEG	NEG	POS	POS	AMB	NEG	NEG	NEG	NEG	POS	NEG	NEG
Bat isolate BDS-53B	NEG	NEG	NEG	NEG	NEG	POS	POS	NEG	NEG	NEG	NEG	NEG	POS	POS	AMB	NEG	NEG	NEG	NEG	POS	NEG	NEG
BDS-54: in silico predicted hybridisation pattern	POS	AMB	NEG	NEG	POS	NEG	AMB	NEG	NEG	NEG	NEG	NEG	POS	POS	AMB	NEG	NEG	NEG	NEG	AMB	NEG	NEG
Bat isolate BDS-54	POS	POS	NEG	NEG	POS	POS	POS	NEG	NEG	NEG	NEG	NEG	POS	POS	AMB	NEG	NEG	NEG	NEG	POS	NEG	NEG
Bat isolate BDH-128	POS	NEG	NEG	NEG	POS	NEG	NEG	NEG	NEG	NEG	NEG	NEG	NEG	POS	NEG	NEG	NEG	NEG	NEG	NEG	NEG	NEG
Bat isolate BDH-157	POS	POS	NEG	NEG	POS	POS	POS	NEG	NEG	NEG	NEG	NEG	POS	POS	AMB	NEG	NEG	NEG	NEG	POS	NEG	NEG
Bat isolate BDS-69C	NEG	NEG	NEG	NEG	NEG	POS	POS	NEG	NEG	NEG	NEG	NEG	NEG	POS	NEG	NEG	NEG	NEG	NEG	AMB	NEG	NEG
Bat isolate BDH-147	NEG	NEG	NEG	NEG	NEG	POS	POS	NEG	NEG	NEG	NEG	NEG	POS	POS	AMB	NEG	NEG	NEG	NEG	POS	NEG	NEG
**CC7342**																						
Zoo-28: in silico predicted hybridisation pattern	NEG	NEG	NEG	NEG	NEG	NEG	NEG	NEG	NEG	NEG	NEG	NEG	NEG	POS	AMB	NEG	NEG	NEG	NEG	AMB	NEG	NEG
Finch isolate Zoo-28	NEG	NEG	NEG	NEG	NEG	AMB	AMB	NEG	NEG	NEG	NEG	NEG	NEG	NEG	NEG	NEG	NEG	NEG	NEG	AMB	NEG	NEG
**"S. singaporensis", CC6105**																						
SS21: in silico predicted hybridisation pattern	POS	AMB	NEG	NEG	POS	NEG	AMB	NEG	NEG	NEG	NEG	NEG	POS	POS	AMB	NEG	NEG	NEG	NEG	AMB	NEG	NEG
DSM11148_Staphylococcus sp. SS21	POS	NEG	NEG	NEG	POS	NEG	NEG	NEG	NEG	NEG	NEG	NEG	NEG	NEG	NEG	NEG	NEG	NEG	NEG	NEG	NEG	NEG
**"S. singaporensis", CC6106**																						
SS35: in silico predicted hybridisation pattern	NEG	NEG	NEG	NEG	NEG	NEG	AMB	NEG	NEG	NEG	NEG	NEG	POS	POS	AMB	NEG	NEG	NEG	NEG	AMB	NEG	NEG
**"S. singaporensis", CC6107**																						
SS60: in silico predicted hybridisation pattern	POS	AMB	NEG	NEG	POS	NEG	AMB	NEG	NEG	NEG	NEG	NEG	NEG	POS	AMB	NEG	NEG	NEG	NEG	AMB	NEG	NEG
**"S. singaporensis", CC6108**																						
SS87: in silico predicted hybridisation pattern	NEG	NEG	NEG	NEG	NEG	NEG	AMB	NEG	NEG	NEG	NEG	NEG	POS	POS	AMB	NEG	NEG	NEG	NEG	AMB	NEG	NEG
**"S. singaporensis", CC6109**																						
SS90: in silico predicted hybridisation pattern	NEG	AMB	NEG	AMB	NEG	NEG	AMB	NEG	NEG	NEG	NEG	NEG	POS	POS	AMB	NEG	NEG	NEG	NEG	AMB	NEG	NEG
SS251: in silico predicted hybridisation pattern	NEG	AMB	NEG	AMB	NEG	NEG	AMB	NEG	NEG	NEG	NEG	NEG	POS	POS	AMB	NEG	NEG	NEG	NEG	AMB	NEG	NEG
**"S. roterodami", CC6999**																						
EMCR19: in silico predicted hybridisation pattern	NEG	AMB	NEG	NEG	NEG	NEG	NEG	NEG	NEG	NEG	NEG	NEG	POS	POS	AMB	NEG	NEG	NEG	NEG	AMB	NEG	NEG
DSM111914_Staphylococcus sp. EMCR19	NEG	AMB	NEG	NEG	NEG	NEG	NEG	NEG	NEG	NEG	NEG	NEG	NEG	POS	NEG	NEG	NEG	NEG	NEG	NEG	NEG	NEG
**S. argenteus, CC1850**																						
MSHR1132: in silico predicted hybridisation pattern	POS	AMB	NEG	NEG	POS	NEG	NEG	NEG	NEG	NEG	NEG	AMB	AMB	POS	AMB	NEG	NEG	NEG	NEG	AMB	NEG	NEG
DSM28299	POS	AMB	NEG	NEG	POS	NEG	NEG	NEG	NEG	NEG	NEG	AMB	POS	POS	AMB	NEG	NEG	NEG	NEG	POS	NEG	NEG
**S. schweitzeri, CC2022**																						
NCTC13712: in silico predicted hybridisation pattern	NEG	NEG	NEG	NEG	NEG	POS	POS	NEG	NEG	NEG	NEG	NEG	POS	POS	AMB	NEG	NEG	NEG	NEG	NEG	NEG	NEG
DSM28300	NEG	NEG	NEG	NEG	NEG	POS	POS	NEG	NEG	NEG	NEG	NEG	POS	POS	AMB	NEG	NEG	NEG	NEG	NEG	NEG	NEG
**S. saureus, CC1**																						
MW2: in silico predicted hybridisation pattern	POS	POS	POS	NEG	NEG	POS	POS	POS	NEG	NEG	NEG	POS	NEG	POS	NEG	POS	NEG	POS	POS	POS	NEG	NEG
MW2	POS	POS	POS	NEG	NEG	POS	POS	POS	NEG	NEG	NEG	POS	NEG	POS	NEG	POS	NEG	POS	POS	POS	NEG	NEG

STRAIN / ISOLATE	hsdS1-RF122	hsdS2-ST5+ST8	hsdS2-MW2+476	hsdS2-RF122	hsdS2-MRSA252	hsdS3-AllOtherThan RF122+252	hsdS3-ST8+ST1+RF122	hsdS3-Mu50+N315	hsdS3-CC51+252	hsdS3-MRSA252	hsdSx-CC25	hsdSx-CC15	hsdSx-etd	ear2 = Q2FXC0	Q2YUB3	Q7A4X2	Q931R4 (CC5, CC15, CC30, CC97, CC188, ST1850)	Q9RL82 (consensus)	Q9RL82 (CC8)	Q9RL82-CC10/361	Q2G1R6-genomic island / cstB
	TYPE I RESTRICTION-MODIFICATION SYSTEM, SINGLE SEQUENCE SPECIFICITY PROTEIN													MISCELLANEOUS GENES							
	hsdS1	hsdS2				hsdS3					hsdSx							Q9RL82			
	type I site-specific deoxyribo-nuclease subunit, 1st locus	type I site-specific deoxyribonuclease subunit, 2nd locus				type I site-specific deoxyribonuclease subunit, 3rd locus					type I site-specific deoxyribonuclease subunit, unknown locus			Putative protein	Multidrug resistance transporter	Putative protein	major facilitator superfamily transporter	Putative protein			
**CC3960/3961**																					
BDS-53E: in silico predicted hybridisation pattern	NEG	NEG	NEG	NEG	NEG	NEG	NEG	NEG	NEG	NEG	NEG	NEG	NEG	NEG	NEG	NEG	AMB	POS	AMB	POS	NEG
Bat isolate BDS-53E	NEG	NEG	NEG	NEG	NEG	NEG	NEG	NEG	NEG	NEG	NEG	NEG	NEG	NEG	NEG	NEG	POS	POS	NEG	AMB	NEG
Bat isolate BDS-53B	NEG	NEG	NEG	NEG	NEG	NEG	NEG	NEG	NEG	NEG	NEG	NEG	NEG	NEG	NEG	NEG	AMB	POS	NEG	AMB	NEG
BDS-54: in silico predicted hybridisation pattern	NEG	NEG	NEG	NEG	NEG	NEG	NEG	NEG	NEG	NEG	NEG	NEG	NEG	NEG	NEG	NEG	AMB	POS	AMB	POS	NEG
Bat isolate BDS-54	NEG	NEG	NEG	NEG	NEG	NEG	NEG	NEG	NEG	NEG	NEG	NEG	NEG	NEG	NEG	NEG	AMB	AMB	NEG	NEG	NEG
Bat isolate BDH-128	NEG	NEG	NEG	NEG	NEG	NEG	NEG	NEG	NEG	NEG	NEG	NEG	NEG	NEG	NEG	NEG					
Bat isolate BDH-157	NEG	NEG	NEG	NEG	NEG	NEG	NEG	NEG	NEG	NEG	NEG	NEG	NEG	NEG	NEG	NEG	POS	POS	NEG	POS	NEG
Bat isolate BDS-69C	NEG	NEG	NEG	NEG	NEG	NEG	NEG	NEG	NEG	NEG	NEG	NEG	NEG	NEG	NEG	NEG	AMB	POS	NEG	NEG	NEG
Bat isolate BDH-147	NEG	NEG	NEG	NEG	NEG	NEG	NEG	NEG	NEG	NEG	NEG	NEG	NEG	NEG	NEG	NEG	AMB	POS	NEG	POS	NEG
**CC7342**																					
Zoo-28: in silico predicted hybridisation pattern	NEG	NEG	NEG	NEG	NEG	NEG	NEG	NEG	NEG	NEG	NEG	NEG	NEG	NEG	NEG	POS	NEG	POS	NEG	POS	NEG
Finch isolate Zoo-28	NEG	NEG	NEG	NEG	NEG	NEG	NEG	NEG	NEG	NEG	NEG	NEG	NEG	NEG	NEG	NEG	NEG	POS	NEG	POS	NEG
**"S. singaporensis", CC6105**																					
SS21: in silico predicted hybridisation pattern	NEG	NEG	NEG	POS	NEG	NEG	NEG	NEG	NEG	NEG	NEG	NEG	NEG	NEG	NEG	NEG	POS	POS	AMB	NEG	NEG
DSM11148_Staphylococcus sp. SS21	NEG	NEG	NEG	POS	NEG	NEG	NEG	NEG	NEG	NEG	NEG	NEG	NEG	NEG	NEG	NEG	POS	POS	NEG	AMB	NEG
**"S. singaporensis", CC6106**																					
SS35: in silico predicted hybridisation pattern	NEG	NEG	NEG	POS	NEG	NEG	NEG	NEG	POS	NEG	AMB	NEG	NEG	NEG	NEG	NEG	POS	NEG	NEG	NEG	NEG
**"S. singaporensis", CC6107**																					
SS60: in silico predicted hybridisation pattern	NEG	NEG	NEG	NEG	NEG	NEG	NEG	NEG	POS	NEG	NEG	NEG	NEG	NEG	NEG	NEG	NEG	NEG	NEG	NEG	POS
**"S. singaporensis", CC6108**																					
SS87: in silico predicted hybridisation pattern	NEG	POS	NEG	NEG	NEG	NEG	NEG	NEG	NEG	NEG	NEG	NEG	NEG	NEG	NEG	POS	NEG	NEG	NEG	NEG	NEG
**"S. singaporensis", CC6109**																					
SS90: in silico predicted hybridisation pattern	NEG	NEG	NEG	POS	NEG	NEG	NEG	POS	NEG	NEG	NEG	NEG	NEG	NEG	NEG	POS	NEG	NEG	NEG	NEG	NEG
SS251: in silico predicted hybridisation pattern	NEG	NEG	NEG	POS	NEG	NEG	NEG	POS	NEG	NEG	NEG	NEG	NEG	NEG	NEG	POS	NEG	NEG	NEG	NEG	NEG
**"S. roterodami", CC6999**																					
EMCR19: in silico predicted hybridisation pattern	NEG	NEG	NEG	POS	NEG	NEG	NEG	NEG	NEG	NEG	NEG	POS	POS	NEG	NEG	POS	NEG	POS	AMB	NEG	NEG
DSM111914_Staphylococcus sp. EMCR19	NEG	NEG	NEG	POS	NEG	NEG	NEG	NEG	NEG	NEG	NEG	AMB	POS	NEG	NEG	NEG	NEG	POS	NEG	AMB	NEG
**S. argenteus, CC1850**																					
MSHR1132: in silico predicted hybridisation pattern	NEG	NEG	NEG	POS	NEG	NEG	NEG	NEG	NEG	NEG	NEG	NEG	NEG	NEG	NEG	POS	POS	NEG	NEG	NEG	NEG
DSM28299	NEG	NEG	NEG	POS	NEG	NEG	NEG	NEG	NEG	NEG	NEG	AMB	NEG	NEG	NEG	POS	POS	NEG	NEG	NEG	NEG
**S. schweitzeri, CC2022**																					
NCTC13712: in silico predicted hybridisation pattern	NEG	NEG	NEG	NEG	NEG	NEG	NEG	NEG	NEG	NEG	NEG	NEG	NEG	NEG	NEG	NEG	NEG	AMB	NEG	POS	NEG
DSM28300	NEG	NEG	NEG	NEG	NEG	NEG	NEG	NEG	NEG	NEG	POS	NEG	NEG	NEG	NEG	NEG					
**S. saureus, CC1**																					
MW2: in silico predicted hybridisation pattern	NEG	NEG	POS	NEG	NEG	POS	POS	NEG	NEG	NEG	POS	NEG	NEG	POS	NEG	NEG	NEG	NEG	NEG	NEG	POS
MW2	NEG	NEG	POS	NEG	NEG	POS	POS	NEG	NEG	NEG	POS	NEG	NEG	POS	NEG	NEG	NEG	NEG	NEG	NEG	POS

STRAIN / ISOLATE	MISCELLANEOUS GENES											HYALURONATE LYASE							
	sau								G7ZTC1			hysA1			hysA2				
	sau3AI	sauUSI	sauRF122	sauSO385	sau96I	G7ZRU6	ycjY	sagD	G7ZTC1	G7ZTC1-argenteus	sdrM / tetEfflux	hysA1 (MRSA252)	hysA1 (MRSA252+RF122) and/or hysA2 (cons)	hysA1 (MRSA252+RF122) and/or hysA2 (COL+USA300)	hysA2 (All Other Than MRSA252)	hysA2 (COL+USA300+NCTC8325)	hysA2 (All Other Than COL+USA300+NCTC8325)	hysA2 (All Other Than COL+USA300+NCTC8325)	hysA2 (MRSA252)
	type II restriction-modification system endonuclease					acetyltransferase, GNAT family, "Argenteus/ST1850-like"	Marker for "Argenteus/ST1850-like", CC12, CC361, CC398	Putative bacteriocin biosynthesis associated protein	TetR family regulator		Multidrug resistance transporter	Hyaluronate lyase, variable first / second locus			Hyaluronate lyase, second locus				
**CC3960/3961**																			
BDS-53E: in silico predicted hybridisation pattern	NEG	POS	NEG	NEG	NEG	NEG	POS	NEG	POS	NEG	NEG	NEG	POS	NEG	POS	NEG	AMB	POS	NEG
Bat isolate BDS-53E	NEG	POS	NEG	NEG	NEG	NEG	POS	NEG	POS	NEG	NEG	NEG	POS	NEG	POS	NEG	POS	POS	NEG
Bat isolate BDS-53B	NEG	AMB	NEG	NEG	NEG	NEG	POS	NEG	POS	NEG	NEG	NEG	POS	NEG	POS	NEG	POS	POS	NEG
BDS-54: in silico predicted hybridisation pattern	NEG	POS	NEG	NEG	NEG	NEG	NEG	NEG	NEG	NEG	NEG	NEG	POS	NEG	POS	NEG	AMB	POS	NEG
Bat isolate BDS-54	NEG	AMB	NEG	NEG	NEG	NEG	NEG	NEG	NEG	NEG	NEG	NEG	POS	NEG	POS	NEG	POS	POS	NEG
Bat isolate BDH-128											NEG	NEG	POS	NEG	POS	NEG	POS	POS	NEG
Bat isolate BDH-157	NEG	POS	NEG	NEG	NEG	NEG	NEG	NEG	NEG	NEG	NEG	NEG	POS	NEG	POS	NEG	POS	POS	NEG
Bat isolate BDS-69C	NEG	NEG	NEG	NEG	NEG	NEG	POS	NEG	POS	NEG	NEG	NEG	POS	NEG	POS	NEG	POS	POS	NEG
Bat isolate BDH-147	NEG	POS	NEG	NEG	NEG	NEG	POS	NEG	POS	NEG	NEG	NEG	POS	NEG	POS	NEG	POS	POS	NEG
**CC7342**																			
Zoo-28: in silico predicted hybridisation pattern	NEG	NEG	POS	NEG	NEG	NEG	POS	NEG	POS	NEG	NEG	POS	POS	POS	AMB	NEG	POS	AMB	NEG
Finch isolate Zoo-28	NEG	NEG	POS	NEG	NEG	NEG	POS	NEG	POS	NEG	NEG	POS	POS	POS	NEG	POS	POS	AMB	NEG
**"S. singaporensis", CC6105**																			
SS21: in silico predicted hybridisation pattern	NEG	POS	NEG	NEG	NEG	NEG	POS	NEG	POS	NEG	NEG	NEG	POS	NEG	NEG	NEG	AMB	POS	NEG
DSM11148_Staphylococcus sp. SS21	NEG	POS	NEG	NEG	NEG	NEG	POS	NEG	POS	NEG	NEG	NEG	NEG	NEG	NEG	NEG	POS	POS	NEG
**"S. singaporensis", CC6106**																			
SS35: in silico predicted hybridisation pattern	NEG	POS	NEG	NEG	NEG	NEG	POS	NEG	POS	NEG	NEG	NEG	POS	NEG	POS	NEG	POS	NEG	NEG
**"S. singaporensis", CC6107**																			
SS60: in silico predicted hybridisation pattern	NEG	POS	NEG	NEG	NEG	NEG	POS	NEG	POS	NEG	NEG	NEG	POS	NEG	NEG	NEG	POS	POS	NEG
**"S. singaporensis", CC6108**																			
SS87: in silico predicted hybridisation pattern	NEG	NEG	NEG	NEG	NEG	NEG	POS	NEG	POS	NEG	NEG	NEG	POS	NEG	AMB	NEG	POS	NEG	NEG
**"S. singaporensis", CC6109**																			
SS90: in silico predicted hybridisation pattern	NEG	POS	NEG	NEG	NEG	NEG	POS	NEG	POS	NEG	NEG	NEG	POS	NEG	POS	NEG	POS	AMB	NEG
SS251: in silico predicted hybridisation pattern	NEG	POS	NEG	NEG	NEG	NEG	POS	NEG	POS	NEG	NEG	NEG	POS	NEG	POS	NEG	POS	AMB	NEG
**"S. roterodami", CC6999**																			
EMCR19: in silico predicted hybridisation pattern	NEG	NEG	NEG	NEG	POS	NEG	POS	NEG	POS	NEG	NEG	NEG	POS	NEG	NEG	NEG	POS	AMB	NEG
DSM111914_Staphylococcus sp. EMCR19	NEG	NEG	NEG	NEG	POS	NEG	POS	NEG	POS	NEG	NEG	NEG	POS	NEG	NEG	NEG	POS	POS	NEG
**S. argenteus, CC1850**																			
MSHR1132: in silico predicted hybridisation pattern	NEG	POS	NEG	NEG	NEG	POS	POS	NEG	NEG	POS	NEG	POS	POS	NEG	NEG	NEG	NEG	NEG	NEG
DSM28299	NEG	POS	NEG	NEG	NEG	POS	POS	NEG	NEG	POS	NEG	POS	POS	NEG	NEG	POS	NEG	NEG	NEG
**S. schweitzeri, CC2022**																			
NCTC13712: in silico predicted hybridisation pattern	NEG	POS	NEG	NEG	NEG	NEG	NEG	POS	NEG	NEG	NEG	NEG	POS	AMB	NEG	NEG	NEG	NEG	NEG
DSM28300											NEG	NEG	POS	POS	NEG	POS	NEG	NEG	NEG
**S. saureus, CC1**																			
MW2: in silico predicted hybridisation pattern	NEG	POS	NEG	NEG	NEG	NEG	NEG	NEG	NEG	NEG	POS	NEG	POS	NEG	POS	NEG	POS	AMB	NEG
MW2	NEG	POS	NEG	NEG	NEG	NEG	NEG	NEG	NEG	NEG	POS	NEG	POS	NEG	POS	NEG	POS	POS	NEG
