# Supplementary material for: Description of Staphylococcal Strains from Straw-Coloured Fruit Bat (Eidolon helvum) and Diamond Firetail (Stagonopleura guttata) and a Review of their Phylogenetic Relationships to Other Staphylococci
Source: Front Cell Infect Microbiol. 2022 May 11;12:878137. doi: 10.3389/fcimb.2022.878137 (PMC9132046; doi:10.3389/fcimb.2022.878137)
Supplement: Supplemental File 1 — Hybridisation profiles of study isolates and reference strains (pdf). [file DataSheet_1.zip › Supplemental File 2_Vitek Data.pdf]

BDS-53 E, ID

bioMerieux-Kunde: | Laborbefund | Gedruckt am 05.02.2021 22:14 CET
Systemnr.: | | Gedruckt von: labsuper

Referenznummer: Flederm_53E_ID95156-1

Kartentyp: GP Testgerät: 000015F14408 (13294)

Bionummer: 050402033673211
Keimzahl:

**Kommentare:**	

Infos zur Identifizierung	**Karte:**	GP	**Chargenbez:**	2421168403	**Verfallsdatum:**	06.02.2021 12:00 CET
	**Beendet:**	03.02.2021 10:05 CET	**Status:**	Fertig	**Analysen-Dauer:**	6,00 Std.
Gewählter Keim	90% Wahrscheinlichkeit		**Staphylococcus aureus**			
	**Bionummer:**	050402033673211			**Beurteilung:**	Gute Identifizierung
**SRF-Keim**						
**Analyse der Keime und Tests aufsplitten:**						
**Meldungen zur Analyse:**						
**Widersprüchliche(s) typische(s) Biomuster**						
Staphylococcus aureus	NOVO(1),URE(2),					

Biochemische Details

2	AMY	-	4	PIPLC	-	5	dXYL	-	8	ADH1	+	9	BGAL	-	11	AGLU	+
13	APPA	-	14	CDEX	-	15	AspA	-	16	BGAR	-	17	AMAN	-	19	PHOS	+
20	LeuA	-	23	ProA	-	24	BGURr	-	25	AGAL	-	26	PyrA	+	27	BGUR	-
28	AlaA	-	29	TyrA	-	30	dSOR	-	31	URE	+	32	POLYB	+	37	dGAL	-
38	dRIB	+	39	ILATk	+	42	LAC	-	44	NAG	-	45	dMAL	+	46	BACI	+
47	NOVO	+	50	NC6.5	+	52	dMAN	+	53	dMNE	+	54	MBdG	+	56	PUL	-
57	dRAF	-	58	O129R	+	59	SAL	-	60	SAC	+	62	dTRE	-	63	ADH2s	-
64	OPTO	+															

Aktion	Name (Anwender ID)	Datum/Uhrzeit	Kommentar
**Geprüft von:**	**(labsuper)**	**04.02.2021 00:17 CET**	

Installierte VITEK 2 Systems Version: 07.01
MHK-Interpretationsrichtlinie: EUCAST 2014 + CLSI 2014 D
Therapeutische Interpretationsrichtlinie: DEUTSCHLAND PHENOTYPIC 2014
Bezeichnung des AES-Parametersets: EUCAST/CLSI + PHAENOTYPISCH 2014 D
Letzte Änderung der AES-Parameter: 25.11.2014 12:06 CET

BDS-53 E, AST

bioMerieux-Kunde:
Systemnr.:

Laborbefund

Gedruckt am 04.02.2021 00:18 CET
Gedruckt von: labsuper

Referenznummer: Flederm_53E_ID95156-1

Kartentyp: GP Testgerät: 000015F14408 (13294)
Kartentyp: AST-P608 Testgerät: 000015F14408 (13294)

Bionummer: 050402033673211

Infos zur Resistenz	**Karte:** AST-P608		**Chargenbez:** 4881414403		**Verfallsdatum:** 10.10.2021 13:00 CEST
	**Beendet:** 03.02.2021 12:05 CET		**Status:** Fertig		**Analysen-Dauer:** 8,00 Std.
**Antibiotikum**	**MHK**	**Interpretation**	**Antibiotikum**	**MHK**	**Interpretation**
Cefoxitin-Screen	NEG	-	Linezolid	2	S
Benzylpenicillin	<= 0,03	S	Teicoplanin	<= 0,5	S
Oxacillin	<= 0,25	S	Vancomycin	1	S
Gentamicin	<= 0,5	S	Tetracyclin	<= 1	S
Tobramycin	<= 1	S	Fosfomycin	<= 8	S
Ciprofloxacin	<= 0,5	S	Nitrofurantoin	<= 16	S
Levofloxacin	0,25	S	Fusidinsäure	<= 0,5	S
Moxifloxacin	<= 0,25	S	Mupirocin	<= 2	S
Induzierbare Clindamycin Resistenz	NEG	-	Rifampicin	<= 0,5	S
Erythromycin	<= 0,25	S	Trimethoprim/Sulfamethoxazol	<= 10	S
Clindamycin	<= 0,25	S			

+= Abgeleitete Antibiotika *= AES modifiziert **= Anwender modifiziert

AES-Befunde:		**Letzte Änderung:** 25.11.2014 12:06 CET	**Parameterset:** EUCAST/CLSI + PHAENOTYPISCH 2014 D
**Zuverlässigkeit-Ebene:**	Konsistent		
**Zur Prüfung markierte Phänotypen:**	MAKROLIDE/LINCOSAMIDE/STREPTOGRAMINE	STREPTOGRAMINE-RESISTENZ (SGA-SGB)	
	MUPIROCIN	LOW-LEVEL-RESISTENZ	

**Aktion**	**Name (Anwender ID)**	**Datum/Uhrzeit**	**Kommentar**
**Geprüft von:**	**(labsuper)**	**04.02.2021 00:17 CET**	

Installierte VITEK 2 Systems Version: 07.01
MHK-Interpretationsrichtlinie: EUCAST 2014 + CLSI 2014 D
Therapeutische Interpretationsrichtlinie: DEUTSCHLAND PHENOTYPIC 2014
Bezeichnung des AES-Parametersets: EUCAST/CLSI + PHAENOTYPISCH 2014 D
Letzte Änderung der AES-Parameter: 25.11.2014 12:06 CET

BDS-54, ID

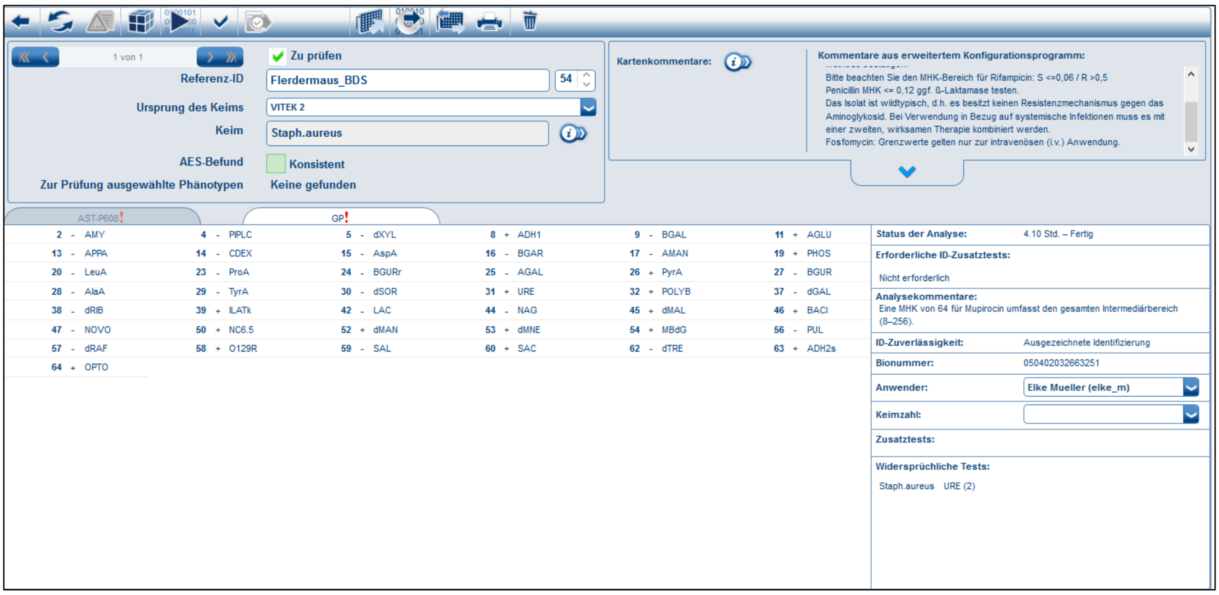
1 von 1
Zu prüfen
Referenz-ID
Flerdermaus_BDS
54
Ursprung des Keims
VITEK 2
Keim
Staph.aureus
AES-Befund
Konsistent
Zur Prüfung ausgewählte Phänotypen
Keine gefunden
Kartenkommentare:
Kommentare aus erweitertem Konfigurationsprogramm:
Bitte beachten Sie den MHK-Bereich für Rifampicin: S <=0,06 / R >0,5
Penicillin MHK <= 0,12 ggf. ß-Laktamase testen.
Das Isolat ist wildtypisch, d.h. es besitzt keinen Resistenzmechanismus gegen das Aminoglykosid. Bei Verwendung in Bezug auf systemische Infektionen muss es mit einer zweiten, wirksamen Therapie kombiniert werden.
Fosfomycin: Grenzwerte gelten nur zur intravenösen (i.v.) Anwendung.
AST-P608!
GP!
2 - AMY | 4 - PIPLC | 5 - dXYL | 8 + ADH1 | 9 - BGAL | 11 + AGLU
13 - APPA | 14 - CDEX | 15 - AspA | 16 - BGAR | 17 - AMAN | 19 + PHOS
20 - LeuA | 23 - ProA | 24 - BGURr | 25 - AGAL | 26 + PyrA | 27 - BGUR
28 - AlaA | 29 - TyrA | 30 - dSOR | 31 + URE | 32 + POLYB | 37 - dGAL
38 - dRIB | 39 + ILATk | 42 - LAC | 44 - NAG | 45 + dMAL | 46 + BACI
47 - NOVO | 50 + NC6.5 | 52 + dMAN | 53 + dMNE | 54 + MBdG | 56 - PUL
57 - dRAF | 58 + O129R | 59 - SAL | 60 + SAC | 62 - dTRE | 63 + ADH2s
64 + OPTO
Status der Analyse: 4.10 Std. – Fertig
Erforderliche ID-Zusatztests:
Nicht erforderlich
Analysekommentare:
Eine MHK von 64 für Mupirocin umfasst den gesamten Intermediärbereich (8–256).
ID-Zuverlässigkeit: Ausgezeichnete Identifizierung
Bionummer: 050402032663251
Anwender: Elke Mueller (elke_m)
Keimzahl:
Zusatztests:
Widersprüchliche Tests:
Staph.aureus URE (2)

BDS-54, AST

AG Ehricht
Laborbefund

bioMérieux-Kunde: 1110869
Systemnr.: 19553
Isolat: Flerdermaus_BDS–54 (Zu prüfen)
Kartentyp: GP Barcode: 2421604403235121 Analysegerät: 00001B1B4286 (19553)
Kartentyp: AST-P608 Barcode: 4881731203403419 Analysegerät: 00001B1B4286 (19553)
Anwender: Elke Mueller(elke_m)

Gedruckt von: elke_m

Bionummer: 050402032663251
Keimzahl:

Gewählter Keim: Staphylococcus aureus

**Kommentare:**	Gemäss Packungsbeilage Limitierung für Erythromycin. Bitte Ergebnis mit alternativer Methode bestätigen.<br>Bitte beachten Sie den MHK-Bereich für Rifampicin: S <=0,06 / R >0,5<br>Penicillin MHK <= 0,12 ggf. ß-Laktamase testen.<br>Das Isolat ist wildtypisch, d.h. es besitzt keinen Resistenzmechanismus gegen das Aminoglykosid. Bei Verwendung in Bezug auf systemische Infektionen muss es mit einer zweiten, wirksamen Therapie kombiniert werden.<br>Fosfomycin: Grenzwerte gelten nur zur intravenösen (i.v.) Anwendung.

**Infos zur Identifizierung**	**Karte:** GP	**Chargenbez:** 2421604403	**Verfallsdatum:** 18.04.2022 13:00 MESZ
	**Status:** Fertig	**Analysen- Dauer:** 4,10 Std.	**Beendet:** 29.09.2021 14:26 MESZ
**Ursprung des Keims**	VITEK 2		
**Gewählter Keim**	96% Wahrscheinlichkeit **Staphylococcus aureus**<br>Bionummer: 050402032663251 Beurteilung: Ausgezeichnete Identifizierung		
**SRF-Keim**			

Analyse der Keime und Tests aufsplitten:

Meldungen zur Analyse:
Eine MHK von 64 für Mupirocin umfasst den gesamten Intermediärbereich (8–256).

Widersprüchliche(s) typische(s) Biomuster
Staphylococcus aureus URE(2),

**Infos zur Resistenz**	**Karte:** AST-P608	**Chargenbez:** 4881731203	**Verfallsdatum:** 23.08.2022 13:00 MESZ
	**Status:** Fertig	**Analysen- Dauer:** 12,65 Std.	**Beendet:** 29.09.2021 22:59 MESZ

Antibiotikum	MHK	Interpretation	Antibiotikum	MHK	Interpretation
Cefoxitin-Screen	NEG	-	Linezolid	2	S
Benzylpenicillin	0,06	S	Teicoplanin	<= 0,5	S
Oxacillin	<= 0,25	S	Vancomycin	<= 0,5	S
Gentamicin	<= 0,5	S	Tetracyclin	<= 1	S
Tobramycin	<= 1	S	Fosfomycin	<= 8	S
Ciprofloxacin	<= 0,5	I	Nitrofurantoin		
Levofloxacin	0,25	I	Fusidinsäure	<= 0,5	S
Moxifloxacin	<= 0,25	S	Mupirocin	<= 2	I
Induzierbare Clindamycin Resistenz	NEG	-	Rifampicin	<= 0,5	I
Erythromycin	<= 0,25	S	Trimethoprim/Sulfamethoxazol	<= 10	S
Clindamycin	<= 0,25	S			

Installierte VITEK 2 Systems Version: 9.02
MHK-Interpretationsrichtlinie: EUCAST 2021
Bezeichnung des AES-Parametersets: EUCAST+Phenotypic DE 2021

Therapeutische Interpretationsrichtlinie: PHENOTYPISCH DE 2021
Letzte Änderung der AES-Parameter: 23.08.2021 12:23 MESZ

Zoo-28, ID

bioMerieux-Kunde:
Systemnr.:

Laborbefund

Gedruckt am 05.02.2021 22:13 CET
Gedruckt von: labsuper

Referenznummer: FLI_Zoo28_ID95376-1

Kartentyp: GP Testgerät: 000015F14408 (13294)

Bionummer: 010400063663231
Keimzahl:

**Kommentare:**	

Infos zur Identifizierung	**Karte:** GP	**Chargenbez:** 2421168403	**Verfallsdatum:**	06.02.2021 12:00 CET
	**Beendet:** 03.02.2021 08:06 CET	**Status:** Fertig	**Analysen-Dauer:**	4,00 Std.
Gewählter Keim	99% Wahrscheinlichkeit	**Staphylococcus aureus**		
	**Bionummer:** 010400063663231		**Beurteilung:**	Ausgezeichnete Identifizierung
**SRF-Keim**				
**Analyse der Keime und Tests aufsplitten:**				
**Meldungen zur Analyse:**				
**Widersprüchliche(s) typische(s) Biomuster**				

Biochemische Details

2	AMY	-	4	PIPLC	-	5	dXYL	-	8	ADH1	+	9	BGAL	-	11	AGLU	-
13	APPA	-	14	CDEX	-	15	AspA	-	16	BGAR	-	17	AMAN	-	19	PHOS	+
20	LeuA	-	23	ProA	-	24	BGURr	-	25	AGAL	-	26	PyrA	-	27	BGUR	-
28	AlaA	-	29	TyrA	-	30	dSOR	-	31	URE	-	32	POLYB	+	37	dGAL	+
38	dRIB	+	39	ILATk	+	42	LAC	-	44	NAG	-	45	dMAL	+	46	BACI	+
47	NOVO	-	50	NC6.5	+	52	dMAN	+	53	dMNE	+	54	MBdG	+	56	PUL	-
57	dRAF	-	58	O129R	+	59	SAL	-	60	SAC	+	62	dTRE	+	63	ADH2s	-
64	OPTO	+															

**Aktion**	**Name (Anwender ID)**	**Datum/Uhrzeit**	**Kommentar**
**Geprüft von:**	**(labsuper)**	**04.02.2021 00:17 CET**	

Installierte VITEK 2 Systems Version: 07.01
MHK-Interpretationsrichtlinie: EUCAST 2014 + CLSI 2014 D

Therapeutische Interpretationsrichtlinie: DEUTSCHLAND PHENOTYPIC 2014

Bezeichnung des AES-Parametersets: EUCAST/CLSI + PHAENOTYPISCH 2014 D

Letzte Änderung der AES-Parameter: 25.11.2014 12:06 CET

Zoo28, AST

bioMerieux-Kunde:
Systemnr.:

Laborbefund

Gedruckt am 04.02.2021 00:18 CET
Gedruckt von: labsuper

Referenznummer: FLI_Zoo28_ID95376-1

Kartentyp: GP Testgerät: 000015F14408 (13294)
Kartentyp: AST-P608 Testgerät: 000015F14408 (13294)

Bionummer: 010400063663231

Infos zur Resistenz	Karte: AST-P608		Chargenbez: 4881414403	Verfallsdatum: 10.10.2021 13:00 CEST	
	Beendet: 03.02.2021 12:21 CET		Status: Fertig	Analysen-Dauer: 8,25 Std.	
**Antibiotikum**	**MHK**	**Interpretation**	**Antibiotikum**	**MHK**	**Interpretation**
Cefoxitin-Screen	NEG	-	Linezolid	2	S
Benzylpenicillin	>= 0,5	R	Teicoplanin	<= 0,5	S
Oxacillin	<= 0,25	S	Vancomycin	1	S
Gentamicin	<= 0,5	S	Tetracyclin	>= 16	R
Tobramycin	<= 1	S	Fosfomycin	<= 8	S
Ciprofloxacin	<= 0,5	S	Nitrofurantoin	<= 16	S
Levofloxacin	<= 0,12	S	Fusidinsäure	<= 0,5	S
Moxifloxacin	<= 0,25	S	Mupirocin	<= 2	S
Induzierbare Clindamycin Resistenz	NEG	-	Rifampicin	<= 0,5	S
Erythromycin	<= 0,25	S	Trimethoprim/Sulfamethoxazol	<= 10	S
Clindamycin	<= 0,25	S			

+= Abgeleitete Antibiotika *= AES modifiziert **= Anwender modifiziert

AES-Befunde:		Letzte Änderung: 25.11.2014 12:06 CET	Parameterset: EUCAST/CLSI + PHAENOTYPISCH 2014 D
**Zuverlässigkeit-Ebene:**	Konsistent		
**Zur Prüfung markierte Phänotypen:**	MAKROLIDE/LINCOSAMIDE/STREPTOGRAMINE	STREPTOGRAMINE-RESISTENZ (SGA-SGB)	
	MUPIROCIN	LOW-LEVEL-RESISTENZ	

**Aktion**	**Name (Anwender ID)**	**Datum/Uhrzeit**	**Kommentar**
**Geprüft von:**	**(labsuper)**	**04.02.2021 00:17 CET**	

Installierte VITEK 2 Systems Version: 07.01
MHK-Interpretationsrichtlinie: EUCAST 2014 + CLSI 2014 D

Therapeutische Interpretationsrichtlinie: DEUTSCHLAND PHENOTYPIC 2014

Bezeichnung des AES-Parametersets: EUCAST/CLSI + PHAENOTYPISCH 2014 D

Letzte Änderung der AES-Parameter: 25.11.2014 12:06 CET

DSM111408 (“*S. singaporensis*” SS21), ID

AG Ehricht

Laborbefund

bioMérieux-Kunde: 1110869
Systemnr.: 19553
Isolat: DSM111408-2–1 (Zu prüfen)
Kartentyp: GP Barcode: 2421604403235104 Analysegerät: 00001B1B4286 (19553)
Anwender: Elke Mueller(elke_m)

Gedruckt von: elke_m

Bionummer: 050402063662231
Keimzahl:

Gewählter Keim: Staphylococcus aureus

**Kommentare:**	

**Infos zur Identifizierung**	**Karte:** GP		**Chargenbez:** 2421604403		**Verfallsdatum:** 18.04.2022 13:00 MESZ	
	**Status:** Fertig		**Analysen- Dauer:** 3,90 Std.		**Beendet:** 07.02.2022 19:26 MEZ	
**Ursprung des Keims**	VITEK 2					
**Gewählter Keim**	99% Wahrscheinlichkeit **Staphylococcus aureus**<br>Bionummer: 050402063662231 Beurteilung: Ausgezeichnete Identifizierung					
**SRF-Keim**						
**Analyse der Keime und Tests aufsplitten:**						
**Meldungen zur Analyse:**						
**Widersprüchliche(s) typische(s) Biomuster**						

Biochemische Details

2	AMY	-	4	PIPLC	-	5	dXYL	-	8	ADH1	+	9	BGAL	-	11	AGLU	+
13	APPA	-	14	CDEX	-	15	AspA	-	16	BGAR	-	17	AMAN	-	19	PHOS	+
20	LeuA	-	23	ProA	-	24	BGURr	-	25	AGAL	-	26	PyrA	+	27	BGUR	-
28	AlaA	-	29	TyrA	-	30	dSOR	-	31	URE	-	32	POLYB	+	37	dGAL	+
38	dRIB	+	39	lLATk	+	42	LAC	-	44	NAG	-	45	dMAL	+	46	BACI	+
47	NOVO	-	50	NC6.5	+	52	dMAN	+	53	dMNE	-	54	MBdG	+	56	PUL	-
57	dRAF	-	58	O129R	+	59	SAL	-	60	SAC	+	62	dTRE	+	63	ADH2s	-
64	OPTO	+															

Installierte VITEK 2 Systems Version: 9.02
MHK-Interpretationsrichtlinie:
Bezeichnung des AES-Parametersets:

Therapeutische Interpretationsrichtlinie:
Letzte Änderung der AES-Parameter:

DSM111408 (“*S. singaporensis*” SS21), AST

AG Ehricht
Laborbefund

bioMérieux-Kunde: 1110869
Systemnr.: 19553
Isolat: DSM111408–1 (Zu prüfen)
Kartentyp: GP Barcode: 2421604403235104 Analysegerät: 00001B1B4286 (19553)
Kartentyp: AST-P608 Barcode: 4881731203403401 Analysegerät: 00001B1B4286 (19553)
Anwender: Elke Mueller(elke_m)

Gedruckt von: elke_m

Bionummer: 050402063662231
Keimzahl:

Gewählter Keim: Staphylococcus aureus

**Kommentare:**	Gemäss Packungsbeilage Limitierung für Erythromycin. Bitte Ergebnis mit alternativer Methode bestätigen.<br>Bitte beachten Sie den MHK-Bereich für Rifampicin: S <=0,06 / R >0,5<br>Das Isolat ist wildtypisch, d.h. es besitzt keinen Resistenzmechanismus gegen das Aminoglykosid. Bei Verwendung in Bezug auf systemische Infektionen muss es mit einer zweiten, wirksamen Therapie kombiniert werden.<br>Fosfomycin: Grenzwerte gelten nur zur intravenösen (i.v.) Anwendung.

**Infos zur Identifizierung**	**Karte:** GP		**Chargenbez:**	2421604403	**Verfallsdatum:**	18.04.2022 13:00 MESZ
	**Status:** Fertig		**Analysen- Dauer:**	3,90 Std.	**Beendet:**	07.02.2022 19:26 MEZ
**Ursprung des Keims**	VITEK 2					
**Gewählter Keim**	99% Wahrscheinlichkeit	**Staphylococcus aureus**				
	Bionummer: 050402063662231			Beurteilung: Ausgezeichnete Identifizierung		
**SRF-Keim**						
**Analyse der Keime und Tests aufsplitten:**						
**Meldungen zur Analyse:**<br>Eine MHK von 64 für Mupirocin umfasst den gesamten Intermediärbereich (8–256).						
**Widersprüchliche(s) typische(s) Biomuster**						

**Infos zur Resistenz**	**Karte:** AST-P608	**Chargenbez:** 4881731203	**Verfallsdatum:**	23.08.2022 13:00 MESZ
	**Status:** Fertig	**Analysen- Dauer:** 12,70 Std.	**Beendet:**	08.02.2022 04:13 MEZ

Antibiotikum	MHK	Interpretation	Antibiotikum	MHK	Interpretation
Cefoxitin-Screen	NEG	-	Linezolid	1	S
Benzylpenicillin	0,12	S	Teicoplanin	2	S
Oxacillin	0,5	S	Vancomycin	<= 0,5	S
Gentamicin	<= 0,5	S	Tetracyclin	<= 1	S
Tobramycin	<= 1	S	Fosfomycin	<= 8	S
Ciprofloxacin	<= 0,5	I	Nitrofurantoin		
Levofloxacin	0,25	I	Fusidinsäure	<= 0,5	S
Moxifloxacin	<= 0,25	S	Mupirocin	<= 2	I
Induzierbare Clindamycin Resistenz	NEG	-	Rifampicin	<= 0,5	I
Erythromycin	<= 0,25	S	Trimethoprim/Sulfamethoxazol	<= 10	S
Clindamycin	<= 0,25	S			

Installierte VITEK 2 Systems Version: 9.02
MHK-Interpretationsrichtlinie: EUCAST 2021
Bezeichnung des AES-Parametersets: EUCAST+Phenotypic DE 2021

Therapeutische Interpretationsrichtlinie: PHENOTYPISCH DE 2021
Letzte Änderung der AES-Parameter: 23.08.2021 12:23 MESZ

DSM111914 (“*S. roterodami*” EMCR19), ID

AG Ehricht

Laborbefund

bioMérieux-Kunde: 1110869
Systemnr.: 19553
Isolat: DSM111914-2–1 (Zu prüfen)
Kartentyp: GP Barcode: 2421604403235105 Analysegerät: 00001B1B4286 (19553)
Anwender: Elke Mueller(elke_m)

Gedruckt von: elke_m

Bionummer: 010402062663231
Keimzahl:

Gewählter Keim: Staphylococcus aureus

**Kommentare:**	

**Infos zur Identifizierung**	**Karte:** GP	**Chargenbez:** 2421604403	**Verfallsdatum:** 18.04.2022 13:00 MESZ
	**Status:** Fertig	**Analysen- Dauer:** 3,88 Std.	**Beendet:** 07.02.2022 19:24 MEZ
**Ursprung des Keims**	VITEK 2		
**Gewählter Keim**	99% Wahrscheinlichkeit **Staphylococcus aureus**<br>Bionummer: 010402062663231 Beurteilung: Ausgezeichnete Identifizierung		
**SRF-Keim**			

Analyse der Keime und Tests aufsplitten:

Meldungen zur Analyse:

Widersprüchliche(s) typische(s) Biomuster

Biochemische Details

2	AMY	-	4	PIPLC	-	5	dXYL	-	8	ADH1	+	9	BGAL	-	11	AGLU	-
13	APPA	-	14	CDEX	-	15	AspA	-	16	BGAR	-	17	AMAN	-	19	PHOS	+
20	LeuA	-	23	ProA	-	24	BGURr	-	25	AGAL	-	26	PyrA	+	27	BGUR	-
28	AlaA	-	29	TyrA	-	30	dSOR	-	31	URE	-	32	POLYB	+	37	dGAL	+
38	dRIB	-	39	lLATk	+	42	LAC	-	44	NAG	-	45	dMAL	+	46	BACI	+
47	NOVO	-	50	NC6.5	+	52	dMAN	+	53	dMNE	+	54	MBdG	+	56	PUL	-
57	dRAF	-	58	O129R	+	59	SAL	-	60	SAC	+	62	dTRE	+	63	ADH2s	-
64	OPTO	+															

Installierte VITEK 2 Systems Version: 9.02
MHK-Interpretationsrichtlinie:
Bezeichnung des AES-Parametersets:

Therapeutische Interpretationsrichtlinie:
Letzte Änderung der AES-Parameter:

DSM111914 (“*S. roterodami*” EMCR19), AST

AG Ehricht

Laborbefund

bioMérieux-Kunde: 1110869
Systemnr.: 19553
Isolat: DSM111914–1 (Zu prüfen)
Kartentyp: GP Barcode: 2421604403235105 Analysegerät: 00001B1B4286 (19553)
Kartentyp: AST-P608 Barcode: 4881731203403402 Analysegerät: 00001B1B4286 (19553)
Anwender: Elke Mueller(elke_m)

Gedruckt von: elke_m

Bionummer: 010402062663231
Keimzahl:

Gewählter Keim: Staphylococcus aureus

**Kommentare:**	Gemäss Packungsbeilage Limitierung für Erythromycin. Bitte Ergebnis mit alternativer Methode bestätigen.<br>Bitte beachten Sie den MHK-Bereich für Rifampicin: S <=0,06 / R >0,5<br>Penicillin MHK <= 0,12 ggf. ß-Laktamase testen.<br>Das Isolat ist wildtypisch, d.h. es besitzt keinen Resistenzmechanismus gegen das Aminoglykosid. Bei Verwendung in Bezug auf systemische Infektionen muss es mit einer zweiten, wirksamen Therapie kombiniert werden.<br>Fosfomycin: Grenzwerte gelten nur zur intravenösen (i.v.) Anwendung.

**Infos zur Identifizierung**	**Karte:** GP<br>**Status:** Fertig	**Chargenbez:** 2421604403<br>**Analysen- Dauer:** 3,88 Std.	**Verfallsdatum:** 18.04.2022 13:00 MESZ<br>**Beendet:** 07.02.2022 19:24 MEZ
**Ursprung des Keims**	VITEK 2		
**Gewählter Keim**	99% Wahrscheinlichkeit **Staphylococcus aureus**<br>Bionummer: 010402062663231		Beurteilung: Ausgezeichnete Identifizierung
**SRF-Keim**			
**Analyse der Keime und Tests aufsplitten:**			
**Meldungen zur Analyse:**<br>Eine MHK von 64 für Mupirocin umfasst den gesamten Intermediärbereich (8–256).			
**Widersprüchliche(s) typische(s) Biomuster**			

**Infos zur Resistenz**	**Karte:** AST-P608<br>**Status:** Fertig	**Chargenbez:** 4881731203<br>**Analysen- Dauer:** 12,68 Std.	**Verfallsdatum:** 23.08.2022 13:00 MESZ<br>**Beendet:** 08.02.2022 04:12 MEZ

Antibiotikum	MHK	Interpretation	Antibiotikum	MHK	Interpretation
Cefoxitin-Screen	NEG	-	Linezolid	2	S
Benzylpenicillin	0,06	S	Teicoplanin	<= 0,5	S
Oxacillin	<= 0,25	S	Vancomycin	1	S
Gentamicin	<= 0,5	S	Tetracyclin	<= 1	S
Tobramycin	<= 1	S	Fosfomycin	<= 8	S
Ciprofloxacin	<= 0,5	I	Nitrofurantoin		
Levofloxacin	0,25	I	Fusidinsäure	<= 0,5	S
Moxifloxacin	<= 0,25	S	Mupirocin	<= 2	I
Induzierbare Clindamycin Resistenz	NEG	-	Rifampicin	<= 0,5	I
Erythromycin	<= 0,25	S	Trimethoprim/Sulfamethoxazol	<= 10	S
Clindamycin	<= 0,25	S			

Installierte VITEK 2 Systems Version: 9.02
MHK-Interpretationsrichtlinie: EUCAST 2021
Bezeichnung des AES-Parametersets: EUCAST+Phenotypic DE 2021

Therapeutische Interpretationsrichtlinie: PHENOTYPISCH DE 2021
Letzte Änderung der AES-Parameter: 23.08.2021 12:23 MESZ
