# Supplementary material for: Description of Staphylococcal Strains from Straw-Coloured Fruit Bat (Eidolon helvum) and Diamond Firetail (Stagonopleura guttata) and a Review of their Phylogenetic Relationships to Other Staphylococci
Source: Front Cell Infect Microbiol. 2022 May 11;12:878137. doi: 10.3389/fcimb.2022.878137 (PMC9132046; doi:10.3389/fcimb.2022.878137)
Supplement: Supplemental File 1 — Hybridisation profiles of study isolates and reference strains (pdf). [file DataSheet_1.zip › Supplemental File 7b_Percentages difference as used for Figures 2a and b.pdf]

Gene	vs. zoo28	vs. EMCR19	vs. SS60	vs. SS90	vs. NCTC13712	vs. MSHR1132	vs MW2	vs. SA17_S6
dnaA	0,51	0,29	0,37	0,51	2,28	1,69	5,43	5,36
dnaN	0,62	0,79	0,79	0,62	3,00	3,88	7,41	7,14
yaaA	0,41	0,00	0,00	0,00	0,81	0,00	1,63	1,63
recF	0,72	0,09	1,89	0,27	4,13	4,04	4,04	4,22
gyrB	1,09	1,60	1,09	1,29	3,46	3,31	7,39	7,34
gyrA	0,64	1,83	0,82	1,05	4,26	3,82	5,99	6,81
nnrD	2,65	2,29	2,65	1,93	6,74	4,21	8,54	8,66
hutH	1,78	1,72	1,58	1,32	6,40	5,28	11,16	11,88
serS	0,62	0,93	0,62	0,93	4,51	2,87	6,22	6,45
azlC	1,58	100,00	0,72	0,43	6,47	6,18	15,23	15,23
azlD	0,90	0,30	0,30	0,00	5,42	4,22	14,76	14,76
metX	0,83	2,17	1,44	1,03	3,72	3,51	12,90	13,21
yybS	0,22	0,43	0,32	0,54	5,41	1,19	8,33	8,01
gdpP	1,12	1,07	1,22	1,17	5,95	3,46	7,88	7,77
rplI	0,44	0,22	0,00	0,66	1,77	1,55	5,08	5,08
dnaC	0,57	0,79	0,64	1,21	3,43	2,57	6,21	6,28
purA	0,70	0,62	0,55	0,55	3,89	3,74	4,28	4,52
walR	1,42	1,28	1,00	1,71	3,28	1,28	3,70	3,85
walK	1,26	1,70	0,93	0,88	4,93	1,92	5,91	6,13
walH	1,72	1,20	1,27	1,05	5,69	3,97	8,76	9,29
walI	1,27	0,76	0,51	0,76	7,35	6,46	8,11	9,38
walJ	1,37	1,50	1,12	2,00	4,11	4,11	1,75	3,37
sasH	4,05	3,41	3,62	3,41	4,10	3,62	4,31	4,48
orfX	3,13	2,92	2,71	3,75	2,50	1,88	2,50	2,71
mcrB	100,00	100,00	100,00	100,00	100,00	100,00	100,00	100,00
mcrC	100,00	100,00	100,00	100,00	100,00	100,00	100,00	100,00
C1PH96	100,00	100,00	0,00	0,00	100,00	0,00	0,00	0,00
H0CD41	0,00	0,00	0,00	0,00	0,00	0,00	0,00	0,00
D2N3D1	0,00	0,00	0,00	0,00	0,00	0,00	0,00	0,00
entH	0,00	0,00	0,00	0,00	0,00	0,00	100,00	0,00
lrpC	100,00	0,00	0,00	0,00	100,00	0,00	18,29	0,00
D9RC56	0,00	0,00	0,00	0,00	0,00	0,00	0,00	0,00
D9RC57	0,00	0,00	0,00	0,00	0,00	0,00	0,00	0,00
hsdM2-CC22_93_425	0,00	0,00	0,00	0,00	0,00	0,00	0,00	0,00
hsdS2-CC22_93_425	0,00	0,00	0,00	0,00	0,00	0,00	0,00	0,00
hsdR2-CC22_93_425	0,00	0,00	0,00	0,00	0,00	0,00	0,00	0,00
F9JXC0	0,00	0,00	0,00	0,00	0,00	0,00	0,00	0,00
Q6GKL3	0,00	0,00	0,00	0,00	0,00	0,00	0,00	0,00
UPI0001C11B31	0,00	0,00	0,00	0,00	0,00	0,00	0,00	0,00
D2N3E0	0,00	0,00	0,00	0,00	0,00	0,00	0,00	0,00
Q6GKL1	0,00	0,00	0,00	0,00	0,00	0,00	0,00	0,00
G7ZLS9	0,00	0,00	0,00	0,00	0,00	100,00	0,00	0,00
G7ZLT3	0,00	0,00	0,00	100,00	0,00	100,00	0,00	0,00
entCM14	0,00	0,00	0,00	0,00	0,00	0,00	0,00	0,00
Q6GD44	2,30	100,00	100,00	100,00	3,13	100,00	3,13	100,00
DUF81-GI	0,00	0,00	0,00	0,00	0,00	0,00	100,00	0,00
cstR-GI	0,00	0,00	0,00	0,00	0,00	0,00	100,00	0,00
cstA-GI	0,00	0,00	100,00	0,00	0,00	0,00	100,00	0,00
cstB-GI	0,00	0,00	100,00	0,00	0,00	0,00	100,00	0,00
sqr	0,00	0,00	100,00	0,00	100,00	0,00	100,00	0,00
dusC	2,53	1,82	2,33	2,33	2,43	3,14	2,33	3,14
A6TXM6	3,41	3,41	100,00	100,00	9,76	8,29	3,90	11,22
A6QD71	5,05	6,06	100,00	4,38	13,80	12,79	7,41	13,47
AGO28474	0,00	100,00	0,00	0,00	0,00	0,00	0,00	0,00
AGO28476	0,00	100,00	0,00	0,00	0,00	0,00	0,00	0,00
AGO28477	0,00	100,00	0,00	0,00	0,00	0,00	0,00	0,00
AGO28478	0,00	100,00	0,00	0,00	0,00	0,00	0,00	0,00
Q5HJT2	100,00	100,00	0,00	0,00	0,00	0,00	100,00	0,00
Q6GD34	0,00	100,00	0,00	0,00	0,00	0,00	100,00	0,00
A6QD75	0,00	100,00	0,00	0,00	0,00	0,00	100,00	0,00
A6QD76	0,00	0,00	0,00	0,00	0,00	0,00	100,00	0,00
A8YZ18	0,00	0,00	0,00	0,00	0,00	0,00	100,00	0,00
G7ZLT6	100,00	0,00	0,00	100,00	0,00	100,00	0,00	0,00
G7ZLT7	100,00	0,00	0,00	100,00	0,00	100,00	0,00	0,00
Q6GKK6	3,27	3,10	2,78	2,45	4,08	6,21	3,27	4,08
Q7A890	0,00	100,00	0,00	0,00	100,00	0,00	100,00	0,00
Q2YUT3	2,17	2,58	1,63	1,36	100,00	100,00	100,00	100,00
Q2YUT2	1,66	2,48	1,66	1,04	3,11	3,11	2,90	3,11
plc	2,33	3,14	2,33	2,94	5,67	6,99	4,26	5,98
Q8NYT6	7,46	6,43	6,34	6,57	8,18	8,49	8,09	8,76
Q8NYT5	1,78	1,70	0,68	1,53	3,56	4,24	2,88	3,73
norC	1,73	1,01	1,44	2,09	100,00	6,05	4,25	4,97
nptA	1,68	1,44	2,47	1,32	5,54	4,63	5,05	5,54
Q2YUS5	2,20	1,97	1,46	1,52	4,84	5,74	4,67	4,28
DUF1648	3,80	3,59	2,32	4,64	9,28	8,65	6,75	7,81
lctP-locus1	1,38	1,57	1,07	1,57	5,21	4,14	4,39	4,46
spa	100,00	100,00	100,00	100,00	100,00	100,00	100,00	100,00
sarS	0,00	0,27	0,27	0,66	7,57	1,73	8,90	9,43
sirC	0,30	0,50	0,40	1,30	9,61	5,31	13,91	13,61
sirB	0,80	2,31	0,10	1,00	8,84	2,81	12,25	13,05
sirA	0,50	1,01	1,01	100,00	3,32	2,21	6,94	7,04
sbnA-cysK1	1,02	0,61	0,92	1,12	3,77	2,55	10,09	10,19
sbnB	0,79	0,89	0,79	0,59	4,65	4,55	10,48	10,39
sbnC	1,88	1,31	1,20	1,54	8,77	7,29	13,85	13,45
sbnD	1,51	1,11	1,19	0,72	11,85	6,21	12,97	13,60
sbnE	1,55	1,61	1,09	2,36	11,00	6,79	15,95	16,06
sbnF	1,88	4,72	1,83	1,29	21,15	13,20	21,58	22,06
sbnG	0,77	100,00	0,90	0,39	10,81	2,96	12,36	11,97
sbnH	1,41	1,41	1,33	0,42	9,39	7,90	9,98	10,31
sbnI	1,44	1,31	1,31	1,05	8,63	6,80	9,67	9,54
Q5HJP3	100,00	0,60	100,00	100,00	11,97	100,00	16,08	16,08

Gene	vs. zoo28	vs. EMCR19	vs. SS60	vs. SS90	vs. NCTC13712	vs. MSHR1132	vs MW2	vs. SA17_S6
butA	0,13	0,51	0,13	0,39	3,60	2,45	9,78	9,52
galE	2,04	1,94	1,94	1,83	3,26	3,77	12,54	12,84
wcaJ-tuaA	0,43	0,43	0,43	0,87	5,77	4,04	15,58	15,58
epsF	1,19	1,11	2,21	1,19	11,99	5,61	19,64	20,58
wzy	0,89	1,37	0,81	0,89	5,17	3,71	11,70	17,19
wzx	0,91	1,40	2,24	0,70	8,87	5,38	17,61	17,54
sodA-L1	2,49	5,80	2,32	2,49	5,80	4,98	6,14	6,30
sasD	0,83	2,62	1,52	2,20	5,65	4,41	23,14	22,45
Q5HJN3	0,79	0,93	1,19	0,53	6,35	6,88	13,36	13,49
deoD-L1	0,99	2,97	1,27	1,55	4,24	3,25	8,62	8,05
tet38	1,18	5,68	1,33	0,96	7,45	6,42	7,60	7,52
deoC-L1	0,60	3,31	100,00	2,86	100,00	100,00	7,97	8,27
deoB	0,85	1,36	0,68	0,59	7,21	4,07	7,12	7,12
phnE2	1,35	0,74	1,10	1,72	6,99	6,99	6,99	6,86
phnE1	1,75	1,50	2,00	2,00	9,24	5,62	10,36	10,99
phnC	1,16	0,52	1,03	1,03	5,68	5,30	13,18	12,27
phnB	1,46	0,73	2,09	1,57	9,40	4,91	8,57	8,46
Q5HJM4	2,91	4,72	3,29	2,97	9,30	7,43	12,02	11,69
cpdB	1,24	2,28	2,99	1,24	9,83	7,94	10,29	9,90
Q1Y4B9	0,34	100,00	100,00	0,00	100,00	4,25	4,00	21,68
adhE	6,86	6,93	6,86	9,08	12,41	9,39	16,09	15,90
capA-L1	1,94	1,64	1,20	1,35	5,08	5,38	10,91	11,21
capB-L1	0,44	1,46	0,58	0,15	4,37	6,70	7,86	10,48
capC-L1	1,18	2,48	1,31	1,44	8,63	4,05	12,68	12,55
capD	2,14	1,86	1,15	1,32	5,70	5,21	13,10	13,27
capE	1,65	2,14	0,87	1,26	9,14	3,69	13,12	13,80
capF	1,62	1,08	0,72	1,35	2,43	8,72	14,38	14,38
capG	2,40	1,78	1,96	1,96	6,31	5,78	12,09	12,00
capH	1,28	2,29	1,28	100,00	100,00	3,84	15,63	100,00
capI	2,18	2,75	2,18	100,00	100,00	5,07	16,77	100,00
capJ	0,43	0,51	0,43	100,00	100,00	1,53	6,91	100,00
capK	2,88	3,50	2,96	100,00	100,00	6,31	16,20	100,00
capL	5,80	5,56	6,30	4,15	9,78	8,21	13,02	12,69
capM	1,79	1,97	1,25	0,54	9,14	4,12	10,39	10,57
capN	4,28	2,48	4,84	3,60	14,53	6,76	14,75	14,19
capO	3,69	2,91	1,34	2,52	6,37	5,74	13,99	13,92
capP	2,04	2,30	0,34	1,87	4,59	5,95	16,07	16,16
isdI	1,53	1,22	0,92	1,22	3,06	3,67	11,01	11,01
ybaN	0,52	1,56	1,56	2,08	3,91	4,69	11,72	11,46
aldA1	1,14	100,00	3,36	2,49	7,80	4,37	7,39	7,46
czcD	16,16	15,09	17,14	15,77	24,44	18,50	25,22	25,90
Q5HJK0-srpF	1,17	1,17	1,17	1,17	7,80	2,34	8,38	8,77
tauB	2,02	2,97	1,89	2,16	18,62	5,67	17,81	100,00
tauA	1,32	2,23	1,42	1,52	14,49	8,41	13,98	14,99
tauC	2,49	2,49	1,71	2,10	15,88	5,38	15,75	15,88
Q5HJJ8	2,10	2,83	1,92	100,00	11,88	11,52	19,93	21,02
lmrP	0,64	2,40	0,80	100,00	6,23	6,95	8,15	100,00
ausA	1,02	0,92	0,79	0,83	3,01	3,16	11,80	11,76
ausB	0,47	0,93	0,31	0,47	2,48	3,10	12,25	12,09
Q5HJJ2	100,00	1,12	100,00	100,00	3,17	2,80	11,57	11,19
argB	1,81	1,42	0,90	1,68	5,17	5,68	24,42	24,81
argJ	0,81	1,29	0,72	0,40	4,59	4,99	16,59	16,59
argC	1,93	2,71	1,26	1,64	5,51	6,28	12,85	12,75
rocD1	3,04	3,21	2,28	3,29	6,50	9,70	14,85	14,85
brnQ1	1,33	1,33	1,62	0,22	6,12	3,76	11,80	12,32
ywoC2	0,90	2,69	1,25	1,08	9,68	20,07	24,37	100,00
ipdC	0,85	3,66	1,52	1,04	7,01	5,97	9,69	10,54
glcA-ptsG	0,73	1,66	0,59	0,64	4,74	5,23	5,77	6,55
DUF871	1,22	1,78	0,28	0,28	14,73	6,94	16,70	16,98
murQ	0,78	1,56	0,67	0,67	9,00	4,11	14,00	14,22
Q5HJI0	2,20	2,68	2,34	2,47	4,60	3,44	11,00	11,07
rpiRB	0,68	2,84	0,91	0,91	5,35	4,10	10,69	10,81
hsdR	1,18	1,22	1,29	1,36	4,59	4,27	9,82	9,61
Q9RL82	2,80	1,08	100,00	100,00	4,09	100,00	100,00	2,48
oppF2	0,82	1,69	1,00	0,94	12,74	11,68	13,06	12,93
oppB2	12,37	15,89	12,24	12,51	23,53	8,32	12,04	23,12
oppC2	1,29	3,01	1,46	1,63	12,37	9,36	12,54	12,71
oppA2	2,25	3,21	3,10	1,86	8,22	8,00	8,56	8,45
ggt	3,12	2,03	1,58	2,23	6,58	8,80	10,48	11,97
acpD	1,12	1,59	0,96	0,64	6,86	7,18	7,18	7,66
Q5HJG7	1,73	0,69	0,86	1,21	10,02	9,84	10,36	10,71
malK-ugpC	0,73	1,91	0,55	1,46	7,29	6,38	9,38	9,56
malE	1,73	100,00	0,79	1,10	5,82	5,50	9,83	9,83
malC	0,55	0,32	0,16	0,16	4,73	3,70	11,35	11,19
malD	1,43	2,02	1,55	100,00	4,40	4,40	11,67	12,02
yrbE	2,04	3,16	0,93	0,56	3,81	5,39	13,00	100,00
mviM	3,27	4,80	4,90	2,69	4,13	5,48	13,74	14,79
iolE	1,03	1,24	1,34	1,14	6,09	7,12	9,70	9,49
Q7A1X1	0,61	1,41	2,63	1,21	6,46	3,43	13,13	100,00
uhpT	0,65	1,09	0,65	0,29	3,48	3,55	9,28	8,91
yesN	1,71	1,98	1,32	0,40	8,43	6,72	14,23	14,23
yesM	1,09	1,09	0,77	1,03	6,42	5,97	17,79	18,24
hptA	1,65	1,96	1,86	1,24	6,91	5,47	16,72	16,51
pflB	0,27	0,76	0,27	0,36	3,60	2,76	3,69	4,22
pflA	0,40	0,40	0,13	0,40	4,76	1,59	6,61	6,22
Q2YV51	0,64	1,28	0,32	0,32	28,43	100,00	14,70	100,00
glpQ1	0,74	0,79	0,57	0,68	16,84	10,60	12,70	12,87
Q2YV49	1,15	100,00	100,00	100,00	19,60	4,61	19,88	19,31
coa	15,65	18,13	100,00	9,02	100,00	100,00	27,52	100,00
fadA	0,76	2,03	0,51	0,76	5,82	3,54	19,32	19,32
fadB	1,59	2,79	1,28	1,50	6,32	5,00	13,93	13,97

Gene	vs. zoo28	vs. EMCR19	vs. SS60	vs. SS90	vs. NCTC13712	vs. MSHR1132	vs MW2	vs. SA17_S6
fadD	3,38	5,20	1,07	0,83	10,97	4,87	12,46	12,79
fadE	1,19	1,86	1,72	0,86	11,20	5,04	13,72	13,52
fadX	1,96	1,71	2,09	1,27	10,52	4,75	17,24	16,92
prsW-prsS	2,24	100,00	1,70	1,78	22,45	7,12	23,76	23,45
nikA	1,22	1,22	1,96	1,22	100,00	4,87	14,20	14,94
DUF488	4,52	100,00	100,00	1,51	21,11	20,10	27,64	100,00
Q5HJD9	2,87	4,60	4,02	4,60	8,62	9,20	16,09	17,82
hmp	2,09	3,93	2,88	2,53	6,37	8,20	7,42	100,00
lctE	0,63	0,73	0,94	0,52	4,93	2,31	6,60	6,60
ptsIIBC	1,50	2,22	1,31	1,83	5,56	4,71	14,58	14,97
rihA	2,35	2,14	1,28	1,60	6,52	13,03	15,38	15,49
bglG	1,24	1,91	2,00	1,53	4,72	12,96	17,73	18,30
Q5HJD2	0,43	0,43	100,00	1,28	4,49	4,91	13,89	13,89
Q5HJD1-gatB1	0,72	0,36	0,36	1,08	2,51	2,15	8,24	8,60
Q2G2C8-gatC1	0,95	1,59	1,03	1,83	4,76	5,32	9,21	9,37
gutB	0,85	1,04	0,76	0,85	6,34	6,53	14,39	14,96
Q5HJC7	1,36	0,68	1,36	0,68	4,76	3,40	8,84	7,48
Q5HJC6	3,26	3,07	2,59	2,20	4,89	6,03	18,58	18,97
tarI1	1,39	0,70	0,28	1,67	3,49	5,30	13,67	13,39
tarJ1	1,17	0,58	0,88	2,14	5,56	4,19	13,45	12,96
tarL1	1,36	2,77	1,83	2,83	5,60	6,14	12,98	13,45
tarF	1,54	2,39	1,97	2,05	5,04	5,47	15,56	15,13
tarJ2	1,07	1,46	0,39	1,07	2,73	2,83	9,94	10,14
tarL2	0,59	1,07	1,07	0,18	2,55	3,73	5,98	5,74
tarS	1,51	1,27	2,32	1,27	4,52	4,35	10,08	7,36
lytS	2,85	11,23	2,45	2,39	5,53	5,87	11,74	11,05
lytR	1,21	7,29	1,08	1,62	7,56	3,24	10,93	11,07
lrgA	0,90	1,80	0,90	0,90	6,98	5,18	4,95	5,18
lrgB	0,28	2,71	0,14	0,57	7,41	2,28	8,69	8,12
Q5HJB2	4,96	0,85	1,84	1,42	3,97	16,31	18,30	18,72
Q1YAD5-ptsG3	1,37	2,61	2,48	1,99	10,56	11,68	28,07	27,33
bglA	0,89	100,00	100,00	100,00	6,84	14,37	16,63	16,02
rbsK	2,18	100,00	1,96	2,40	6,21	4,14	18,08	17,21
rbsD	2,22	0,25	1,48	1,73	2,72	3,70	13,83	12,84
rbsU	2,15	2,15	2,04	1,70	4,65	4,20	12,13	11,22
rbsR	1,30	1,70	1,60	0,80	5,91	8,21	18,82	100,00
yusP	2,53	2,75	1,23	2,31	6,07	7,16	14,03	13,81
yxeI	1,11	100,00	1,11	1,11	5,84	5,33	15,90	100,00
lytM	1,77	2,28	1,56	2,18	5,92	6,44	9,14	9,24
Q5HJ98-ybhF	1,82	1,67	1,67	100,00	10,15	6,82	13,48	12,88
Q5HJ97	0,65	1,30	0,98	0,98	12,27	7,06	14,88	15,20
Q5HJ96	1,29	0,94	1,20	1,11	10,03	6,86	12,60	12,08
Q5HJ95	0,52	1,77	0,85	0,92	7,94	3,67	8,33	8,86
ssaA	1,44	1,00	1,00	0,89	6,20	4,32	13,73	13,73
esxA	0,00	0,68	0,34	0,34	0,34	0,68	3,06	3,40
esaA	1,42	1,32	1,52	1,16	4,52	3,96	7,69	8,05
essA	0,65	0,65	0,65	0,87	1,53	0,87	3,05	3,27
esaB	2,88	0,82	1,65	2,88	4,12	7,41	7,00	7,82
essB	2,40	1,95	2,17	5,24	4,94	4,49	5,17	5,77
essC	4,43	1,35	1,08	5,06	2,09	5,42	4,88	5,24
esxB	100,00	0,00	0,00	100,00	0,00	100,00	100,00	100,00
Q1Y4S0	0,80	1,60	1,07	1,33	6,93	1,87	2,40	2,67
Q2G173	1,00	4,73	1,00	1,00	11,94	4,73	0,50	1,74
focA-L1-nirC	0,73	0,73	0,24	0,48	4,36	3,88	0,85	4,97
brnQ2	1,22	1,30	1,22	100,00	6,88	6,96	2,83	7,95
Q99WS0-sapS	1,23	1,46	1,23	1,01	6,06	5,05	4,83	6,17
Q2G168	1,43	1,62	1,62	1,14	8,38	6,19	9,05	9,14
A5IPH6	1,03	0,44	0,74	0,88	11,06	9,44	11,80	11,65
pfoR	0,87	1,07	0,58	0,78	10,27	5,52	9,59	9,59
Q5HJ57	0,36	0,80	0,27	0,54	9,29	4,47	9,29	9,20
psuG	1,08	2,06	1,62	100,00	7,90	6,17	8,23	8,66
nupC2	1,47	1,56	1,56	1,31	9,25	5,98	7,78	100,00
nanT	1,17	2,28	0,85	1,17	3,46	5,28	9,00	10,83
nanA	1,36	1,59	0,91	1,93	10,66	5,56	10,66	11,00
nanK	0,58	0,58	0,70	1,16	17,89	8,59	17,07	17,65
nanR	1,00	0,37	1,12	0,75	15,61	10,36	15,98	16,35
nanE	0,89	100,00	1,04	0,89	12,93	7,88	14,12	13,67
Q5HJ49	1,52	1,37	1,52	1,52	14,16	7,00	18,95	100,00
lip2	3,14	3,57	2,24	2,48	17,25	6,53	21,68	21,92
A5IPQ2	1,69	1,93	1,57	1,69	18,24	4,71	17,15	100,00
Q6GJZ4	1,56	1,15	100,00	100,00	100,00	6,73	15,11	100,00
limB1	1,60	2,69	1,50	100,00	18,36	7,29	16,67	17,37
gcvH1	2,10	100,00	1,50	1,50	13,51	3,60	12,01	12,31
UPF0189-ymdB	1,99	2,86	2,36	3,11	17,16	10,82	16,92	17,29
sir2	1,24	1,24	1,35	1,97	21,84	11,39	15,42	15,42
lplA1	1,47	2,35	2,15	2,15	8,41	5,57	10,17	10,85
ulaA	0,89	1,19	0,45	1,19	9,21	4,75	11,51	11,36
Q5HIW4	3,16	1,75	0,70	1,75	9,12	1,75	10,53	10,53
Q5HIW3	2,25	0,90	100,00	0,90	13,06	3,60	12,39	13,29
A8YZF7-licR2	1,48	2,15	1,53	1,64	19,58	4,91	19,68	19,89
mepR	1,19	1,19	0,71	0,00	13,10	2,38	14,29	14,52
mepA	0,59	1,40	0,37	1,03	11,14	3,32	11,73	12,46
mepB	2,04	1,81	2,49	2,95	25,17	8,16	23,36	100,00
glpT	1,03	1,62	1,25	1,47	10,38	5,00	10,01	10,82
mhqA-1	1,62	9,91	1,08	1,19	20,26	12,07	26,83	100,00
ssuD-luxA	1,51	3,01	1,03	1,69	11,48	5,08	11,48	11,01
ssuE	1,94	2,12	2,12	1,94	9,70	6,00	8,82	8,99
yeiH	0,40	2,81	0,80	0,50	10,64	100,00	10,94	11,04
rimL-ydaF	0,18	1,83	0,73	1,65	8,61	8,79	8,24	100,00
fepA	1,52	1,64	1,87	1,29	10,29	5,61	8,07	7,95
fepB	4,96	10,89	3,41	3,33	16,49	10,15	14,70	100,00

Gene	vs. zoo28	vs. EMCR19	vs. SS60	vs. SS90	vs. NCTC13712	vs. MSHR1132	vs MW2	vs. SA17_S6
fepC	100,00	100,00	100,00	100,00	100,00	100,00	100,00	100,00
tatC	100,00	0,00	100,00	100,00	100,00	100,00	100,00	100,00
tatA	100,00	0,00	100,00	0,00	100,00	0,00	100,00	100,00
DUF1398	100,00	0,26	0,26	1,03	10,51	8,21	3,33	10,51
Q5HIU5	0,00	0,00	0,00	0,98	100,00	1,47	5,88	5,88
DUF3169	1,68	2,52	1,40	1,12	8,12	4,90	5,74	7,84
Q5HIU3	1,66	2,49	100,00	1,07	100,00	8,53	4,15	4,38
Q5HIU2	2,06	4,60	2,22	2,70	4,92	4,92	5,56	5,08
ltrA-L1	2,64	2,73	0,79	1,41	9,60	7,92	10,39	100,00
thlA-yqil	0,68	2,37	0,51	0,76	11,51	5,16	11,68	12,01
mdh	0,40	1,46	0,80	1,20	9,83	4,38	9,30	9,16
metE	0,31	2,92	0,54	0,31	8,61	4,49	8,30	8,08
metF	0,76	1,03	1,14	0,27	8,85	3,96	9,34	9,01
metC	1,03	1,64	0,86	1,12	12,32	5,68	12,58	12,58
metI	1,09	1,81	1,81	1,99	9,60	6,16	9,78	9,51
spo0J	0,24	6,86	0,00	0,35	15,60	5,20	13,48	14,66
ykuT	0,23	0,23	0,23	0,23	8,28	8,39	1,25	14,74
yyzM	0,98	0,49	0,49	0,49	6,86	0,98	0,98	5,39
yyaF	0,82	0,46	0,46	0,91	6,19	3,28	4,46	7,74
Q5HIT0	1,04	100,00	0,52	0,52	6,25	6,25	3,13	4,17
rpsF	0,00	0,00	0,00	0,00	0,34	6,40	6,73	0,00
ssb	0,20	0,79	0,40	0,20	0,99	1,59	0,79	0,60
rpsR	0,00	0,00	0,00	0,00	0,00	0,00	0,00	0,41
setC	2,94	1,14	100,00	2,12	4,24	6,04	4,89	100,00
Q2G106	2,23	1,98	3,96	2,97	100,00	17,82	13,61	100,00
Q1Y9M1	1,40	1,57	1,40	1,40	4,71	4,01	6,28	4,89
Q5HIS2	1,89	1,52	1,52	2,27	4,17	3,41	3,79	3,03
Q5HIS1	5,53	1,19	5,14	100,00	5,53	6,72	4,35	7,91
Q7A7J4	1,03	2,06	1,03	1,55	6,19	6,19	12,03	13,23
Q5HIR9	0,52	0,52	0,26	0,26	7,03	10,16	7,55	8,33
A5IPV8	1,44	6,70	1,59	100,00	8,77	9,73	8,61	8,61
Q6GJR9	100,00	0,31	100,00	0,12	100,00	100,00	100,00	100,00
ahpF	1,44	0,79	0,85	0,79	6,63	4,72	5,64	5,91
ahpC	0,35	0,53	0,35	0,35	3,33	2,28	4,91	4,74
nfrA	1,46	5,03	1,46	1,46	12,04	8,99	13,23	13,36
tcyP	1,94	1,37	1,01	0,72	8,78	6,26	13,97	13,89
Q5HIR2	1,78	1,36	1,36	1,25	7,00	8,78	16,51	16,51
Q5HIR1	0,30	0,45	1,36	1,36	5,28	6,94	12,97	12,67
yflT	0,74	0,25	0,25	0,49	9,07	7,84	10,54	100,00
xpt	0,52	0,52	0,69	0,69	14,51	7,08	8,81	8,81
pbuX	0,71	1,02	0,63	0,63	12,61	5,59	8,67	8,90
guaB	2,66	2,25	1,30	2,32	3,54	2,66	1,70	2,11
guaA	1,43	1,69	1,69	0,78	2,33	3,18	2,40	2,66
Q2YVN4	100,00	100,00	2,04	1,53	3,24	3,92	100,00	100,00
Q5HIQ1	2,78	1,67	1,39	0,83	12,78	7,50	10,56	100,00
Q2G0Y0	1,77	4,49	1,54	1,89	10,76	7,21	10,40	11,47
ssl01	100,00	100,00	100,00	100,00	100,00	100,00	100,00	100,00
ssl02	100,00	0,00	100,00	100,00	100,00	100,00	100,00	0,00
ssl03	100,00	100,00	100,00	100,00	0,00	0,00	100,00	0,00
ssl04	100,00	100,00	100,00	100,00	0,00	100,00	100,00	0,00
ssl05	100,00	100,00	100,00	100,00	100,00	100,00	100,00	0,00
ssl06	100,00	0,00	0,00	0,00	0,00	0,00	100,00	0,00
ssl07	100,00	100,00	100,00	100,00	0,00	100,00	100,00	0,00
ssl08	100,00	100,00	100,00	100,00	100,00	0,00	100,00	0,00
ssl09	100,00	100,00	100,00	100,00	0,00	0,00	100,00	100,00
ssl10	100,00	100,00	100,00	100,00	100,00	100,00	100,00	0,00
hsdM-ssl	100,00	100,00	0,00	0,00	0,00	100,00	100,00	100,00
hsdS-ssl	100,00	100,00	0,00	0,00	0,00	0,00	100,00	0,00
ssl11	100,00	100,00	100,00	0,00	100,00	100,00	100,00	100,00
Q5HIM7	0,00	0,00	100,00	100,00	100,00	100,00	100,00	100,00
cobW1	3,33	100,00	2,66	2,49	4,99	4,82	4,32	4,49
psmA4	0,00	1,59	0,00	0,00	3,17	1,59	3,17	3,17
psmA3	0,00	4,35	0,00	1,45	5,80	2,90	7,25	7,25
psmA2	1,52	0,00	0,00	1,52	0,00	0,00	0,00	0,00
psmA1	0,00	1,52	0,00	0,00	3,03	0,00	0,00	0,00
mpsA-nuoL	2,36	2,29	2,29	2,29	9,63	6,20	10,51	9,97
mpsB-ybcC	1,95	2,21	2,39	2,58	11,89	9,86	14,94	14,46
mpsC-ybcI	1,65	2,48	1,38	1,93	9,92	5,51	8,54	8,54
Q5HIM2	100,00	0,80	0,80	1,06	100,00	3,71	15,38	15,38
G7ZRU6	0,00	0,00	0,00	0,00	0,00	100,00	0,00	0,00
Q5HIM1	1,04	3,26	1,04	1,93	8,59	7,85	11,41	12,00
Q5HIM0-est1	0,82	1,49	1,36	0,95	100,00	5,57	13,32	100,00
Q5HIL8	1,87	2,02	100,00	2,17	5,98	5,31	14,50	14,13
mccA-var915	0,98	2,18	1,20	2,07	7,97	7,97	12,88	100,00
mccB	0,35	0,61	0,26	0,35	5,60	5,07	9,71	9,62
metN2	1,36	0,88	1,36	1,17	3,80	6,92	14,62	15,40
metP2	0,30	1,36	0,30	0,45	5,30	5,15	13,48	13,48
metQ2	1,19	1,66	1,07	1,30	3,56	7,12	15,54	15,78
aaa	0,10	0,59	0,40	0,49	5,14	4,05	6,32	6,03
Q5HIL1	1,11	0,74	0,00	1,48	4,81	3,70	14,81	14,81
nudG	1,00	0,75	1,00	0,25	4,01	4,51	16,54	16,79
bltD	1,23	2,46	100,00	1,03	8,42	8,62	22,38	100,00
yibF	1,40	1,53	1,66	0,77	4,34	3,83	14,94	14,69
yibE	1,16	1,34	0,80	1,07	5,63	4,20	13,32	13,23
gltC	1,13	0,79	0,34	0,90	4,52	4,18	15,59	15,93
gltB	0,98	1,22	0,73	1,20	5,00	4,60	8,87	8,78
gltD	1,23	1,43	1,23	1,37	7,10	4,92	10,04	10,04
treP	1,61	1,75	1,68	1,26	4,06	7,07	12,68	100,00
treC	1,76	100,00	100,00	100,00	5,78	6,81	14,05	100,00
treR	0,82	100,00	0,82	0,82	6,99	7,53	16,03	100,00
Q5HIK0	1,90	0,95	0,38	0,57	8,38	6,29	14,29	100,00

Gene	vs. zoo28	vs. EMCR19	vs. SS60	vs. SS90	vs. NCTC13712	vs. MSHR1132	vs MW2	vs. SA17_S6
dnaX	0,59	1,24	0,88	0,77	4,48	4,89	12,49	100,00
yaaK	0,63	0,63	0,31	0,94	2,83	1,89	1,89	100,00
recR	1,01	1,17	0,84	1,17	3,02	2,01	9,55	100,00
yaaO	5,31	7,85	6,13	5,16	12,26	11,58	13,30	13,45
tmk	1,29	1,29	0,97	1,78	9,71	4,21	10,03	9,71
darA	0,30	0,00	0,30	0,30	2,73	1,21	4,85	5,15
holB	0,86	0,54	0,43	0,32	3,34	3,13	9,60	9,60
yaaT-tpl	1,87	1,24	0,75	0,62	4,98	8,46	12,31	12,44
yabA	2,59	3,16	0,00	0,29	5,17	7,76	15,23	15,52
yabB	4,13	1,38	0,69	1,24	4,41	4,55	14,33	13,91
yazA	0,40	0,00	0,40	0,40	2,01	1,20	9,64	9,64
rsmI	0,83	100,00	0,83	0,71	5,12	6,31	16,79	16,67
metS	0,61	1,87	0,35	1,52	5,52	5,02	9,68	9,52
tatP	0,78	1,55	1,03	1,03	5,17	5,56	12,27	12,27
rnmV	1,49	100,00	1,30	0,93	3,54	4,47	13,04	13,22
ksgA-rsmA	0,44	1,10	0,11	1,21	8,11	4,50	14,14	14,14
veg	0,38	0,38	1,52	0,00	0,38	0,38	1,89	1,52
ispE	0,47	0,94	1,30	0,71	2,94	2,47	7,54	7,89
purR	0,24	0,97	1,33	0,24	1,45	1,82	5,70	5,45
yabJ	0,52	2,10	1,84	1,05	2,89	4,46	10,76	10,50
spoVG	0,00	0,33	0,33	0,00	1,65	1,98	3,96	4,29
glmU	0,81	1,03	2,44	0,52	3,70	6,73	13,60	13,60
prs	0,21	0,00	2,28	0,21	2,07	1,97	5,38	5,49
rplY	0,15	0,46	0,31	0,15	1,22	1,07	1,68	1,68
pth	0,35	0,52	0,35	0,52	3,14	5,58	10,30	10,47
mfd	1,23	1,48	1,03	1,23	5,30	4,99	13,09	13,32
yabM	1,64	1,51	1,11	1,57	5,76	6,42	17,55	17,55
mazG	1,84	2,09	1,93	1,59	7,20	6,03	18,51	100,00
hslR	0,38	0,38	0,76	0,00	100,00	6,44	15,53	16,67
divIC	0,00	0,76	0,76	0,25	2,80	3,56	12,98	12,98
yabR	0,00	0,00	0,00	0,00	1,00	1,00	4,48	4,48
tilS	1,47	100,00	1,47	1,47	4,71	4,94	17,98	17,98
hpt	0,93	0,37	0,74	0,56	2,59	2,96	9,07	9,26
ftsH	0,81	0,72	0,48	0,38	2,72	3,49	7,64	7,74
hslO	0,79	0,91	0,79	1,02	5,56	5,33	10,54	10,77
cysK	1,39	0,54	1,18	1,07	2,57	3,32	7,72	7,72
folP	1,37	1,87	1,00	1,00	4,98	5,47	14,55	14,93
folB	0,27	1,09	0,27	0,27	4,64	4,37	10,11	10,11
folK	1,67	1,46	0,42	1,25	23,33	6,04	22,71	21,88
lysS_leader	0,00	0,00	0,00	0,00	100,00	100,00	100,00	100,00
lysS	0,81	0,81	0,20	0,34	10,55	6,92	11,22	11,49
pdxR	1,37	1,52	1,08	1,45	19,31	2,53	8,68	9,18
pdxS	1,91	3,60	0,45	1,80	6,87	5,97	7,55	8,00
pdxT	0,89	100,00	0,36	100,00	4,27	5,69	13,70	13,88
nupC1	0,66	0,41	0,74	0,58	4,28	4,44	8,48	9,05
ctsR	2,12	2,12	1,91	2,34	1,70	1,49	8,07	8,07
mcsA	0,88	0,35	0,53	0,71	4,76	4,06	13,05	13,23
mcsB	0,59	0,69	1,19	0,30	2,97	2,67	11,08	11,18
clpC	0,45	0,90	0,69	0,53	3,74	4,35	10,66	10,79
radA	1,47	0,51	0,81	2,05	4,25	4,03	11,50	11,43
pilT	0,28	0,47	0,19	0,28	100,00	4,56	12,66	12,76
gltX	0,82	1,72	1,10	1,10	3,99	7,08	10,79	10,72
cysE	1,54	1,54	2,16	1,39	3,24	2,93	8,49	8,64
cysS	2,28	2,86	1,71	2,07	5,21	6,71	12,13	11,71
mrnC	0,00	0,00	0,49	0,25	3,21	3,46	13,83	13,58
yacO	0,80	1,20	0,40	0,80	5,49	5,76	12,18	12,45
yacP	0,76	0,76	0,76	0,57	3,81	5,71	14,67	14,67
sigH	0,35	0,35	0,18	0,53	6,49	8,60	25,44	25,44
rpmG3	2,78	0,69	2,78	0,00	1,39	2,78	4,17	5,56
secE	0,55	0,00	0,00	0,00	0,55	0,00	1,09	1,09
nusG	2,91	0,36	0,36	0,36	2,37	2,73	8,01	8,56
rplK	0,47	1,18	0,00	0,00	0,47	0,71	1,42	1,89
rplA	0,14	0,14	0,14	0,00	1,73	1,30	1,88	1,88
rplJ	0,00	0,60	0,00	0,00	1,60	1,80	1,20	1,20
rplL	0,27	0,27	0,27	0,27	0,54	0,81	0,27	0,27
ybxB-rsmC	0,98	100,00	0,98	0,82	100,00	8,52	17,54	17,05
rpoB	0,70	1,01	0,87	0,53	2,90	3,13	6,33	6,33
rpoC	0,50	1,43	0,58	0,50	3,64	2,70	5,38	5,38
ybxF	1,18	1,57	1,18	1,18	100,00	1,96	4,31	4,31
rpsL	0,72	0,24	0,24	0,24	1,21	0,48	2,42	2,66
rpsG	0,00	0,21	0,21	0,21	0,21	0,00	1,27	1,06
efg	0,34	0,58	0,38	0,24	1,87	1,10	1,97	2,02
tuf	0,25	0,42	0,42	0,25	0,84	1,10	1,52	1,86
yhaA-amaA	0,68	1,79	0,60	0,51	100,00	6,63	16,50	16,41
kbl	1,68	2,27	2,10	1,01	4,63	5,47	11,03	11,03
hchA	1,36	1,02	3,18	1,02	4,89	5,68	11,82	100,00
araB	0,55	1,22	0,98	1,28	3,97	8,79	16,18	16,73
Q5HIC2	0,31	100,00	0,62	0,31	7,76	5,38	11,90	12,01
ilvE	0,65	1,39	1,30	1,67	4,64	1,76	10,40	10,68
ppaX1	6,42	1,95	0,70	1,53	6,14	4,88	17,43	17,02
dck	0,90	0,75	0,00	0,75	8,75	4,22	12,37	11,46
dgk	0,65	0,65	1,46	1,13	8,25	6,15	5,99	5,83
tadA	1,06	1,70	1,27	1,06	10,40	4,25	8,70	8,70
A5IQB3	1,84	1,84	0,69	1,26	5,86	8,51	12,76	12,53
azo1	1,59	0,35	2,65	1,23	6,00	6,17	11,11	11,46
sdrC	3,57	28,07	100,00	17,77	20,30	23,14	15,92	100,00
sdrD	0,00	100,00	100,00	100,00	0,00	100,00	100,00	100,00
bbp	0,00	100,00	100,00	100,00	100,00	100,00	100,00	100,00
sdgA	2,04	1,70	2,04	2,38	19,42	7,60	20,30	19,96
sdgB	3,35	2,82	2,48	3,09	16,97	6,04	16,83	17,17
folE2	0,46	0,68	0,34	0,34	9,44	4,10	9,90	9,90

Gene	vs. zoo28	vs. EMCR19	vs. SS60	vs. SS90	vs. NCTC13712	vs. MSHR1132	vs MW2	vs. SA17_S6
bshB2	0,45	2,25	0,75	0,45	9,76	3,30	9,31	9,76
yojF	0,55	100,00	0,55	0,55	7,16	1,65	6,89	6,89
nagB	1,32	7,91	1,45	3,43	12,12	7,91	12,91	12,52
hxlA	0,47	6,64	0,95	1,26	6,00	6,48	9,16	10,27
sis	2,19	2,91	2,00	2,19	17,12	8,38	20,04	20,40
Q2FJ68	0,62	100,00	0,46	1,85	100,00	100,00	14,15	13,69
proP	0,64	1,36	0,64	1,14	4,50	3,78	13,28	100,00
vraA	2,47	100,00	3,12	100,00	27,14	10,16	26,27	26,78
vraB	1,40	1,67	1,67	1,49	18,25	5,96	18,25	18,95
vraC	0,53	3,69	0,53	2,90	100,00	18,47	19,00	19,26
Q5HI98	1,80	100,00	2,88	0,72	16,91	20,50	15,83	14,39
thiD1	2,05	2,29	2,05	2,05	10,83	7,58	10,11	10,23
ung	3,35	2,28	3,20	4,11	8,07	10,05	8,07	8,22
ywdI	1,05	6,04	7,35	0,52	17,32	11,02	17,59	17,06
ywdK	1,63	2,71	2,17	1,08	4,07	3,25	11,11	11,11
yfnA2	0,94	1,28	0,74	0,81	3,70	4,11	9,70	9,43
DUF3815	1,10	0,44	100,00	1,10	6,61	3,96	8,59	100,00
DUF1212_L1	0,78	1,57	0,39	0,26	4,84	5,10	7,71	8,24
hemQ	0,80	0,40	1,86	1,20	9,96	5,05	10,62	10,49
pta	1,11	1,01	1,11	1,72	9,22	7,09	9,12	8,92
lipL	0,72	1,08	0,60	0,60	13,02	8,00	13,74	13,62
mvaK1	0,76	0,87	1,09	1,19	6,73	4,45	7,06	7,06
mvaD	1,63	2,74	1,63	2,34	3,25	6,81	3,35	3,15
mvaK2	1,39	1,30	0,84	0,84	4,46	2,04	5,01	4,83
DUF1450	0,58	0,88	1,17	1,46	3,22	2,05	2,63	2,05
ykgC	1,96	2,64	100,00	2,26	2,94	5,05	3,09	3,31
Q5HI81	0,91	0,45	1,59	0,45	3,40	100,00	2,95	2,72
Q5HI79	9,98	100,00	9,70	8,49	8,14	8,49	1,77	8,78
Q5HI67	1,81	1,38	2,34	2,66	5,64	5,22	5,54	5,64
Q5HI66	1,30	1,86	3,17	2,23	10,99	8,57	10,43	10,80
Q8NXU4	0,63	0,42	0,42	0,21	2,94	6,08	2,94	3,35
Q5HI65	1,62	2,08	2,01	2,08	5,48	5,79	5,63	5,63
ywhD	1,53	1,15	3,64	0,77	4,60	4,79	4,79	4,79
adhA	2,47	3,07	2,77	2,67	5,54	5,74	5,64	5,24
ywiB	0,00	0,47	0,23	0,70	2,33	2,10	2,10	1,86
argS	1,32	1,38	0,48	1,50	5,96	5,35	5,60	6,02
nth2	2,83	3,62	1,10	2,20	7,70	12,42	7,08	8,96
A5IQG3	3,15	0,90	0,79	0,34	9,91	9,68	10,59	10,59
yvrB	2,52	2,63	1,16	3,68	4,73	6,10	21,66	21,66
ppaX2	0,42	0,56	0,42	0,69	4,72	4,03	11,25	11,11
ydjP	1,00	1,12	0,87	3,25	6,99	5,62	17,73	17,35
Q5HI54	0,79	0,79	0,99	1,78	100,00	2,76	7,89	7,89
Q5HI53	2,09	1,95	1,95	1,53	100,00	6,56	15,62	15,76
Q5HI52	0,38	0,63	0,51	0,13	2,66	4,18	15,08	14,83
sarA	0,00	0,00	0,53	0,27	1,07	0,80	4,80	5,07
Q2YSV8	2,79	100,00	100,00	2,79	100,00	6,55	22,53	24,25
A6QET1	2,22	2,67	0,89	2,67	7,56	5,78	16,89	16,89
Q7A1N3	0,98	2,94	0,49	1,47	100,00	5,88	20,59	19,12
A5IQH4	0,71	0,71	0,36	0,71	8,02	8,38	23,17	100,00
mrpA	1,87	1,87	2,08	1,37	5,74	6,62	20,06	19,73
mrpB	2,82	2,35	1,88	2,82	4,46	4,46	15,96	18,78
mrpC	1,16	2,03	1,45	1,16	6,38	5,51	15,36	17,10
mrpD	2,20	2,67	1,67	1,34	5,28	4,81	17,90	17,70
mrpE	0,83	1,24	0,62	0,62	3,11	3,11	10,56	10,97
mrpF	0,33	0,99	0,99	0,99	7,26	5,28	13,53	13,53
mrpG	2,26	4,07	1,36	100,00	8,14	7,69	17,65	18,10
nhaK1	0,64	0,69	0,64	0,39	2,94	2,69	10,38	10,57
mntC	0,86	0,43	0,75	0,75	1,83	1,61	5,70	5,70
mntB	0,24	1,08	0,12	0,12	3,35	3,46	6,33	6,45
mntA	0,27	0,94	0,27	0,27	2,02	1,48	6,59	6,59
mntR	0,16	1,55	0,78	0,31	3,10	2,95	10,39	11,47
Q5HI33	0,53	1,33	0,40	0,93	6,11	5,18	18,46	18,06
tarA	0,78	0,78	0,92	0,92	4,44	4,44	15,69	16,21
tarH	1,89	2,14	100,00	1,76	3,40	5,79	13,33	12,58
tarG	0,72	0,48	0,36	0,72	3,60	2,40	7,07	7,31
tarB	1,09	1,09	1,72	1,27	23,82	7,52	23,37	23,73
tarX	1,03	1,03	1,31	1,40	26,78	7,49	26,87	27,25
tarD	1,50	1,00	0,50	0,25	12,78	3,01	12,78	12,03
pbpD	1,70	0,93	1,54	1,39	9,26	5,02	15,82	15,51
msbA1	1,16	0,87	0,75	1,04	4,63	4,17	11,11	10,94
nupG	1,22	1,63	1,71	1,87	9,02	6,10	9,84	9,84
yxkD	0,36	0,60	2,28	0,36	1,92	3,36	13,79	13,91
fhuC	0,50	0,63	1,38	0,63	2,38	3,26	11,90	11,78
fhuB	1,29	0,60	2,89	1,00	1,99	6,87	16,02	16,12
fhuG	1,87	0,29	1,47	1,47	5,11	7,77	13,86	13,77
dakK	0,52	0,83	0,52	0,52	5,57	4,95	16,10	16,31
dakL	1,20	0,51	1,20	1,03	4,27	4,79	18,12	18,46
dakP	1,38	3,31	3,03	3,86	5,23	5,51	19,01	18,73
Q5HI15	0,60	0,20	0,80	0,40	1,41	1,00	1,81	1,81
Q5HI14	0,19	1,78	0,09	0,28	2,90	4,12	9,46	8,71
lip4	1,44	2,11	0,86	1,15	6,03	4,79	17,05	16,48
Q5HI12	0,93	1,63	1,17	0,70	100,00	5,36	19,35	19,58
Q5HI11	0,79	0,59	0,39	0,00	4,14	3,94	13,02	13,41
graX	0,11	1,41	0,32	0,43	5,41	4,00	17,64	17,32
graR	0,44	1,04	0,44	0,74	2,81	4,00	10,96	10,96
graS	1,34	0,96	0,77	1,15	3,36	2,69	13,74	13,74
vraF	1,18	0,92	0,52	0,66	6,69	6,43	14,04	14,83
vraG	0,37	0,90	1,80	0,69	100,00	6,61	15,81	100,00
ykaA-pitR	0,00	0,00	0,16	0,32	1,62	2,10	4,05	3,72
pitA	0,40	0,30	0,10	0,50	1,88	2,08	10,62	9,42
ssaA5	0,63	0,63	0,50	0,63	3,13	2,88	9,02	9,52

Gene	vs. zoo28	vs. EMCR19	vs. SS60	vs. SS90	vs. NCTC13712	vs. MSHR1132	vs MW2	vs. SA17_S6
yetJ	0,31	0,46	0,31	0,31	3,67	2,75	20,18	100,00
rbf	0,33	0,60	0,56	0,51	4,88	4,42	16,13	15,81
sarX	0,47	0,47	0,00	0,00	2,35	2,11	7,75	7,75
yeeI	0,70	0,98	0,84	0,70	3,77	4,60	12,83	13,39
DUF985	2,29	2,91	2,49	1,46	7,07	5,20	20,58	100,00
A5IQM5	0,15	1,23	0,46	0,61	2,92	3,69	9,98	9,98
ccpE	3,14	3,82	3,03	3,14	3,25	3,93	12,68	13,02
A8Z196	3,69	0,90	1,47	0,49	5,90	6,55	13,43	13,02
Q2YSN2	0,61	0,41	0,61	0,41	100,00	7,16	15,54	15,54
Q2YSQ6	0,87	1,01	1,01	0,87	6,52	5,80	13,77	13,77
A5IQN0	5,84	5,39	5,39	2,47	8,54	8,99	28,31	100,00
A8YZU8	0,00	0,00	5,21	0,00	5,46	6,70	22,08	100,00
Q5HHZ0	0,33	0,33	0,67	0,33	2,67	4,00	12,33	12,67
ykkB	2,21	2,58	2,21	1,84	7,55	7,00	19,89	20,99
yvdD	1,06	1,06	1,06	0,88	3,88	5,29	13,23	13,76
yqxD	0,00	0,65	1,31	0,87	5,66	4,58	17,21	17,43
Q5HHY6	1,17	1,02	0,88	0,44	100,00	4,68	12,13	12,28
uppP	0,23	0,11	0,57	0,11	3,42	3,31	12,21	12,10
cydD	0,61	0,98	0,67	0,98	7,60	6,86	19,24	19,18
cydC	1,19	2,20	1,13	1,73	12,09	6,79	20,55	20,61
mgrA	0,00	0,00	0,00	0,00	1,35	1,80	1,58	1,35
cobW2	1,70	1,27	4,03	1,80	6,90	8,28	20,81	20,49
ycsN	1,10	1,87	1,98	1,43	6,05	6,38	17,82	17,38
Q5HHX9	1,04	0,69	0,35	0,00	6,25	5,56	15,97	15,63
yflS	2,32	1,67	1,29	1,03	5,92	5,34	18,02	17,37
phrB	1,16	2,11	1,02	1,16	5,46	6,62	21,11	100,00
Q2G0A5	2,09	1,39	100,00	3,48	100,00	100,00	25,44	25,78
Q5HHX6	3,72	1,29	0,97	1,29	2,91	4,37	17,15	17,48
Q5HHX5	1,65	2,84	1,65	0,47	6,15	6,86	23,88	23,40
norA	0,09	100,00	0,09	0,43	5,22	4,71	17,47	17,04
yedL	0,88	1,97	0,88	0,88	7,68	7,46	27,85	28,51
ybaK	1,45	1,24	1,66	1,24	1,24	7,25	20,70	21,12
fruR	1,57	0,78	1,05	1,83	5,23	5,10	12,94	12,94
fruB	0,65	0,54	0,65	0,76	4,13	4,13	13,03	12,92
fruA	1,02	1,42	2,09	1,68	8,96	4,89	12,67	11,86
nagA	1,18	2,88	0,76	1,02	7,45	14,21	15,40	15,23
corC-mpfA	0,07	1,85	0,15	0,15	2,96	3,78	10,37	10,37
yvgN1	1,07	1,07	0,83	1,19	7,50	5,60	16,90	17,14
csbB	0,71	0,61	0,50	0,50	4,64	3,83	14,63	100,00
saeS	0,38	0,85	0,38	0,38	1,23	1,70	7,10	7,29
saeR	0,29	0,58	0,29	0,15	2,47	1,75	8,44	8,44
saeQ	0,21	0,84	0,63	0,42	100,00	1,48	6,96	6,75
saeP	0,91	0,91	0,68	0,68	3,63	2,95	6,80	6,58
Q5HHW1	0,51	0,51	0,34	0,51	3,59	3,42	12,48	12,31
queE	1,26	1,54	0,56	1,40	5,18	5,32	15,13	14,85
queD	0,48	0,24	100,00	0,24	5,48	4,52	13,10	13,10
queC	0,00	0,15	0,15	0,00	3,59	3,89	11,21	11,51
pabA	0,51	1,52	0,34	0,51	5,39	6,06	24,58	24,24
pabB	0,69	100,00	0,78	0,78	9,54	9,54	25,07	100,00
pabC	1,15	1,31	0,99	0,82	10,51	7,55	25,45	25,45
Q6GIS6	0,00	0,48	1,45	0,97	3,86	1,93	23,67	23,19
ahs1	0,28	2,11	0,14	0,56	3,80	4,22	14,77	14,63
ahs2	1,99	2,19	1,69	1,79	4,58	6,77	15,82	15,82
ltaS	0,21	0,52	0,21	0,21	2,78	2,16	8,66	8,29
uup?	1,22	2,12	1,43	1,59	4,25	4,94	15,07	15,23
recQ1	0,39	1,18	0,34	0,45	4,26	3,98	11,56	11,62
opuBA	0,41	1,33	1,23	1,53	4,29	3,27	12,99	13,80
opuBB	0,92	0,92	0,59	1,25	4,49	4,29	13,33	13,14
hisC2	0,66	0,85	0,66	100,00	5,76	5,57	15,30	15,39
yorS	1,84	2,39	1,10	0,74	5,16	5,34	12,15	11,60
bmrU-dgkA1	0,87	1,96	0,65	0,65	6,64	8,93	11,76	11,22
dtpT	0,53	0,80	0,60	0,66	3,92	5,18	9,43	9,69
queF	0,60	1,00	0,00	0,00	3,59	4,59	11,38	11,58
yxxF	0,34	0,34	0,46	0,46	2,64	3,68	10,57	10,23
nrdI	0,25	0,25	0,25	0,25	1,00	0,50	2,51	2,76
nrdE	0,43	0,62	0,33	0,28	2,52	2,52	8,83	8,50
nrdF	0,41	0,82	0,10	0,51	3,29	3,40	9,77	8,85
sstA	1,13	0,82	0,31	0,41	3,19	2,67	12,65	12,24
sstB	1,25	1,99	1,36	1,15	5,12	5,75	17,24	17,03
sstC	0,52	100,00	1,05	0,52	3,41	4,20	16,14	16,27
sstD	1,07	3,40	0,58	0,39	3,79	3,98	9,14	9,33
Q5HHT3	0,63	0,63	0,63	0,63	2,22	1,59	13,65	14,29
murB	0,76	0,97	1,30	0,43	4,98	4,22	13,85	14,18
grpB	2,12	0,96	2,31	1,73	5,78	5,39	17,92	17,53
Q5HHT0	0,91	0,80	0,57	0,46	2,96	5,23	14,79	15,02
ytxJ	1,87	2,18	0,00	0,31	2,49	2,49	4,98	4,67
glxK2	1,33	1,60	0,89	1,33	4,89	5,16	14,58	15,02
pepT	0,98	1,39	1,14	0,57	5,05	6,03	13,20	13,28
Q5HHS6	0,00	1,41	0,81	0,40	2,63	3,84	11,11	11,11
DUF1212_L2	0,13	0,92	0,39	0,79	3,15	4,07	11,68	11,68
gdpS	0,56	0,84	0,37	0,37	3,64	2,89	9,34	9,34
tarO	0,47	0,57	0,47	0,28	3,31	3,50	13,35	13,16
Q5HHS2	1,87	0,93	1,56	0,62	3,89	4,67	14,64	14,64
degV1	0,69	1,15	0,69	0,46	3,46	3,81	10,27	10,27
comFA	2,22	5,24	1,42	1,51	10,12	10,12	26,26	25,47
comFC	2,95	2,21	2,80	3,39	6,19	5,90	23,30	23,45
yfiA	0,17	0,17	0,17	0,17	2,09	1,75	7,85	7,50
secA1	0,55	0,91	0,59	1,82	4,74	4,50	9,83	10,23
prfB	0,99	1,17	0,90	0,72	3,42	5,67	12,15	11,97
lysM	0,48	2,50	0,48	0,48	6,67	7,26	17,14	17,02
yfbR	0,92	0,46	0,31	0,61	3,99	4,61	10,14	9,98

Gene	vs. zoo28	vs. EMCR19	vs. SS60	vs. SS90	vs. NCTC13712	vs. MSHR1132	vs MW2	vs. SA17_S6
DUF2198	0,42	1,27	0,42	1,27	5,49	5,91	16,03	16,03
uvrB	0,75	1,05	1,46	1,05	4,72	4,42	13,35	13,25
uvrA	1,40	100,00	1,33	1,37	4,25	4,21	14,01	14,47
hpr	0,75	1,18	0,96	0,64	3,32	3,54	12,97	13,18
lgt	1,31	2,50	1,07	1,31	4,05	4,29	13,93	13,33
yvoF	1,44	2,46	2,05	1,64	6,57	7,19	22,79	22,38
yvcD	0,90	3,46	1,24	0,90	100,00	4,91	13,90	14,18
trxB	1,60	2,46	1,60	1,60	3,53	3,53	9,51	9,62
yvcJ	1,32	0,99	0,44	0,77	4,50	5,70	10,86	10,86
mgfK-yvcK	7,33	6,22	5,62	5,02	100,00	7,83	5,82	5,72
whiA	0,32	0,63	0,32	0,11	4,23	5,19	12,28	12,49
clpP	0,51	1,02	1,02	1,19	2,38	2,38	4,93	5,10
yfcH	3,10	100,00	1,66	1,88	100,00	10,07	16,81	17,37
Q5HHP8	2,02	100,00	0,78	0,93	6,67	7,60	20,31	20,62
gapR	1,48	0,99	1,38	1,78	2,76	2,17	8,38	8,28
gapA	0,10	0,10	0,00	0,20	0,49	0,69	1,88	1,78
pgk	0,59	0,84	0,92	0,84	2,18	2,18	8,23	8,56
tpi	0,92	0,79	0,39	0,39	1,97	1,57	5,38	5,64
gpmI	0,33	0,46	0,59	0,72	2,24	4,15	7,25	7,44
eno	0,46	0,54	0,23	0,46	2,45	1,84	3,14	3,60
Q2YSE7	1,96	1,31	0,87	0,87	2,18	7,19	16,34	16,78
secG	0,43	0,00	0,00	0,00	0,00	0,00	0,00	0,00
est	1,08	0,81	0,94	0,67	4,32	3,78	2,43	8,37
rnr	2,91	3,41	2,99	2,95	7,75	5,90	6,70	8,55
ssrP	3,23	3,01	2,15	0,65	5,16	5,59	3,44	5,59
Q1YB79	0,00	100,00	100,00	100,00	100,00	100,00	100,00	100,00
Q5HHN0	0,00	0,00	0,00	0,00	0,00	0,00	100,00	100,00
Q1XZI1	100,00	29,50	100,00	29,68	100,00	100,00	100,00	100,00
clfA	11,72	24,65	100,00	100,00	19,91	100,00	25,89	100,00
vwb	23,77	100,00	23,25	22,80	23,90	29,53	28,43	18,07
emp	2,55	12,48	100,00	100,00	100,00	29,33	24,68	17,49
vwb2	26,02	3,74	25,49	100,00	100,00	27,45	100,00	100,00
nuc1	0,43	0,43	0,29	0,58	6,81	6,96	19,28	20,29
cspC-L1	1,00	0,00	0,50	0,50	1,99	1,00	1,49	1,49
Q1Y1Z8	0,00	0,00	1,83	100,00	7,31	10,96	100,00	100,00
Q2G007	4,21	6,67	4,21	4,21	6,32	6,67	22,81	23,16
Q1YB69	1,23	1,40	1,23	0,88	4,39	5,26	21,05	100,00
Q6GIJ6	0,00	0,53	0,00	0,00	2,65	3,70	16,40	16,40
Q6GIJ5	0,00	0,00	0,00	0,38	4,98	3,07	14,94	15,33
DUF1250	0,00	0,00	0,00	0,00	3,43	8,82	16,18	16,18
Q6GIJ3	0,00	0,00	0,00	0,00	1,27	1,27	3,80	4,64
gpmA1-cobC	1,54	1,37	1,54	1,03	100,00	4,62	15,21	15,56
yisU1	1,13	0,97	1,13	1,46	100,00	8,25	21,04	20,87
Q5HHL4	0,40	3,43	0,61	0,61	4,44	2,42	12,73	13,13
ohrB	0,71	0,95	0,95	0,95	6,15	5,20	16,78	16,55
aroD	2,50	2,78	2,50	1,67	8,75	10,14	28,19	27,36
ntrA	0,56	1,30	0,56	0,56	3,70	4,44	10,19	10,19
trxA2	0,93	1,25	0,62	0,62	5,30	4,05	15,89	14,64
yusI	0,56	1,12	0,84	1,12	100,00	1,96	11,48	12,04
gcvH2	0,52	0,52	0,52	0,79	1,57	1,57	3,67	3,94
ywqG	2,28	1,37	1,14	3,41	100,00	6,83	11,15	11,60
yusF	0,78	1,03	1,03	0,78	8,79	6,46	15,25	14,99
yusE	0,67	0,34	0,34	0,67	5,05	2,69	9,09	9,09
metN1	1,85	1,46	0,58	1,36	4,19	4,29	14,91	15,20
metP1	1,29	2,30	2,73	2,30	4,60	3,88	12,36	12,36
metQ1	2,19	3,28	1,09	0,85	3,89	4,62	10,10	11,31
csbD-L1	2,05	0,51	1,03	0,51	4,10	3,59	5,64	6,15
DUF368	2,46	1,41	0,70	1,29	6,22	7,04	13,62	13,38
sufC	1,18	0,79	1,05	1,05	3,54	1,44	3,81	4,20
sufD	1,99	1,61	1,38	1,99	4,59	4,05	8,41	8,03
sufS	1,60	0,88	0,64	0,48	6,79	4,16	11,19	11,43
sufN	0,65	0,00	0,00	0,00	4,95	3,66	4,95	4,95
sufB	1,36	1,93	0,72	1,65	4,51	2,50	6,87	7,15
Q2YWM5	4,02	100,00	4,64	1,86	17,65	17,34	17,34	100,00
corB	0,67	2,11	0,67	0,86	5,09	4,03	11,91	100,00
npd	1,50	2,15	1,59	1,78	100,00	6,84	15,07	15,17
yunF	2,71	2,00	1,06	0,24	4,12	4,48	9,78	10,25
yunE	2,54	2,17	1,33	0,72	6,88	8,45	13,04	13,77
yunD	0,76	3,03	1,06	0,83	5,38	8,03	15,98	16,36
lipA	0,11	0,65	0,22	0,22	1,53	1,96	5,23	5,34
DUF1027	1,02	0,25	1,27	1,02	8,65	2,29	9,92	9,92
DUF3055	0,75	1,87	0,75	1,12	4,12	7,12	17,60	17,98
yutE	2,07	1,61	1,15	1,84	8,05	4,60	12,87	12,87
yutF	1,03	1,15	0,90	0,90	3,97	5,00	13,21	14,36
gyaR	1,04	1,88	1,04	1,46	6,67	6,35	19,58	19,58
dltX	0,65	1,31	0,65	0,65	1,31	0,65	5,23	5,23
dltA	1,17	1,58	1,37	0,69	4,18	3,50	10,08	10,29
dltB	1,32	0,91	1,07	1,07	1,98	1,81	7,57	7,74
dltC	0,00	0,00	0,00	0,00	0,42	0,42	0,42	0,84
dltD	0,26	0,60	0,17	0,68	1,53	2,64	7,14	7,14
nfuA	0,82	0,82	1,65	1,65	9,88	1,65	4,94	5,35
yuzD	1,85	0,62	0,93	0,62	4,01	5,25	17,90	17,90
yutJ-ndhF	0,94	1,22	1,31	1,13	2,63	3,94	12,49	12,02
yuzB	0,42	0,00	1,27	0,00	1,27	1,27	8,02	8,02
sufA	1,11	1,39	1,39	0,28	4,44	4,17	13,33	14,44
ndhC-yumB	0,17	0,66	0,33	0,66	3,56	1,90	5,87	5,96
pepZ	1,48	2,50	0,74	1,35	8,84	8,43	18,42	18,22
nhaC3	1,29	2,05	1,21	0,99	3,80	4,56	12,68	13,06
yuxO	0,27	0,53	0,53	0,27	2,13	6,93	12,80	12,00
Q5HHE0	1,30	2,25	1,39	2,08	14,11	5,37	17,40	16,80
mnhG	0,56	1,40	0,56	1,40	10,08	4,76	10,08	11,20

Gene	vs. zoo28	vs. EMCR19	vs. SS60	vs. SS90	vs. NCTC13712	vs. MSHR1132	vs MW2	vs. SA17_S6
mnhF	1,02	1,36	1,02	1,02	3,74	3,74	11,90	11,56
mnhE	1,67	0,83	1,67	1,67	3,54	3,33	9,38	8,96
mnhD	0,40	0,80	0,40	0,33	5,08	4,28	13,29	13,16
mnhC	0,58	0,88	0,58	0,29	0,29	2,34	6,14	5,85
mnhB	0,47	0,93	0,47	0,23	1,40	2,56	7,93	8,16
mnhA	0,58	1,04	0,58	0,62	2,91	4,57	9,85	9,85
kapB	1,30	2,86	1,56	0,52	3,91	6,51	11,72	11,72
prsA1	0,67	0,51	1,68	0,00	3,20	4,71	8,92	8,92
yugI	0,26	0,26	0,00	0,00	0,26	0,26	1,85	1,59
namA	0,57	1,05	0,65	0,57	100,00	7,20	12,62	12,46
rocD2	1,09	0,84	1,26	0,08	3,36	5,46	7,39	7,64
gluD	0,80	1,04	0,64	0,96	5,22	4,74	7,31	6,99
glpQ2	0,97	1,61	1,08	1,40	4,41	4,84	12,15	12,37
argH	2,10	3,91	2,46	1,96	6,30	7,61	10,14	10,22
argG	1,00	1,33	1,16	0,50	3,40	6,97	7,79	7,55
pgi	0,53	0,83	0,38	0,45	2,70	5,11	5,48	5,48
yhjE	0,69	0,87	0,69	0,87	3,99	6,60	12,15	12,50
spsA	0,95	0,76	1,33	1,14	100,00	4,38	8,57	8,76
spsB	1,74	4,86	2,08	1,91	5,03	5,56	9,55	9,90
rexB	1,38	1,73	1,15	1,32	2,16	7,19	12,94	13,34
rexA	2,38	2,24	1,94	1,94	2,87	6,95	15,02	15,30
Q5HHB6	1,66	0,44	0,33	1,11	3,21	3,88	7,97	7,86
UPF0344	2,56	3,08	2,56	2,56	100,00	9,49	11,03	13,59
cdr	0,91	1,59	0,68	0,84	11,92	7,44	13,44	13,44
yitU-yidA	1,58	1,21	0,48	0,73	3,15	4,73	9,70	9,33
sufT-paaD	0,32	0,32	0,32	0,00	1,94	5,83	9,06	8,74
Q5HHB1	2,61	2,12	2,50	2,34	7,88	12,17	21,52	21,63
clpB	2,15	1,07	1,49	1,46	11,00	8,35	10,80	10,92
Q5HHA9	1,37	1,37	1,37	0,80	20,39	15,46	20,27	20,62
Q5HHA5	100,00	0,91	0,91	0,91	100,00	100,00	100,00	100,00
eapH-2	0,00	0,00	0,00	0,00	100,00	100,00	100,00	100,00
A6U088	0,54	0,00	0,00	0,54	10,22	2,69	10,75	10,75
fabH	0,64	0,32	0,85	1,27	7,54	3,40	8,60	8,39
fabF	1,29	100,00	1,69	1,12	10,20	7,71	10,68	10,76
Q2YWV0	0,80	100,00	100,00	100,00	18,18	10,70	18,18	18,45
oppB	2,78	2,67	2,56	2,46	8,87	4,06	9,40	9,62
oppC	0,75	1,40	1,31	1,31	11,86	5,42	11,67	11,48
oppD	0,46	0,55	1,11	0,92	8,31	2,12	8,68	100,00
oppF	0,21	100,00	100,00	0,74	11,24	3,50	8,27	11,56
oppA	0,18	100,00	0,91	3,50	3,32	6,28	10,57	11,11
appA	0,29	4,60	0,52	0,64	100,00	14,33	4,89	5,82
appD	1,11	1,11	2,13	1,52	100,00	19,74	4,25	4,86
appF	1,02	1,32	1,42	100,00	100,00	15,77	5,39	7,73
appB	29,86	100,00	100,00	100,00	100,00	100,00	100,00	100,00
appC	24,04	24,49	23,71	24,04	100,00	100,00	27,19	26,85
trpS	1,11	1,41	1,01	1,11	8,89	5,56	10,20	9,29
spxA	0,00	0,00	0,00	0,00	0,00	0,00	0,25	0,25
trfA	0,83	1,25	1,11	1,39	9,03	7,08	8,89	9,31
trfB	1,92	2,13	3,24	2,43	28,64	16,70	28,54	28,04
pepF1	1,44	1,66	1,11	1,49	10,61	6,69	10,67	10,50
yjbH	0,87	1,61	0,87	1,49	3,10	5,82	9,05	9,29
yjbI	0,55	0,27	0,55	1,09	3,28	4,64	10,93	12,02
yjbK	1,01	0,84	0,67	0,51	6,40	11,45	17,85	18,18
yjbL	0,57	0,29	0,00	0,00	1,15	1,44	11,21	11,21
relQ	0,94	0,63	1,10	0,79	5,03	6,13	11,95	12,26
ppnK	1,48	1,48	1,36	1,36	3,09	3,21	12,84	12,96
rluE	1,17	2,11	2,46	100,00	7,02	8,42	19,53	20,35
mgtE	1,52	2,09	1,88	1,66	6,28	6,78	12,41	13,06
cpaA	1,14	2,55	2,01	1,95	5,37	5,58	13,66	13,60
fabI	1,95	2,08	3,11	1,43	6,74	6,36	6,10	6,61
UPF0118	1,01	5,06	1,29	0,55	7,37	6,45	5,99	6,17
yrbD	16,51	2,36	0,57	0,89	8,03	6,37	3,57	3,70
yjcH	100,00	100,00	3,02	100,00	9,99	10,91	7,62	8,41
UPF0477	0,78	0,78	0,59	0,39	1,18	0,78	1,37	1,57
ltaA	2,77	2,10	1,51	2,60	6,21	8,82	9,74	9,82
ugtP	5,70	1,36	0,77	0,26	5,70	7,40	8,33	8,67
murE	1,62	100,00	2,42	2,22	10,17	8,96	10,44	10,37
yueH	2,67	3,05	3,05	3,82	100,00	10,69	21,37	21,37
prfC	1,34	100,00	2,24	1,22	8,13	7,42	9,60	9,66
terC	0,62	1,62	1,12	0,37	9,33	8,46	8,71	9,20
htrA	2,24	2,87	6,12	1,77	10,34	12,28	100,00	100,00
ktrD	1,10	0,88	1,03	0,81	4,12	4,71	11,70	11,92
Q1XY52-yfkN3	1,85	1,32	1,25	1,45	6,27	5,61	16,04	100,00
comK	0,35	0,35	1,05	0,88	4,04	3,86	6,84	6,67
Q6GI58	1,32	0,88	1,32	1,32	2,63	2,19	2,63	2,63
lplA2	0,61	1,11	5,07	2,23	5,27	9,02	4,36	3,85
Q6GI56	0,00	0,00	0,00	0,00	0,00	2,26	0,00	0,00
Q5HH56	0,66	0,83	0,83	0,66	5,97	5,80	2,49	2,16
txpA-var1	0,49	0,49	0,49	0,49	100,00	1,46	2,44	1,46
Q7A194	10,30	2,66	10,96	11,63	1,99	5,32	7,97	3,32
yujE	0,76	2,34	1,02	1,27	1,98	6,82	3,77	3,56
yxeA	0,62	0,62	0,62	0,31	0,93	7,48	2,49	2,49
yujD	1,40	2,65	1,56	1,71	2,34	10,59	3,74	3,89
Q6GI49	1,37	1,37	1,37	1,03	8,25	7,90	7,90	100,00
Q6GI46	0,28	0,00	0,00	0,00	0,00	1,40	4,76	4,48
Q5HH47	10,74	4,29	7,06	10,53	9,41	12,27	14,42	100,00
shpA	0,85	2,56	0,85	2,56	8,55	3,42	17,95	18,80
Q7A191	4,63	4,63	4,17	5,09	100,00	8,80	19,44	19,91
Q6GAH2	0,89	1,43	1,07	0,72	9,66	9,84	19,68	19,68
menA	0,85	0,96	0,43	0,85	3,51	3,94	12,03	11,93
menF	0,29	0,81	0,44	0,37	3,82	3,38	13,88	100,00

Gene	vs. zoo28	vs. EMCR19	vs. SS60	vs. SS90	vs. NCTC13712	vs. MSHR1132	vs MW2	vs. SA17_S6
menD	1,43	2,15	0,78	0,48	4,48	5,97	16,55	16,73
menH	0,75	1,12	0,87	1,37	6,22	4,98	22,14	22,64
menB	1,22	1,09	1,46	0,12	2,07	2,80	8,76	8,03
sspC	0,30	1,49	0,30	2,38	5,06	5,36	17,56	18,15
sspB	0,84	1,51	1,09	1,00	4,94	8,70	13,81	13,89
sspA	5,82	7,88	6,76	4,02	18,92	15,58	23,72	24,14
aspC	0,61	1,90	0,43	0,43	3,29	6,75	17,14	100,00
ykrP	2,77	0,99	1,38	1,09	4,35	5,14	14,64	100,00
Q5HH32	0,48	1,19	1,19	0,48	2,38	3,33	13,57	13,57
atl	1,02	2,35	1,15	0,89	3,63	6,42	12,82	13,19
UPF0039	1,14	100,00	0,68	0,68	100,00	7,74	22,10	100,00
Q5HH29	0,42	0,64	0,42	0,42	100,00	3,18	10,17	100,00
tagV-lcpB	1,77	0,96	0,88	0,88	5,46	6,59	11,57	12,13
fmtA	3,18	3,27	1,17	0,92	5,61	5,44	14,57	100,00
qoxD	0,69	0,69	0,69	0,69	5,50	1,03	4,47	4,12
qoxC	0,17	0,50	0,17	0,17	1,32	1,16	3,96	4,13
qoxB	0,45	0,35	0,40	0,45	1,26	1,16	3,02	3,22
qoxA	0,27	0,36	0,45	0,45	1,36	1,27	3,27	3,27
iraE	1,89	1,89	1,26	1,89	1,57	0,31	9,43	9,75
folD	1,51	2,44	1,62	1,16	5,10	4,76	12,41	12,41
purE	1,45	2,28	1,04	0,83	6,21	5,59	14,49	14,29
purK	1,51	1,87	1,16	1,51	4,80	4,62	17,07	17,78
purC	0,99	1,99	1,13	0,99	5,11	6,67	17,16	18,16
purS	0,00	1,89	0,38	1,89	4,17	3,79	11,36	11,74
purQ	0,89	2,23	0,89	1,34	4,02	3,72	12,20	12,50
purL	2,28	2,83	2,15	2,56	3,20	4,84	13,29	13,52
purF	1,95	2,76	1,75	1,41	5,79	4,85	12,93	13,00
purM	1,75	1,75	1,07	2,24	4,96	100,00	15,94	15,74
purN	1,06	0,88	1,41	1,23	3,70	4,23	14,29	13,76
purH	1,08	1,28	1,15	1,15	4,87	4,87	12,71	12,78
purD	1,44	1,44	2,08	100,00	3,04	6,32	16,64	100,00
ykoC	1,36	2,23	1,36	0,99	3,97	2,97	13,63	13,26
ykoD	1,64	2,78	1,71	1,57	5,07	4,28	17,99	17,84
ykoE	0,35	1,39	1,04	0,52	3,99	3,30	9,38	9,03
graF	0,00	0,74	0,00	2,96	100,00	5,19	7,41	7,41
Q5HH05	0,77	0,54	0,46	0,69	2,24	4,40	7,72	7,95
ywbD	1,62	2,56	2,47	2,13	6,99	5,37	14,32	14,41
Q5HH03	0,92	0,74	0,74	0,92	3,87	3,68	11,79	10,50
ptsH	0,00	0,00	0,00	0,75	1,87	1,50	1,50	1,12
ptsI	0,64	0,87	0,99	0,58	2,62	2,56	6,69	6,86
nrdH	0,43	0,85	0,43	0,85	100,00	100,00	21,70	21,28
cydA	0,95	1,40	1,54	1,17	7,93	4,04	11,53	11,60
cydB	1,08	0,98	0,69	0,69	6,86	3,14	12,35	12,94
ktrA	1,06	0,90	1,36	0,45	3,17	3,92	14,03	13,88
rnjA	0,47	0,47	0,06	0,06	3,30	2,83	10,66	10,66
rnpZA	0,46	0,46	0,46	0,46	1,37	1,37	4,11	4,11
defB-def1	0,00	0,72	0,00	0,54	5,25	13,41	15,22	15,40
ykyA	0,79	4,58	0,63	0,16	100,00	10,27	17,69	16,75
pdhA	0,99	0,09	0,90	0,45	0,27	2,79	3,32	3,86
pdhB	0,10	0,20	0,00	0,10	0,72	2,35	6,24	5,93
pdhC	2,69	2,00	1,92	1,46	2,00	3,38	5,92	7,38
pdhD	0,43	0,50	0,43	0,43	0,71	2,27	3,84	3,84
UPF0223	0,36	1,81	0,00	0,72	5,80	6,16	23,19	22,83
puuR	0,19	0,19	0,19	0,37	1,48	2,96	10,19	10,56
potA	0,64	0,82	100,00	0,27	4,84	4,66	10,50	10,96
potB	0,63	0,63	0,00	0,25	5,01	5,64	10,53	10,28
potC	0,62	1,23	0,62	2,72	4,44	100,00	14,57	14,94
potD	1,02	0,93	0,65	0,74	4,10	5,12	12,85	12,85
Q5HGY1	0,69	0,59	1,08	0,78	2,65	2,94	13,14	13,14
mntH	0,74	1,18	0,44	0,59	4,51	9,53	11,83	12,12
yktB	0,49	1,63	0,65	0,65	5,85	7,97	13,50	13,50
suhB1	1,09	1,33	0,24	100,00	4,71	4,95	17,75	16,91
ylaF	0,52	0,52	0,52	0,52	5,73	4,69	6,77	5,73
typA	1,03	1,57	1,84	1,30	3,41	3,25	9,79	9,36
ylaL	0,21	0,62	1,24	1,04	3,73	2,69	11,18	11,18
ylaN	0,00	0,00	0,00	1,81	3,62	1,81	5,80	5,80
ftsW2	0,81	2,53	0,65	0,90	4,48	3,75	13,61	13,61
pycA	1,07	1,65	1,07	0,61	100,00	4,11	11,82	11,87
ctaA	0,63	100,00	0,74	100,00	7,50	3,17	13,09	13,41
ctaB	0,44	0,22	0,33	0,33	2,63	1,43	5,59	5,81
ctaM-yozB	0,43	0,22	1,30	1,08	8,66	2,16	9,09	8,87
ylbC2	0,33	0,67	0,56	0,33	100,00	6,33	13,89	13,56
ylbF	0,46	100,00	0,23	0,46	14,94	5,52	15,63	14,94
yhdW	2,00	1,58	2,00	1,69	5,27	6,12	20,68	22,89
ylbG	0,78	1,18	0,39	1,18	7,84	2,75	8,63	8,24
Q5HGW1	0,26	3,07	0,77	100,00	100,00	3,84	14,83	14,83
rsmD	0,55	0,55	0,37	0,55	4,79	2,58	10,68	10,68
coaD	0,62	0,41	0,00	100,00	5,80	4,35	7,66	7,66
ylbM	0,79	1,05	0,70	0,53	9,74	6,05	20,61	21,05
ylbN-Q4L5E0	0,36	0,72	0,54	0,54	1,61	3,41	5,73	5,73
rpmF	0,00	0,00	0,00	0,00	1,72	1,15	1,15	1,15
isdB	1,35	2,51	8,82	1,25	18,19	19,59	20,09	20,79
isdA	0,84	1,78	0,84	0,84	13,38	13,28	20,49	19,74
isdC	0,15	0,73	0,15	3,51	8,63	11,11	16,08	16,52
isdD	1,20	1,85	0,83	0,92	12,00	9,60	19,67	20,41
isdE	0,46	0,57	0,68	0,57	0,68	6,26	13,20	13,54
isdF	0,31	0,52	0,31	0,41	0,83	5,28	20,50	20,39
srtB	0,68	0,68	0,68	0,82	100,00	5,58	15,24	16,05
isdG	0,62	0,62	0,62	0,62	2,16	3,09	9,88	9,57
spoU	0,81	1,62	1,62	0,81	1,48	7,96	14,17	13,63
pheS	1,89	2,46	1,70	1,51	2,64	4,06	8,59	8,78

Gene	vs. zoo28	vs. EMCR19	vs. SS60	vs. SS90	vs. NCTC13712	vs. MSHR1132	vs MW2	vs. SA17_S6
pheT	1,58	2,04	1,58	1,66	2,00	5,53	14,36	14,40
rnhC	0,96	1,17	0,53	2,77	1,49	4,79	18,64	18,53
zapA	0,75	0,75	0,37	0,75	0,75	1,50	10,86	11,24
yshB	2,30	0,96	0,96	0,57	0,96	5,94	16,48	100,00
polX	1,46	1,34	1,23	0,64	1,87	6,01	14,54	14,24
mutS2	1,28	1,32	1,23	2,13	3,45	5,36	10,56	10,60
trxA1	1,27	1,59	1,27	1,27	1,27	1,27	3,81	3,49
uvrC	0,79	1,63	1,01	0,79	5,05	5,84	13,24	100,00
sdhC	0,00	1,14	0,98	0,00	2,11	3,74	7,64	7,64
sdhA	0,96	1,30	1,19	1,53	4,19	5,15	7,81	7,75
sdhB	0,61	0,00	0,86	0,86	5,02	4,17	9,44	9,19
murI	0,75	0,25	1,25	1,12	11,49	5,49	12,98	12,98
ntpA	0,85	0,34	1,53	3,57	100,00	13,58	15,28	100,00
ysnB	2,98	4,17	2,18	3,17	16,87	11,11	14,29	14,29
ecb	7,58	3,94	4,24	7,58	10,91	12,42	9,70	12,42
flr	24,14	100,00	14,04	1,23	100,00	26,35	23,65	100,00
Q2YXB9	0,39	0,59	0,20	0,39	100,00	11,39	11,00	100,00
efb	1,81	1,61	0,60	1,20	10,04	15,46	7,63	100,00
scc	1,14	2,84	1,14	1,14	100,00	23,58	18,18	100,00
A5IS45	0,54	1,08	1,08	0,00	8,60	16,67	11,83	13,98
Q6GHS5	0,00	0,00	0,00	0,00	0,00	100,00	100,00	0,00
A5IS47	1,71	100,00	1,71	1,28	19,23	14,10	19,66	20,94
hla	0,42	1,04	100,00	1,04	10,21	14,48	10,00	10,10
A6U0Y3	0,00	0,00	0,00	0,00	0,00	100,00	100,00	0,00
Q2FZB4	3,45	2,46	2,46	3,45	100,00	19,70	16,26	100,00
setB3	1,94	1,66	0,55	1,80	100,00	20,78	20,36	100,00
setB2	0,00	0,00	0,00	0,00	0,00	100,00	100,00	0,00
setB1	1,51	5,08	100,00	100,00	100,00	15,91	15,91	100,00
arcB-L1	1,60	1,30	1,00	0,90	7,88	12,38	7,68	7,78
arcC-L1	0,53	0,64	0,96	0,75	5,24	7,26	12,50	13,03
arcD-L1	1,67	1,93	2,31	2,44	7,51	9,57	12,20	12,14
A5IS57	0,43	100,00	100,00	2,17	10,87	13,48	14,78	100,00
per	100,00	100,00	1,16	100,00	17,05	7,26	17,47	100,00
psmB1	0,00	1,48	1,48	0,00	4,44	3,70	4,44	5,19
Q1Y2B3	0,99	0,56	0,99	0,99	100,00	9,04	20,76	100,00
Q931T2	2,27	0,91	1,36	2,49	4,08	2,95	9,30	9,52
bshC	1,98	0,99	1,12	1,24	21,19	7,00	21,38	21,75
mraZ	0,46	0,93	0,00	0,00	1,85	1,39	3,24	3,24
mraW-rsmH	3,21	4,38	2,78	3,21	8,65	3,42	9,19	9,19
ftsL	0,25	0,25	0,25	0,25	1,24	1,24	1,24	1,49
pbpA	0,36	1,34	0,45	0,45	4,30	3,94	11,28	11,28
mraY	1,04	1,55	0,83	0,72	4,76	5,69	13,66	13,77
murD	0,52	0,96	0,52	0,52	4,44	4,59	14,67	14,52
ftsQ	0,85	100,00	0,92	0,64	7,71	8,56	21,44	21,44
ftsA	0,14	0,57	0,07	0,21	1,49	1,34	4,53	3,82
ftsZ	0,17	1,11	0,09	0,09	4,01	2,64	7,93	7,93
ylmD	0,75	3,51	0,75	1,13	18,92	8,52	18,92	19,17
ylmE	0,30	6,81	100,00	0,30	8,15	2,52	8,30	8,44
sepF	1,06	5,50	0,53	0,53	5,85	2,13	6,21	6,03
ylmG	0,00	4,12	0,00	0,00	6,87	3,44	5,84	5,50
ylmH	0,25	0,50	0,37	0,62	2,85	4,34	13,63	10,53
divIVA	0,79	3,17	0,79	1,27	7,94	6,35	12,86	12,70
ileS	1,02	1,34	0,73	0,44	4,68	4,97	9,80	9,66
PF00903-catE	2,63	100,00	3,00	1,63	100,00	7,26	17,52	17,27
lspA	0,81	0,20	0,81	1,22	3,25	3,05	13,41	13,21
ylyB	2,83	5,34	1,96	3,16	6,21	5,12	13,51	13,62
pyrR	0,76	2,46	0,00	1,33	4,17	5,49	10,98	11,17
pyrP	0,61	2,68	1,07	0,69	3,82	3,82	12,31	12,54
pyrB	1,69	1,13	1,02	1,02	4,97	5,31	12,54	12,54
pyrC	1,41	2,97	1,96	1,41	6,10	5,48	11,27	11,42
pyrAA	1,36	3,36	1,63	2,18	5,36	5,09	10,90	11,17
pyrAB	1,64	1,67	2,30	1,67	5,55	4,91	12,10	12,10
pyrF	1,15	4,76	0,72	2,74	5,63	4,62	14,57	14,57
pyrE	1,63	2,78	1,80	2,94	5,72	4,41	12,91	12,25
Q5HGM6	0,00	0,47	0,00	0,94	2,82	3,29	22,07	22,07
Q5HGM5	1,24	0,25	0,75	0,75	4,73	3,73	14,18	15,17
fbpA	1,47	1,53	1,59	1,41	5,65	5,65	15,25	15,19
gmk	0,64	1,12	0,32	0,16	1,44	1,28	5,45	5,45
rpoZ	0,00	0,91	0,00	0,00	1,37	0,91	8,68	8,22
coaBC	0,50	2,58	1,00	2,00	4,92	6,00	15,33	14,83
priA	1,54	1,78	1,45	1,33	8,05	6,27	15,15	15,32
Q5HGL9	100,00	0,82	100,00	100,00	100,00	100,00	100,00	100,00
Q5HGL8	0,72	0,72	1,43	0,36	4,30	100,00	100,00	100,00
defA-def2	0,41	1,02	100,00	2,04	15,95	10,84	16,16	16,56
fmt	1,50	2,56	2,46	2,14	10,47	7,37	13,46	13,46
rsmB-sun	1,15	1,76	0,38	0,92	3,59	3,67	15,52	15,98
rlmN	0,73	0,73	0,27	0,27	7,58	2,83	9,95	9,68
prpC-stp1	1,08	1,48	1,34	1,08	2,96	1,61	8,74	8,87
prkC-pknB	0,95	1,30	1,10	1,20	13,73	4,71	15,04	14,74
cpgA-engC	0,57	1,83	0,80	0,80	4,34	4,34	16,21	15,98
rpe	1,55	1,40	0,78	0,78	4,96	5,58	16,28	16,28
thiN	1,71	2,64	1,71	1,55	6,67	4,50	19,22	19,84
rpmB	0,00	0,00	0,00	0,00	0,00	0,00	0,53	0,53
yloU	1,60	1,33	1,87	2,13	2,40	2,93	4,80	5,60
yloV	0,79	1,03	1,09	1,03	4,31	3,58	11,05	11,35
recG	1,75	2,13	1,21	1,21	5,63	6,26	16,25	16,30
fapR	3,66	0,87	3,32	3,32	4,71	1,75	8,38	7,68
plsX	0,41	1,62	1,22	0,71	9,22	4,26	10,84	11,25
fabD	1,19	1,29	0,76	0,76	5,72	3,34	11,43	11,54
fabG1	1,36	0,68	1,50	0,82	3,95	3,40	10,07	9,93
acpP	0,00	0,00	0,00	0,00	0,00	0,00	0,00	0,00

Gene	vs. zoo28	vs. EMCR19	vs. SS60	vs. SS90	vs. NCTC13712	vs. MSHR1132	vs MW2	vs. SA17_S6
rnc	0,27	0,68	0,27	0,41	1,91	2,19	8,88	8,74
smc	1,29	1,85	1,26	1,15	5,46	4,82	15,15	15,13
ftsY	0,72	2,32	2,24	2,00	5,92	4,32	13,35	13,51
ylxM	0,00	0,60	0,60	0,30	1,80	2,40	9,01	9,31
ffh	1,39	0,73	1,46	2,56	3,87	4,31	9,94	9,65
rpsP	1,09	0,36	1,09	0,36	1,81	1,81	2,54	2,90
rimM	0,79	0,99	0,60	0,60	5,75	5,16	13,89	13,89
trmD	1,90	1,49	2,44	2,17	8,27	8,13	12,60	12,33
rplS	0,57	0,28	0,57	0,57	1,14	0,57	0,85	1,14
yfhO	3,76	100,00	3,80	100,00	12,08	12,38	14,07	14,00
rbgA	1,13	1,47	0,90	1,02	9,15	6,78	8,25	9,27
rnhB	1,30	3,26	1,43	2,60	9,77	12,50	13,54	13,28
sucC	1,46	1,71	1,46	1,37	4,28	4,03	4,37	4,03
sucD	0,77	4,73	0,99	1,21	5,61	3,74	5,06	5,06
dprA	25,11	1,36	1,14	1,36	28,07	27,95	27,73	3,41
topA	0,67	0,82	0,63	0,82	7,13	7,66	8,00	8,04
trmFO-gid	1,30	1,45	100,00	100,00	100,00	4,50	12,44	12,90
xerC	0,45	1,23	0,33	0,45	3,01	3,46	12,49	12,37
hslV	0,73	1,10	0,73	1,10	4,76	6,04	11,36	11,36
hslU	0,36	0,71	0,36	0,85	4,13	4,91	11,11	11,04
codY	0,52	0,65	0,78	0,26	4,01	5,17	8,27	8,79
rpsB	0,52	0,39	0,39	0,39	1,43	1,17	2,34	2,21
tsf	0,79	1,02	0,91	0,91	1,13	1,59	5,22	5,67
pyrH	0,14	0,69	0,00	0,00	1,38	2,77	7,19	7,33
frr	0,54	0,36	1,08	1,08	2,88	3,42	10,99	10,99
uppS	0,00	1,17	0,00	0,13	2,85	2,72	10,12	9,47
cdsA	1,53	1,15	1,28	1,28	3,32	5,24	10,86	11,11
rseP	1,79	1,79	1,71	1,71	10,02	6,68	11,11	11,27
proS	1,23	2,11	0,76	1,17	9,10	4,99	10,97	11,33
polC	0,79	1,69	0,76	0,93	5,86	4,45	7,62	7,92
rimP	0,85	0,85	1,07	0,64	2,78	4,06	5,34	5,98
nusA	1,02	2,64	1,96	2,47	2,98	7,06	12,16	12,16
ylxR	1,05	1,75	1,75	1,75	1,40	4,21	7,72	8,42
ylxQ	1,25	0,00	1,25	0,31	100,00	1,25	7,84	100,00
infB	0,99	2,21	1,60	1,22	4,42	5,17	9,12	9,07
rbfA	0,00	0,57	0,28	0,00	1,42	2,56	7,12	7,12
truB	1,63	2,61	2,29	2,18	4,14	3,92	14,60	14,05
ribF	1,03	1,23	1,44	0,93	4,42	4,53	12,24	12,04
rpsO	0,37	0,74	0,37	0,37	1,48	1,48	2,22	2,22
pnpA	0,48	1,67	0,86	1,05	4,29	5,29	9,92	10,01
rnjB	1,14	1,37	1,08	1,25	3,46	3,41	10,04	10,04
ftsK	2,23	2,23	2,19	2,15	5,23	5,27	19,72	14,75
ymfC	1,26	1,82	1,12	1,12	3,36	3,78	12,89	12,61
ymfF	1,58	1,11	1,90	0,71	100,00	4,90	15,01	15,09
ymfH	1,55	1,40	1,40	2,72	4,12	5,52	14,53	14,84
fabG2	2,98	1,99	2,27	1,99	5,25	5,39	18,72	19,15
ymfK	0,97	1,09	1,09	0,60	1,21	3,14	11,84	11,84
ymfM	1,78	1,27	0,00	0,00	1,27	3,31	11,20	11,45
pgsA	0,17	0,52	0,86	1,04	2,25	3,97	12,61	12,61
cinA	2,08	2,26	2,08	2,34	5,03	5,47	21,53	21,53
recA	0,96	1,25	1,44	0,57	3,07	4,21	10,92	10,73
rny	0,90	0,90	0,58	0,71	1,73	1,60	3,65	3,78
Q5HGE4	1,39	2,31	1,39	1,39	4,17	2,31	11,57	11,57
ymdB	1,88	1,75	1,50	1,75	1,75	3,51	14,66	14,79
porA	0,97	1,31	0,80	0,85	100,00	4,54	14,42	14,48
porB	0,58	1,27	1,27	1,15	4,27	3,23	12,34	12,34
DUF77	0,34	1,02	100,00	1,02	4,07	6,44	13,56	12,88
miaB	1,68	1,23	1,36	1,36	3,56	4,21	12,10	12,49
ymcA	3,01	100,00	3,01	3,01	4,10	4,10	15,30	15,57
thiW	2,24	2,85	2,24	2,44	5,28	3,46	12,80	12,60
mutS	1,34	2,63	1,57	0,88	4,51	5,12	14,89	15,08
mutL	1,44	0,95	1,54	1,59	5,72	5,02	15,17	15,42
glpP	0,92	1,66	0,18	1,10	3,87	6,45	15,84	15,84
glpF	0,98	1,47	0,98	0,73	5,01	6,96	10,50	10,74
glpK	1,20	1,87	1,54	1,80	4,81	4,81	11,36	11,09
glpD	3,43	2,61	0,75	0,75	8,25	6,62	11,67	12,72
pldB2	0,87	0,66	0,66	1,64	6,12	8,31	16,39	17,16
miaA	1,28	0,75	1,07	1,28	2,35	3,42	12,29	12,50
hfq	0,43	0,43	0,43	0,43	2,99	1,28	2,56	2,99
gpxA-L1	1,26	100,00	1,26	0,84	3,14	5,45	13,00	12,16
ynbA	1,69	0,89	0,97	0,97	5,88	5,88	12,16	12,24
ynbB	0,97	2,10	100,00	1,13	7,26	7,02	11,22	11,78
glnR	0,00	0,27	0,81	0,27	0,27	1,08	1,08	1,08
glnA	0,67	0,37	0,45	0,75	2,61	2,61	2,83	5,15
A6U1C8	0,51	1,52	100,00	0,51	2,02	2,53	2,53	2,02
Q2FYY4	1,78	2,22	4,44	2,67	3,11	4,44	4,00	3,56
A5ISJ3	1,90	100,00	100,00	100,00	10,95	10,00	1,90	8,57
Q2YXQ4	2,15	1,08	100,00	3,23	8,60	8,06	8,06	9,68
A6QGL8	1,46	2,63	2,63	2,34	4,19	9,06	9,65	10,23
A5ISJ9	3,03	2,53	3,03	2,53	10,61	7,07	2,53	12,12
cls1	1,89	2,50	1,35	1,21	7,29	8,64	6,55	11,94
Q99UF3	0,00	9,52	0,53	1,06	8,99	8,99	11,64	11,64
nuc2	0,19	0,19	0,56	0,00	3,18	7,49	9,74	9,36
Q5HG96	0,70	1,29	0,94	0,82	1,40	8,07	10,06	9,71
Q5HG95	0,17	0,85	5,41	0,34	1,35	4,57	10,15	9,31
thrA	0,29	1,08	0,72	0,80	2,31	5,13	12,44	12,22
hom	0,62	0,86	0,62	1,17	1,41	3,67	10,77	11,09
thrC	1,51	3,48	1,60	1,69	2,64	7,16	13,37	13,18
thrB	1,75	13,55	1,09	0,98	4,15	5,46	13,01	14,10
yxeH	1,37	100,00	1,24	1,49	1,87	4,35	12,94	11,94
Q5HG88	0,63	2,86	0,63	0,32	3,49	3,17	9,52	9,21

Gene	vs. zoo28	vs. EMCR19	vs. SS60	vs. SS90	vs. NCTC13712	vs. MSHR1132	vs MW2	vs. SA17_S6
lysP2-Q5HG87	0,55	0,89	0,55	0,55	5,22	3,51	17,25	17,46
katA	1,25	1,91	1,05	1,05	4,35	4,87	11,13	11,40
rpmG2	0,67	1,33	0,67	0,67	0,67	0,67	2,00	2,00
rpsN	0,00	0,74	0,00	0,00	2,59	2,22	7,41	7,78
guaC	0,72	1,74	100,00	100,00	5,82	3,17	11,13	11,75
ylbC1	15,91	1,96	0,39	0,69	1,28	9,82	13,56	13,36
lexA	0,32	1,44	1,12	0,32	4,33	4,81	13,30	13,14
Q5HG79	0,85	5,98	0,43	0,85	5,56	3,42	16,67	17,95
DUF896	3,75	100,00	3,75	2,92	5,00	3,75	11,25	11,25
tkt	1,11	1,46	1,01	0,96	3,67	3,22	10,26	10,61
UPF0154	1,23	100,00	0,82	0,82	2,88	2,06	5,35	5,76
ccdC	1,28	0,64	1,28	0,64	3,85	2,78	12,61	13,25
sbcD	1,43	1,78	1,60	1,52	6,68	4,90	20,86	20,94
sbcC	1,48	2,14	1,91	1,91	7,75	7,59	25,49	25,89
mscL	0,55	0,55	0,55	0,55	1,93	2,48	10,47	11,02
opuD1	0,49	0,85	0,49	0,61	3,70	2,61	11,60	12,02
acnA-citB	1,92	1,74	0,85	0,74	3,03	4,07	11,57	11,71
yneP	0,21	0,43	0,85	0,85	0,85	5,56	16,03	100,00
yneR	1,01	1,68	1,01	1,01	2,36	4,04	8,08	7,74
plsY	0,99	0,99	0,66	0,82	3,12	3,12	10,02	10,34
grlB	0,15	1,10	1,00	0,70	4,52	4,02	10,99	11,40
grlA-parC	1,08	1,25	1,17	100,00	4,62	4,99	13,44	13,28
alsT	0,34	0,75	0,27	0,07	5,00	3,08	11,77	11,77
glcT	0,70	0,70	0,59	0,23	2,58	4,58	13,85	14,44
yubA	0,25	0,66	0,17	0,17	3,64	3,06	12,82	12,66
mprF	1,66	0,95	0,79	1,39	4,40	3,61	12,72	12,37
msrA1	0,39	0,39	1,76	0,59	4,71	3,53	13,53	13,73
lcpA-tagT	0,20	0,41	0,20	0,20	5,59	5,69	9,45	9,65
dmpI	0,54	100,00	0,00	0,54	1,08	2,69	10,75	10,75
uvrX	0,95	0,71	0,55	0,40	8,47	6,25	11,32	11,56
tyrA	1,83	1,65	1,47	1,37	17,22	10,62	17,40	17,58
yhfE	1,45	2,33	1,16	1,36	15,60	8,14	15,60	15,60
trpE	1,28	1,63	1,85	1,00	16,13	6,82	16,77	16,92
trpG	4,20	100,00	100,00	2,97	19,58	16,78	19,06	19,76
trpD	1,90	3,40	2,00	1,40	10,21	11,31	10,31	10,51
trpC	3,70	5,49	3,83	3,32	9,32	10,47	11,37	10,60
trpF	6,45	6,92	7,55	7,39	5,82	9,12	4,56	5,35
trpB	1,64	2,38	1,40	1,64	8,88	9,95	8,80	8,63
trpA	3,02	4,80	3,70	3,43	7,54	12,07	8,50	8,50
femA	1,03	1,43	0,63	0,79	3,88	4,75	2,69	3,56
femB	0,56	2,30	0,95	0,87	4,84	4,92	4,76	4,44
cof	3,86	3,98	3,98	4,11	6,68	14,14	21,47	100,00
Q5HG42	2,50	3,13	100,00	3,54	12,50	14,58	13,54	13,33
nikF	1,71	2,42	100,00	2,42	22,93	13,96	22,51	22,51
nikD	1,16	2,58	1,16	1,42	100,00	8,00	23,23	100,00
nikC	1,07	2,15	2,15	1,07	100,00	8,35	20,64	100,00
nikB	1,52	2,53	1,62	100,00	100,00	10,22	23,38	23,48
Q5HG37	1,16	2,03	1,16	0,87	9,57	4,64	7,83	7,25
pepF2	1,93	2,81	1,49	1,60	7,44	7,49	8,26	100,00
phoU	1,09	2,18	1,09	1,87	8,10	6,39	12,15	12,46
pstB	0,82	2,82	0,70	0,94	5,63	5,05	7,63	8,57
pstA	1,31	3,05	2,40	2,94	8,06	6,54	13,29	13,40
pstC	4,31	1,94	0,65	4,21	10,46	5,93	15,43	15,43
pstS	0,61	0,81	0,20	0,71	8,13	4,67	8,03	7,93
cvfB	1,33	2,21	1,99	100,00	5,43	5,09	5,87	6,53
ykpA	1,00	0,87	1,44	1,12	5,56	4,37	4,37	5,37
lysC	0,91	0,83	0,66	1,58	4,48	5,39	13,76	13,35
asd	1,72	1,52	2,02	100,00	4,65	6,36	10,30	9,80
dapA	1,35	100,00	1,91	2,03	2,82	11,49	14,64	13,63
dapB	5,26	7,61	0,83	0,83	2,21	9,54	10,51	10,79
dapD	2,36	2,64	2,50	2,50	4,86	6,81	9,31	9,86
hipO	2,78	2,52	1,65	1,65	2,95	6,16	13,54	13,63
alr2	1,66	1,84	1,84	1,84	1,66	7,27	13,26	13,81
lysA	3,40	2,05	1,97	2,37	6,79	11,93	13,90	14,14
cspC-L2-msaB	1,00	1,00	1,49	1,49	1,49	1,00	1,00	1,00
msaA	0,32	0,97	0,00	0,32	1,62	1,94	6,15	7,12
acyP	0,74	1,48	0,37	1,11	6,67	4,44	12,59	12,96
xpaC	0,79	1,27	0,63	0,63	7,78	4,29	8,57	8,57
terA	1,32	1,32	0,79	1,06	100,00	5,89	13,81	13,28
brnQ3	2,46	2,46	1,64	1,93	14,88	7,37	14,81	14,81
cobT	1,17	2,12	1,32	0,95	10,28	6,52	12,72	12,98
norQ	1,26	0,88	2,40	1,14	6,44	5,68	10,98	10,98
yozC	1,47	2,45	1,96	1,96	4,41	3,43	10,78	9,80
Q5HG08	1,36	0,86	0,49	0,12	6,42	8,02	11,98	12,22
odhB	0,93	0,70	0,78	0,85	5,36	5,43	11,49	11,80
odhA	1,11	1,29	1,50	1,04	8,18	3,86	11,58	11,75
arlS	1,18	100,00	0,81	1,11	3,83	4,42	15,12	15,19
arlR	1,06	1,21	0,91	0,91	3,48	3,79	11,97	11,67
pgpB	0,65	2,44	0,49	0,33	100,00	6,50	10,24	11,22
murG	1,21	1,03	1,21	1,12	4,30	6,35	13,17	13,45
Q7A0W4	0,20	0,39	0,20	0,39	4,51	3,33	10,98	100,00
ctpA	0,87	0,94	0,80	1,07	3,75	6,96	16,32	16,79
yozE	0,00	0,00	0,00	0,00	1,80	2,70	9,01	100,00
crr	0,60	1,00	0,80	0,80	2,99	3,59	12,18	12,38
msrB	0,00	0,47	0,47	0,00	2,80	5,36	9,32	9,09
msrA2	0,56	0,75	0,19	0,56	1,87	3,93	5,43	5,81
degV2	0,12	0,12	0,12	0,12	8,10	4,88	15,71	15,83
folA	1,04	1,67	0,21	0,63	4,38	9,17	14,58	13,96
thyA-chr	1,78	3,66	1,67	1,67	15,99	11,29	15,36	15,05
cvfC4	0,46	1,37	0,68	2,51	14,38	3,42	11,87	12,79
cvfC3	0,36	100,00	1,33	1,60	21,24	9,33	20,80	20,27

Gene	vs. zoo28	vs. EMCR19	vs. SS60	vs. SS90	vs. NCTC13712	vs. MSHR1132	vs MW2	vs. SA17_S6
cvfC2	0,40	0,40	0,79	0,40	12,30	2,78	12,30	11,51
cvfC1	0,00	0,00	1,03	1,03	6,15	2,56	5,64	5,64
Q5HFZ1-yhhQ	2,55	100,00	100,00	3,40	13,46	8,78	11,61	12,61
rnhA	3,98	3,98	1,99	1,00	7,21	13,93	4,48	16,17
ebh	1,24	1,40	1,28	1,32	3,29	9,15	2,66	9,15
norB	1,00	1,56	1,28	1,49	14,86	14,44	16,36	16,71
steT	1,20	2,41	1,20	1,20	11,06	8,05	12,26	11,81
ilvA1	0,86	1,34	0,58	0,86	13,16	7,01	12,78	12,58
ald1	0,54	1,43	0,98	1,07	12,15	10,63	11,89	11,62
exo53	1,48	1,71	1,59	0,57	6,94	12,40	7,96	8,19
Q2FGZ8	1,19	100,00	1,22	1,07	100,00	10,75	17,52	17,93
piuB	2,83	1,79	2,46	1,86	16,18	6,86	17,08	17,23
Q5HFY0	0,60	100,00	1,49	100,00	100,00	10,45	11,94	10,75
ypsC	1,13	0,96	1,66	1,05	12,30	8,73	11,78	11,95
gpsB	0,29	0,29	0,29	0,00	1,16	0,87	3,77	3,48
ypsA	0,18	0,35	0,53	0,53	4,96	5,14	7,80	7,80
yppE	0,85	1,42	1,42	2,28	100,00	9,69	16,81	16,24
recU	0,59	0,88	0,59	0,59	9,88	2,21	5,90	5,90
pbpB	1,09	100,00	100,00	100,00	5,84	4,51	8,53	8,57
Q1YBU7	1,93	100,00	1,65	1,65	12,40	7,16	18,46	18,46
nth1	0,91	1,21	2,27	2,88	3,79	5,76	11,52	11,82
dnaD	1,60	1,46	1,60	100,00	2,18	4,08	12,52	12,81
asnS	0,93	1,24	0,70	1,01	3,17	3,71	8,43	9,13
dinG1	1,37	1,11	1,04	1,08	3,56	4,83	13,14	13,40
birA	1,23	1,54	0,72	1,44	3,60	4,32	11,21	11,32
cca-papS	0,67	1,25	0,91	0,83	17,37	6,48	18,70	18,45
bshA	0,87	0,87	1,05	0,35	9,62	5,07	13,39	12,86
ypjD	2,20	1,26	1,26	1,57	11,32	3,46	15,41	15,09
yugP	0,85	0,56	0,14	0,85	4,66	1,69	4,52	4,52
ypjA	1,02	1,02	100,00	1,02	20,24	6,63	20,07	20,75
ypiB	1,03	0,86	0,69	2,24	20,86	8,45	21,03	20,86
ypiA	1,69	2,65	1,04	1,29	10,52	4,02	12,61	12,45
aroA	1,31	1,15	1,69	1,46	6,62	6,47	12,70	15,09
aroB	0,94	1,41	2,25	0,94	6,01	7,51	14,93	18,03
aroC	1,03	1,03	0,69	1,20	1,63	7,11	5,74	11,74
ndk	0,44	0,44	0,67	0,44	4,00	5,56	4,89	5,33
gerCC	0,94	0,94	1,35	1,25	6,04	4,79	13,33	15,10
ubiE	1,24	0,83	1,65	1,24	4,55	4,41	10,47	10,47
gerCA	0,87	0,52	0,70	0,70	2,79	2,62	9,08	9,08
hup	0,00	0,00	0,00	0,00	0,00	0,00	0,73	0,73
gpdA	1,00	0,70	1,20	1,10	11,41	6,41	12,41	12,21
engA	0,92	1,14	1,30	1,37	5,03	3,05	9,61	9,92
rpsA	1,19	0,60	0,77	0,60	3,06	3,91	9,18	9,27
Q2FYF8	0,00	0,52	100,00	0,00	100,00	2,06	0,52	100,00
cmk	0,76	1,21	0,76	0,61	1,06	3,94	9,24	10,15
ansA	0,93	1,34	1,03	0,72	6,30	8,67	15,17	15,07
ypdA	0,71	0,91	1,01	0,91	9,73	5,88	11,55	11,55
ebpS	0,34	1,89	1,28	1,55	8,63	10,45	25,02	25,62
recQ2	1,45	2,24	100,00	1,67	100,00	9,99	17,52	17,74
A5IT10	2,09	0,94	100,00	2,30	9,83	100,00	13,91	13,91
fer	1,20	0,80	0,80	1,61	3,61	1,61	4,42	4,42
ribU	1,83	1,83	2,93	2,20	7,69	10,26	7,14	7,88
A5IT17	100,00	100,00	100,00	100,00	100,00	100,00	5,40	10,28
srrB	2,00	1,66	1,31	1,77	5,19	5,94	4,51	4,68
srrA	0,69	0,55	0,41	0,69	2,07	100,00	1,79	1,79
rluB	0,81	2,44	1,36	1,08	10,03	3,66	11,38	11,38
scpB	1,10	1,66	0,74	2,03	3,87	4,42	8,29	8,10
scpA	0,41	0,68	0,41	0,82	2,32	3,69	3,14	3,83
Q2FY75	1,57	1,57	0,00	0,20	100,00	8,86	19,69	20,28
xerD	0,90	1,58	1,01	0,79	4,73	7,32	13,29	13,51
fur	0,22	1,11	0,44	0,00	2,22	3,78	6,89	6,89
nudF	1,10	1,10	2,58	2,21	9,94	9,02	8,47	8,10
yhdN	2,08	2,08	1,75	1,86	8,96	7,65	12,02	11,91
Q5HFS1	3,60	2,40	3,20	2,00	100,00	22,40	10,80	100,00
yqjQ	3,04	4,37	3,17	1,85	10,71	14,15	5,69	7,94
proC	1,10	1,59	0,98	1,72	9,19	6,99	12,01	11,40
rnz	4,89	1,74	3,04	3,69	5,65	5,32	10,21	9,23
zwf	0,74	0,81	0,40	0,87	4,63	5,37	9,00	8,73
graE	0,35	0,23	0,35	0,35	3,34	3,69	3,92	100,00
malA-yugT	4,61	2,24	2,36	100,00	10,67	10,00	9,70	10,06
malR-kdgR	1,27	1,76	2,25	1,76	15,98	17,45	9,61	10,98
Q5HFR3	2,15	2,96	2,42	2,42	5,38	4,84	1,34	1,88
Q931R4	100,00	100,00	100,00	100,00	100,00	100,00	100,00	100,00
gnd	1,42	2,06	0,85	0,85	4,76	5,54	8,74	9,03
Q5HFR1-pepT2	1,49	3,41	1,92	1,92	100,00	7,79	18,29	18,46
yqjA	0,61	0,92	0,61	0,71	1,33	3,16	13,86	13,56
yqiW	2,97	0,68	0,46	1,14	2,97	5,25	10,73	11,19
bfmBB	2,03	0,94	0,47	1,87	2,34	5,70	18,81	18,58
bfmBAB	0,61	0,41	0,51	0,61	8,33	3,96	11,59	11,59
bfmBAA	0,70	1,30	0,60	0,60	9,01	4,30	14,61	14,51
lpdA	0,84	1,34	0,84	0,98	3,59	4,64	14,77	14,91
recN	0,30	1,01	0,60	0,42	3,93	3,99	13,81	100,00
ahrC	0,66	0,44	0,44	0,44	2,65	1,99	8,61	8,17
ispA	0,91	1,47	0,34	0,23	5,22	5,56	21,54	20,86
xseB	0,43	0,87	0,43	0,43	1,73	2,16	6,49	6,49
xseA	0,75	1,20	1,12	0,97	4,71	3,96	13,83	13,83
nusB	0,26	0,77	0,26	0,26	4,10	2,56	11,03	11,28
yqhY	0,55	0,28	0,28	0,55	2,20	1,65	5,79	6,06
accC	0,66	0,59	0,59	0,66	4,19	3,24	10,45	10,74
accB	0,22	0,43	0,00	0,22	1,72	1,72	9,46	9,68
efp	0,36	0,36	0,36	0,72	1,08	1,43	5,20	5,20

Gene	vs. zoo28	vs. EMCR19	vs. SS60	vs. SS90	vs. NCTC13712	vs. MSHR1132	vs MW2	vs. SA17_S6
yqhT	2,17	1,22	1,13	1,13	5,74	6,21	14,97	14,97
Q5HFM8	2,56	0,68	0,68	0,68	100,00	4,95	12,80	100,00
Q5HFM7	0,91	0,46	0,46	0,46	3,65	2,28	13,70	13,24
lipM	0,96	0,84	0,24	0,12	5,54	4,57	13,60	13,12
yqhL	0,52	0,52	0,52	0,26	1,55	2,07	5,17	5,17
gcvPB	3,80	2,58	4,34	5,09	6,92	5,70	11,95	12,08
gcvPA	1,11	1,78	1,19	0,97	4,45	4,53	10,69	11,14
gcvT	1,01	1,19	0,64	1,10	3,75	3,48	10,99	11,08
aroK	0,76	100,00	0,57	0,57	4,76	4,19	19,43	18,48
comGF	9,44	0,80	0,60	0,80	15,86	8,23	100,00	100,00
comGE	0,87	0,58	0,58	0,87	8,99	6,38	100,00	100,00
comGD	2,91	2,46	0,67	2,24	8,95	100,00	24,16	23,94
comGC	1,60	1,60	0,32	100,00	5,77	9,29	17,95	18,27
comGB	2,33	2,24	1,03	2,15	5,70	8,78	22,32	22,88
comGA	0,51	0,62	0,51	0,51	5,64	5,95	14,26	14,56
yqgX	0,32	0,16	0,16	0,16	12,98	13,78	4,01	4,81
yqgV	0,61	1,52	0,00	0,30	100,00	9,09	5,15	5,45
glk	1,52	1,32	0,41	1,72	4,26	5,27	12,06	11,85
yqgQ	0,49	0,98	0,49	0,98	2,94	1,47	7,35	7,35
gluP	0,34	1,37	0,34	1,78	4,23	4,23	12,36	12,50
yqgN	0,56	1,48	0,93	0,74	6,48	5,37	19,07	20,74
rpmG1	0,67	0,67	0,67	0,67	2,00	1,33	4,00	4,00
pbpC	0,67	0,96	0,82	0,82	4,14	4,14	13,54	13,25
sodA-L2	0,17	0,17	0,66	0,66	1,33	2,33	5,65	100,00
zur	1,22	0,00	0,49	0,00	2,92	2,92	10,22	100,00
znuB	4,16	0,81	0,58	0,35	10,40	8,44	11,68	100,00
znuC	2,16	0,64	0,13	2,67	5,22	4,71	11,83	100,00
nfo	0,34	0,45	0,34	1,23	3,25	3,59	9,88	100,00
cshB	0,97	1,34	0,97	1,71	3,86	4,01	9,21	100,00
ybgI	1,72	100,00	1,63	1,91	7,08	6,99	20,05	100,00
trmK	0,74	1,77	0,44	0,74	5,31	4,13	17,26	100,00
sigA	1,54	1,63	1,63	1,26	2,89	2,35	6,78	6,87
dnaG	1,05	1,76	1,05	2,70	3,74	5,28	15,18	14,08
yqfL	1,83	1,34	1,83	1,71	4,76	4,64	14,29	14,41
ccpN	0,16	0,64	0,16	0,16	2,08	2,72	11,54	11,54
glyS	1,08	1,51	0,72	1,22	2,80	5,96	7,26	7,61
recO	0,53	1,33	1,06	0,66	1,86	4,52	15,54	15,54
era	1,00	1,11	1,00	0,67	1,56	3,67	12,78	12,78
cdd	0,99	0,99	0,74	0,74	0,99	4,69	11,11	10,86
dgkA	0,58	0,58	1,16	0,58	2,03	4,93	15,07	13,91
ybeY	0,64	1,07	0,43	0,85	5,34	3,21	13,89	13,46
phoH	0,11	0,63	0,42	0,00	0,95	7,17	16,14	16,14
Q5HFI8	0,72	1,43	0,29	0,86	1,00	10,01	6,01	6,87
floA	0,20	0,81	0,51	0,40	2,32	8,69	3,74	4,04
yqeZ	0,13	0,13	0,13	0,13	10,36	9,03	11,16	5,58
rpsU	0,00	0,00	0,00	0,00	0,00	0,00	0,00	0,00
mtaB	1,04	0,45	1,04	0,82	0,89	5,42	12,84	13,21
rsmE	0,93	0,93	0,93	0,93	2,52	6,77	15,14	14,74
prmA	1,28	2,34	1,28	1,17	4,37	4,37	11,08	11,82
dnaJ	0,44	0,35	0,44	0,44	3,33	4,30	11,05	11,23
dnaK	0,22	0,27	0,22	0,33	2,24	3,00	8,73	8,73
grpE	0,32	0,64	0,32	0,32	3,19	3,03	8,93	8,77
hrcA	0,41	0,92	0,41	0,61	3,58	3,58	7,16	7,06
hemN	0,52	0,78	0,43	0,60	5,27	5,27	20,47	21,24
lepA	0,27	1,54	0,27	0,27	4,11	4,44	11,29	11,40
rpsT	0,40	0,40	0,40	0,40	2,78	1,59	2,78	2,78
holA	0,31	1,54	0,92	0,31	4,10	11,90	12,92	12,92
comEC	1,59	4,22	2,45	1,54	8,04	29,79	100,00	29,75
comEB	0,43	1,95	0,43	0,43	11,04	10,61	10,17	9,96
comEA	5,66	6,49	6,91	5,52	8,15	29,14	25,28	25,28
yqeM-cypM	1,81	100,00	1,67	1,67	5,44	7,11	19,11	18,69
rsfS	0,28	0,28	0,00	0,28	4,24	3,11	11,30	10,73
yqeK	0,68	1,03	0,34	0,00	1,03	2,39	10,94	10,77
nadD	0,18	0,53	0,00	0,18	0,70	4,04	17,54	17,72
yhbY	0,00	1,03	0,00	0,00	0,69	1,72	10,31	10,65
aroE	0,50	1,36	0,50	0,50	2,60	6,44	15,99	15,99
yqeH	0,27	0,27	0,91	0,36	4,09	5,36	12,44	12,17
yqeG	0,94	1,13	100,00	100,00	100,00	4,53	10,00	10,19
mtnN	0,87	1,02	0,87	0,15	3,49	4,22	10,19	10,63
Q5HFG1	0,74	0,00	0,37	0,00	100,00	8,52	21,48	21,85
entX	0,00	0,00	0,00	100,00	0,00	0,00	100,00	100,00
ycsG	2,03	2,60	1,22	1,62	5,92	8,52	12,81	12,90
ycsF	1,06	2,39	0,66	0,80	2,92	5,05	16,07	16,07
accC2	100,00	100,00	100,00	100,00	0,00	0,00	0,00	0,00
accB2	3,10	3,33	2,88	2,88	5,32	10,42	26,39	26,61
kipA	1,29	3,26	1,19	1,48	3,36	7,81	24,53	25,02
kipI	0,95	1,36	1,63	0,82	1,63	8,03	21,63	100,00
greA	0,42	0,63	0,63	0,21	3,56	4,82	11,11	10,48
udk	0,80	1,12	0,16	0,00	2,88	3,21	7,69	7,53
yrrO	0,79	1,89	1,34	0,87	2,76	3,31	8,90	8,67
yrrN	0,54	0,76	0,43	0,54	2,60	3,25	10,93	10,93
yrrM	0,94	0,94	100,00	100,00	4,84	3,91	10,78	11,56
yrzB	0,65	1,62	1,29	1,94	1,62	4,53	7,77	7,77
yrrK	0,47	0,47	0,93	0,47	1,40	1,17	6,53	6,53
yrzL	0,38	0,00	0,77	0,38	1,15	1,15	5,75	5,75
alaS	1,60	1,22	1,06	0,80	2,09	4,14	10,45	10,68
recD	1,85	1,65	1,61	1,69	1,85	5,58	15,94	15,78
yrrB	0,30	1,64	0,75	0,30	0,60	6,28	15,99	15,70
mnmA	1,88	1,61	1,70	1,79	4,11	5,99	12,24	12,33
csd1	1,49	2,10	1,49	1,31	4,46	5,25	12,42	12,69
limB2	1,18	1,78	1,18	1,08	2,76	7,30	18,54	18,74

Gene	vs. zoo28	vs. EMCR19	vs. SS60	vs. SS90	vs. NCTC13712	vs. MSHR1132	vs MW2	vs. SA17_S6
Q5HFD8	0,00	0,00	0,00	0,00	0,68	1,36	14,29	14,29
csbD-L2	1,09	1,64	1,64	1,09	1,09	5,46	9,84	9,84
cymR	0,00	0,47	0,00	0,00	2,36	2,84	8,75	8,27
rarA	1,18	1,73	0,63	1,02	2,35	5,02	14,20	13,88
yrvM	2,84	1,68	1,03	2,20	0,65	3,75	12,40	12,02
aspS	0,79	1,64	0,85	0,85	2,55	5,26	10,75	10,81
hisS	1,03	1,11	0,95	0,95	1,98	4,83	11,01	11,32
lytH	0,68	1,03	0,57	0,57	3,77	2,51	10,05	10,39
dtd	1,55	1,99	1,55	1,77	3,97	9,05	14,13	13,25
relA	1,28	1,28	1,14	1,10	4,16	5,48	11,64	11,46
apt	1,16	1,54	0,96	1,16	3,28	1,93	12,33	12,33
recJ	1,36	1,41	1,10	1,06	5,63	5,06	16,18	16,40
secDF	0,57	1,05	0,57	0,66	3,29	3,29	11,40	11,36
yajC	0,77	0,77	0,77	0,77	2,30	3,07	4,98	4,98
tgt	0,09	0,79	0,09	0,18	2,28	2,28	6,05	5,88
queA	1,95	0,68	1,95	0,88	5,65	6,04	11,40	12,09
ruvB	1,49	1,59	1,49	1,59	4,48	4,88	11,74	11,74
ruvA	1,66	1,82	1,82	0,66	3,81	4,15	12,27	12,60
pheB	0,65	0,87	0,44	1,31	3,05	1,74	12,64	12,64
obgE	0,62	1,01	0,62	0,46	4,25	4,49	11,52	11,76
rpmA	0,70	0,70	0,70	0,70	0,70	0,70	1,05	1,05
DUF464	0,00	0,31	0,00	0,31	1,87	1,25	2,80	2,80
rplU	0,00	0,00	0,00	0,97	1,62	0,32	1,62	1,94
mreD	0,94	1,69	0,94	0,94	3,95	5,46	16,38	16,76
mreC	0,95	1,30	1,07	1,30	3,80	3,56	11,63	11,74
Q5HFB3	0,84	1,48	0,84	0,84	1,48	4,43	14,35	15,19
Q5HFB2	0,35	100,00	0,35	0,00	0,35	4,91	18,95	18,95
radC	0,73	0,87	0,87	1,46	4,66	5,39	18,49	18,20
comC-outO	1,27	100,00	0,99	0,71	6,21	5,65	27,54	27,26
folC	0,71	1,10	0,55	0,63	100,00	4,08	15,16	100,00
valS	0,99	1,41	0,95	0,68	3,57	4,71	10,34	10,34
tag	2,50	2,32	0,36	0,18	2,14	6,24	14,80	15,86
abrB	1,21	3,16	0,37	1,02	3,81	6,70	20,56	20,74
gsaA	1,63	1,48	1,48	1,63	4,82	4,58	12,43	12,28
hemB	1,64	2,67	1,95	1,64	7,18	5,64	11,79	11,18
hemD	0,75	3,14	1,79	0,90	6,43	6,88	17,94	18,39
hemC	1,19	3,45	1,19	0,97	5,72	6,36	14,35	14,35
hemX	0,86	1,96	0,98	0,49	3,43	2,70	11,40	12,01
hemA1	0,37	1,56	0,82	0,45	3,04	2,75	7,87	7,72
engB	0,51	0,85	0,85	0,51	7,95	7,28	13,87	14,04
clpX	0,79	100,00	0,87	0,87	4,28	3,40	9,03	8,95
tig	0,46	1,00	0,46	0,46	1,92	3,30	7,30	7,30
ysoA	0,97	2,37	1,08	1,18	6,88	6,02	22,37	22,15
ymaB	0,49	2,13	0,49	0,49	4,93	3,94	12,64	12,48
rplT	0,00	1,12	0,00	0,00	0,84	1,12	2,52	2,52
rpmI	0,00	0,00	0,00	0,00	2,49	1,49	1,00	1,00
infC	0,19	0,38	0,19	0,38	11,55	4,73	4,73	4,73
lysP1	0,67	4,55	1,14	2,01	10,44	8,90	11,18	11,11
thrS	1,55	1,60	2,58	0,62	5,47	5,52	10,11	10,84
dnaI	1,19	1,09	1,09	0,76	1,41	3,91	15,96	16,29
dnaB	1,07	1,28	1,00	1,35	4,55	5,90	17,20	17,41
nrdR	0,21	0,42	0,21	0,21	1,27	1,49	8,07	8,07
gapB	0,78	0,49	0,68	0,88	4,39	5,17	13,06	12,96
coaE	1,12	0,64	0,96	1,12	2,56	2,88	15,22	15,54
mutM	3,78	4,47	4,12	3,44	4,93	4,24	14,89	14,43
polA	2,20	1,71	2,09	100,00	4,52	5,17	14,48	14,29
Q2YTE9	2,68	0,87	2,48	0,87	100,00	100,00	17,92	17,99
phoR	0,96	1,68	0,72	1,32	5,66	6,32	16,19	16,25
phoP	0,28	0,71	0,28	0,99	3,26	2,70	8,37	8,51
citC	0,39	1,50	0,24	0,24	3,39	3,62	9,30	9,54
citZ	0,89	0,62	0,71	0,89	3,48	5,53	9,54	9,71
aapA1	0,88	0,81	0,37	0,37	3,88	2,86	12,97	13,11
pykA	0,51	0,74	0,17	0,28	1,99	2,79	7,17	7,17
pfkA	0,41	0,83	0,41	0,10	3,30	3,30	7,33	7,64
accA	1,27	2,86	1,27	0,95	3,60	3,49	11,75	11,64
accD	0,00	2,33	0,12	0,00	2,80	3,50	8,62	8,28
maeB	0,89	1,38	0,89	1,30	3,25	3,90	13,17	13,66
dnaE	1,03	1,00	0,69	0,53	4,97	5,35	18,11	18,23
nrnA	1,80	2,12	0,96	0,64	4,99	4,35	18,26	100,00
ytoI	1,39	1,39	1,54	1,00	3,77	4,39	12,70	13,09
uspA	0,48	0,48	0,24	1,69	100,00	4,35	9,90	10,14
ytkL	0,87	1,74	1,59	100,00	4,34	5,93	11,29	100,00
pepQ	0,66	0,95	2,08	1,52	4,64	6,16	13,73	14,20
ald2	0,63	1,16	0,98	0,89	8,85	5,99	12,60	12,87
yxiE	0,00	0,20	0,00	0,00	3,59	0,80	4,79	4,39
ackA	0,91	1,50	1,33	1,25	3,91	3,91	7,23	7,23
ytxK	0,32	0,32	0,21	0,00	3,90	3,90	10,97	10,65
tpx	0,81	0,81	1,01	1,41	4,24	2,63	10,10	10,91
ytnM	1,29	1,16	0,90	1,54	5,41	5,66	18,53	18,28
thiI	0,57	0,33	0,74	0,65	4,49	4,41	13,73	13,24
csd2	1,57	1,57	1,48	1,22	4,96	6,01	19,84	20,02
ezrA	0,53	0,77	0,47	0,29	2,65	3,19	8,97	8,85
ytsP-msrC	0,22	1,94	0,22	0,22	100,00	6,24	14,62	15,27
rpsD	0,00	0,17	0,00	0,00	2,16	0,50	1,99	2,16
ugpQ2	1,61	100,00	1,48	1,88	23,92	22,18	12,77	11,69
osmC	2,01	4,25	2,01	2,01	100,00	25,50	8,28	8,72
pucG	1,72	1,89	1,81	0,95	5,68	7,92	14,81	14,47
serA	0,50	100,00	1,62	0,87	5,67	6,16	16,44	16,44
Q5HF49	2,56	100,00	1,94	2,29	100,00	6,18	20,83	100,00
nagE	0,89	2,93	1,30	1,09	12,13	6,41	11,04	11,18
plsC	0,16	0,49	0,16	0,16	6,15	4,53	11,17	11,49

Gene	vs. zoo28	vs. EMCR19	vs. SS60	vs. SS90	vs. NCTC13712	vs. MSHR1132	vs MW2	vs. SA17_S6
htrC	0,63	0,94	0,39	0,78	9,10	4,00	11,92	12,16
tyrS	100,00	100,00	0,24	100,00	9,96	6,96	10,99	11,46
sgtA	0,22	0,99	0,44	0,33	15,01	8,06	14,24	14,35
isdH-harA	2,36	4,89	2,32	2,74	27,82	26,13	22,86	100,00
fhs	0,72	1,50	0,54	0,72	11,33	6,29	10,37	10,37
acsA1	0,94	1,82	1,05	1,11	8,14	6,15	15,41	15,58
acuA	0,47	0,79	0,47	0,79	6,16	8,06	16,75	17,06
acuC	0,60	1,97	0,51	1,03	4,79	6,84	17,35	100,00
ccpA	0,71	0,81	0,81	0,81	2,93	8,69	11,52	11,82
aroA2	0,73	1,56	0,73	0,64	1,47	2,93	10,16	10,53
Q99TC6	12,82	13,26	12,75	13,13	100,00	100,00	29,67	100,00
yoxC	0,61	0,81	0,41	0,61	1,83	2,03	3,25	3,86
murC	0,38	0,76	0,23	0,46	2,82	3,58	7,69	8,37
Q5HF33	1,66	1,99	1,46	1,08	7,38	7,08	25,96	26,06
pheT2	0,67	0,67	0,50	0,50	3,85	3,35	13,23	13,23
Q5HF31	1,28	0,70	0,82	0,58	3,15	2,91	11,42	11,19
ytpP	0,64	1,60	0,64	0,00	2,88	3,85	11,22	10,90
pepA1	2,88	2,69	2,97	2,04	4,36	4,36	14,67	14,30
ytzB	1,92	1,60	1,28	1,60	2,88	2,88	9,29	8,65
ytnP	2,60	0,95	2,49	2,13	19,53	7,69	19,05	18,93
trmB	0,31	1,09	0,31	0,31	11,47	4,65	12,09	11,47
ytmP	0,38	1,39	0,25	0,00	8,96	2,53	8,96	8,59
daaA	0,59	1,18	0,59	0,71	13,43	5,42	13,31	13,43
ytjP	0,50	1,06	0,50	0,85	5,74	3,90	11,21	10,85
Q5HF22	1,65	2,60	1,65	2,13	11,35	1,89	16,31	15,37
rsuA	2,16	2,30	1,72	100,00	11,93	4,17	17,67	16,95
ytgP	0,60	0,48	0,42	0,36	3,01	3,31	10,83	10,95
Q1Y9Y5	1,65	2,59	1,89	1,49	18,32	8,33	18,16	18,79
sasC	4,19	4,39	4,27	4,09	12,27	11,42	12,16	13,67
ytwF-moeB	17,95	17,63	18,27	16,03	100,00	16,99	9,62	9,62
leuS	7,93	6,77	8,01	8,10	8,80	9,50	3,72	3,97
yttB	2,20	2,71	1,95	0,76	4,91	7,11	11,42	11,25
ytqA	0,84	2,20	0,63	0,52	4,61	5,56	12,26	12,68
ytqB	2,13	0,89	2,13	1,95	6,74	7,09	19,33	18,62
rot	1,49	0,75	0,50	0,75	3,23	2,24	11,94	11,19
pldB1	0,85	1,09	0,48	0,85	2,42	4,47	10,39	100,00
putA	1,60	2,40	0,90	1,40	4,99	9,88	15,57	100,00
ribH	1,72	1,72	1,94	3,66	6,24	10,97	17,63	100,00
ribA	1,27	1,69	0,76	1,69	4,48	6,77	15,06	100,00
ribB	0,32	1,26	0,63	2,05	5,06	4,58	15,80	100,00
ribD	0,86	2,20	1,25	1,05	3,83	4,60	14,27	100,00
Q5HF04	1,26	100,00	1,73	0,53	100,00	6,18	19,02	100,00
arsR	100,00	100,00	100,00	0,60	12,50	13,99	8,93	100,00
arsB	1,93	2,40	3,63	100,00	12,67	13,91	12,44	100,00
arsC	5,52	100,00	5,76	100,00	100,00	15,59	100,00	100,00
sagB-gad	0,58	1,28	1,52	0,70	6,64	2,45	7,69	7,93
Q5HEZ9	0,00	0,00	0,00	0,00	1,33	1,78	5,78	5,78
sigS	1,47	0,63	1,68	2,31	6,50	2,94	19,29	18,87
Q5HEZ7-comK	0,45	1,35	1,12	1,80	100,00	7,42	22,70	22,70
Q5HEZ6	0,68	2,03	1,80	3,15	6,31	9,01	20,72	20,95
Q5HEZ5	1,84	100,00	1,84	1,53	100,00	3,53	100,00	100,00
tal	1,68	100,00	1,68	0,70	4,76	1,54	10,50	9,80
Q7A500	1,98	1,98	1,98	1,98	4,95	3,30	12,87	13,53
crcB1	2,45	21,16	2,45	2,67	23,83	100,00	100,00	100,00
crcB2	2,82	1,69	2,26	3,11	7,91	6,50	25,42	100,00
ytbE-yvgN	3,48	1,08	2,16	1,68	100,00	7,55	13,79	14,51
nrd	0,99	0,99	1,54	1,43	2,75	3,96	5,17	5,17
metK	1,34	0,84	0,75	0,75	4,52	3,18	7,37	7,62
pckA	1,19	1,76	0,88	1,32	5,21	5,02	7,85	7,60
ytmA	3,26	100,00	100,00	100,00	100,00	14,72	14,48	17,13
rppH	2,02	2,63	1,21	2,22	16,97	17,37	18,18	17,98
ytjA-yidD	1,94	0,39	0,00	1,55	100,00	7,36	8,91	9,69
menC	3,69	7,88	4,49	2,69	13,17	11,08	15,57	15,97
menE	3,85	4,60	2,70	2,30	10,75	10,48	12,24	12,58
Q5HEY1	1,24	2,07	1,24	1,65	8,26	14,46	26,24	25,62
Q5HEY0	3,84	7,37	3,69	0,92	3,07	6,45	28,73	27,80
DUF4352	1,79	2,68	100,00	3,97	9,83	6,16	6,85	10,23
Q5HEX8	2,07	2,55	0,80	3,03	6,06	9,73	7,02	9,89
Q5HEX7	0,87	100,00	1,45	1,73	100,00	7,80	4,05	6,65
A5ITW8	0,87	100,00	1,22	100,00	100,00	6,09	6,26	6,96
hsdS-spl	0,00	100,00	0,00	0,00	0,00	0,00	100,00	0,00
hsdM-spl	0,00	0,00	0,00	0,00	0,00	100,00	100,00	0,00
splF	0,00	100,00	0,00	0,00	0,00	0,00	100,00	0,00
splE	0,00	100,00	0,00	0,00	0,00	0,00	0,00	0,00
splD2	0,00	100,00	0,00	0,00	0,00	0,00	0,00	0,00
splC	0,00	100,00	0,00	0,00	0,00	0,00	100,00	0,00
splB	0,00	100,00	0,00	0,00	0,00	0,00	100,00	0,00
splA	0,00	0,00	0,00	0,00	0,00	0,00	100,00	0,00
Q2FXC0-ear2	100,00	100,00	100,00	100,00	100,00	100,00	100,00	0,00
epiG	100,00	0,00	100,00	100,00	100,00	100,00	100,00	100,00
epiE	100,00	0,00	100,00	100,00	100,00	0,00	100,00	100,00
epiF	100,00	0,00	100,00	100,00	100,00	0,00	100,00	100,00
epiP	100,00	0,00	100,00	100,00	100,00	0,00	100,00	100,00
epiD	100,00	0,00	100,00	100,00	100,00	0,00	100,00	100,00
epiC	100,00	0,00	100,00	100,00	100,00	0,00	100,00	100,00
epiB	100,00	0,00	100,00	100,00	100,00	0,00	100,00	100,00
epiA	100,00	0,00	100,00	100,00	100,00	0,00	100,00	100,00
bsaX	0,00	0,00	0,00	0,00	0,00	0,00	100,00	0,00
lukD	100,00	0,00	100,00	100,00	0,00	100,00	100,00	100,00
lukE	100,00	100,00	100,00	100,00	0,00	0,00	100,00	100,00
Q7A4X2	0,00	0,00	0,00	0,00	0,00	100,00	27,01	27,39

Gene	vs. zoo28	vs. EMCR19	vs. SS60	vs. SS90	vs. NCTC13712	vs. MSHR1132	vs MW2	vs. SA17_S6
entG	0,00	0,00	100,00	0,00	100,00	100,00	0,00	0,00
entN	0,00	0,00	100,00	0,00	100,00	100,00	0,00	0,00
entU	0,00	0,00	100,00	0,00	100,00	100,00	0,00	0,00
entI	0,00	0,00	100,00	0,00	100,00	100,00	0,00	0,00
entM	0,00	0,00	0,00	0,00	100,00	100,00	0,00	0,00
entO	0,00	0,00	100,00	0,00	100,00	100,00	0,00	0,00
ydeN	1,08	1,80	1,98	0,54	9,91	9,19	8,47	9,37
hemY	3,64	3,71	2,78	3,14	10,42	8,99	11,35	12,56
hemH	1,73	1,73	2,16	1,95	7,25	5,84	10,82	11,36
hemE	0,48	0,77	0,19	0,48	5,97	4,14	10,12	10,40
traP	1,39	2,98	1,98	1,79	3,37	3,97	3,37	14,88
ecsB	0,98	1,72	1,14	1,06	7,35	6,62	16,26	16,34
ecsA	1,21	0,67	0,94	1,48	2,70	2,02	11,20	11,07
hit	0,47	0,71	0,24	0,47	1,89	2,84	7,57	7,80
gvpP	0,82	0,82	0,82	0,55	3,28	4,92	11,48	100,00
yhaJ	0,90	1,61	0,72	1,08	3,41	2,87	11,29	10,57
prsA2	0,73	0,52	0,73	0,62	3,74	3,32	13,40	13,29
cbf	0,96	0,64	0,74	0,64	2,55	2,97	6,37	5,94
Q5HET2	1,43	1,26	1,53	1,57	3,47	4,09	11,20	11,20
Q5HET1	0,08	0,08	0,58	0,92	3,84	2,59	9,27	9,44
UPF0342	0,29	0,58	0,00	0,00	1,45	0,87	3,77	3,77
UPF0754	3,64	1,78	1,07	2,49	18,04	7,38	19,56	19,29
xdrA	1,72	0,86	1,29	1,51	1,94	2,15	3,01	3,23
airR	0,80	0,80	0,80	0,96	12,02	4,17	12,18	12,66
airS	1,26	0,90	0,54	0,54	16,53	5,75	16,71	16,98
rluA2	1,95	1,82	2,19	1,22	10,95	6,20	14,11	14,36
fumC-citG	2,16	1,80	2,89	1,66	11,26	4,04	11,54	11,69
Q5HES3	0,25	1,99	100,00	100,00	20,84	8,19	19,11	19,35
Q5HES1	0,64	0,00	100,00	100,00	6,37	8,92	7,01	8,28
Q5HES0	0,50	0,50	1,50	1,50	9,67	2,50	10,00	10,17
cspR-trmL	0,85	100,00	1,70	0,64	5,10	3,82	11,89	12,31
queG	2,03	3,01	0,71	1,15	9,02	7,25	12,20	12,20
artR	0,82	4,80	1,10	0,96	5,49	3,02	12,21	11,80
artQ	2,95	100,00	2,88	2,13	5,83	5,35	12,48	12,41
Q1Y9N4	1,88	1,32	1,69	2,54	100,00	5,56	16,57	16,95
perR	0,45	0,67	1,12	0,45	4,47	1,79	5,37	5,37
Q5HER2	0,53	2,84	100,00	0,84	10,09	9,88	8,73	9,15
bcp	1,75	0,66	12,94	5,04	7,46	11,84	3,73	5,26
gsaB	1,09	2,25	3,10	2,71	10,39	4,96	10,39	10,23
DUF939_L1	0,37	5,48	2,37	0,55	1,10	9,04	0,73	0,91
msbA2	0,98	0,46	0,40	1,21	11,11	7,02	10,77	10,54
DUF402	0,55	1,10	0,37	1,10	5,89	1,84	9,21	8,47
mutY	0,96	0,87	1,35	2,21	5,20	5,87	15,30	100,00
DUF457	0,41	1,23	0,72	0,72	4,70	5,32	12,88	12,88
tagG	2,12	1,88	1,30	100,00	5,65	5,42	21,67	21,55
tagH	1,65	1,38	1,05	1,38	5,20	4,41	13,04	13,11
yfhH	0,63	1,27	0,32	0,32	0,95	1,27	8,25	7,94
recX	2,08	1,22	2,32	2,32	13,06	4,40	15,14	15,51
sgtB	0,49	1,23	0,49	0,62	3,09	3,21	10,00	10,37
pfpI	0,96	100,00	0,96	0,96	3,08	5,20	12,33	13,10
Q5HEP8	0,00	0,62	0,00	0,00	1,23	1,85	8,64	8,64
A5IU43-yfkAB	0,78	0,95	0,78	0,26	5,70	6,99	14,94	14,85
A5IU44	0,19	100,00	0,00	0,00	1,51	2,07	10,92	11,68
ampS	1,76	2,16	1,60	1,52	6,65	6,73	17,07	17,31
Q5HEP4	0,00	0,00	0,00	0,00	7,25	2,42	11,11	7,73
ptpA	1,08	0,86	1,08	0,86	4,09	3,23	7,96	7,53
Q5HEP2	0,36	1,45	0,36	1,09	3,99	6,16	5,43	5,80
rbn	0,74	0,98	1,23	0,98	14,62	5,07	18,06	17,73
vraR	1,11	1,11	1,11	1,27	2,54	3,81	9,21	9,05
vraS	0,48	0,77	0,67	0,38	5,46	4,21	10,92	10,82
vraT	0,71	1,14	0,71	1,14	5,27	4,27	12,82	12,68
vraU	0,00	1,03	0,26	0,00	1,29	5,43	10,85	11,37
ampM	1,19	1,98	1,58	1,58	3,82	7,64	12,91	12,52
DUF939_L2	1,52	1,22	1,52	2,84	10,54	5,98	14,08	14,29
Q5HEN4	2,16	100,00	0,96	1,44	100,00	100,00	9,59	100,00
gatD	2,46	3,69	2,32	2,60	5,46	6,28	10,38	10,93
murT	1,07	0,99	0,68	1,29	5,78	4,34	9,89	10,43
ftnA	1,60	0,40	1,40	2,00	6,79	2,99	9,58	9,98
dinG2	0,54	1,62	1,44	0,72	3,96	2,88	11,71	11,53
dinB	2,05	1,31	2,52	2,15	13,35	4,67	14,75	14,01
Q5HEM6	3,95	0,94	3,77	4,33	100,00	1,88	17,33	17,51
rlmCD	1,40	1,10	1,32	1,69	12,19	4,92	13,66	14,10
dagK	1,37	1,69	100,00	1,37	4,01	3,48	11,92	12,03
gatB	0,56	1,05	0,63	0,42	6,30	2,80	6,72	6,23
gatA	1,51	1,78	1,37	1,44	1,92	3,29	9,47	9,40
gatC	0,66	0,00	0,33	0,00	0,33	0,99	2,97	2,97
putP	0,78	0,97	1,30	0,84	3,77	5,07	12,80	12,35
camS	0,91	1,66	0,83	0,83	5,89	5,97	9,37	9,29
ligA	0,30	1,05	0,85	1,05	5,79	6,84	13,27	13,37
pcrA	0,55	1,55	0,68	0,68	4,42	6,25	10,40	10,81
pcrB	0,58	0,87	0,58	0,58	4,04	7,79	14,14	14,00
yerC	0,00	0,00	0,00	0,00	0,99	1,32	5,28	5,28
purB	1,00	4,16	1,08	0,69	3,85	4,46	10,08	10,32
sspP	0,00	0,00	0,00	0,00	100,00	0,00	100,00	100,00
sspS	0,00	0,00	0,00	0,00	0,00	0,00	100,00	100,00
Q5HEL1	1,15	0,57	0,00	0,57	2,30	3,45	12,07	14,37
UPF0316	0,17	0,17	0,00	0,33	1,66	1,82	8,96	8,96
nadE	1,58	1,09	1,46	2,07	4,74	4,62	15,21	14,60
nadC	2,11	1,90	0,27	0,61	3,40	3,88	11,29	11,70
nos	0,00	0,93	100,00	0,37	8,54	4,55	11,33	11,42
pdt	0,50	0,75	0,75	1,01	12,45	4,91	11,95	11,70

Gene	vs. zoo28	vs. EMCR19	vs. SS60	vs. SS90	vs. NCTC13712	vs. MSHR1132	vs MW2	vs. SA17_S6
sdcS	0,19	5,50	1,15	1,02	5,31	5,76	12,41	12,35
Q5HEK3-pfbA	2,16	4,04	0,72	4,04	100,00	5,66	19,68	19,68
pncA	0,18	0,53	0,18	0,18	1,60	0,89	5,53	5,53
ppaC	0,54	0,43	0,00	0,22	4,62	4,62	6,02	5,91
aldH	0,58	0,87	0,72	0,51	3,55	3,62	14,42	14,64
Q5HEJ8	0,87	1,55	1,07	1,07	5,34	5,93	16,52	17,01
Q5HEJ7	100,00	1,49	1,99	1,74	4,73	6,72	14,93	14,93
A6U329	0,00	0,57	0,00	0,00	3,45	2,30	6,90	6,32
A6U330	0,36	7,63	7,18	7,18	14,45	5,12	12,66	12,66
Q5HEJ4	0,71	0,83	1,30	1,19	4,39	4,98	19,22	19,10
DUF1700	0,54	0,36	0,00	0,00	2,69	3,58	12,90	12,37
Q5HEJ2	0,19	0,19	0,19	0,19	2,67	13,52	22,86	23,43
Q5HEJ1	0,00	0,35	0,18	0,18	1,95	3,55	8,33	8,69
pmtD	0,81	3,64	0,67	0,40	100,00	12,94	13,34	100,00
pmtC	0,11	0,92	0,23	0,34	7,45	7,79	7,56	8,25
pmtB	1,47	0,88	0,59	0,59	14,54	11,89	14,24	14,68
pmtA	1,00	1,34	0,89	0,78	100,00	12,49	18,62	18,84
pmtR	0,79	0,79	0,79	0,79	12,60	3,94	12,34	13,39
A6U340	1,13	0,56	0,56	1,13	11,30	9,04	10,73	10,73
Q5HEI3	1,24	1,48	1,17	1,32	14,69	10,88	14,69	15,77
map	100,00	0,86	0,54	0,68	25,62	100,00	26,61	100,00
hlb	0,85	1,45	0,60	0,77	23,72	9,35	10,54	10,03
lukX	4,72	4,23	9,34	9,54	12,59	13,67	7,67	13,86
lukY	3,03	1,99	100,00	100,00	18,26	14,10	8,04	13,53
dapE	1,15	0,54	0,61	0,68	9,44	15,22	2,58	8,83
ktrB	0,99	0,76	0,99	0,99	3,82	5,12	0,76	5,58
groL	1,55	1,30	0,49	0,62	2,84	3,65	6,43	8,29
groS	0,00	0,00	0,35	0,35	0,35	0,70	0,70	2,46
ydiL	1,88	0,67	0,00	2,02	6,05	4,84	10,35	18,28
sdrH	1,98	100,00	0,92	2,52	11,37	9,53	15,26	100,00
Q5HEG8	1,12	0,96	0,96	0,96	5,10	4,94	13,88	14,04
mtnU-ramA	1,27	100,00	2,03	0,51	100,00	6,23	18,30	17,92
hld	0,00	0,00	0,00	0,00	0,74	0,74	0,74	1,48
agrB	8,29	100,00	100,00	8,29	17,86	8,93	26,32	22,17
agrD	5,63	100,00	100,00	4,93	14,08	2,11	26,76	6,34
agrC	3,79	100,00	17,87	3,94	9,16	5,15	22,10	9,08
agrA	0,14	0,98	0,56	0,14	2,65	2,37	6,83	7,25
scrK-frk	0,83	2,71	0,42	0,73	6,67	8,44	19,27	19,69
scrB	1,34	1,68	1,08	1,41	4,64	4,91	15,46	14,99
scrR	0,74	1,16	0,63	0,53	5,68	5,15	14,41	13,99
nrgA	0,48	1,12	0,40	0,48	4,88	4,00	13,99	14,07
Q5HEF7-tusA	0,89	1,33	0,89	0,89	100,00	4,00	11,56	12,44
yeeE-DUF395	0,37	1,48	0,65	0,56	8,61	7,04	17,78	18,15
rex	0,47	0,63	0,79	0,31	4,87	2,83	7,86	8,02
yheS	1,61	1,97	1,66	0,88	5,50	5,29	14,52	14,62
mutS3	1,55	2,36	1,92	1,80	5,83	6,33	18,19	19,24
tsaD	1,46	0,88	0,78	0,58	100,00	6,14	16,28	16,08
rimI	0,43	0,65	0,00	0,43	2,58	2,15	10,11	10,32
tsaB	1,36	100,00	1,81	0,45	4,52	4,98	19,91	19,00
tsaE	0,65	100,00	100,00	0,00	3,23	9,27	20,47	20,69
ilvD	1,42	1,95	1,60	1,66	3,26	7,10	11,96	12,02
ilvB	1,41	0,62	1,19	0,85	1,02	4,29	12,99	13,05
ilvH	1,96	1,18	0,39	0,39	0,78	3,92	12,16	11,76
ilvC	0,70	2,09	0,70	0,90	2,29	2,79	8,46	8,16
leuA1	1,05	0,78	100,00	0,59	100,00	4,11	11,69	11,37
leuB	0,76	100,00	100,00	0,76	4,20	4,96	14,50	14,41
leuC	1,31	1,02	2,84	1,17	5,47	5,32	11,08	10,86
leuD	0,52	0,70	0,70	0,52	4,36	4,19	9,25	11,87
ilvA2	2,13	1,97	1,73	1,89	4,33	100,00	5,67	10,24
sprL	3,26	1,09	0,65	100,00	6,74	6,30	13,26	100,00
yhgF	2,00	2,14	2,09	1,07	6,56	5,63	11,48	100,00
sigB	1,04	0,78	0,91	0,91	2,98	3,76	9,47	9,47
rsbW	1,04	0,83	1,04	1,67	3,13	2,71	8,75	7,92
rsbV	0,31	0,31	0,31	0,00	2,14	0,92	5,20	4,89
rsbU	0,30	0,40	0,20	0,30	2,69	2,99	9,88	9,58
mazF	0,28	0,55	0,55	0,55	1,93	1,65	6,61	6,34
mazE	0,00	0,00	0,00	0,00	0,58	1,17	1,17	1,17
alr1	1,22	2,44	0,87	1,57	4,00	3,66	15,58	15,06
acpS	1,11	1,67	1,67	0,56	6,11	4,72	17,50	16,39
A5IUL8	1,19	1,39	1,98	0,99	5,75	5,36	17,86	17,86
DUF304	1,75	100,00	1,31	0,56	7,44	11,25	20,31	19,25
Q9ZAH9	1,46	1,88	1,04	1,04	5,63	6,04	21,46	21,67
kdpC-chr	0,00	0,00	0,00	0,00	0,00	0,00	100,00	100,00
kdpB-chr	0,00	0,00	0,00	0,00	0,00	100,00	100,00	0,00
kdpA-chr	0,00	0,00	0,00	0,00	0,00	0,00	100,00	100,00
kdpF-chr	0,00	0,00	0,00	0,00	0,00	100,00	100,00	0,00
kdpD-chr	1,73	3,12	1,65	2,93	7,25	7,55	21,07	100,00
kdpE-chr	2,56	3,28	2,56	2,42	5,70	5,41	15,95	15,67
cshA	0,66	0,46	0,33	0,59	2,30	1,91	7,03	6,71
murF	1,17	1,10	0,66	1,17	6,15	6,37	20,15	19,78
ddlA	0,84	0,28	0,37	0,19	3,73	3,45	11,48	11,30
ftsW1-rodA	0,75	1,25	0,50	1,25	2,99	3,99	10,89	10,64
Q5HEB5	0,00	0,00	0,00	1,45	3,62	1,45	3,62	4,35
csoZ	0,48	1,43	0,48	1,43	5,71	6,19	17,62	100,00
csoR	0,68	0,68	0,68	0,68	2,04	2,04	7,48	7,82
cls2	1,21	1,62	1,14	1,08	4,44	5,39	11,78	11,92
yedJ	3,40	2,78	3,86	3,09	7,41	9,72	16,98	16,51
yidC	0,46	1,95	0,46	1,15	2,18	2,63	3,89	3,67
thiE	2,80	4,05	2,18	3,12	8,88	10,12	19,94	20,40
thiM	2,90	100,00	2,53	2,53	6,06	5,18	18,81	18,56
thiD2	1,68	3,85	2,05	2,29	100,00	5,17	16,13	15,88

Gene	vs. zoo28	vs. EMCR19	vs. SS60	vs. SS90	vs. NCTC13712	vs. MSHR1132	vs MW2	vs. SA17_S6
tenA	1,30	3,04	2,32	1,59	5,50	5,64	14,18	100,00
sceD	0,00	0,00	0,14	0,29	2,58	3,16	8,75	8,03
Q5HEA3	0,76	0,76	0,76	0,25	3,78	4,53	12,09	12,09
ywpF	0,23	0,91	0,00	0,00	3,40	2,04	9,75	9,98
fabZ	0,68	1,36	0,23	0,23	2,72	2,27	12,02	12,02
murA	0,55	0,55	0,39	0,39	3,16	3,40	10,58	10,19
ywzB	0,00	0,43	0,00	0,00	0,85	0,85	7,69	7,69
atpE	0,49	0,49	0,49	0,49	0,99	0,25	2,47	2,47
atpD	1,06	1,27	0,64	0,64	2,55	2,48	5,45	5,38
atpG	1,27	1,15	1,04	1,04	1,85	1,85	3,92	3,81
atpA	0,40	0,53	0,40	0,33	1,52	1,26	4,17	4,17
atpH	0,37	0,93	1,48	0,37	3,52	2,78	8,15	8,33
atpF	0,19	0,38	0,38	0,19	0,77	0,38	2,11	2,49
atpL	0,00	0,00	0,00	0,00	1,41	0,00	0,94	0,94
atpB	0,27	0,00	0,00	0,00	0,96	0,82	3,29	3,02
atpI	0,00	0,00	0,00	0,00	0,00	0,00	0,00	0,00
mnaA	1,68	0,44	0,26	0,18	3,44	2,91	14,37	14,46
upp	0,63	1,27	1,59	1,27	3,02	2,54	6,03	6,19
glyA	0,81	0,65	1,21	0,24	2,74	2,82	8,31	8,31
ywlG	0,38	0,00	0,19	0,57	1,71	2,10	11,62	11,81
ptpB	1,43	0,24	4,29	1,67	16,43	4,76	24,29	24,52
tsaC	1,32	0,76	0,76	1,32	100,00	6,62	18,26	18,92
prmC	2,51	2,27	2,99	2,51	13,98	6,57	19,35	19,35
prfA	0,84	1,30	0,93	0,93	3,81	2,97	10,58	10,21
tdk	0,33	0,33	0,33	1,00	1,33	1,67	6,00	5,83
rpmE	0,39	0,00	0,39	0,39	0,78	0,78	1,96	1,96
rho	0,84	0,53	0,99	0,84	5,69	4,48	6,83	6,91
aldA3	2,87	3,64	2,80	2,24	9,66	4,62	12,82	13,10
arxR	0,89	4,46	0,89	1,19	3,27	3,27	6,55	6,85
murZ	0,48	2,14	0,48	1,03	3,89	3,17	12,38	11,83
fbaA	0,70	0,58	0,70	0,46	1,97	1,28	4,18	4,53
DUF2529	2,49	0,57	1,72	0,57	4,21	4,41	13,41	14,56
pyrG	0,62	0,87	0,87	0,99	3,35	3,17	8,13	9,25
rpoE	1,13	0,19	0,38	0,38	1,32	1,32	0,75	7,34
Q5HE71	1,39	2,09	1,28	1,28	4,53	5,11	14,40	14,17
coaW	1,00	1,62	1,24	0,62	4,73	4,98	13,18	13,68
DUF2750	0,30	1,19	0,89	0,89	4,61	2,38	15,92	16,37
A5IUS7	1,67	2,01	1,17	100,00	17,04	8,27	18,71	18,80
hmrA	1,60	1,10	1,35	1,60	5,91	5,49	14,43	100,00
luxS	1,06	1,49	0,64	1,06	3,61	5,94	11,46	11,89
Q5HE65	1,71	2,28	0,28	0,28	6,27	6,84	16,81	100,00
pdp	1,92	2,00	0,77	0,84	7,68	6,22	14,67	14,36
deoC-L2	1,81	2,26	100,00	1,20	100,00	6,17	8,89	9,79
deoD-L2	0,70	0,70	0,98	0,42	3,23	3,66	7,45	7,88
dpsA	0,90	1,35	1,80	0,68	2,03	100,00	9,23	9,46
DUF393	0,00	1,45	0,96	0,00	100,00	4,34	11,33	100,00
Q5HE59	1,02	0,95	0,95	0,87	3,06	3,78	11,50	11,35
Q7A0B3	2,16	0,00	0,43	0,86	100,00	5,17	19,83	20,26
manA1	0,32	1,17	0,96	0,43	100,00	3,19	14,59	14,80
yhfK1	1,50	0,75	1,50	3,75	100,00	5,71	15,77	15,92
czrA	0,62	1,87	0,93	0,62	4,05	2,80	8,10	8,41
czrB	1,53	100,00	1,43	1,73	6,73	5,50	7,85	8,77
Q9ZB00	0,55	1,46	100,00	100,00	100,00	1,55	7,22	7,31
ylmA	2,30	4,34	2,43	3,70	7,92	7,54	18,52	20,18
glmS	1,22	1,50	1,16	0,72	3,88	2,99	6,98	7,25
mtlA	1,74	2,26	1,61	1,55	4,51	3,93	7,93	9,16
mtlR	1,63	1,95	1,40	1,40	16,05	8,66	17,59	17,87
mtlF	1,38	1,15	1,61	0,92	12,87	5,98	9,20	8,97
mtlD	0,81	100,00	1,72	2,08	12,10	6,50	12,65	12,56
sasB	0,47	0,48	0,48	0,58	100,00	18,78	19,51	23,44
glmM	0,15	0,37	0,22	0,37	3,69	4,50	10,91	11,14
ybbR-cdaR	0,43	1,39	0,43	0,86	3,86	4,18	11,90	11,36
cdaA-dacA	0,25	0,25	0,25	0,25	2,72	3,70	7,90	7,90
argI-rocF	0,66	1,43	1,10	0,88	100,00	11,10	18,35	100,00
salA	0,75	1,22	0,75	0,66	100,00	4,79	11,27	10,23
ycnB	2,28	1,87	1,87	2,14	5,67	6,64	16,60	100,00
sepA	0,21	0,42	100,00	100,00	5,66	6,71	17,61	100,00
sdrM	0,22	1,12	0,74	0,60	6,40	6,77	17,49	17,86
hlIII	0,58	100,00	0,44	1,17	4,53	4,09	14,62	15,35
Q5HE34-urtF	0,93	0,76	0,67	0,76	5,72	3,70	12,79	13,72
yvsG-ydjM	2,31	100,00	0,96	0,77	4,23	5,77	16,35	100,00
ynzG	0,38	0,38	0,38	0,00	2,30	1,15	2,68	3,83
Q5HE31	0,36	2,26	0,22	0,44	5,18	4,52	12,18	100,00
htsC-fecD	1,86	2,68	0,31	2,27	100,00	5,26	14,43	100,00
htsB	2,03	1,94	1,74	1,74	3,49	3,29	12,79	12,69
htsA	0,61	1,42	0,81	0,51	2,03	2,95	7,11	7,22
Q5HE27-sfnaC	0,84	1,87	0,84	0,93	4,30	3,73	16,25	100,00
rhbC1	1,36	0,96	1,19	1,02	6,86	5,16	17,91	17,74
Q5HE25	0,75	0,92	0,67	1,26	5,78	5,11	13,15	12,98
rhbC2	2,48	1,72	1,63	0,99	100,00	6,14	18,02	100,00
asp23	0,39	0,78	0,39	0,20	0,78	1,57	3,92	3,73
DUF2273	0,42	0,00	0,00	0,00	0,83	0,83	8,75	9,17
Q5HE21	0,91	0,73	0,73	0,73	6,01	2,73	9,29	11,66
opuD2	1,34	1,09	1,47	1,41	9,98	5,37	12,41	12,28
Q5HE19	1,39	1,49	0,99	1,19	16,87	7,24	16,96	17,26
qorA	0,60	0,30	0,40	0,70	8,38	6,79	14,57	14,07
DUF915	1,61	1,72	1,38	1,61	9,31	5,52	12,76	12,64
lacG	1,06	2,12	1,98	100,00	5,94	4,67	5,87	5,94
lacE	1,34	1,11	1,16	0,99	5,64	2,15	5,12	5,70
lacF	0,32	0,32	1,28	0,32	8,97	2,24	6,41	7,05
lacD	0,82	1,53	1,12	1,33	7,34	5,50	6,63	7,65

Gene	vs. zoo28	vs. EMCR19	vs. SS60	vs. SS90	vs. NCTC13712	vs. MSHR1132	vs MW2	vs. SA17_S6
lacC	1,07	7,82	0,96	1,93	12,11	4,61	11,58	11,68
lacB	0,58	0,97	0,78	0,97	4,65	2,52	5,23	5,04
lacA	0,23	0,47	0,93	0,93	3,03	1,40	3,03	2,33
lacR	1,78	2,73	0,95	7,47	14,95	11,03	11,03	10,44
cobB	3,78	5,67	4,05	3,37	10,26	8,23	8,91	9,45
Q5HE05	3,24	100,00	100,00	100,00	9,72	14,35	100,00	100,00
yvgN2	2,24	4,24	2,00	3,29	5,06	6,00	2,82	100,00
adhR	1,20	0,24	0,24	0,00	2,40	5,76	1,20	100,00
hysA-L2	9,04	10,52	100,00	7,36	100,00	100,00	100,00	100,00
eap-L2-eapH-1	1,41	2,58	3,05	2,35	5,16	7,51	5,87	100,00
alsD-L1	4,68	3,40	4,40	2,70	4,96	3,55	4,68	5,39
alsS	1,86	2,64	1,98	1,98	5,65	3,12	4,56	6,01
rpsI	0,50	0,75	0,75	0,50	0,75	1,00	0,50	0,50
rplM	0,00	0,00	0,00	0,23	0,46	0,23	0,00	0,00
truA	5,10	3,86	5,10	3,11	7,09	4,60	5,47	6,97
ecfT	2,97	1,86	2,97	1,98	7,81	6,07	8,30	8,43
ecfA1	1,86	3,14	1,86	2,44	9,52	6,97	11,27	11,73
ecfA2	1,73	1,98	1,73	3,09	100,00	4,81	14,20	13,70
rplQ	0,27	0,27	0,27	0,27	0,54	0,54	1,08	1,08
rpoA	0,21	0,11	0,11	0,11	0,11	0,11	1,38	1,59
rpsK	1,03	0,51	1,03	0,77	0,77	1,28	1,79	1,79
rpsM	0,55	0,55	0,55	0,55	0,55	1,37	1,91	1,91
rpmJ	0,00	0,00	0,00	0,00	0,00	0,00	0,00	0,00
infA	0,46	0,46	0,46	0,46	1,37	0,91	0,91	0,91
adk	0,15	0,31	0,15	0,15	0,62	0,93	2,62	2,62
secY1	0,54	0,39	0,54	0,62	0,70	1,31	4,02	4,10
rplO	0,00	0,00	0,00	0,23	0,00	0,45	0,45	0,45
rpmD	0,00	0,00	0,00	0,56	0,00	0,00	0,56	0,56
rpsE	0,20	0,20	0,20	0,20	0,20	0,20	2,59	2,59
rplR	0,28	0,00	0,28	0,00	0,83	0,83	1,39	1,39
rplF	0,37	0,37	0,37	0,37	1,49	1,30	2,23	2,05
rpsH	0,00	0,00	0,50	0,00	0,25	0,25	1,25	1,50
rpsZ	0,54	0,54	0,54	0,54	0,54	0,54	0,54	0,54
rplE	0,00	0,37	0,19	0,00	0,19	0,00	0,93	0,93
rplX	0,00	0,00	0,00	0,00	0,31	0,63	1,26	0,94
rplN	0,54	0,54	0,54	0,54	0,54	0,54	1,36	1,08
rpsQ	0,76	1,14	0,76	0,76	1,14	1,14	0,76	0,76
rpmC	0,00	0,00	0,00	0,00	0,00	0,00	0,00	0,00
rplP	0,46	0,46	0,46	0,46	0,46	0,46	1,15	1,61
rpsC	0,31	0,00	0,00	0,00	0,15	0,46	1,22	1,38
rplV	0,28	0,28	0,56	0,56	0,28	0,28	0,85	1,13
rpsS	0,00	0,00	0,00	0,00	0,00	0,00	0,00	0,00
rplB	0,12	0,12	0,12	0,12	1,44	0,48	1,56	1,56
rplW	0,36	0,36	0,36	0,36	1,81	0,72	2,90	2,54
rplD	0,48	0,32	0,32	0,32	1,44	1,12	2,40	2,56
rplC	0,15	0,60	0,30	0,30	0,90	0,30	0,75	0,75
rpsJ	0,32	0,32	0,00	0,00	0,97	0,97	0,32	0,32
Q5HDV6	1,03	1,54	1,03	1,28	5,90	4,87	13,85	14,10
pbuG	0,37	1,57	0,75	0,45	5,02	4,57	10,64	10,71
topB	2,11	2,15	2,43	2,43	6,60	6,04	17,32	17,18
Q5HDV3	1,69	1,13	0,90	0,56	5,74	9,23	17,00	17,12
glcU	0,46	1,04	3,13	1,16	4,75	4,86	10,76	11,23
Q1Y7Y7	0,99	1,21	2,09	0,66	5,06	6,60	13,86	100,00
Q5HDU8	0,31	0,94	0,94	0,94	1,89	1,26	10,38	10,06
acrB	1,23	1,67	1,36	1,48	4,61	5,71	13,51	13,76
fmhB	1,42	1,11	1,50	1,58	3,95	3,71	11,14	10,51
Q5HDU5	2,33	1,30	2,33	2,85	3,89	4,67	14,66	14,27
Q5HDU4	0,88	1,31	0,88	0,66	100,00	14,00	24,73	24,29
ybfD	0,99	100,00	1,15	1,07	100,00	5,93	20,26	100,00
sarV	0,00	0,28	0,00	0,28	1,99	0,28	7,69	7,69
moaA	0,49	1,56	1,47	0,88	5,08	2,74	14,76	15,05
mobA	1,00	1,17	1,00	0,67	4,50	5,83	16,17	16,00
moaD	1,71	2,56	1,28	1,71	4,70	5,98	13,68	13,68
moaE	0,22	1,79	0,67	0,00	3,36	4,03	12,53	12,98
mobB	0,21	1,03	0,41	0,21	7,61	4,73	18,52	18,93
moeA	2,38	1,98	5,00	1,83	5,56	5,71	17,22	17,06
moaC	1,01	1,21	0,81	1,21	4,44	3,84	15,96	16,57
moaB	0,99	0,99	0,00	0,00	4,93	5,13	18,34	100,00
moeB	0,99	100,00	100,00	100,00	3,97	4,87	15,99	15,89
modC	1,81	100,00	2,31	1,48	100,00	4,28	15,82	15,49
modB	1,34	1,49	1,79	1,79	3,13	3,42	9,82	9,97
modA	0,64	100,00	1,15	2,17	3,70	4,72	13,27	15,05
fdhD	1,50	1,75	1,50	1,50	4,99	6,24	16,35	16,35
Q5HDS6	0,78	1,55	0,78	0,91	100,00	9,20	22,28	100,00
bioY	1,08	1,62	0,54	1,08	4,50	5,23	15,86	15,50
rihB	1,70	2,12	1,91	1,59	5,10	5,84	17,52	17,73
fhuD2	1,54	1,54	0,77	1,43	4,07	6,27	8,91	9,57
caiA	1,21	1,82	1,65	1,39	6,06	5,80	16,36	16,71
utp	4,48	2,73	5,03	4,04	9,18	8,31	20,55	20,77
ureA	0,00	1,32	0,99	0,33	1,98	1,98	9,57	9,57
ureB	2,92	2,43	3,65	3,65	4,62	4,38	12,90	10,71
ureC	2,16	1,34	2,10	1,57	4,25	3,61	10,90	11,07
ureE	0,44	0,66	0,44	1,32	2,65	2,65	12,14	11,70
ureF	2,03	2,61	1,30	1,01	3,91	3,19	12,46	13,04
ureG	1,63	1,30	2,28	0,98	3,41	3,41	12,85	12,68
ureD	2,15	2,75	1,55	1,55	3,46	4,18	17,56	17,32
sarR	0,57	0,86	0,00	0,29	0,86	1,15	5,75	6,61
rsr	0,61	0,31	0,31	0,31	3,06	2,14	13,15	13,76
iraD	1,61	1,88	0,81	0,94	4,16	5,10	21,61	21,88
iraC	1,58	1,02	1,33	0,87	100,00	5,31	22,87	22,82
ssaA2	3,31	0,98	3,06	0,61	3,43	6,00	8,95	9,56

Gene	vs. zoo28	vs. EMCR19	vs. SS60	vs. SS90	vs. NCTC13712	vs. MSHR1132	vs MW2	vs. SA17_S6
nhaC	1,36	1,36	1,78	1,36	8,35	5,21	16,35	16,06
Q5HDQ7	0,65	0,92	0,65	0,09	3,23	3,42	12,19	12,00
Q2FEJ1	0,95	4,26	0,71	0,71	11,35	11,35	4,73	3,55
ssaA3	1,76	1,57	1,76	1,18	4,31	3,14	10,98	10,39
yvcT	0,31	0,84	0,31	0,21	5,14	5,45	9,96	10,27
Q8NV79	0,80	100,00	0,71	0,80	5,42	5,78	11,38	11,38
sagA-lytA	0,39	1,29	0,26	0,26	5,02	4,76	11,33	11,84
Q5HDQ1	0,00	0,00	0,00	0,00	7,94	1,19	3,17	10,71
yrhD	0,42	0,63	0,42	0,21	1,69	2,32	4,85	7,59
fdhL	0,47	1,35	0,85	0,58	4,91	3,72	6,29	7,38
lytR2-lcpC	0,63	3,67	0,73	0,63	6,50	4,82	10,17	9,33
suhB2	1,86	1,61	1,73	2,10	7,67	6,93	22,90	22,40
Q5HDP6	1,73	1,59	4,04	1,15	3,90	5,05	13,71	14,29
Q5HDP4	0,40	5,51	0,81	100,00	9,68	14,52	18,28	12,50
Q5HDP3	1,52	2,73	1,52	3,33	5,15	9,39	100,00	100,00
rpiRC	0,23	0,46	0,23	0,00	2,63	2,41	11,91	11,57
proY	1,23	0,94	1,23	1,16	1,67	3,41	10,87	100,00
Q5HDP0	2,23	2,51	1,68	2,79	5,03	6,70	21,79	20,67
Q5HDN9	1,08	1,61	0,54	1,08	4,30	4,84	10,22	9,14
Q5HDN8	0,00	0,52	0,52	0,00	4,17	4,69	20,31	20,83
glpW	3,62	2,99	2,99	2,99	8,81	8,65	17,14	17,14
yocS	0,11	0,76	0,43	0,43	100,00	2,71	18,51	18,40
Q5HDN5	0,56	0,75	0,38	0,56	3,58	5,65	15,07	15,07
glvC	0,81	1,31	1,87	1,12	4,49	4,49	11,84	12,15
glvR	2,09	3,40	3,40	3,40	5,10	6,27	13,86	14,38
Q5HDN2	0,39	100,00	1,18	0,59	100,00	3,15	11,61	10,63
nhaC2	1,64	100,00	1,71	1,00	100,00	6,20	14,81	14,32
Q5HDN0	1,74	4,87	1,74	1,74	100,00	7,65	11,83	21,39
yhxD-yghA	1,59	1,93	1,13	1,13	100,00	4,76	13,04	13,15
Q5HDM8-yxeP	1,78	100,00	2,23	1,25	7,49	9,09	18,98	19,07
hutI	1,05	1,53	0,89	0,97	4,28	5,08	12,75	13,08
hutU	1,08	2,29	1,32	0,90	4,21	3,55	11,19	11,25
hutR	0,90	1,92	0,68	0,56	6,21	5,08	13,22	12,88
fosB	100,00	100,00	100,00	1,18	5,41	14,35	100,00	100,00
hutG	1,60	1,50	1,18	1,50	5,24	2,88	8,76	12,29
lyrA	0,08	1,34	0,55	0,24	5,75	4,57	19,86	19,31
rpiA	1,02	1,02	1,31	2,62	4,37	3,64	12,37	13,25
yflK-yiiM	0,61	2,45	0,92	0,92	100,00	6,73	21,25	20,34
galM	0,49	0,88	0,59	0,59	3,82	4,90	13,53	13,33
ynfA	1,22	1,22	2,45	0,92	4,59	2,45	12,54	13,46
yhaP	1,30	0,73	1,30	0,81	7,22	6,49	14,36	14,03
yhaQ	2,56	1,33	2,00	2,33	14,11	12,33	4,67	5,67
Q5HDL3-yhaI	7,10	7,25	5,40	7,25	10,80	9,10	23,46	10,80
yxlJ	4,11	0,16	2,13	4,27	6,24	3,94	13,79	13,63
gltS	1,65	0,99	0,99	0,25	3,64	5,38	11,91	12,32
fni-idi2	2,00	0,38	0,10	0,29	5,05	5,52	15,05	14,76
corA	1,16	0,21	0,63	0,32	4,22	4,43	12,24	12,45
Q5HDK8	0,00	0,00	4,51	0,20	6,27	6,08	15,69	16,27
Q5HDK7	0,47	0,24	0,47	0,24	100,00	6,40	16,59	100,00
mlhB	0,32	0,43	1,30	0,43	4,00	5,95	14,39	14,29
Q5HDK5	3,29	1,88	0,47	2,66	5,16	3,29	3,76	4,23
semB	1,60	2,53	1,65	1,50	3,82	4,39	5,21	9,55
semA	0,46	0,93	0,46	0,62	1,54	1,54	1,85	7,10
Q5HDK2	1,08	0,90	1,08	1,08	6,13	5,59	13,15	12,79
tcaB	1,48	100,00	100,00	4,51	7,30	10,00	18,52	100,00
tcaA	1,37	3,76	100,00	3,18	4,55	4,34	15,32	100,00
tcaR	0,00	0,44	0,66	1,75	2,19	1,54	9,43	9,65
Q5HDJ7	0,82	1,44	1,75	2,37	100,00	4,63	15,95	16,26
hrtA	1,80	1,95	1,95	1,05	4,35	6,16	17,27	16,82
hrtB	1,80	3,79	1,61	1,70	6,34	4,45	16,76	16,29
hssR	1,19	0,44	0,74	2,37	10,22	5,19	15,85	15,85
hssS	0,80	7,86	1,02	4,29	7,86	8,66	13,32	13,90
lytT	0,45	4,28	2,25	3,60	7,43	5,86	5,63	5,63
Q5HDJ1	3,67	2,44	100,00	2,65	7,54	7,54	9,78	8,76
mqo	1,22	2,64	1,83	2,50	12,91	6,90	13,32	13,25
lctP-locus2	0,88	1,19	100,00	100,00	9,44	5,00	9,63	8,94
tagF	1,43	4,10	100,00	4,16	16,23	16,47	18,85	100,00
Q5HDI7	0,63	1,27	0,95	1,59	9,84	16,67	14,44	16,51
paiA	1,72	11,11	1,34	1,92	13,41	12,45	18,97	19,16
yhfP-A1KWX9	1,69	8,26	100,00	1,59	8,76	4,98	12,34	12,74
Q5HDI4	1,49	4,21	0,99	100,00	100,00	11,14	11,14	100,00
iruO	0,77	1,45	0,39	1,93	6,47	7,54	7,54	7,25
Q5HDI2	0,93	1,16	0,23	0,23	1,16	3,01	6,48	6,71
pip	0,55	0,86	0,78	0,23	4,15	3,13	11,03	100,00
Q5HDH9-tetR21	0,48	1,12	0,48	0,48	12,50	3,04	12,66	12,50
cobI	0,85	0,42	0,11	0,11	6,88	2,01	9,63	10,26
scrA	3,05	1,52	1,59	2,22	5,06	4,85	11,64	11,78
DUF1722	1,82	0,52	0,78	2,08	5,47	7,29	21,61	100,00
rsp-araC	0,81	1,00	0,90	0,62	3,32	3,70	11,97	12,16
ydaG	1,18	0,71	0,47	0,24	5,67	3,55	6,15	100,00
Q5HDH2	0,56	0,28	0,28	0,28	2,24	2,24	0,84	7,56
gltT	0,39	0,55	0,39	0,78	2,35	1,64	7,82	8,06
DUF3139	0,82	3,01	7,38	0,82	5,19	7,65	13,66	13,93
sarZ	0,67	1,12	0,67	0,67	2,01	2,01	12,98	13,20
hsp20	0,23	1,86	1,63	1,63	6,06	4,90	26,34	100,00
narK	0,46	2,09	1,08	1,78	100,00	100,00	100,00	100,00
nreC	0,92	1,83	0,31	0,46	14,22	8,10	14,22	14,83
nreB	0,68	100,00	0,68	0,97	20,58	3,19	20,58	20,39
nreA	1,10	1,10	1,10	1,10	12,58	1,77	12,58	12,80
narI	1,47	1,62	1,47	1,18	16,37	4,42	15,93	16,22
narJ	3,05	4,91	4,06	4,06	12,69	5,58	9,31	8,97

Gene	vs. zoo28	vs. EMCR19	vs. SS60	vs. SS90	vs. NCTC13712	vs. MSHR1132	vs MW2	vs. SA17_S6
narH	0,51	0,77	0,38	100,00	8,46	6,73	7,50	7,56
narG	0,76	3,69	0,60	1,41	9,43	6,91	5,83	7,75
nasF	0,31	4,19	0,51	1,12	100,00	12,47	12,27	12,88
nasE	0,32	0,95	0,00	0,63	7,94	8,89	9,84	9,52
nasD	1,33	2,74	1,54	2,24	10,14	9,64	6,28	7,81
nirR-nasR	0,27	0,82	0,27	0,27	11,60	11,60	10,64	11,05
Q5HDF4	3,35	100,00	2,23	1,49	5,02	6,13	10,41	14,87
focA-L2-nirC	0,85	1,33	0,85	0,73	3,03	2,55	1,58	13,70
Q8NV18	0,00	1,61	0,00	0,00	3,76	1,61	6,99	22,04
Q5HDF2	1,50	2,14	1,92	0,64	5,34	5,56	15,17	15,81
zinT	1,10	3,68	1,16	0,84	4,51	3,22	7,54	7,80
yoeB1	4,87	2,62	3,00	8,99	5,62	9,74	4,87	4,49
yefM1	1,98	1,98	1,19	3,57	8,33	7,94	8,73	100,00
dsbA	0,83	1,17	0,50	100,00	5,67	3,00	11,17	10,50
DUF4467	0,83	1,65	0,28	1,10	6,89	3,86	12,95	12,95
fmhA	1,76	4,56	0,96	1,12	6,00	6,79	13,51	13,27
tcyC	0,27	0,96	0,68	0,27	4,23	3,14	7,10	7,38
tcyB	1,23	3,43	0,69	1,23	3,98	6,31	9,05	8,92
tcyA	1,15	2,44	0,77	0,64	2,82	2,69	7,05	6,92
mdeA	1,73	100,00	1,18	1,46	100,00	9,49	17,12	100,00
gpmA2	0,29	3,78	0,44	1,16	100,00	3,49	9,02	9,46
cdf	1,50	2,54	1,50	1,15	4,15	6,81	15,11	13,38
sbi	5,63	1,63	100,00	5,55	15,84	10,51	11,92	14,43
hlgA	1,18	0,86	1,18	0,86	10,11	18,39	4,19	6,77
lukS	2,22	2,64	2,64	1,90	12,45	18,67	9,49	17,72
lukF	0,31	0,61	0,20	0,10	9,10	14,21	7,36	8,18
bioX	3,42	4,27	3,42	3,85	19,87	10,26	19,23	19,66
bioW	1,15	1,44	1,01	1,44	11,69	20,78	12,12	100,00
bioF	2,95	100,00	1,88	3,21	11,96	20,00	10,36	10,27
bioB	1,68	3,57	1,49	2,97	6,44	10,21	5,65	5,55
bioA	1,69	2,35	2,21	2,06	10,45	9,20	7,73	7,87
bioD	3,35	2,77	3,06	3,49	14,99	14,99	8,73	8,73
Q5HDC6-L1	100,00	1,76	100,00	100,00	100,00	100,00	100,00	100,00
msbA3	1,15	1,38	1,10	1,33	16,54	9,80	2,25	100,00
msbA4	0,90	1,07	1,24	1,41	12,39	7,58	1,81	100,00
Q5HDC6-L2	100,00	100,00	100,00	100,00	6,25	1,79	3,57	6,25
ldr_fst-L2	1,85	0,93	1,85	1,85	0,93	3,70	1,85	1,85
gtrA	3,10	2,58	2,33	2,33	4,39	4,91	3,88	100,00
glxK1	1,22	0,70	1,05	0,79	6,47	7,70	11,20	11,81
Q5HDC0	2,73	1,97	2,42	2,42	100,00	7,88	16,67	17,12
bcr	1,32	1,49	0,91	0,50	4,95	6,35	15,02	100,00
aarP	3,13	1,46	1,88	2,50	8,13	11,67	100,00	100,00
Q5HDB7	0,30	1,07	0,61	1,22	4,11	7,76	15,22	15,07
Q5HDB6	0,87	100,00	2,22	3,34	100,00	11,50	20,27	20,64
aapA3	1,77	2,91	2,48	100,00	9,29	4,68	11,84	11,91
nhaK2	0,87	1,11	0,91	0,67	6,88	5,58	16,64	100,00
ydaO	1,15	2,35	0,87	0,77	3,99	5,57	11,97	12,19
flp-fmtA	1,08	1,08	1,27	0,95	1,96	5,13	15,96	100,00
rfbD	1,29	1,06	0,70	1,41	5,05	4,46	10,80	11,03
panE1	2,77	100,00	2,24	1,17	100,00	8,86	17,08	16,97
mmr	1,21	2,14	0,86	1,21	13,20	6,35	14,56	13,92
opuCD	2,30	1,29	2,44	1,15	9,91	6,90	17,24	17,53
opuCC	0,96	1,38	1,06	1,27	5,31	6,26	14,33	14,12
opuCB	1,57	1,26	0,63	1,10	8,96	8,81	14,78	15,25
opuCA	1,29	1,78	0,65	1,61	11,06	7,67	11,30	13,56
ydeI-opuCB	3,33	2,00	1,17	2,17	100,00	6,83	10,17	9,33
ybeC	1,40	3,11	1,46	1,53	100,00	8,66	13,48	100,00
pnbA	1,33	1,40	1,92	1,92	9,46	8,50	14,63	14,86
pbuE	1,09	1,94	1,35	1,68	4,29	5,81	13,80	14,06
yjkA-ybbM	2,57	1,93	2,57	2,45	100,00	6,82	10,42	15,57
yjkB	0,75	2,11	1,06	1,51	3,47	3,77	1,81	16,14
pepA2	1,30	1,49	1,58	1,30	1,95	5,11	10,77	10,96
Q5HD92	0,43	0,65	0,43	0,43	3,66	2,80	15,27	15,05
yerD	0,13	100,00	100,00	0,25	100,00	100,00	100,00	100,00
Q5HD90	7,07	100,00	2,40	0,67	7,20	8,27	4,40	10,80
norD	1,01	1,26	0,92	0,75	8,79	3,69	9,05	9,13
cntF	0,93	2,80	0,53	0,27	9,87	4,40	9,20	9,73
cntD	1,84	2,08	2,08	1,35	11,52	5,88	8,46	11,52
cntC	1,26	1,61	1,84	0,80	8,16	4,37	8,28	8,62
cntB	1,60	1,60	2,14	2,14	4,59	4,17	3,21	3,63
cntA	1,13	0,81	1,13	1,50	4,82	3,25	4,69	5,07
DUF2338-cntM	1,69	1,84	1,92	1,23	16,02	5,75	7,36	8,43
fmrO	1,34	1,59	1,47	3,54	3,79	5,01	4,03	3,42
dapF	0,36	0,49	0,49	1,70	2,92	6,81	2,31	2,55
yxbG	1,83	2,08	1,71	1,83	10,50	11,23	12,09	10,13
Q5HD78	2,08	2,27	2,73	2,53	11,18	10,20	11,31	10,85
aphD	0,71	1,65	1,18	0,95	5,67	4,26	21,28	21,04
bdhA	0,00	4,45	0,00	1,87	8,19	2,73	4,74	5,32
iraB	0,35	1,06	0,35	0,71	9,93	4,61	1,77	10,28
Q5HD71	0,52	0,52	0,52	0,00	0,52	0,00	0,52	1,04
pgcA?	100,00	100,00	11,63	100,00	7,00	14,55	16,59	16,48
Q5HD60	0,00	24,47	4,26	0,00	23,40	6,74	23,76	23,76
Q5HD59	4,85	100,00	6,80	5,83	14,56	19,09	12,94	11,33
sasG	100,00	100,00	20,77	100,00	100,00	100,00	23,85	100,00
sarT	100,00	100,00	0,83	100,00	7,78	8,06	1,67	100,00
sarU	100,00	100,00	0,13	100,00	5,61	1,07	2,81	100,00
gtaB	1,27	2,65	1,85	2,08	3,81	5,65	3,46	3,81
fnbB	11,39	9,87	15,96	12,37	100,00	15,62	19,51	15,18
Q5HD52	100,00	100,00	100,00	100,00	100,00	100,00	21,88	21,09
fnbA	17,27	100,00	14,70	100,00	20,98	17,16	15,42	21,53
Q2G207	4,46	3,96	4,95	1,98	17,33	21,29	19,31	19,80

Gene	vs. zoo28	vs. EMCR19	vs. SS60	vs. SS90	vs. NCTC13712	vs. MSHR1132	vs MW2	vs. SA17_S6
gntP	2,87	3,90	3,31	2,43	11,48	10,45	9,35	9,49
gntK	1,74	2,45	1,42	1,67	100,00	10,55	11,26	12,03
gntR	2,64	1,17	1,62	1,62	10,28	10,57	8,96	9,40
merR3	1,91	100,00	100,00	1,91	100,00	17,32	15,92	100,00
relP	1,15	1,30	1,01	1,30	5,05	7,50	13,28	10,68
DUF2188	10,43	0,82	0,61	11,25	25,77	1,23	11,25	12,07
yccS	6,28	1,50	1,45	6,78	8,73	12,37	27,33	100,00
ycbE	0,78	0,78	0,63	0,55	100,00	3,83	1,41	1,64
dedA	0,98	1,31	1,63	1,14	2,94	3,43	3,27	3,43
stp	0,43	1,15	0,14	0,14	4,74	3,74	5,17	4,60
smp	0,39	0,26	0,13	0,26	4,15	4,02	6,23	5,32
fbp	0,97	1,02	1,17	0,87	6,97	7,33	1,68	7,33
Q1Y5K2	1,94	1,67	1,39	1,39	3,61	3,15	2,87	3,15
Q1Y5K1	1,52	1,35	0,67	1,01	100,00	5,22	1,35	5,05
mhqA-2	0,62	0,62	0,10	0,21	7,64	8,98	5,78	7,12
mhqR	0,00	0,46	0,23	100,00	6,21	4,14	4,83	5,06
Q5HD32	1,05	0,00	1,05	1,05	7,02	8,77	3,16	3,51
catE	2,35	2,85	2,11	2,23	6,44	6,20	3,59	5,45
frp	1,64	1,64	0,60	2,68	6,25	5,80	4,91	5,06
ldhD	0,50	1,01	1,21	0,40	2,72	1,51	1,81	1,91
ywtE	2,12	2,62	1,75	2,75	100,00	2,50	2,62	3,25
srtA	0,48	0,64	0,16	0,48	2,25	3,06	1,61	1,45
ywnH	1,83	1,63	1,63	1,22	100,00	9,15	9,96	1,63
sdaA	1,33	2,67	2,56	0,78	4,11	3,56	5,44	5,78
sdaB	2,79	3,23	2,20	1,91	6,02	6,75	6,02	5,87
ptsEIIC	0,67	1,44	2,68	0,96	4,02	5,65	3,26	3,74
Q5HD18	3,38	3,38	3,86	3,38	8,21	6,76	9,18	9,66
ydeD	1,18	1,60	2,03	1,39	15,28	7,48	14,85	15,49
aes	0,62	2,99	0,52	0,62	8,46	9,29	8,57	8,57
Q5HD15	3,75	6,34	100,00	100,00	100,00	11,24	11,24	12,10
Q5HD14	3,41	4,14	0,97	0,97	100,00	8,03	9,73	9,49
glcB	1,06	2,37	2,13	1,84	5,95	6,48	5,81	6,05
cidC-pox	0,46	1,38	0,86	0,86	3,74	8,33	3,22	7,47
cidB	1,30	1,01	1,30	1,30	3,91	4,78	7,97	9,28
cidA	0,25	0,00	0,25	0,51	0,76	3,03	7,83	8,08
cidR	0,23	0,91	0,80	0,91	7,96	8,76	8,76	9,90
Q2FV82	0,00	0,00	0,46	0,00	2,28	0,00	0,46	100,00
ssaA4	1,85	1,85	1,85	1,62	6,94	7,41	6,94	7,41
mvaA	1,17	1,64	1,17	100,00	11,62	7,10	9,36	12,71
mvaS	1,29	1,20	1,29	0,94	5,48	5,06	6,00	6,68
adaB	5,75	7,66	4,02	4,60	11,30	11,49	15,52	14,56
clpL	0,76	1,09	0,85	0,62	2,23	6,35	1,99	11,71
A8Z3E9	2,34	16,96	2,34	1,75	7,02	1,17	19,88	19,88
feoB	2,86	3,90	2,86	2,37	7,45	11,59	20,57	20,47
feoA	0,44	1,75	0,44	0,44	4,82	6,14	20,18	20,18
mmpL-farE	1,29	1,57	1,33	1,33	6,55	6,39	10,08	13,86
Q8NUR3	1,46	1,82	2,37	1,46	7,83	2,37	12,93	18,58
Q5HCZ7	5,36	9,52	6,55	5,36	16,07	10,71	9,52	9,52
rocA-pruA	0,52	2,39	1,29	0,52	5,37	5,11	6,67	6,86
maa	0,33	5,00	100,00	2,50	14,00	8,83	17,50	15,67
cwrA	0,00	0,00	1,56	0,00	7,29	6,25	6,25	7,81
copA	1,16	1,70	100,00	1,25	8,72	8,18	10,38	10,50
copZ	0,48	0,00	0,48	0,48	2,42	0,48	2,42	2,90
ddh	1,20	6,41	0,80	0,20	10,61	6,31	12,01	12,01
ywfG	0,87	0,78	1,39	0,35	8,57	5,80	14,20	14,11
crtN	1,66	2,19	1,39	1,66	12,19	100,00	11,79	100,00
crtM	2,20	0,92	2,08	0,92	14,57	100,00	13,06	100,00
crtQ	1,06	1,24	0,80	1,06	12,05	100,00	12,93	100,00
crtP	2,54	2,94	2,94	100,00	4,82	100,00	11,37	100,00
crtO	1,20	2,61	3,61	1,20	3,01	100,00	13,83	100,00
ssaA1	13,98	14,82	14,22	13,86	15,42	19,04	22,53	24,46
oatA	1,65	1,71	1,27	1,71	6,16	6,55	12,87	12,82
isaA	0,14	0,43	0,28	0,28	2,14	1,42	2,14	2,28
Q5HCY0	0,28	1,99	0,09	0,28	4,83	7,10	14,58	14,39
acrA	0,18	1,58	1,23	0,88	5,44	4,91	15,61	15,96
Q5HCX8	0,00	0,60	0,60	0,00	4,56	2,78	100,00	100,00
ynzC	0,87	0,43	2,16	0,87	4,33	5,19	14,29	14,29
Q5HCX6	1,32	1,85	2,38	2,38	5,29	5,56	16,14	16,40
nmrA	2,79	2,67	2,92	2,31	6,68	7,29	21,39	23,21
Q5HCX4	1,33	5,67	1,67	1,67	100,00	8,33	22,67	22,67
gbaA	6,43	0,68	0,85	0,68	5,08	2,88	17,60	18,10
gbaB	0,71	1,70	1,56	0,71	8,51	6,95	15,18	14,75
Q5HCX0	1,78	7,52	0,99	1,48	11,67	9,89	11,28	11,47
yraK	4,57	2,17	2,05	1,56	10,59	7,94	12,03	12,64
cobW3	0,90	2,36	1,46	1,57	10,33	12,79	5,39	7,30
Q6GDL8	1,56	100,00	100,00	100,00	5,63	11,04	5,26	5,04
Q5HCW6	2,44	4,25	3,52	2,08	6,05	5,60	4,79	4,52
DUF4176	2,35	2,35	100,00	100,00	3,69	3,69	4,03	2,35
Q8NUP1	3,49	2,69	4,30	100,00	7,26	6,72	5,65	5,65
Q8NUP0	4,30	0,36	3,58	3,58	3,23	7,53	4,66	4,66
Q8NUN9	0,58	1,73	0,92	0,81	5,42	5,77	3,81	4,61
pyrD	0,65	100,00	1,21	1,12	8,00	4,47	2,33	3,16
Q5HCW0	2,90	2,54	2,90	2,17	3,99	3,26	3,26	2,54
DUF208	0,83	1,80	0,97	1,52	3,60	4,56	3,87	3,73
Q5HCV8	0,22	8,67	6,89	8,44	8,89	9,78	9,33	8,22
pepX	1,13	100,00	2,79	1,13	8,79	5,94	5,17	5,35
panD	0,78	1,82	0,52	2,34	1,04	2,86	1,04	0,78
panC	1,41	1,88	1,06	3,05	4,11	4,11	3,40	3,17
panB	0,73	1,71	0,98	0,85	4,88	7,45	2,20	2,56
panE2	3,71	100,00	1,16	100,00	13,09	11,59	8,92	8,81
alsD-L2	100,00	0,00	0,00	0,00	100,00	100,00	100,00	100,00

Gene	vs. zoo28	vs. EMCR19	vs. SS60	vs. SS90	vs. NCTC13712	vs. MSHR1132	vs MW2	vs. SA17_S6
ldh2	1,35	1,88	1,88	1,67	5,42	4,90	4,90	5,10
yfnA1	1,52	0,55	0,41	1,59	10,56	4,62	10,42	10,21
yhxA-gabT	1,12	100,00	0,82	1,05	6,43	6,05	12,93	13,23
Q5HCU7	1,92	3,84	0,96	1,44	27,10	8,63	27,10	100,00
fda	0,22	1,12	0,22	0,56	2,02	2,58	1,80	4,26
lqo	0,33	0,60	0,20	0,33	2,67	1,40	2,94	3,27
acsA2-bclA	0,63	1,44	1,25	1,31	7,07	5,50	13,45	12,95
Q5HCU3	0,67	100,00	0,45	0,45	100,00	7,40	17,26	100,00
Q5HCU2	0,00	0,00	0,00	0,00	2,82	0,94	5,63	5,16
betA	0,64	2,87	0,64	0,29	3,22	4,15	9,47	9,42
betB	0,60	1,14	0,47	0,60	8,05	5,37	9,79	9,52
A8Z5A6	100,00	100,00	100,00	100,00	100,00	100,00	100,00	100,00
Q79ZX2	0,35	0,18	0,89	0,18	5,32	6,03	10,82	11,52
cudT	3,02	3,82	2,96	2,96	7,46	8,26	5,11	5,05
nrdG	0,56	0,93	1,12	0,74	4,84	5,03	12,29	11,92
nrdD	1,19	0,76	1,13	0,59	3,51	3,24	8,86	100,00
citM	0,36	1,24	0,36	0,29	3,57	4,08	14,51	14,22
cysG	2,44	1,32	2,34	2,44	100,00	5,18	16,77	16,46
cysJ	0,95	1,01	1,59	0,69	5,25	5,41	17,38	16,91
gpxA-L2	1,41	0,60	1,41	1,41	3,21	2,61	9,64	9,24
A6QKB0	4,37	4,37	3,97	5,16	6,75	5,56	17,46	16,67
nsaB	2,59	2,94	4,13	3,53	5,52	6,77	24,38	24,63
nsaA	3,40	2,75	2,22	1,70	4,31	6,01	18,82	18,69
nsaS	3,72	1,24	1,91	1,24	4,50	2,48	15,54	100,00
nsaR	1,20	0,90	0,60	0,60	4,80	3,00	16,52	15,47
Q5HCS2	1,00	6,47	1,99	1,99	5,97	9,45	28,36	27,86
phoB	1,23	100,00	1,42	1,11	7,22	5,06	13,26	12,95
DUF2648	0,00	0,00	0,00	0,00	0,98	0,00	2,94	3,92
slyA	0,44	0,88	0,88	0,66	2,85	1,10	4,61	5,48
estA	2,23	1,97	0,79	0,52	8,01	9,19	9,32	9,84
clfB	100,00	100,00	100,00	29,53	100,00	100,00	100,00	100,00
arcR	0,99	2,41	0,57	0,43	10,64	1,84	10,92	11,21
arcC-L2	0,85	0,85	0,64	0,74	9,02	4,99	8,92	9,02
arcD-L2	0,56	1,81	1,61	0,91	9,77	4,61	12,28	12,28
arcB-L2	0,89	1,98	0,59	0,69	4,15	3,36	11,28	11,37
arcA-L2	0,57	0,81	0,57	0,32	7,85	4,45	11,57	11,81
argR-L2	1,78	0,67	2,00	1,33	3,56	2,22	13,56	100,00
aur	1,50	2,22	1,05	1,05	4,25	3,27	13,27	13,53
isaB	22,28	21,77	20,92	21,09	23,81	23,47	100,00	100,00
Q6G635	5,00	5,22	2,17	2,61	4,78	8,26	28,26	28,48
manR	3,95	1,92	1,65	1,87	5,92	7,68	21,44	21,23
manP	1,73	2,04	1,83	1,94	5,25	4,74	18,25	17,99
manA2-yvyI	1,17	1,60	1,60	1,60	5,54	5,54	19,28	19,60
yhgE	1,04	1,04	100,00	100,00	4,32	5,40	14,95	100,00
scaH-lytZ	0,80	1,43	0,80	0,58	4,99	6,53	18,15	18,42
ywoC1	1,42	1,42	1,06	1,77	14,01	4,61	14,18	14,36
sasF	1,36	1,41	1,51	1,46	26,02	7,30	24,66	24,35
gtfB	1,32	1,32	0,74	1,40	8,82	5,22	21,40	21,32
gtfA	1,86	1,39	2,05	1,86	7,42	5,24	18,03	18,03
secA2	0,54	0,67	0,71	0,33	4,14	5,52	15,43	15,60
asp3	2,19	3,33	1,67	1,77	3,02	17,19	24,27	23,85
asp2	0,45	0,45	0,13	0,51	0,57	9,56	17,40	17,72
asp1	0,64	1,03	0,39	0,51	1,35	5,92	19,43	100,00
secY2	0,58	0,66	1,16	0,66	0,50	4,21	16,83	100,00
sasA	8,57	5,65	6,37	5,40	6,40	11,67	17,58	18,00
A6U540	0,58	1,44	1,59	100,00	7,20	8,65	1,73	100,00
Q5HCP0	19,49	0,00	100,00	0,32	0,00	12,14	100,00	100,00
Q5HCN9	0,56	2,22	0,83	6,11	8,33	6,11	3,06	5,56
ywrF	1,31	1,63	1,47	3,43	10,95	8,66	10,62	10,95
msrA3	2,31	2,31	1,68	1,68	11,11	11,11	11,32	12,79
Q5HCN6	0,40	2,38	100,00	0,40	11,31	11,51	13,89	11,31
capC-L2	0,65	0,78	0,78	0,91	7,52	2,46	10,89	10,89
capB-L2	0,43	0,72	100,00	0,87	3,61	2,74	9,96	10,25
capA-L2	0,90	1,06	0,45	0,45	2,71	2,56	10,26	10,41
icaR	0,53	1,25	0,53	0,53	3,74	2,50	11,76	12,30
icaA	0,89	0,65	0,32	0,40	2,50	3,31	8,96	8,88
icaD	0,98	0,33	1,31	0,65	5,56	3,27	13,07	13,07
icaB	0,34	1,60	0,57	0,57	5,37	5,25	14,04	14,50
icaC	2,18	1,42	2,09	0,28	4,37	3,51	14,06	11,49
lip1	6,96	7,35	3,89	6,13	10,46	9,88	9,64	9,83
hisI	3,79	3,48	4,27	4,27	6,95	6,95	3,95	4,42
hisF	1,84	0,92	1,98	1,58	6,46	5,01	6,19	5,93
hisA	2,41	2,70	3,40	2,84	9,79	8,79	8,37	8,23
hisH	1,90	100,00	5,87	0,86	8,12	8,98	8,12	9,15
hisB	1,38	9,33	3,63	2,42	8,12	8,46	8,46	7,77
hisC1	1,78	3,75	1,38	2,76	8,48	8,28	8,48	7,59
hisD	2,16	3,20	1,28	1,68	9,35	8,47	7,59	8,31
hisG	1,30	0,81	1,30	1,14	7,97	8,29	9,11	9,43
hisZ	3,30	3,42	3,17	100,00	5,49	6,47	16,24	15,87
Q5HCL6	1,24	100,00	0,44	0,18	5,41	4,17	12,15	12,94
Q5HCL5	100,00	100,00	100,00	0,38	11,79	17,87	19,39	100,00
yceI	1,16	2,13	1,74	1,36	3,10	3,10	2,33	5,23
drp35	2,37	1,85	2,47	1,44	4,94	9,36	11,32	9,88
Q5HCK8	0,63	1,04	0,63	1,04	7,11	7,42	7,31	6,27
pcp	2,35	2,03	2,97	2,03	10,95	10,49	12,36	11,27
Q5HCK6	0,39	1,69	0,65	1,04	10,92	5,46	100,00	6,37
padR	0,59	2,06	0,59	0,88	9,41	5,59	100,00	3,53
Q2FUS3	1,94	1,72	1,08	1,51	100,00	9,25	11,18	10,75
cna	0,00	0,00	0,00	0,00	0,00	0,00	100,00	100,00
Q7WS08	1,62	100,00	1,20	100,00	6,55	7,68	6,76	7,04
rarD	1,10	4,29	0,22	1,65	13,86	11,66	15,84	100,00

Gene	vs. zoo28	vs. EMCR19	vs. SS60	vs. SS90	vs. NCTC13712	vs. MSHR1132	vs MW2	vs. SA17_S6
Q5HCK1	4,17	3,30	1,56	3,99	24,31	7,64	24,31	24,48
nixA	0,39	5,01	0,39	0,39	7,57	10,03	6,98	6,69
Q5HCJ9-nhoA	2,09	2,09	2,21	4,05	7,24	9,08	15,21	17,42
hdeD	2,53	100,00	3,51	2,92	3,90	9,94	7,21	12,09
vraD	0,40	1,71	0,40	0,92	5,93	6,32	11,99	11,99
vraE	2,02	1,22	2,13	1,54	10,26	6,06	9,36	9,73
Q5HCJ5	1,04	2,08	1,04	1,56	9,90	7,81	6,77	9,38
cspC-L3-cspB	0,00	0,00	0,00	0,50	0,50	0,50	0,00	0,00
Q5HCI9-immR	4,21	5,09	4,04	7,54	6,84	6,67	9,30	3,86
DUF3147_L1	1,77	0,51	2,53	1,52	9,09	3,54	9,09	12,37
DUF3147_L2	2,26	2,82	0,85	0,85	13,84	10,73	11,58	11,30
noc	1,43	1,90	0,71	1,43	8,93	6,19	8,81	8,45
rsmG-gidB	1,11	0,83	0,56	1,94	7,08	5,00	8,61	9,31
mnmG-gidA	1,97	1,54	1,33	2,66	5,06	5,75	8,79	9,16
mnmE-trmE	0,65	1,67	2,03	0,58	2,97	4,13	9,78	9,78
rnpA	0,86	0,57	0,57	0,29	3,16	4,60	10,92	10,63
rpmH	0,00	0,00	0,00	0,00	0,00	0,00	0,00	0,00
MEAN DIFFERENCE:	3,77	9,54	7,12	7,11	14,40	8,45	14,61	20,09
MEDIAN DIFFERENCE:	1,02	1,38	1,02	1,02	5,22	5,05	11,73	11,99
